# Asymmetric C(sp^3^)–H functionalization of unactivated alkylarenes such as toluene enabled by chiral Brønsted base catalysts

**DOI:** 10.1038/s42004-021-00459-5

**Published:** 2021-03-16

**Authors:** Tsubasa Hirata, Io Sato, Yasuhiro Yamashita, Shū Kobayashi

**Affiliations:** Department of Chemistry, School of Science, The University of Tokyo, Hongo, Bunkyo-ku, Tokyo Japan

**Keywords:** Synthetic chemistry methodology, Stereochemistry

## Abstract

Benzylic functionalisation of unactivated alkylarenes remains as a significant challenge in asymmetric catalysis due to their less reactive nature. Here, we show development of catalytic asymmetric C(sp^3^)–H functionalization of unactivated alkylarenes such as toluene with imines. The reactions proceeded smoothly under proton-transfer conditions using a chiral, strong Brønsted base catalyst system. A chiral Brønsted base prepared from an alkylpotassium and a chiral amine ligand was found to effectively form a promising asymmetric environment around a benzyl anion. Optimization of the reaction conditions revealed that the use of the alkaline metal amide, potassium hexamethyldisilazide (KHMDS), as an additive was most effective, and enantioselective and atom economical carbon–carbon bond-forming reactions at the benzylic positions of unactivated alkylarenes was achieved without using any transition-metal catalyst.

## Abstract (web summary)

Benzylic functionalisation of unactivated toluenes remains a challenge in asymmetric catalysis. Here a chiral Brønsted base catalyses the enantioselective C(sp3)-H functionalization of unactivated alkylarenes, enabling carbon-carbon bond formation at benzylic positions of toluene derivatives.

## Introduction

Unactivated alkylarenes, such as toluene and xylenes, are inert and abundant materials that are stable, inexpensive, readily available, and easy to handle. While these compounds are common solvents in organic synthesis, they have also been employed as ideal feedstocks for the introduction of aromatic moieties into organic frameworks. For example, electrophilic substitution reactions on their aromatic rings in the presence of Lewis acids, represented by Friedel–Crafts reactions, are a well-known methodology^1,2^. On the other hand, although carbon–carbon (C–C) bond formation through catalytic activation of benzylic carbon(sp^3^)–hydrogen (C(sp^3^)–H) bonds is an efficient methodology, it is generally difficult because such C–H bonds are quite inert to many kinds of reagents. Moreover, its expansion to asymmetric catalysis is very challenging^3^. Only a few examples have been reported that describe enantioselective catalysis using alkylarenes directly as substrates. Since 2002, Davis’s group has been developing rhodium-catalyzed asymmetric C–H insertion reactions of diazoacetates into alkylarenes^4,5^. Melchiorre and co-workers achieved photocatalytic asymmetric C–H functionalization of alkylarenes using a chiral organocatalyst, but the reactions required at least 50 mol% of zinc triflate as a Lewis acid^6^. In 2019, the Jiang and Gong groups reported asymmetric photocatalytic benzylic C–H functionalization of alkylarenes using particular substrates, such as benzo[*d*]isothiazole 1,1-dioxides^7^ and *N*-tosylated isatins^8^. In these cases, however, the substrates were activated in advance and large amounts of the catalyst or specific structures of substrates were needed to achieve the desired reactions. Truly efficient catalytic asymmetric reactions of unactivated alkylarenes remains a very challenging target.

Chiral Brønsted base-mediated catalytic asymmetric C(sp^3^)–H functionalization through deprotonation of such benzylic C–H bonds offer a possible approach to construct C–C bonds with chiral centers. However, such benzylic C–H bonds are generally very inert to typical Brønsted bases, and stoichiometric amounts of strong Brønsted bases, such as butyllithium (BuLi) or its derivatives, are generally required to form the corresponding carbanions^9,10^. Thus, catalytic asymmetric variants have been recognized to be very difficult to develop. To address this issue, our group has been focusing on the development of strong Brønsted base-catalyzed asymmetric 1,4-addition reactions of weakly acidic substrates, such as esters^11^, nitriles^12^, amides^13^, alkanesulfonamides^14^, and alkylazaarenes^15^ (p*K*_a_ 30–35 in DMSO), using the product-base strategy^16^, in which strongly basic reaction intermediates were designed to promote a catalytic cycle. However, these methodologies have not been applicable for catalytic, highly enantioselective addition reactions of much less acidic substrates, such as alkylarenes (e.g., p*K*_a_ 42 in DMSO for toluene)^17^. Herein, we report on our efforts to develop catalytic enantioselective direct-addition reactions of unactivated alkylarenes with imines to afford optically active 2-arylethylamine compounds using newly designed chiral, strong Brønsted base catalyst systems.

## Results and discussion

### Reaction optimization

The reaction of toluene with *N*-*p*-methoxycumyl (PMC) benzaldehyde imine (**1a**) was first conducted in the presence of catalytic amounts of a chiral ligand, potassium *tert*-butoxide (KO^t^Bu) and lithium 2,2,6,6-tetramethylpiperidide (LiTMP), in which a species similar to potassium 2,2,6,6-tetramethylpiperidide (KTMP) might work as an active species (Table 1). In general, clathrate compounds, such as chiral crown ethers, have been employed for chiral modification of potassium ions because of their low Lewis acidity and large ionic radius. We applied (*R*,*R*)-binaphtho-34-crown-10 ether, which was used for chiral modification of a potassium ion in our previous work; however, decomposition of the chiral crown ether occurred during the reaction (see the Supplementary Table 1 in Supplementary Methods). To overcome this problem, we focused on chiral amine ligands, which are often used for chiral modification of a lithium ion. When 22 mol% of ligand **L1**, derived from phenyl glycine^18^, was combined with KO^t^Bu (10 mol%) and LiTMP (10 mol%), low but significant levels of enantioselectivity were obtained (Table 1, entry 3), whereas the reaction either without or with a lower amount of **L1** (11 mol%) did not proceed (entries 1 and 2). These results indicate that ligand **L1** could coordinate to the potassium ion and accelerate the addition reaction; however, when the reaction was performed with 11 mol% **L1**, it is possible that the ligand coordinated to the lithium ion preferentially and that a ligand-free benzyl potassium species existed that did not react with the imine. To decrease the amount of **L1** and to simplify the catalyst system by removing lithium ion, KCH_2_SiMe_3_, the conjugate acid of which is noncoordinative tetramethylsilane, was employed as a base catalyst. As expected, the desired adduct was obtained in good yield with moderate enantioselectivity (entry 4)^17^.

**Table 1:** Optimization studies.


Entry	Base-Cat.	Ligand	Yield (%)	ee (%)
1	KO^t^Bu-LiTMP	None	Trace	–
2	KO^t^Bu-LiTMP	**L1**	Trace	–
3	KO^t^Bu-LiTMP	**L1**^a^	92	36
4	KCH_2_SiMe_3_	**L1**	86	56
5	KCH_2_SiMe_3_	**L2**	82	40^b^
6	KCH_2_SiMe_3_	**L3**	95	30^b^
7	KCH_2_SiMe_3_	**L4**	61	6^b^
8	KCH_2_SiMe_3_	**L5**	90	31
9	KCH_2_SiMe_3_	**L6**	96	75

Reaction conditions (unless otherwise noted): **1a** (0.50 mmol), **2a** (1.0 mL), base catalyst (0.050 mmol), ligand (0.055 mmol), –78 °C, 18 h.

^a^**L1** (22 mol%, 0.11 mmol) was used.

^b^(*R*)-**3aa** was obtained as a major enantiomer.

The structure of the chiral amine ligands was then investigated. When chiral tetraamine ligand **L2** was employed^19^, which has much stronger, tetradentate coordination ability to a metal cation, the desired adduct was obtained in good yield with moderate enantioselectivity (Table 1, entry 5). Although several varieties of *C*_2_-symmetric chiral amine ligands^20^ were examined, the enantioselectivity was not improved (entries 6–8). Further structural optimization of ligand **L1** (see the Supplementary Tables 2 and 3 in Supplementary Methods) established that ligand **L6**, bearing a 4-methylpiperazine component, afforded the product in excellent yield with 75% ee (entry 9). Notably, successful chiral modification of a potassium cation using a chiral multidentate amine ligand is very rare.

To improve the enantioselectivity further, we focused again on the reaction using a mixed-base system (KO^t^Bu-LiTMP) and **L1**, which afforded a different enantioselectivity from the reaction using KCH_2_SiMe_3_ (Table 1, entries 3 and 4). We hypothesized that another metal species could change the asymmetric environment significantly and included a second potassium compound as an additive (Table 2). KO^t^Bu and KTMP were tested initially; however, these conditions did not improve the enantioselectivity (entries 1 and 2). Surprisingly, the inclusion of potassium hexamethyldisilazide (KHMDS) was found to be effective for this purpose, and the reaction gave the desired adduct with high enantioselectivities (entry 3). When cesium hexamethyldisilazide (CsHMDS), which has stronger basicity and cesium has a larger ionic radius, was employed; however, both the yield and the enantioselectivity decreased (entry 4). The structure of the alkyl substituents on the potassium disilylamide was then examined, and it was found that bulkier substituents decreased the reactivity significantly (entries 5 and 6). Although the actual role of KHMDS in the system remains unclear, we assume that KHMDS changes the asymmetric environment around the benzyl potassium species. In fact, KHMDS also accelerated the background reaction, which proceeded without the chiral ligand to afford the racemic adduct. This implied that KHMDS might affect an aggregation structure of the active benzyl potassium species (entries 7 and 8)^21^.

**Table 2:** Effect of additives.


Entry	Additive	*x*	*y*	*z*	Yield (%)	ee (%)
1	KO^t^Bu	10	10	11	Trace	–
2	KTMP	10	10	11	91	70
3	KHMDS	10	10	11	96	87
4	CsHMDS	10	10	11	33	9
5	KN(SiMe_3_)(Si^t^BuMe_2_)	10	10	11	79	86
6	KN(Si^t^BuMe_2_)_2_	10	10	11	25	67
7	None	10	0	0	Trace	–
8	KHMDS	10	10	0	36	–
9	KHMDS	10	5.0	11	92	86
10	KHMDS	10	20	11	92	85
11	KHMDS	10	10	22	94	84
12^a^	KHMDS	7.5	7.5	8.3	93	87

Reaction conditions (unless otherwise noted): **1a** (0.50 mmol), **2a** (1.0 mL), base catalyst, additive, **L6**, –78 °C, 18 h.

^a^Reaction conditions **1a** (1.0 mmol), **2a** (2.0 mL), KCH_2_SiMe_3_ (0.075 mmol), KHMDS (0.075 mmol), **L6** (0.083 mmol).

Next, the ratio of KCH_2_SiMe_3_, KHMDS, and ligand **L6** was examined. When 5 or 20 mol% of KHMDS was employed in the presence of 10 mol% of KCH_2_SiMe_3_ and 11 mol% of **L6**, the enantioselectivity decreased slightly (entries 9 and 10). Therefore, at least one equivalent of KHMDS relative to the chiral base catalyst was necessary to improve the enantioselectivity. However, when 22 mol% of ligand **L6** was employed with 10 mol% of KCH_2_SiMe_3_ and KHMDS for the chiral modification of all the potassium ions, almost no effect was observed (entry 11). Finally, the catalyst loading was decreased and we were pleased to find that the desired adduct was obtained in excellent yield with high enantioselectivity in the presence of 7.5 mol% of KCH_2_SiMe_3_ and KHMDS, and 8.3 mol% of **L6** (entry 12).

### Substrate scope

**Fig. 1:**
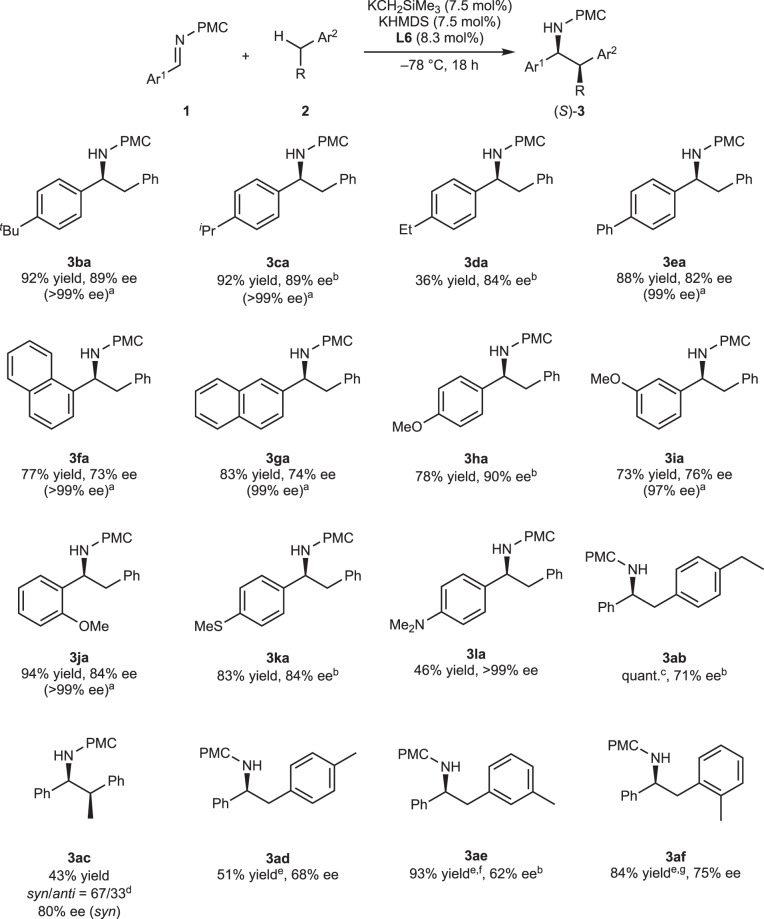
Scope of the reaction with respect to imines. Reaction conditions: **1** (1.0 mmol), **2** (2.0 mL), KCH_2_SiMe_3_ (0.075 mmol), KHMDS (0.075 mmol), **L6** (0.083 mmol), −78 °C, 18 h. ^a^After recrystallization. ^b^The enantiomeric excess was determined after transformation into the corresponding free amine. ^c^At −70 °C for 65 h. ^d^The relative configuration of the product shown in the table is defined as *syn*. ^e^Reaction conditions: **1** (0.50 mmol), **2** (2.5 mmol), KCH_2_SiMe_3_ (0.10 mmol), KHMDS (0.10 mmol), **L6** (0.11 mmol), Cumene (1.0 mL), −60 °C, 18 h. ^f^At −78 °C for 60 h. ^g^At −70 °C for 60 h.

The substrate scope of the reaction was then examined under the optimized reaction conditions (Fig. 1). When imines bearing tertiary or secondary alkyl substituents were employed, such as *tert*-butyl and isopropyl groups at the *para*-positions of their benzene rings, the desired adducts **3ba** and **3ca** were obtained in excellent yields with high enantioselectivities. Although an imine substituted with an ethyl group afforded high enantioselectivity, the yield of the product **3da** was moderate because this substrate has another reactive benzylic hydrogen atom, whose acidity was enhanced by the imino group on the aromatic ring. Additionally, imines bearing biphenyl or a 1-naphthyl or 2-naphthyl group afforded the corresponding adducts **3ea**–**ga** in good yields with good enantioselectivities. When imines bearing a methoxy group on the benzene ring were used, the reactions proceeded smoothly with good to high enantioselectivities to give **3ha**–**ja**. It was also possible to employ imines substituted with a methylthio or dimethylamino group at the *para*-positions (**3ka**, **3la**). Next, other alkylarenes were employed to further probe the generality of the reaction. The reaction of 4-ethyltoluene proceeded in quantitative yield with excellent regioselectivity and moderate enantioselectivity (**3ab**). Ethylbenzene afforded the desired product **3ac** with good enantioselectivity, although the yield and the diastereoselectivity were moderate. Unfortunately, cumene, which has a potential to form tertiary benzylic carbanion, did not react with the imine (**1a**) even at −60 °C because of its low reactivity (see the Supplementary Table 4 in Supplementary Methods). On the other hand, we hypothesized that cumene could be employed as a solvent in those reactions, because its freezing point is less than –78 °C (–96 °C), and alkylarenes with high freezing points could be employed in cumene solvent at low temperature. Based on this hypothesis, we tried to use xylenes in cumene solvent. In the cases of *ortho*-xylenes and *para*-xylenes, the reactions proceeded in moderate to good yields with moderate enantioselectivities (**3ad**, **3af**). *meta*-Xylene afforded the desired adduct **3ae** in excellent yield with moderate enantioselectivity. It is noted that the enantiopurities could be enhanced by recrystallization in several cases.

### Synthetic transformations and applications

**Fig. 2:**
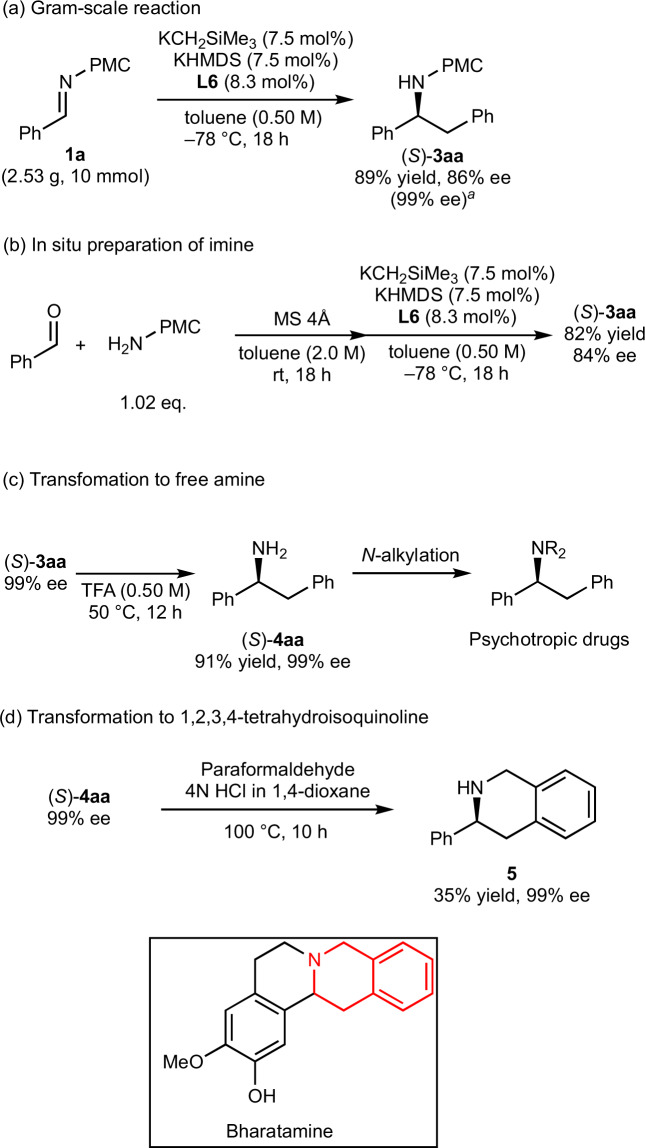
Synthetic utility. **a** Gram-scale reaction, Reaction conditions: **1a** (10 mmol), toluene (20 mL), KCH_2_SiMe_3_ (0.75 mmol), KHMDS (0.75 mmol), **L6** (0.83 mmol), −78 °C, 18 h. **b** In-situ preparation of imine, Reaction conditions: benzaldehyde (2.0 mmol), the corresponding amine (2.04 mmol), toluene (4 mL), KCH_2_SiMe_3_ (0.15 mmol), KHMDS (0.15 mmol), **L6** (0.17 mmol), −78 °C, 18 h. **c** Transformation to free amine and further derivatization. **d** Transformation to 1,2,3,4-tetrahydroisoquinoline. ^a^After recrystallization.

To demonstrate the utility of the approach, a gram-scale reaction was conducted, and the desired adduct was obtained without any significant decrease in either yield or enantioselectivity (Fig. 2a). The enantioselective reaction using an imine prepared in situ was conducted, and the desired reaction proceeded with slightly lower but promising enantioselectivity (Fig. 2b). Removal of the PMC group from **3aa** was achieved upon treatment with acid to afford the corresponding free amine **4aa** without loss of optical purity (Fig. 2c). It is known that the obtained free amine **4aa** can be further transformed into pharmaceuticals, such as lefetamine upon *N*-alkylation^22^. We conducted subsequent benzylation and methylation reactions and found that they proceeded smoothly without any significant reduction in optical purity (see the Experimental Section 2-6-4 in Supplementary Information). Furthermore, the free amine **4aa** could be derivatized into optically active 4-phenyl-1,2,3,4-tetrahydroisoquinoline **5** by Pictet–Spengler reaction (Fig. 2d). Although the corresponding transformation via *N*-formylation strategy was reported previously, we achieved the direct transformation in similar yield^23^. Thus, our reactions can be used as part of a simple synthetic route to 1,2,3,4-thetrahydroisoquinoline frameworks bearing a chiral center at the 3-position, which is an important moiety contained in natural products such as baritone. To our knowledge, asymmetric synthesis of **5** has only been achieved by hydrogenation of isoquinolines, for which high pressure (40 atm) of H_2_ gas is required^24^.

### Proposed mechanism

**Fig. 3:**
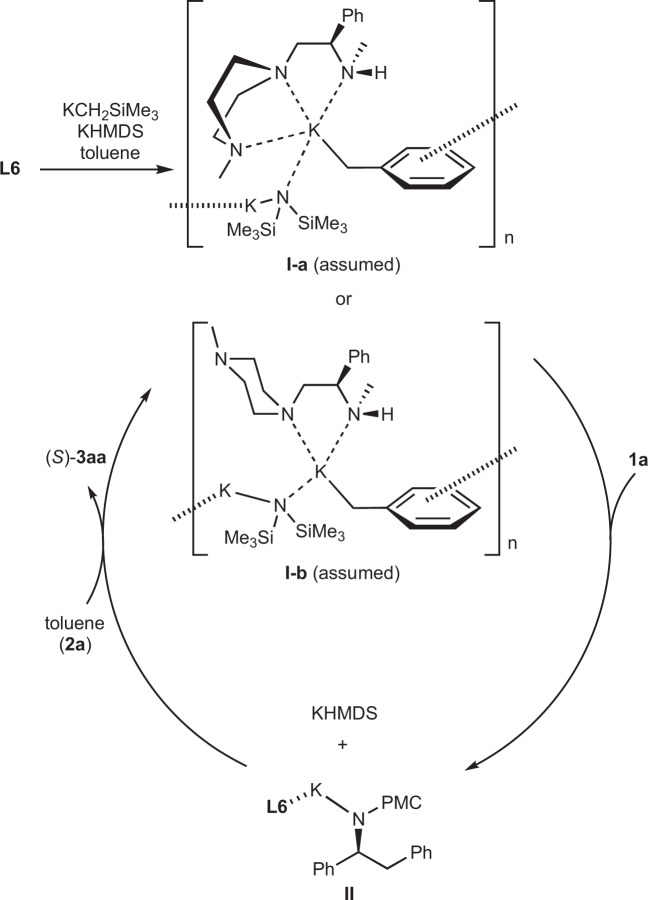
Proposed mechanism. Fist, oligomeric complex composed of benzyl anion, the chiral ligand, and KHMDS is formed. Then, nucleophilic addition which affords a strongly basic reaction intermediate undergoes after deaggregation. The intermediate deprotonates the next toluene to afford the desired adduct and regenerate the original oligomeric complex.

A plausible reaction mechanism for the addition reaction is shown in Fig. 3. Given that a slight nonlinear effect was observed (see the Supplementary Figs. 1, 2 in Supplementary Methods), chiral oligomeric species **I-a** or **b**, which consist of benzyl potassium, KHMDS, and chiral amine ligand **L6** in 1:1:1 ratio, might be formed^25–27^. The postulated ratio of the components was based on the results presented in Table 2 (entries 3 and 9–11) and on NMR experiments (see the Supplementary Figs. 3–5 in Supplementary Methods). Considering the large ionic radius and low Lewis acidity of potassium ion, it is generally harder to form boat-type coordination species **I-a**, and **I-b** is thus more plausible from a thermodynamic standpoint. However, the ligand bearing a 4-methylpiperidine moiety gave a similar result to **L1** rather than **L6**, which means that the additional nitrogen atom of **L6** might be effective in improving the enantioselectivity. Therefore, we cannot discard the possibility of formation of **I-a** (see the Supplementary Table 5 in Supplementary Methods). Upon addition of the imine, oligomeric species **I** is deaggregated and attacks the imine to afford strongly basic reaction intermediate **II**, which is termed the product base. The absolute configuration of the product is determined in this step (also see the Supplementary Fig. 6 in Supplementary Methods). Subsequently, product-base **II** deprotonates toluene to afford the desired adduct (*S*)-**3aa**. Simultaneously, chiral nucleophilic species **I** is regenerated, and the catalytic cycle is completed. At the current stage, an asymmetric environment of the active chiral catalyst complex with its detailed structure is not clear. Further mechanistic study is ongoing.

## Conclusion

In summary, we achieved catalytic asymmetric C(sp^3^)–H functionalization of unactivated alkylarenes using chiral, strong Brønsted base catalyst systems. The desired reactions proceeded smoothly in high yields and high enantioselectivities, and a chiral base prepared from an alkylpotassium and a chiral amine ligand formed a promising asymmetric environment. We achieved chiral modification of the potassium ion using simple diamine ligands and applied this cation to asymmetric reactions, although clathrate ligands were generally employed for chiral modification of the potassium ion due to its large radius and low Lewis acidity. These results expand the range of possibilities available for chiral alkali metal catalysis. Interestingly, the enantioselectivity was significantly improved using KHMDS as an additive. These reactions are synthetically valuable because both gram-scale reaction and successive asymmetric reaction via in situ-prepared imine were realized. The obtained adducts can be transformed into various useful compounds. It should be noted that this is an atom-economical carbon–carbon bond-forming reaction that takes place at an unactivated benzylic position of alkylarenes without using any transition-metal catalyst. Further investigations into the application of this chiral, strong base catalyst system to other reactions is ongoing in our laboratory.

## Methods

### Catalytic asymmetric addition reactions of alkylarenes with imines

Using a general procedure, KCH_2_TMS (9.5 mg, 7.5 × 10^−2^ mmol) and KHMDS (15.0 mg, 7.5 × 10^−2^ mmol) were placed in a flame-dried 10 mL flask inside a glove-box filled with argon. The flask was cooled to −40 °C, then amine ligand **L6** (13.0 mg, 8.3 × 10^−2^ mmol) in the employed alkylarene (0.80 mL) was added, and the chiral base mixture was stirred for 30 min at the same temperature. After the flask was cooled at −78 °C, *p*-methoxycumylimine **1** (1.000 mmol) dissolved in the employed alkylarene (1.20 mL) was successively introduced via a well-dried cannula, and the whole mixture was stirred for 18 h at the same temperature. The reaction was then quenched by adding a few drops of MeOH, the mixture was extracted with DCM (3 × 10 mL), and the combined organic layer was dried over anhydrous Na_2_SO_4_. After filtration and concentration under reduced pressure, the crude product was purified by preparative thin-layer chromatography to afford the desired amine **3**.

## Supplementary information

Supplementary Information

## Data Availability

For ^1^H NMR, ^13^C NMR spectra, and high performance liquid chromatography (HPLC) spectra, see Supplementary Figs. 7–156.
