# Supplementary material for: Asymmetric C(sp3)–H functionalization of unactivated alkylarenes such as toluene enabled by chiral Brønsted base catalysts
Source: Commun Chem. 2021 Mar 16;4:36. doi: 10.1038/s42004-021-00459-5 (PMC9814754; doi:10.1038/s42004-021-00459-5)
Supplement: Supplementary file 1 — Supplementary Information [file 42004_2021_459_MOESM1_ESM.pdf]

Asymmetric C(sp^3)–H Functionalization of Unactivated Alkylarenes such as Toluene Enabled by Chiral Brønsted Base Catalysts

Tsubasa Hirata, Io Sato, Yasuhiro Yamashita*, Shū Kobayashi*

Department of Chemistry, School of Science, The University of Tokyo, Hongo, Bunkyo-ku, Tokyo, Japan, 113-0033

Supplementary Information

Table of contents

1. Supplementary Methods 2
   1-1. Initial investigation of the ligand structures 2
   1-2. Optimization of the catalyst preparation conditions 2
   1-3. Optimization of the diamine ligand structures 3
   1-4. Limitation of Substrates 4
   1-5. Nonlinear effect 5
   1-6. NMR experiments 7
   1-7. Effect of the ligand structure (the piperazine part) 13
   1-8. Investigation of backward reaction 14
2. Experimental Section 15
   2-1. General 15
   2-2. Preparation of imines 16
   2-3. Preparation of chiral ligands 18
   2-4. Optimization 24
   2-5. Substrate scope 26
   2-6. Synthetic utility 33
   2-7. Mechanistic studies 40
3. References 42
4. NMR and HPLC charts 43

1. Supplementary Methods

1-1. Initial investigation of the ligand structures

Initial investigation of chiral ligand structures was conducted (Supplementary Table 1). A chiral macrocyclic crown ether (**L0**, 34-crown-10 ether) was not effective for the desired reaction (entry 1). On the other hand, chiral amines were found to be promising, and tetradentate ligand **L2** gave some level of ee in *tert*-butyl methyl ether (TBME) solvent (entry 2). In toluene solvent, the reaction with **L2** gave the product in higher yield but almost the same ee (entry 3). Finally, chiral diamine **L1** showed the most promising ee among them (entry 4).

Supplementary Table 1 Initial investigation of the ligand structures

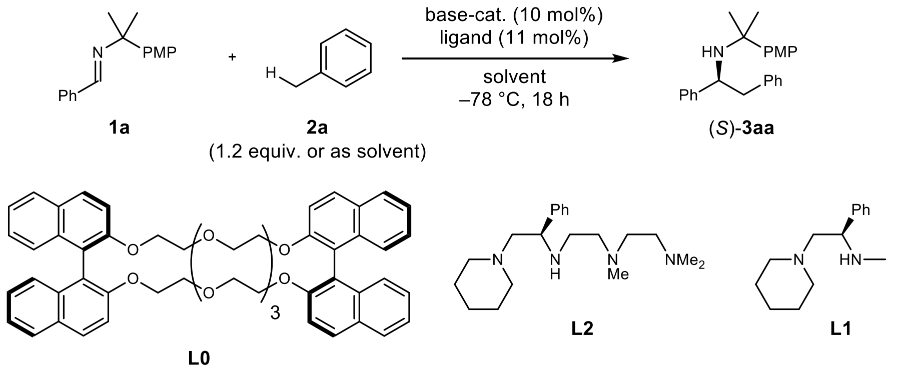

entry	base-cat.	ligand	solvent	yield (%)	ee (%)
1	KO^{t}Bu-LiTMP	**L0**	TBME	43	0
2	KO^{t}Bu-LiTMP	**L2**	TBME	46	12[a]
3	KCH_2SiMe_3	**L2**	toluene	85	11[a]
4	KCH_2SiMe_3	**L1**	toluene	25	21

[a] (*R*)-**3aa** was obtained as a major enantiomer.

1-2. Optimization of the catalyst preparation conditions

It is known that catalyst preparation conditions of chiral catalysts sometimes affect results of the asymmetric reactions dramatically. Here, catalyst preparation temperature and time were optimized (Supplementary Table 2). It was found that the preparation at –40 °C for 30 min gave the best result, and the ee was improved to 56% (entry 5).

Supplementary Table 2 Optimization of the catalyst preparation conditions

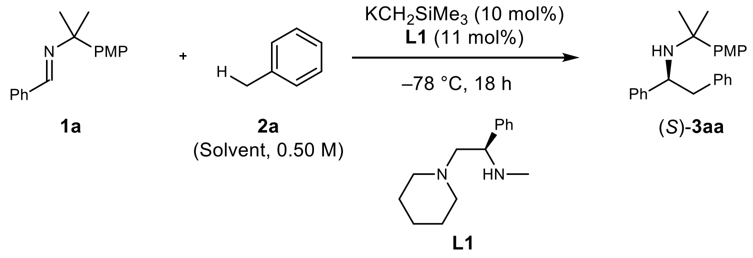

entry	conditions of pre-mixing	yield (%)	ee (%)
1	−78 °C, 30 min	25	21
2	−60 °C, 60 min	80	41
3	−40 °C, 60 min	88	50
4	−20 °C, 60 min	11	7
5	−40 °C, 30 min	85	56
6	−40 °C, 15 min	46	18

1-3. Optimization of the diamine ligand structures

In our initial investigation, ligand **L1** showed the most promising enantioselectivity. Therefore, we investigated effect of chiral diamine ligands with related structures (Supplementary Table 3). Firstly, diamines derived from other chiral amino acids were examined (**L7**-**L10**); however, the selectivity was not improved. Next, structures of the *N*-alkyl substituents were examined. It was found that longer alkyl groups were all not effective, and methyl group showed the highest ee (**L11**-**L13**). The piperidine part was then modified. A noncyclic structure, smaller and larger ring systems, dimethyl substitution and oxygen introduction at the 4-position were investigated; however, further improvement of the enantioselectivity was not observed (**L14**-**L18**).

Supplementary Table 3 Optimization of the diamine ligand structures

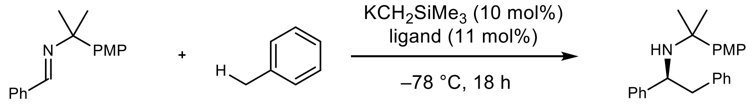

1a + **2a** (Solvent, 0.50 M) → (*S*)-**3aa**

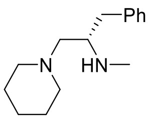

L7
No reaction

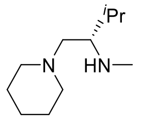

L8
94% yield, 16% ee[a]

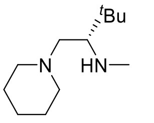

L9
89% yield, 2% ee[a]

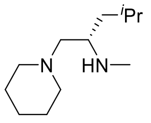

L10
87% yield, 13% ee

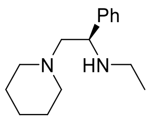

L11
54% yield, 7% ee[a]

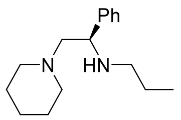

L12
62% yield, 8% ee

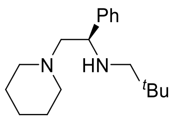

L13
93% yield, –21% ee

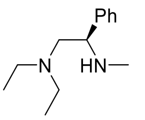

L14
22% yield, 31% ee

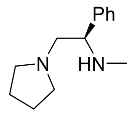

L15
61% yield, 28% ee

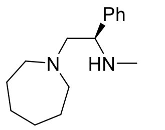

L16
85% yield, 47% ee

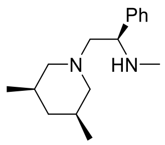

L17
94% yield, 43% ee

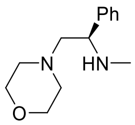

L18
No reaction

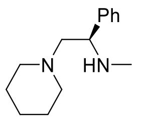

L1
86% yield, 56% ee

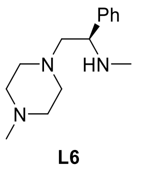

L6
96% yield, 75% ee

[a] (*R*)-**3aa** was obtained as a major enantiomer.

1-4. Limitation of Substrates

The reactions of the following alkylarenes that were not shown in the main text were conducted (Supplementary Table 4).

Supplementary Table 4 Reactions of other alkylarenes

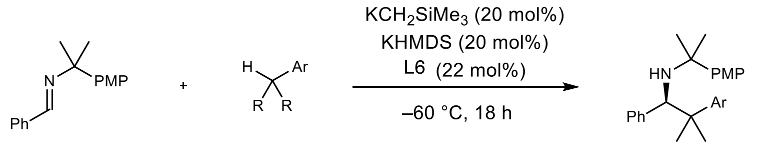

1a **2** **3**

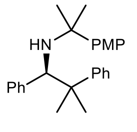

0% yield[a]
3ag

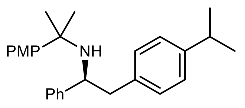

77% yield[a], 18% ee
3ah

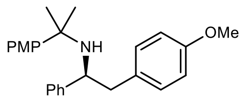

0% yield[b]
3ai

[a] Reaction conditions: **1** (0.5 mmol), **2** (1.0 mL), KCH_2SiMe_3 (0.10 mmol), KHMDS (0.10 mmol), **L6** (0.11 mmol), –60 °C, 18 h.

[b] Reaction conditions: **1** (0.50 mmol), **2** (2.5 mmol), KCH_2SiMe_3 (0.10 mmol), KHMDS (0.10 mmol), **L6** (0.11 mmol), Cumene (1.0 mL), –60 °C, 18 h.

When cumene (**2g**) was employed as a pronucleophile, the desired adduct was not obtained probably because of its low acidity or bulkiness at the reaction cite. Next, *p*-cymene (**2h**) was used as a pronucleophile. However, the reaction proceeded with low enantioselectivity. *p*-Methoxytoluene (**2i**), whose freezing point is −50 °C, is a challenging substrate because it is frozen under the optimized conditions. To overcome this problem, cumene was used as a solvent, but the reaction did not proceed presumably because of its low acidity.

1-5. Nonlinear effect

Relationship between optical purity of **L6** and that of the product obtained in the presence or absence of KHMDS was examined. When KHMDS was not used, almost linear relationship was observed (Supplementary Figure 1). On the other hand, when KHMDS was used, negative non-linear effect was observed but the level was not significant (Supplementary Figure 2). Those results could support that more reactive heterooligomer species of the catalyst (racemic) formed in the presence of KHMDS, but they did not deny formation of homooligomer species of the active catalyst in the reaction system.

Supplementary Figure 1 Nonlinear effect between optical purity of **L6** and that of the product (without KHMDS)

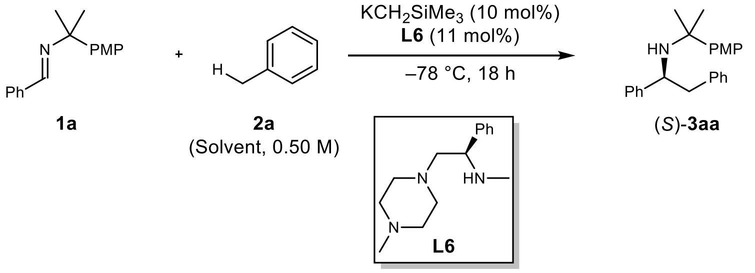

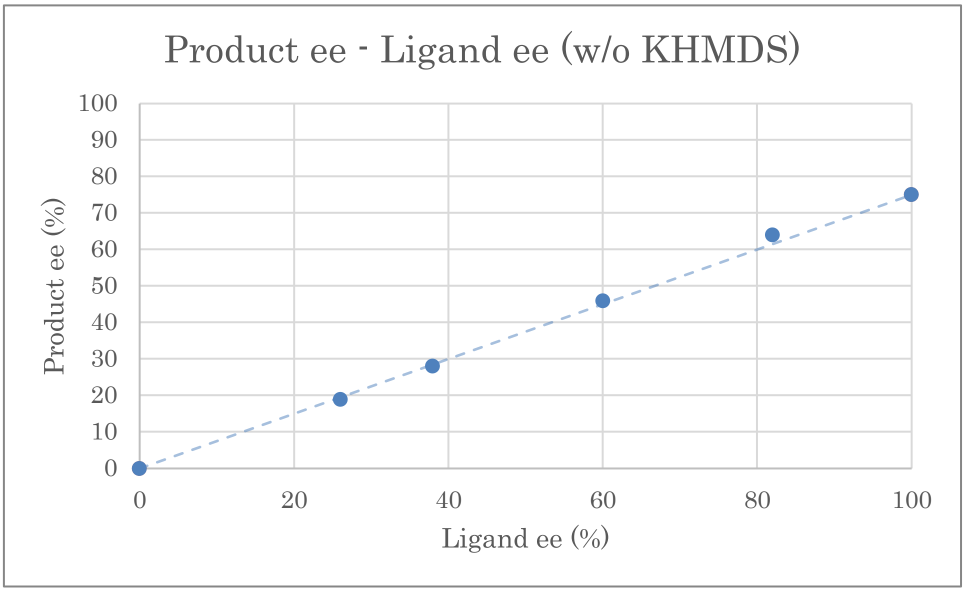

Supplementary Figure 2 Nonlinear effect between optical purity of **L6** and that of the product (with KHMDS)

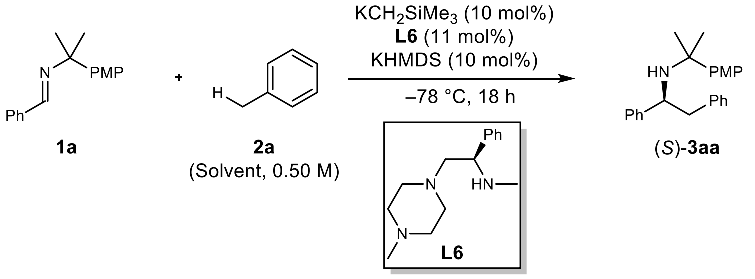

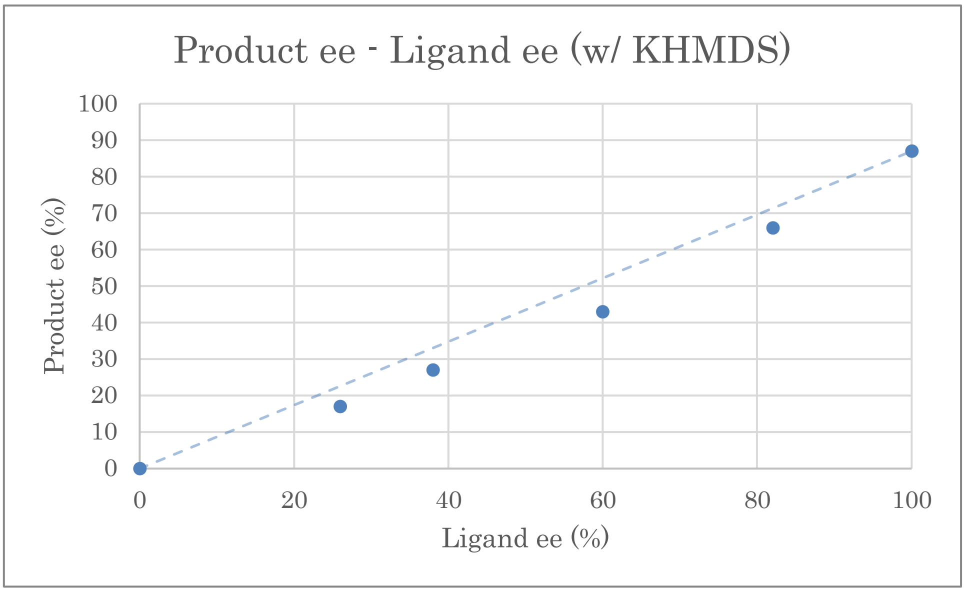

1-6. NMR experiments

NMR experiments were conducted to obtain information of the base catalyst system. Firstly, KCH_2SiMe_3 KHMDS, and **L6** were mixed in 1:1:1 ratio in toluene-d_8 to form an active chiral benzyl potassium species, and a spectrum was collected at –78 °C (Supplementary Figure 3). On the spectrum, remained free **L6**, a new set of peaks derived from **L6**, free KHMDS, and other new KHMDS species were observed. The ratio of the new KHMDS species (Me_3Si protons, 18 H), which might coordinate to the active catalyst, and a newly appeared species derived from **L6** looks almost 1:1 ratio. This result might support that the active chiral benzyl potassium complex is consisting of benzyl potassium, **L6** and KHMDS in 1:1:1 ratio; however, the spectrum was a little messy, therefore we also investigated the complex prepared from **L1**.

Supplementary Figure 3

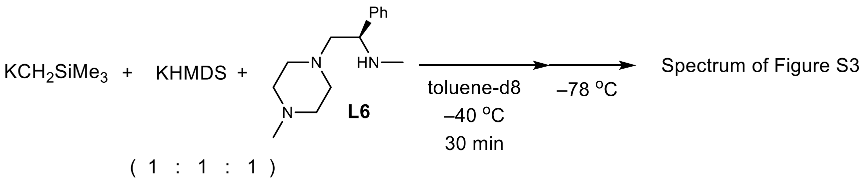

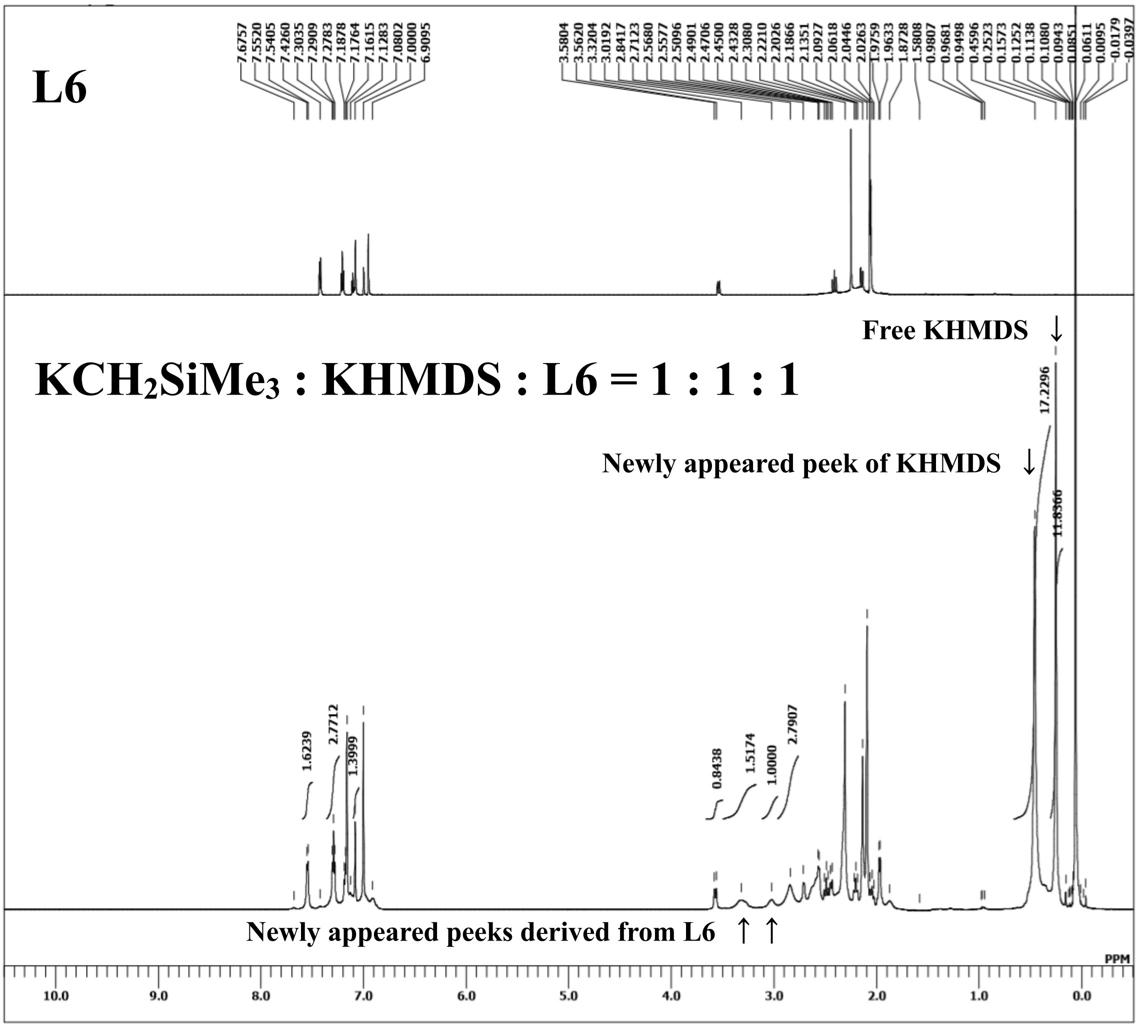

(expanded)

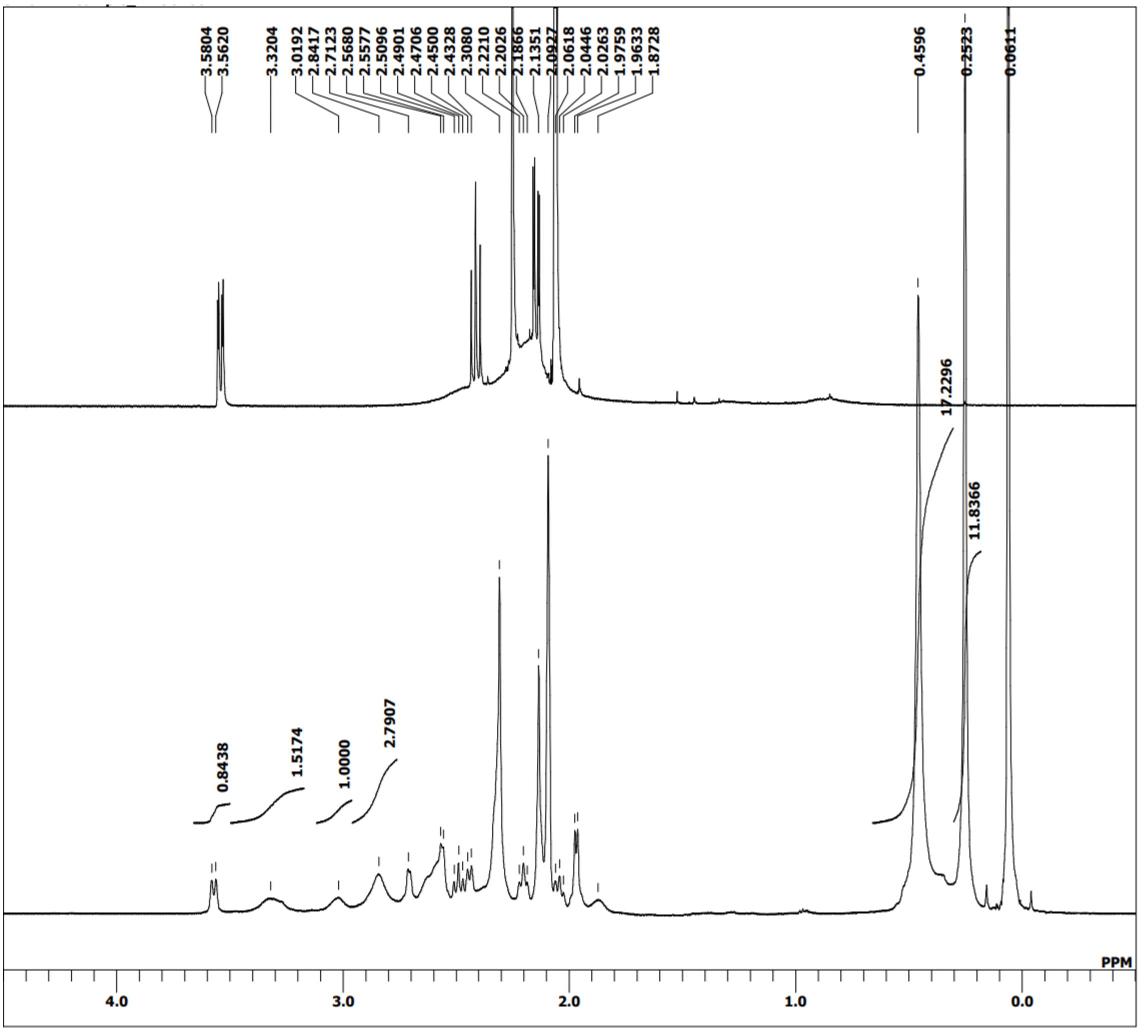

The spectrum of the NMR experiment using **L1** was shown in Figure S4. KCH_2SiMe_3 KHMDS, and **L1** were mixed in 1:1:1 ratio in toluene-d_8, and a spectrum was collected at –78 °C. Compared to the spectrum of the **L6** complex, a cleaner spectrum was obtained, and the ratio of the new KHMDS species (Me_3Si protons, 2 peaks) and a newly appeared peak derived from **L1** was almost 1:1. Furthermore, when KCH_2SiMe_3 KHMDS, and **L1** were mixed in 1:0.5:1 ratio in toluene-d_8, almost the same spectrum was observed, and the ratio of the new KHMDS species and the newly appeared peak from **L1** was the same (Supplementary Figure 5). Those results might support that the active complex is consisting of benzyl potassium, **L1** and KHMDS in 1:1:1.

However, the amount of the newly appeared species was small, and much amount of the free ligand was observed. This observation indicated the complex formation was not perfect, and the amount of the active species should be small in the reaction system.

Supplementary Figure 4

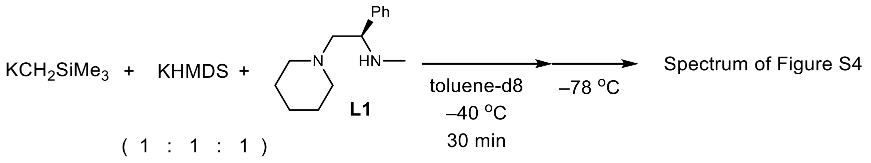

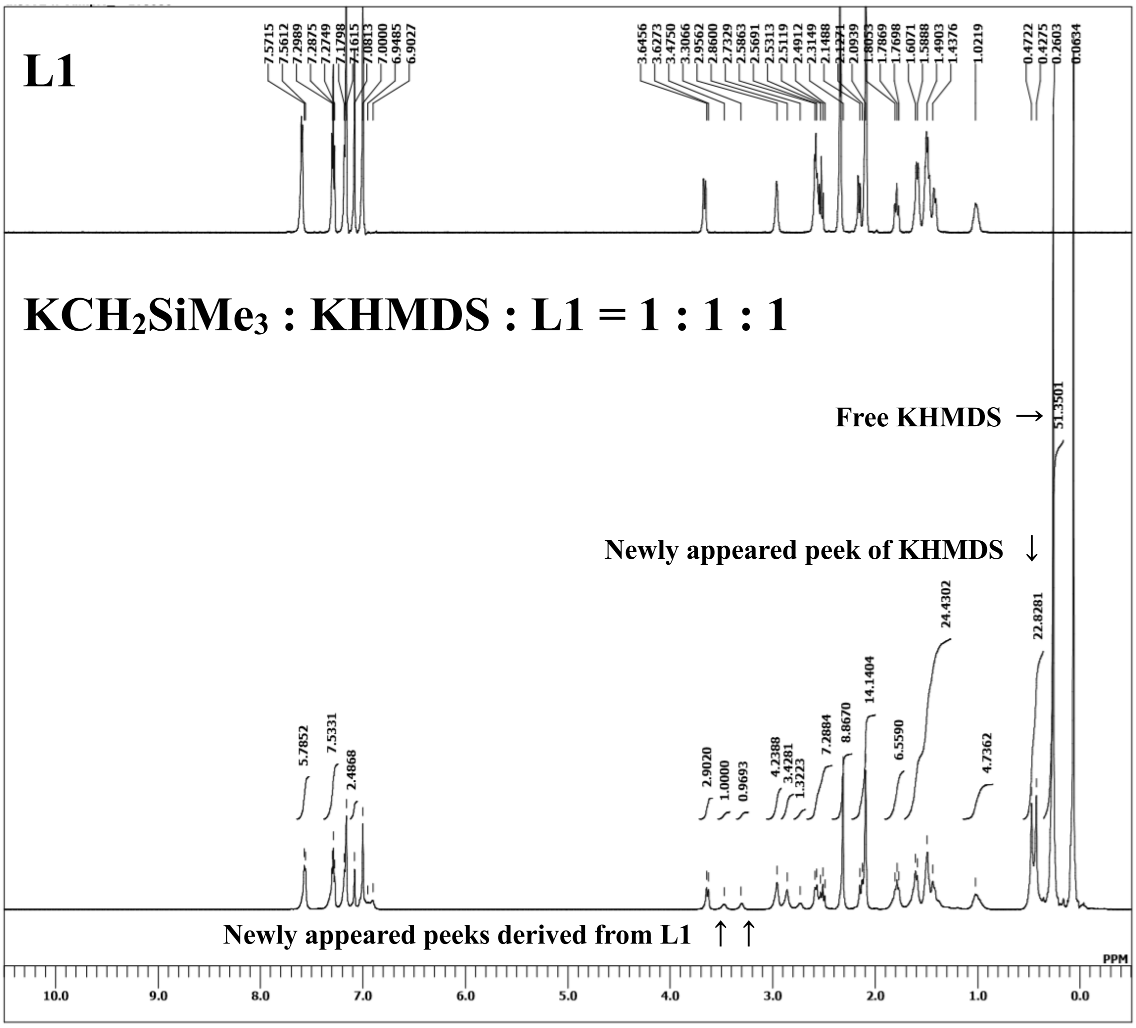

(expanded)

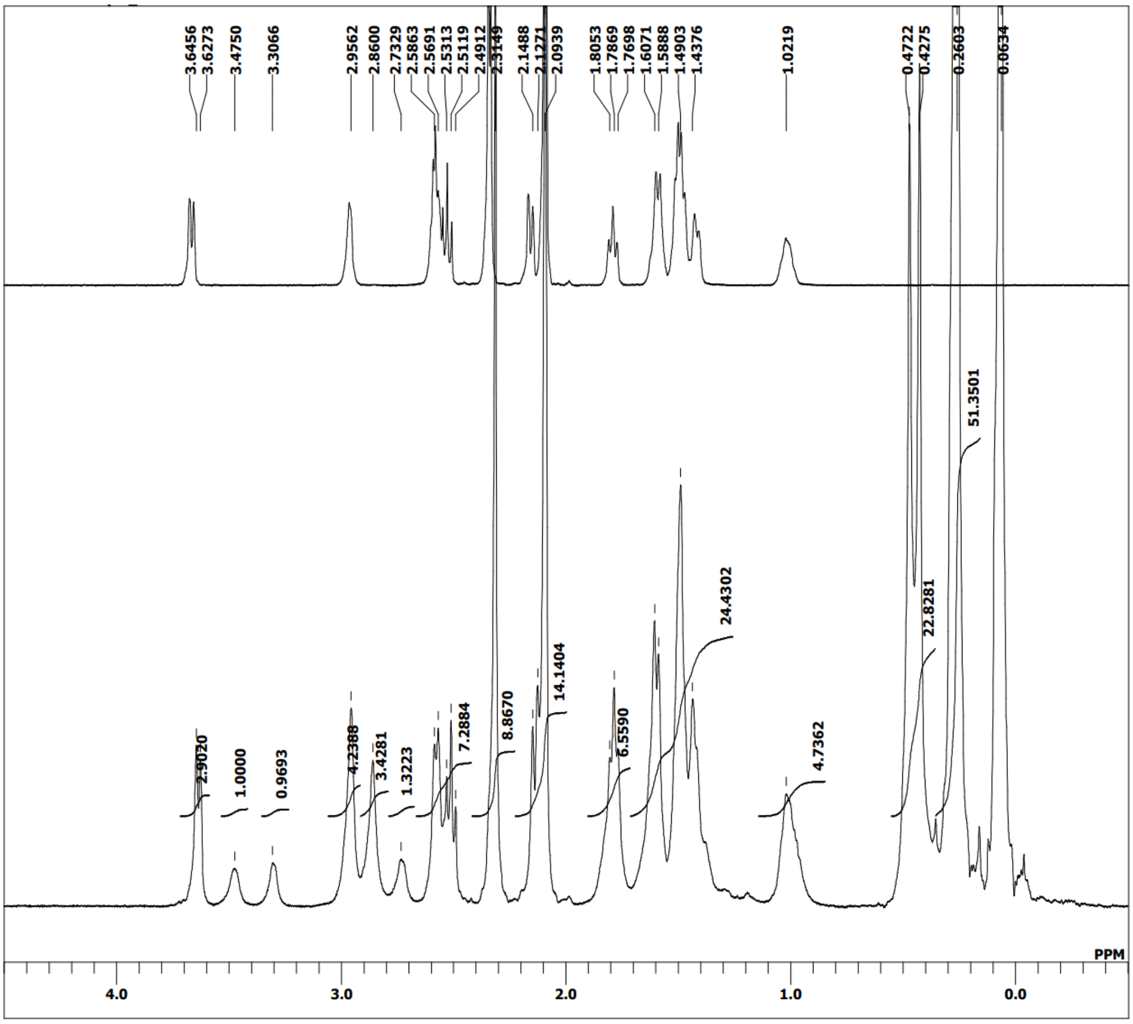
3.6456
3.6273
3.4750
3.3066
2.9562
2.8600
2.7329
2.5863
2.5691
2.5313
2.5119
2.4912
2.3149
2.1488
2.1271
2.0939
1.8053
1.7869
1.7698
1.6071
1.5888
1.4903
1.4376
1.0219
0.4722
0.4275
0.2603
0.0634
2.9020
1.0000
0.9693
4.2388
3.4281
1.3223
7.2884
8.8670
14.1404
6.5590
24.4302
4.7362
22.8281
51.3501
PPM
4.0
3.0
2.0
1.0
0.0

Supplementary Figure 5

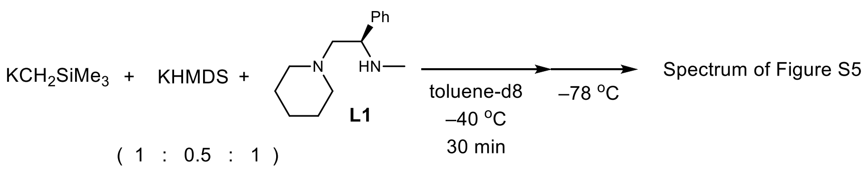

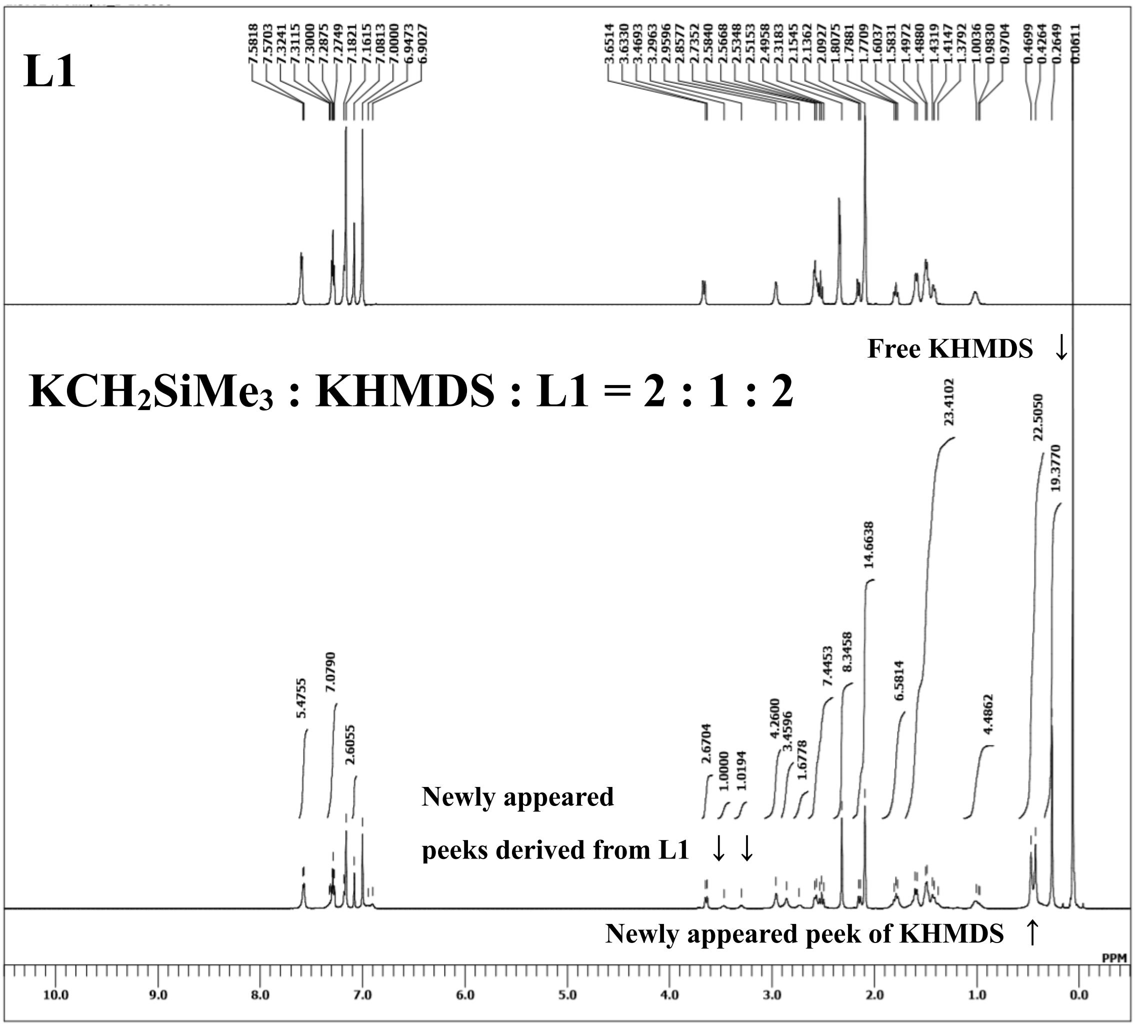

(expanded)

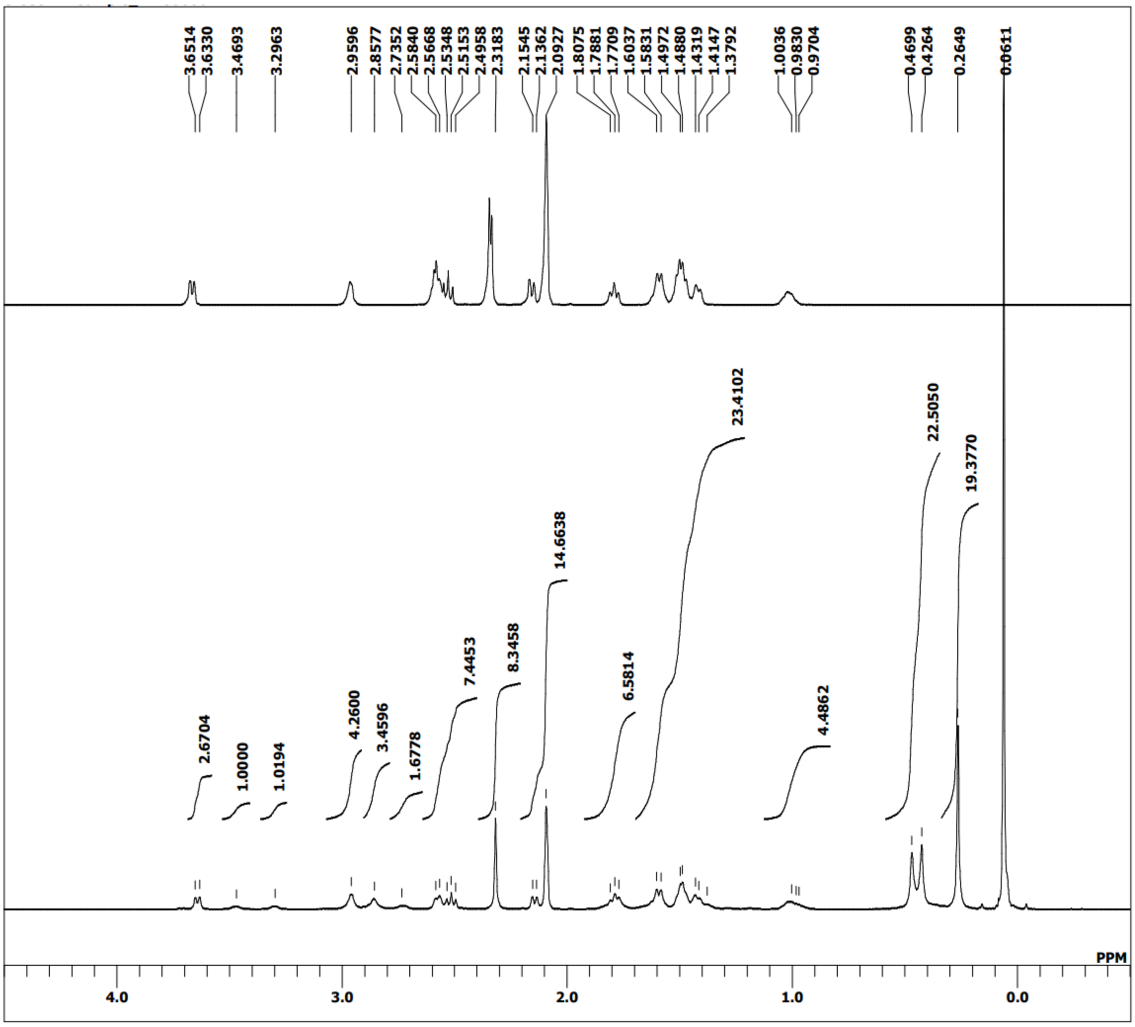

1-7. Effect of the ligand structure (the piperazine part)

Detailed investigation of the ligand structure about the piperazine part of **L6** was performed (Supplementary Table 5). When the methyl group on the piperazine part was changed into sterically larger alkyl groups, the enantioselectivity was not improved. However, enantioselectivities using the ligands bearing the piperazine parts was higher than that of **L1**. The ligand **L22**, which has 4-methyl group on the piperidine part of **L1**, gave almost the same result to the reaction using **L1**. Those results suggested that the nitrogen atom of the piperazine ring played an important role in the asymmetric environment.

Supplementary Table 5 Effect of the piperazine part

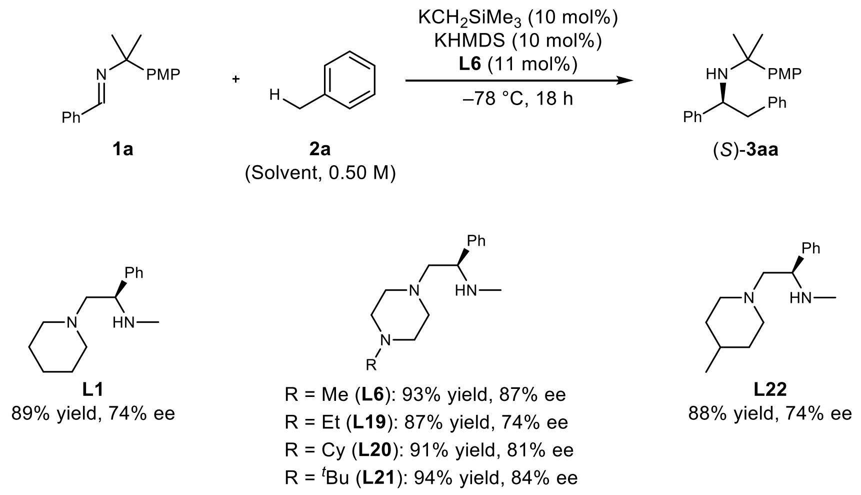

1-8. Investigation of backward reaction

Possibility of backward reaction was investigated (Supplementary Figure 6). When product **3aa** with 99% ee was treated by the chiral base catalyst for 18 h at –78 °C, the product was recovered in high yield with the same ee. This result indicated that the backward reaction in the process under the reaction conditions could be ignored.

Supplementary Figure 6 Investigation of backward reaction

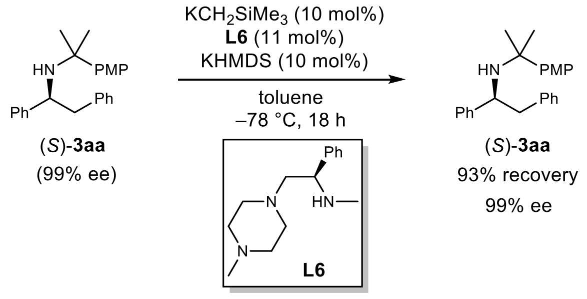

2. Experimental Section

2-1. General

Melting points were measured with Büchi Melting Point D-545. ^{1}H and ^{13}C NMR spectra were recorded on JEOL JNM-ECA500 and JNM-ECX600 spectrometers in $CDCl_3$ unless otherwise noted. Tetramethylsilane (TMS) served as internal standard (δ = 0) for ^{1}H NMR, and $CDCl_3$ served as internal standard (δ = 77.0) for ^{13}C NMR. IR spectra were measured on JASCO FT/IR-610 spectrometer. WILMAD screw-cap NMR tube (Aldrich Co., Ltd.) was used for NMR experiments. HPLC analysis was performed on Shimadzu LC-20AB, SPD-20A, and DGU-20A3. DART mass spectra were recorded on JEOL JMS-T100TD mass spectrometer. Preparative thin-layer chromatography (PTLC) was carried out using Wakogel B-5F. Potassium *tert*-butoxide (KO^{t}Bu) was purchased from Kanto Chemical Co., Inc. Lithium 2,2,6,6-tetramethylpiperizide (LiTMP), potassium bis(trimethylsilyl)amide (KHMDS), sodium bis(trimethylsilyl)amide (NaHMDS), and lithium bis(trimethylsilyl)amide (LiHMDS) were purchased from Aldrich Co., Ltd.. Trimethylsilylmethylpotassium (KCH_2SiMe_3) was prepared according to literature.[1] Potassium 2,2,6,6-tetramethylpiperidide (KTMP) was prepared by deprotonation of 2,2,6,6-tetramethylpiperiddine with KCH_2SiMe_3. Potassium bis(trialkylsilyl)amides were also prepared by deprotonation of the corresponding bis(trialkylsilyl)amine. Ethylbenzene was purchased from Tokyo Chemical Industry Co., Ltd. and was distilled and stored in an ampule. Toluene was purchased from Kanto Chemical Co., Inc. and purified further by Glass Contour NIKKO HANSEN & Co., LTD. TBME was distilled in the presence of benzophenone and sodium. **L0**,[2] **L1**,[3] and **L2**[4] ware prepared according to the literatures.

2-2. Preparation of imines

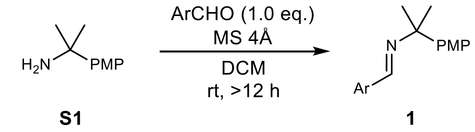

The imines ware prepared according to the literature.[5] *p*-Methoxycumylamine **S1** (10.0 mmol) was added to a solution of aldehyde (1.0 eq.) and MS 4 Å (5 g) in DCM (10 mL). The whole mixture was stirred for 12 h or more at room temperature, and then filtered through a Celite pad. The filtrate was concentrated under reduced pressure, and the crude product obtained was purified by distillation or recrystallization to afford the corresponding imine **1**. Structures of **1a-1c**, **1g-1j** and **1l** were confirmed by comparison with data shown in the literature which our group have reported[17].

(*E*)-1-(4-ethylphenyl)-*N*-(2-(4-methoxyphenyl)propan-2-yl)methanimine (1d);

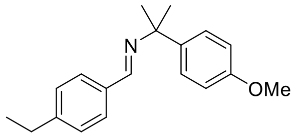

Colorless oil; ^{1}H NMR ($CDCl_3$, 500 MHz) δ: 8.13 (1H, s), 7.68 (2H, d, *J* = 8.50 Hz), 7.34 (2H, d, *J* = 9.07 Hz), 7.23 (2H, d, *J* = 8.50 Hz), 6.86 (2H, d, *J* = 9.07 Hz), 3.80 (3H, s), 2.67 (2H, q, *J* = 7.56 Hz), 1.62 (6H, s), 1.24 (3H, t, *J* = 7.65 Hz); ^{13}C NMR (150 MHz, $CDCl_3$): δ = 157.9, 157.0, 146.9, 140.3, 134.6, 128.1, 128.0, 127.3, 113.4, 62.1, 55.2, 29.9, 28.8, 15.5; IR (neat, cm^{-1}); 798, 827, 977, 1034, 1180, 1244, 1298, 1509, 1609, 1644, 2966; HRMS (DART) calcd for $C_{19}H_{24}NO$ [M + H]$^+$ 282.18579. found: 282.18523.

(*E*)-1-([1,1'-biphenyl]-4-yl)-*N*-(2-(4-methoxyphenyl)propan-2-yl)methanimine (1e);

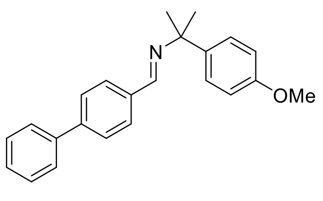

Colorless solid; Mp: 88-89 °C; ^{1}H NMR ($CDCl_3$, 600 MHz) δ: 8.19 (1H, s), 7.84 (2H, d, *J* = 8.25 Hz), 7.63-7.61 (4H, m), 7.43 (2H, t, *J* = 7.56 Hz), 7.36-7.33 (3H, m), 6.88 (2H, d, *J* = 8.94 Hz), 3.79 (3H, s), 1.65 (6H, s); ^{13}C NMR (150 MHz, $CDCl_3$): δ: 158.0, 156.7, 143.1, 140.6, 140.2, 135.9, 128.8, 128.5, 127.6, 127.4, 127.2, 127.1, 113.5, 62.3, 55.2, 29.9; IR (neat, cm^{-1}); 418, 558, 769, 811, 832, 1035, 1246, 1508, 1559; HRMS (DART) calcd for $C_{23}H_{24}NO$ [M + H]$^+$ 330.18524. found: 330.18627.

(*E*)-*N*-(2-(4-methoxyphenyl)propan-2-yl)-1-(naphthalen-1-yl)methanimine (1f);

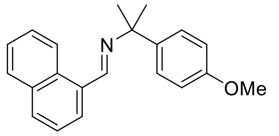

Colorless oil; ^{1}H NMR (500 MHz, $CDCl_3$) δ: 8.80-8.78 (2H, m), 7.90-7.88 (3H, m), 7.55-7.51 (3H, m), 7.42 (2H, d, *J* = 9.07 Hz), 6.91 (2H, d, *J* = 8.50 Hz), 3.82 (3H, s), 1.74 (6H, s); ^{13}C NMR (125 MHz, $CDCl_3$) δ: 158.1, 157.1, 140.0, 133.8, 132.4, 131.4, 130.6, 128.6, 128.1, 127.5, 126.9, 125.9, 125.3, 124.2, 113.5, 63.1, 55.3, 30.1; IR (neat, cm^{-1}) 2970, 1637, 1611, 1508, 1242, 1177, 1034, 828, 800, 774, 565; HRMS (DART) calcd for $C_{21}H_{22}NO$ [M + H]$^+$ 304.17014, found 304.16882.

(*E*)-*N*-(2-(4-methoxyphenyl)propan-2-yl)-1-(4-(methylthio)phenyl)methanimine

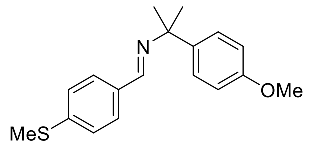

(1k); Colorless solid; Mp: 55-57 °C; ^{1}H NMR (500 MHz, $CDCl_3$) δ: 8.09 (1H, s), 7.67 (2H, d, *J* = 8.50 Hz), 7.33 (2H, d, *J* = 9.07 Hz), 7.25 (2H, m), 6.87 (2H, d, *J* = 8.50 Hz), 3.81 (3H, s), 2.50 (3H, s), 1.62 (6, s); ^{13}C NMR (150 MHz, $CDCl_3$) δ: 157.9, 156.4, 141.5, 140.2, 133.7, 128.4, 127.3, 125.8, 113.4, 62.2, 55.2, 29.9. 15.3; IR (neat, cm^{-1}) 1634, 1511, 1300, 1240, 1177, 1085, 1034, 825, 557, 494; HRMS (DART) calcd for $C_{18}H_{22}NOS$ [M + H]$^+$ 300.14221, found 300.14115.

2-3. Preparation of chiral ligands

Preparation of chiral ligand L4

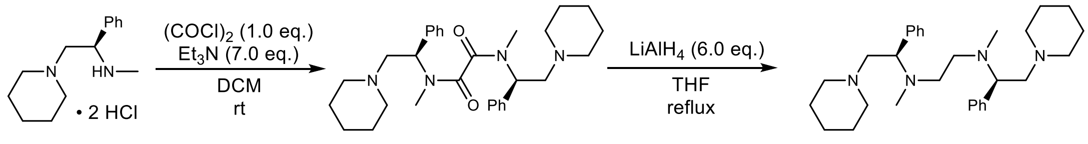

L1•2HCl (10 eq.) **S2** **L4**

Ammonium chloride salt **L1·2HCl** was prepared by acidic treatment of **L1** with 4N HCl (in ethyl acetate) and was recrystallized in EtOH. Then, Ammonium chloride salt **L1·2HCl** was converted to ligand **L4** by the following procedure. Triethylamine (4.88 mL, 35.0 mmol) was added to dispersion of **S2** (2.91 g, 10.0 mmol) in dichloromethane (DCM, 50 mL). After the mixture was cooled to 0 °C, oxalyl chloride (0.43 mL, 5.0 mmol) was added. Subsequently, the reaction mixture was stirred overnight at room temperature. After the reaction was quenched by adding saturated aq. $NaHCO_3$, the mixture was extracted with DCM (30 mL x 3). The combined organic layer was dried over anhydrous Na_2SO_4. After filtration and concentration under reduced pressure, the obtained crude product was purified by column chromatography (hexane-ethyl acetate) and recrystallization (in hexane-ethyl acetate) to afford the desired amide **S1** (0.930 mg, 38% yield). The obtained product was used without further purification in the next step.

Solution of the obtained amide **S1** (0.900 mg, 1.83 mmol) in THF (20 mL) was added to dispersion of $LiAlH_4$ (417.5 mg, 10.98 mmol) in THF (15 mL) at 0 °C. Subsequently, the reaction mixture was refluxed overnight and quenched with 3% aq. NaOH (5 mL). After filtration through a Celite pad and concentration under reduced pressure, the crude product was distilled to afford the desired tetraamine **L4** (296.5 mg, 35% yield).

***N^1*,*N^2*-dimethyl-*N^1*,*N^2*-bis((*R*)-1-phenyl-2-(piperidin-1-yl)ethyl)ethane-1,2-diamine (L4);** Colorless oil; ^{1}H NMR (600 MHz, $CDCl_3$) δ: 7.21-7.20 (4H, m), 7.15-7.13 (6H, m), 3.62 (2H, dd, *J* = 7.33, 3.67 Hz), 2.75 (2H, dd, *J* = 6.42, 3.21 Hz), 2.52 (2H, dd, *J* = 6.42, 3.21 Hz), 2.46-2.38 (4H, m), 2.28 (8H, s), 2.08 (6H, s), 1.43-1.39 (8H, m), 1.29 (4H, d, *J* = 4.81 Hz).; ^{13}C NMR (150 MHz, $CDCl_3$) δ: 140.1, 128.6, 127.7, 126.6, 65.8, 61.7, 55.1, 52.5, 39.0, 25.9, 24.3; IR (neat, cm^{-1}) 2932, 2783, 1451, 1305, 1154, 1111, 1040, 994, 867, 781, 740, 698, 591, 530; HRMS (DART) calcd for $C_{30}H_{47}N_4$ [M + H]$^+$ 463.38007, found 463.37806; $[\alpha]_D^{20}$ = –13.69 (c = 0.93, $CHCl_3$).

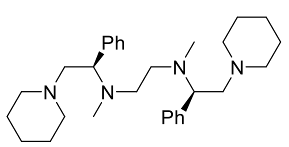

Preparation of chiral ligand L5

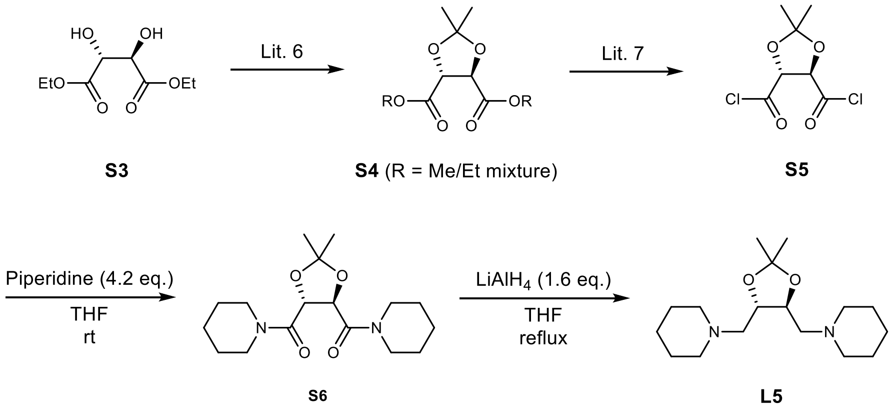

Carbonyl chloride **S5** was synthesized from tartrate **S3** according to the literatures.[6,7] Ester mixture **S4** was used instead of pure methyl ester, which was employed in the literature. Then, carbonyl chloride **S5** was converted to ligand **L5** by the following procedure.

Carbonyl chloride **S5** (2.10 g, 9.25 mmol) dissolved in THF (10 mL) was added to a solution of piperidine (4.57 mL, 46.3 mmol) in THF (8.50 mL) at 0 °C. Then, the reaction mixture was stirred overnight at room temperature, and quenched with water. The mixture was extracted with DCM (20 mL x 3). Then, the combined organic layer was washed with saturated $NaHCO_3$ and was dried over Na_2SO_4. After filtration and concentration under reduced pressure, the crude product obtained was recrystallized (in hexane) to afford the desired amide **S6** (2.22 g, 6.86 mmol, 74% yield). In the next step, hydride reduction similar to ligand **L4** was performed to afford ligand **L5** (70% yield).

1,1'-(((4*S*,5*S*)-2,2-dimethyl-1,3-dioxolane-4,5-diyl)bis(methylene))dipiperidine (L5);

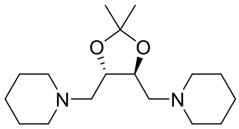

Colorless solid; Mp 37–39 °C; ^{1}H NMR (500 MHz, $CDCl_3$) δ: 3.83 (2H, dd, *J* = 12.47, 10.20 Hz), 2.51-2.45 (12H, m), 1.60-1.56 (4H, m), 1.43-1.39 (10H, m); ^{13}C NMR (125 MHz, $CDCl_3$) δ: 109.0, 77.9, 62.1, 55.3, 27.2, 25.8, 24.2; IR (neat, cm^{-1}) 2932, 2853, 1442, 1368, 1301, 1214, 1157, 1124, 1077, 1058, 995, 863, 845, 805, 780, 513; HRMS (Dart) calcd for $C_{17}H_{33}N_2O_2$ $[M + H]^+$ 297.25358, found 297.25420; $[\alpha]_D^{20}$ = –23.73 (c 1.22, $CHCl_3$).

Preparation of chiral ligands L6-L10 and L14-L22

Title ligands were synthesized from Cbz-protected amino acids according to the reported procedure for synthesis of **L1**.

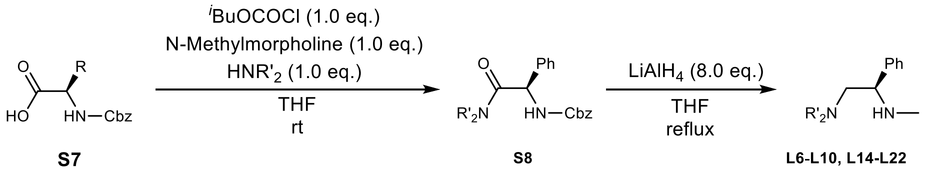

Isobutyl chloroformate (6.57 mL, 50.0 mmol) was added to a solution of *N*-Cbz-

protected amino acid **S7** (50.0 mmol) and 4-methylmorpholine (5.50 mL, 50.0 mmol) in 125 mL of THF at −15 °C with stirring, and the corresponding secondary amine (50.0 mmol) was subsequently added to the reaction mixture. The reaction mixture was stirred for 1 h at the same temperature, and then stirring was continued at room temperature overnight. The reaction mixture was concentrated and dissolved in 200 mL of ethyl acetate. The obtained mixture was washed with 1 N HCl (100 mL x 2), water (100 mL), saturated $NaHCO_3$ aqueous solution (100 mL x 2), and water (100 mL). The ethyl acetate layer was dried over anhydrous Na_2SO_4. After filtration, the corrected organic layer was concentrated under vacuumed condition to afford crude **S8**, and the obtained product **S8** was used in the next step without further purification.

The crude product obtained above was dissolved in THF (100 mL) and was added dropwise to a dispersion of $LiAlH_4$ (15.2 g, 400 mmol) in THF (150 mL) at 0 °C under argon atmosphere. Subsequently, the dispersion was refluxed overnight. The dispersion was cooled to 0 °C and quenched with 15 mL of water followed sequentially by 15 mL of 15% NaOH aq. and 10 ml of water. After filtration to remove white solid, the filtrate was concentrated to afford an oily residue. The oily residue was dissolved in 200 mL of Et_2O, and 50 mL of 4N HCl in EtOAc was added to this solution to afford an ammonium salt derived from the desired amine. The salt was collected by filtration and dried under reduced pressure. Subsequently, the obtained salt was recrystallized in EtOH to enhance the enantiopurity. The purified salt was dissolved in 100 mL water and was basified with 15% aq. NaOH. The mixture was extracted with DCM (50 mL x 3), and the combined organic layer was dried over anhydrous Na_2CO_3. After filtration and concentration, the obtained crude product was distilled under vacuumed condition to afford the desired amine. Structures of **L7-L9**, **L14-L16**, and **L18** were confirmed by comparison with data shown in the literature[28].

(*R*)-*N*-methyl-2-(4-methylpiperazin-1-yl)-1-phenylethan-1-amine (L6); Colorless

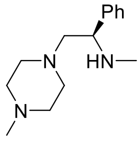

solid; Mp 31–33 °C; ^{1}H NMR (600 MHz, $CDCl_3$) δ: 7.35-7.32 (4H, m), 7.26-7.24 (1H, m), 3.62 (1H, dd, *J* = 11.00, 3.44 Hz), 2.66-2.29 (17H, m); ^{13}C NMR (150 MHz, $CDCl_3$) δ: 142.3, 128.3, 127.4, 127.2, 65.7, 62.2, 55.3, 53.3 (broaden), 46.1, 34.7; IR (neat, cm^{-1}) 2930, 2789, 1454, 1357, 1282, 1164, 1132, 1117, 1033, 1011, 910, 837, 763, 703, 608, 566; HRMS (DART) calcd for $C_{14}H_{24}N_3$ $[M + H]^+$ 234.29702, found 234.19794; $[\alpha]_D^{20}$ = –93.04 (c 1.86, $CHCl_3$).

(*S*)-*N*,4-dimethyl-1-(piperidin-1-yl)pentan-2-amine (L10); Colorless oix; ^{1}H NMR

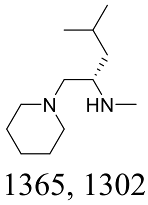

(600 MHz, $CDCl_3$) δ: 2.56-2.35 (6H, m), 2.28-2.15 (4H, m), 2.00 (1H, s), 1.69-1.61 (1H, m), 1.59-1.50 (4H, m), 1.42-1.33 (3H, m), 1.10-1.03 (1H, m), 0.93-0.87 (6H, m); ^{13}C NMR (150 MHz, $CDCl_3$) δ:63.8, 54.9, 54.2, 42.1, 34.1, 26.1, 24.9, 24.5, 23.5, 22.6; IR (neat, cm^{-1}) 2933, 2785, 1468, 1442, 1365, 1302, 1155, 1121, 1105, 1040, 992, 780, 418; HRMS (DART) calcd for $C_{12}H_{27}N_2$ $[M + H]^+$ 199.21742, found 199.21766; $[\alpha]_D^{20}$ = +96.98 (c 1.33, $CHCl_3$).

(*R*)-2-(*meso*-3,5-dimethylpiperidin-1-yl)-*N*-methyl-1-phenylethan-1-amine (L17);

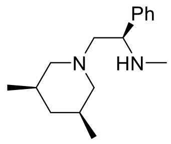

Colorless oil; ^{1}H NMR (500 MHz, $CDCl_3$) δ: 7.35-7.32 (4H, m), 7.25-7.23 (1H, m), 3.63 (1H, dd, J = 11.34, 3.40 Hz), 2.97 (1H, dt, J = 10.77, 1.70 Hz), 2.71 (1H, dt, J = 10.58, 1.56 Hz), 2.47 (1H, dd, *J* = 12.47, 10.77 Hz), 2.34-2.30 (4H, m), 2.24 (1H, dd, *J* = 12.75, 3.12 Hz), 1.76-1.58 (4H, m), 1.37 (1H, t, *J* = 11.05 Hz), 0.88 (3H, d, *J* = 6.24 Hz), 0.82 (3H, d, *J* = 6.24 Hz), 0.51 (1H, q, *J* = 12.28 Hz); ^{13}C NMR (125 MHz, $CDCl_3$) δ: 142.8, 128.3, 127.3, 127.0, 66.2, 63.7, 62.5, 60.2, 42.3, 34.7, 31.3, 31.2, 19.7, 19.5; IR (neat, cm^{-1}) 2949, 2785, 1454, 1354, 1312, 1191, 1137, 1080, 1023, 881, 755, 700, 635, 576, 501; HRMS (DART) calcd for $C_{16}H_{27}N_2$ $[M + H]^+$ 247.21742, found 247.21673; $[\alpha]_D^{20}$ = –83.14 (c 1.33, $CHCl_3$).

(*R*)-2-(4-ethylpiperazin-1-yl)-*N*-methyl-1-phenylethan-1-amine (L19); Colorless

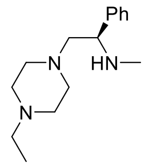

solid; Mp 51–55 ºC; ^{1}H NMR (600 MHz, $CDCl_3$) δ: 7.34-7.32 (4H, m), 7.26-7.23 (1H, m), 3.62 (1H, dd, *J* = 11.00, 3.44 Hz), 2.67-2.26 (16H, m), 1.09 (3H, t, *J* = 7.22 Hz); ^{13}C NMR (125 MHz, $CDCl_3$) δ: 142.4, 128.3, 127.4, 127.1, 65.8, 62.1, 53.3 (broaden), 52.9, 52.3, 34.7, 12.0; IR (neat, cm^{-1}) 2942, 2809, 1448, 1350, 1290, 1162, 1121, 1011, 837, 757, 701, 640, 606, 563, 514; HRMS (DART) calcd for $C_{15}H_{26}N_3$ $[M + H]^+$ 248.21267, found 248.21301; $[\alpha]_D^{20}$ = –73.42 (c 2.19, $CHCl_3$).

(*R*)-2-(4-cyclohexyl-1-yl)-*N*-methyl-1-phenylethan-1-amine (L20); Colorless solid;

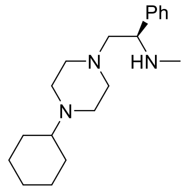

Mp 56–60 ºC; ^{1}H NMR (600 MHz, $CDCl_3$) δ: 7.34-7.32 (4H, m), 7.26-7.21 (2H, m), 3.61 (1H, dd, *J* = 11.00, 2.75 Hz), 2.62-2.17 (14H, m), 1.90-1.62 (6H, m), 1.24-1.20 (4H, m), 1.14-1.03 (1H, m); ^{13}C NMR (150 MHz, $CDCl_3$) δ: 142.5, 128.3, 127.4, 127.1, 65.9, 63.5, 62.2, 49.1, 34.7, 29.1, 29.0, 26.3, 25.9; IR (neat, cm^{-1}) 2926, 2853, 2809, 1452, 1345, 1292, 1157, 1122, 1052, 1008, 979, 754, 701; HRMS (DART) calcd for $C_{19}H_{32}N_3$ $[M + H]^+$ 302.25962, found 302.25934; $[\alpha]_D^{20}$ = –54.82 (c 1.05, $CHCl_3$).

(*R*)-2-(4-(*tert*-butyl)piperazin-1-yl)-*N*-methyl-1-phenylethan-1-amine (L21);

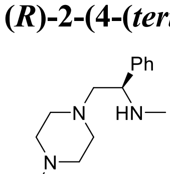

Colorless solid; Mp 58–64 ºC; $[\alpha]_D^{20}$ = –66.02 (c 1.20, $CHCl_3$); ^{1}H NMR (600 MHz, $CDCl_3$) δ: 7.34-7.32 (4H, m), 7.26-7.24 (1H, m), 3.61 (1H, dd, *J* = 11.00, 3.44 Hz), 2.65-2.26 (14H, m), 1.09 (9H, s).; ^{13}C NMR (150 MHz, $CDCl_3$) δ: 142.5, 128.3, 127.4, 127.1, 65.8, 62.2, 53.9 (broaden), 45.8, 34.7, 25.8; IR (neat, cm^{-1}) 2966, 2938, 2803, 1438, 1357, 1297, 1207, 1128, 1010, 967, 907, 838, 753, 728, 700, 601, 494; HRMS (DART) calcd for $C_{17}H_{30}N_3$ $[M + H]^+$ 276.24397, found 276.24364.

(*R*)-*N*-methyl-2-(4-methylpiperidin-1-yl)-1-phenylethan-1-amine (L22); Colorless oil; ^{1}H NMR (500 MHz, $CDCl_3$) δ: 7.36-7.30 (4H, m), 7.25-7.23 (1H, m), 3.61 (1H, dd, J = 10.77, 3.40 Hz), 3.03 (1H, d, *J* = 11.34 Hz), 2.74 (1H, d, *J* = 11.34 Hz), 2.47 (1H, dd, *J* = 12.47, 11.34 Hz), 2.29-2.25 (5H, m), 2.10 (1H, td, *J* = 11.34, 2.46 Hz), 1.85 (1H, td, *J* = 11.48, 2.65 Hz), 1.66-1.56 (2H, m), 1.36-1.17 (3H, m), 0.92 (3H, d, *J* = 6.24 Hz); ^{13}C NMR (125 MHz, $CDCl_3$) δ: 142.8, 128.3, 127.4, 127.0, 66.3, 62.5, 55.9, 52.5, 34.8, 34.6, 34.5, 30.9, 21.9; IR (neat, cm^{-1}) 2922, 2788, 1442, 1351, 1288, 1142, 1114, 1083, 1025, 977, 911, 837, 754, 700, 637, 603; HRMS (DART) calcd for $C_{15}H_{25}N_2$ $[M + H]^+$ 233.20177, found 233.20201; $[\alpha]_D^{20}$ = –90.42 (c 1.12, $CHCl_3$).

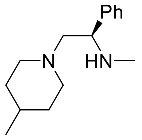

Preparation of chiral ligands L11-L13

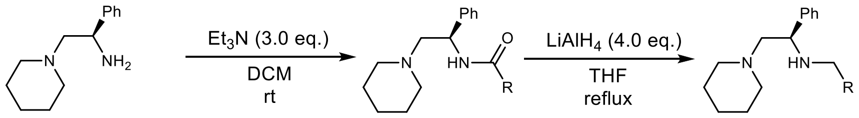

S9 **S10** **L11-L13**

Diamine **S9** was synthesized according to the literature[4] and was converted to the desired ligands **L11-L13**. To a mixture of diamine **S9** (5.0 mmol), triethylamine (25 mmol), and DCM (25 mL), the corresponding alkanoyl chloride (6.0 mmol) was added. Then, the reaction mixture was stirred overnight at room temperature and was quenched with saturated $NaHCO_3$. Subsequently, the mixture was extracted with DCM (20 mL x 3), and the combined organic layer was dried over Na_2SO_4. After filtration and concentration under reduced pressure, the crude product **S10** was obtained and used without further purification in the next step.

The crude product **S10** dissolved in THF (5.0 mL) was added to dispersion of $LiAlH_4$ (20 mmol) in THF (5.0 mL) at 0 °C. Then, the reaction mixture was refluxed overnight and was quenched with saturated Na_2SO_4. After filtration through a Celite pad, the crude product obtained was distilled to afford the desired amine. Structure of **L13** was confirmed by comparison with data shown in the literature[29].

(*R*)-*N*-ethyl-1-phenyl-2-(piperidin-1-yl)ethan-1-amine (L11); The title compound is known as its racemic form. The structure was confirmed by comparison with data shown in the literature.[8] $[\alpha]_D^{20}$ = –97.62 (c 1.07, $CHCl_3$).

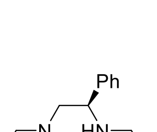

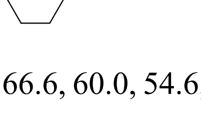

(*R*)-*N*-propyl-1-phenyl-2-(piperidin-1-yl)ethan-1-amine (L12); Colorless oil; ^{1}H NMR (500 MHz, $CDCl_3$) δ: 7.37 (2H, d, *J* = 7.37 Hz), 7.31 (2H, t, *J* = 7.37 Hz), 7.24 (1H, q, *J* = 6.99 Hz), 3.75 (1H, dd, *J* = 11.34, 3.40 Hz), 2.55-2.24 (9H, m), 1.65-1.43 (8H, m), 0.89 (3H, t, *J* = 7.37 Hz).; ^{13}C NMR (125 MHz, $CDCl_3$) δ: 143.4, 128.2, 127.3, 126.8, 66.6, 60.0, 54.6, 49.8, 26.2, 24.5, 23.1, 11.8; IR (neat, cm^{-1}) 2933, 2799, 1452, 1357, 1301,

1154, 1110, 994, 865, 753, 700, 627, 583, 528; HRMS (DART) calcd for $C_{16}H_{27}N_2$ [M + H]$^+$ 247.21742, found 247.21691; $[\alpha]_D^{20}$ = –94.91 (c 1.13, $CHCl_3$).

2-4. Optimization

General procedure of initial investigations (Supplementary Table 1)

A mixed base (KO^{t}Bu 5.6 mg, 5.0 x 10^{-2} mmol; LiTMP 7.4 mg, 5.0 x 10^{-2} mmol) or KCH_2SiMe_3 (6.3 mg, 5.0 x 10^{-2} mmol) was placed in a flame-dried 10 mL flask inside a glove box fulfilled with argon. The flask was cooled to –78 °C, then the corresponding ligand (5.5 x 10^{-2} mmol) dissolved in the employed solvent (0.40 mL) was added. When TBME was employed as a solvent, toluene (63.8 µL, 0.60 mmol) was also introduced into the flask. After the mixture was stirred for 30 min at the same temperature, *p*-methoxycumylimine **1a** (126.7 mg, 0.50 mmol) dissolved in the solvent (0.60 mL) was successively introduced into the flask via a well-dried cannula, and the whole mixture was stirred for 18 h at the same temperature. The reaction was quenched by adding a few drops of MeOH, and the obtained mixture was extracted with DCM (10 mL x 3). The combined organic layer was dried over anhydrous Na_2SO_4. After filtration and concentration under reduced pressure, the obtained crude product was purified by PTLC (hexane-ethyl acetate) to afford the desired amine **3aa**. The enantioselectivity was determined by HPLC

General procedure of catalyst preparation conditions (Supplementary Table 2)

A mixed base (KO^{t}Bu 5.6 mg, 5.0 x 10^{-2} mmol; LiTMP 7.4 mg, 5.0 x 10^{-2} mmol) or KCH_2SiMe_3 (6.3 mg, 5.0 x 10^{-2} mmol) was placed in a flame-dried 10 mL flask inside a glove box fulfilled with argon. The flask was cooled to the temperature shown in Supplementary Table 2, then the corresponding amine **L1** (12.0 mg, 5.0 x 10^{-2} mmol) in the solvent (0.40 mL) was added. When TBME was employed as a solvent, toluene (63.8 µL, 0.60 mmol) was also introduced into the flask. After the mixture was stirred under the conditions shown in Supplementary Table 2, the flask was cooled to –78 °C, and then *p*-methoxycumylimine **1a** (126.7 mg, 0.50 mmol) dissolved in the solvent (0.60 mL) was successively introduced into the flask via a well-dried cannula, and the whole mixture was stirred for 18 h. The reaction was quenched by adding a few drops of MeOH, and the obtained mixture was extracted with DCM (10 mL x 3). The combined organic layer was dried over anhydrous Na_2SO_4. After filtration and concentration under reduced pressure, the obtained crude product was purified by PTLC (hexane-ethyl acetate) to afford the desired amine **3aa**. The enantioselectivity was determined by HPLC

General procedure for ligand screening (Table 1, Supplementary Table 3)

A mixed base (KO^{t}Bu 5.6 mg, 5.0 x 10^{-2} mmol; LiTMP 7.4 mg, 5.0 x 10^{-2} mmol) or KCH_2SiMe_3 (6.3 mg, 5.0 x 10^{-2} mmol) was placed in a flame-dried 10 mL flask inside a glove box fulfilled with argon. After the flask was cooled to –40 °C, the corresponding amine ligand **L** (5.5 × 10^{-2} mmol) in toluene (0.40 mL) was added, and the mixture was stirred at the same temperature for 30 minutes. Subsequently, the flask was cooled to –78 °C, and imine **1a** (126.7 mg, 0.500 mmol) dissolved in toluene (0.60 mL) was

successively introduced into the flask via a well-dried cannula. After the whole mixture was stirred for 18 h at the same temperature, the reaction was quenched by adding a few drops of MeOH, and the obtained mixture was extracted with DCM (10 mL x 3), then combined organic layer was dried over anhydrous Na_2SO_4. After filtration and concentration under reduced pressure, the obtained crude product was purified by PTLC (hexane-ethyl acetate) to afford the desired amine **3aa**. The enantioselectivities were determined by HPLC.

General procedure for investigation of additives (Table 2)

KCH_2SiMe_3 (6.3 mg, 5.0 x 10^{-2} mmol) and the employed additive (5.0 x 10^{-2} mmol) were placed in a flame-dried 10 mL flask inside a glove box fulfilled with argon. After the flask was cooled to the corresponding temperature, the corresponding amine ligand **L6** (12.8 mg, 5.5 x 10^{-2} mmol) in toluene (0.40 mL) was added, and the mixture was stirred at –40 °C for 30 minutes. Subsequently, the flask was cooled to –78 °C, and imine **1a** (126.7 mg, 0.500 mmol) dissolved in toluene (0.60 mL) was successively introduced into the flask via a well-dried cannula. After the whole mixture was stirred for 18 h at the same temperature, the reaction was quenched by adding a few drops of MeOH, and the obtained mixture was extracted with DCM (10 mL x 3), then combined organic layer was dried over anhydrous Na_2SO_4. After filtration and concentration under reduced pressure, the obtained crude product was purified by PTLC (hexane-ethyl acetate) to afford the desired amine **3aa**. The enantioselectivities were determined by HPLC.

2-5. Substrate scope

Experimental procedure of the catalytic asymmetric addition reaction (Figure 1)

KCH_2SiMe_3 (9.6 mg, 7.5 x 10^{-2} mmol) and KHMDS (15.0 mg, 7.5 x 10^{-2} mmol) were placed in a flame-dried 10 mL flask inside a glove box fulfilled with argon. The flask was cooled to –40 ºC, then diamine ligand **L6** (19.4 mg, 8.3 x 10^{-2} mmol) in toluene (0.80 mL) was added, and the chiral base mixture was stirred for 30 min at the same temperature. After the flask was cooled at –78 ºC, *p*-methoxycumylimine **1a** (253.4 mg, 1.000 mmol) dissolved in toluene (1.20 mL) was successively introduced via a well-dried cannula, and the whole mixture was stirred for 18 h at the same temperature. After the reaction was quenched by adding water, the mixture was extracted with DCM (10 mL x 3). The combined organic layer was dried over anhydrous Na_2SO_4. After filtration and concentration under reduced pressure, the crude product obtained was purified by PTLC (hexane-ethyl acetate) to afford the desired adduct **3**. As for **3aa**, **3ea**, **3fa**, and **3ga**, the enantiopurities could be enhanced by recrystallization. After a tiny amount of the obtained products was used for characterization, the remaining parts were employed for the recrystallization. The recovery yields were calculated based on the remaining parts.

In order to determine the absolute configuration of **3aa**, it was derivatized to the corresponding amide via removal of PMC group and typical amidation as shown below.[9] Those of the other adducts were determined as analogies.

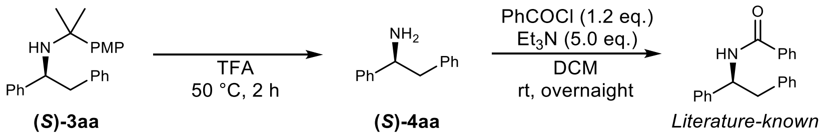

Experimental procedure of the catalytic asymmetric addition reaction in cumene solvent (Figure 1, for 3ad, 3ae, 3af)

KCH_2SiMe_3 (12.3 mg, 0.100 mmol) and KHMDS (20.0 mg, 0.100 mmol) were placed in a flame-dried 10 mL flask inside a glove box fulfilled with argon. The flask was cooled to –40 ºC, and cumene (0.20 mL) was added. Then alkylarene (2.5 mmol) was introduced into the flask, and diamine ligand **L6** (25.7 mg, 0.110 mmol) in cumene (0.20 mL) was added, and the chiral base mixture was stirred for 30 min at the same temperature. After the flask was cooled at –60 ºC, *p*-methoxycumylimine **1a** (126.7 mg, 0.5000 mmol) dissolved in cumene (0.60 mL) was successively introduced via a well-dried cannula, and the whole mixture was stirred for 18 h at the same temperature. After the reaction was quenched by adding water, the mixture was extracted with DCM (10 mL x 3). The combined organic layer was dried over anhydrous Na_2SO_4. After filtration and concentration under reduced pressure, the crude product obtained was purified by PTLC (hexane-ethyl acetate) to afford the desired adduct **3**.

(*S*)-*N*-(1,2-diphenylethyl)-2-(4-methoxyphenyl)propan-2-amine (3aa); The structure was confirmed by comparison with data of ^{1}H and ^{13}C NMR shown in literature which our group reported.[5] The product obtained in the gram-scale reaction was used for the recrystallization to enhance the enantiopurity (the result is shown in its procedure). ^{1}H NMR (500 MHz, $CDCl_3$) δ: 7.25-7.15 (8H, m), 6.98 (2H, dd, *J* = 7.37, 1.70 Hz), 6.94 (2H, td, *J* = 5.95, 3.78 Hz), 6.69 (2H, td, *J* = 6.09, 3.59 Hz), 3.79 (3H, s), 3.55 (1H, dd, *J* = 9.15, 5.15 Hz), 2.78 (1H, dd, *J* = 13.60, 5.10 Hz), 2.67 (1H, dd, *J* = 13.60, 9.07 Hz), 1.75 (1H, s), 1.21 (3H, s), 1.07 (3H, s); ^{13}C NMR (125 MHz, $CDCl_3$) δ: 157.7, 147.3, 140.1, 138.9, 129.5, 128.3, 128.0, 127.1, 126.9, 126.4, 126.3, 113.1, 59.8, 56.0, 55.2, 46.9, 32.4, 27.9; HPLC analysis using Shinwa Chemical Industries Ltd. ULTRON ES-PhCD column (20 mM KH_2PO_4:MeCN = 75:25, 1.0 mL/min, 210 nm, tR = 42.4 min (*R*), 47.6 min (*S*)); $[\alpha]^{20}_{D}$ = −47.12 (99% ee, c 0.63, $CHCl_3$).

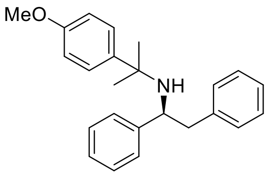

(*S*)-*N*-(1-(4-(*tert*-butyl)phenyl)-2-phenylethyl)-2-(4-methoxyphenyl)propan-2-amine (3ba); The structure was confirmed by comparison with data of ^{1}H and ^{13}C NMR shown in literature which our group reported.[5] ^{1}H NMR (500 MHz, $CDCl_3$) δ: 7.24-7.22 (5H, m), 7.16 (2H, d, *J* = 7.94 Hz), 7.01 (2H, dd, *J* = 7.65, 1.42 Hz), 6.91 (2H, d, *J* = 8.50 Hz), 6.67 (2H, d, *J* = 8.50 Hz), 3.79 (3H, s), 3.53 (1H, dd, *J* = 4.72, 2.36 Hz), 2.79 (1H, dd, *J* = 13.60, 5.10 Hz), 2.65 (1H, dd, *J* = 13.60, 9.07 Hz), 1.74 (1H, s), 1.31 (9H, s), 1.20 (3H, s), 1.06 (3H, s); ^{13}C NMR (150 MHz, $CDCl_3$) δ: 157.6, 149.2, 144.1, 140.1, 139.2, 129.5, 128.2, 127.0, 126.7, 126.2, 124.8, 113.0, 59.4, 55.9, 55.1, 46.8, 34.4, 32.2, 31.5, 28.1; HPLC analysis using Shinwa Chemical Industries Ltd. ULTRON ES-OVM column (20 mM KH_2PO_4:MeCN = 85:15, 1.0 mL/min, 210 nm, tR = 5.8 min (*R*), 7.7 min (*S*)); $[\alpha]^{20}_{D}$ = −45.86 (89% ee, c 1.47, $CHCl_3$).

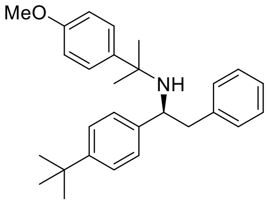

(*S*)-*N*-(1-(4-isopropylphenyl)-2-phenylethyl)-2-(4-methoxyphenyl)propan-2-amine (3ca); The structure was confirmed by comparison with data of ^{1}H and ^{13}C NMR shown in literature which our group reported.[5] ^{1}H NMR (500 MHz, $CDCl_3$) δ: 7.23-7.21 (3H, m), 7.16 (2H, d, *J* = 8.50 Hz), 7.09 (2H, d, *J* = 7.94 Hz), 7.00 (2H, d, *J* = 6.24 Hz), 6.91 (2H, d, J = 9.07 Hz,), 6.67 (2H, d, J = 8.50 Hz), 3.79 (3H, s), 3.53 (1H, dd, *J* = 4.72, 2.36 Hz), 2.91-2.83 (1H, m), 2.78 (1H, dd, *J* = 13.32, 4.82 Hz), 2.65 (1H, dd, *J* = 13.32, 9.35 Hz), 1.58 (1H, s), 1.23-1.21 (9H, m), 1.06 (3H, s); ^{13}C NMR (125 MHz, $CDCl_3$) δ: 157.6, 146.9, 144.5, 140.1, 139.2, 129.5, 128.3, 127.0, 126.9, 126.2, 126.0, 113.0, 59.5, 56.0, 55.2, 46.8, 33.7, 32.3, 28.0, 24.08, 24.06; The ee value was evaluated after derivatization to **4ca**.

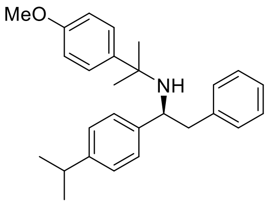

(*S*)-*N*-(1-(4-ethylphenyl)-2-phenylethyl)-2-(4-methoxyphenyl)propan-2-amine

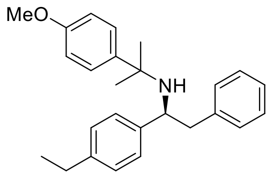

(3da); Colorless solid; Mp: 65-67 °C; ^{1}H NMR (500 MHz, $CDCl_3$) δ: 7.24-20 (3H, m), 7.16 (2H, d, *J* = 7.94 Hz), 7.07 (2H, td, *J* = 7.94 Hz), 6.99 (2H, d, *J* = 6.24 Hz), 6.92 (2H, d, J = 8.50 Hz), 6.68 (2H, d, *J* = 9.07 Hz), 3.79 (3H, s), 3.53 (1H, dd, *J* = 4.72, 2.36 Hz), 2.78 (1H, dd, *J* = 13.32, 4.82 Hz), 2.65-2.62 (3H, m), 1.73 (1H, s), 1.23-1.22 (6H, m), 1.07 (3H, s); ^{13}C NMR (150 MHz, $CDCl_3$) δ: 157.6, 144.5, 142.3, 140.2, 130.1, 129.5, 128.3, 127.5, 127.5, 127.00, 126.95, 126.2, 113.0, 59.5, 56.0, 55.2, 46.9, 32.4, 28.4, 28.0, 15.5; IR (neat, cm^{-1}) 2970, 1608, 1579, 1508, 1457, 1300, 1251, 1180, 1031, 825, 737, 697, 548; HRMS (DART) calcd for $C_{26}H_{32}NO$ $[M + H]^+$ 374.24839, found 374.24985; The ee value was evaluated after derivatization to **4da**.

(*S*)-*N*-(1-([1,1'-biphenyl]-4-yl)-2-phenylethyl)-2-(4-methoxyphenyl)propan-2-amine

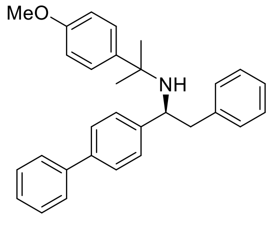

(3ea); The enantiopurity was enhanced by recrystallization (99% ee, 59% recovery). Colorless solid, Mp: 78-81 °C; ^{1}H NMR (500 MHz, $CDCl_3$) δ: 7.60 (2H, d, *J* = 8.50 Hz), 7.49 (2H, d, *J* = 7.94 Hz), 7.43 (2H, t, *J* = 7.65 Hz), 7.32-7.22 (6H, m), 7.02 (2H, d, *J* = 7.94 Hz), 6.95 (2H, d, *J* = 8.50 Hz), 6.69 (2H, d, *J* = 8.50 Hz), 3.79 (3H, s), 3.61 (1H, dd, *J* = 4.72, 2.36 Hz), 2.83 (1H, dd, *J* = 13.32, 5.38 Hz), 2.70 (1H, dd, *J* = 13.60, 9.07 Hz), 1.80 (1H, s), 1.24 (3H, s), 1.11 (3H, s); ^{13}C NMR (125 MHz, $CDCl_3$) δ: 157.7, 146.5, 141.1, 140.0, 139.2, 138.9, 129.5, 128.7, 128.3, 128.0, 127.8, 127.6, 127.5, 126.9, 126.7, 126.3, 113.1, 59.5, 56.1, 55.2, 46.8, 32.3, 28.1; IR (neat, cm^{-1}) 3022, 1602, 1508, 1482, 1105, 1248, 1180, 1028, 825, 763, 725, 691, 614, 586, 554, 526, 491; HRMS (DART) calcd for $C_{30}H_{32}NO$ $[M + H]^+$ 422.24839, found 422.24712; HPLC analysis using Shinwa Chemical Industries Ltd. ULTRON ES-OVM column (20 mM KH_2PO_4:MeCN = 85:15, 1.0 mL/min, 210 nm, tR = 16.0 min (*S*), 70.0 min (*R*)); $[\alpha]_D^{20}$ = −71.73 (99% ee, c 0.46, $CHCl_3$).

(*S*)-2-(4-methoxyphenyl)-*N*-(1-(naphthalen-1-yl)-2-phenylethyl)propan-2-amine

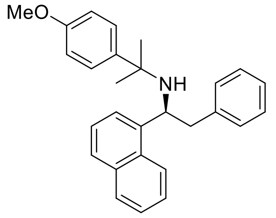

(3fa); The enantiopurity was enhanced by recrystallization (>99% ee, 71% recovery). Colorless solid, Mp: 104-108 °C; ^{1}H NMR (500 MHz, $CDCl_3$) δ: 7.95-7.89 (2H, m, broaden), 7.72 (1H, d, *J* = 7.94 Hz), 7.46-7.43 (3H, m, broaden), 7.27-7.21 (4H, overlapped with $CHCl_3$), 7.05 (2H, d, *J* = 5.67 Hz), 6.91 (2H, d, *J* = 9.07 Hz), 6.65 (2H, d, *J* = 8.50 Hz), 4.47 (1H, s, broaden), 3.79 (3H, s), 2.97 (1H, d, *J* = 11.34), 2.65 (1H, s, broaden), 1.90 (1H, s), 1.22 (3H, s), 0.99 (3H, s); ^{13}C NMR (150 MHz, $CDCl_3$) δ: 157.6, 143.3, 140.1, 139.1, 133.9, 130.5, 129.4, 128.9, 128.4, 126.9, 126.7, 126.3, 125.54, 125.48, 125.0, 124.9, 122.0, 113.1, 56.1, 55.2, 53.4, 45.8, 32.1, 27.4; IR (neat, cm^{-1}) 1608, 1508, 1462, 1374, 1302, 1251, 1180, 1034, 831, 803, 785, 743, 697, 622, 517, 500, 431; HRMS (DART) calcd for $C_{28}H_{30}NO$ $[M + H]^+$ 396.23274, found 396.23452; HPLC analysis using Shinwa Chemical Industries Ltd.

ULTRON ES-OVM column (20 mM KH_2PO_4:MeCN = 80:20, 1.0 mL/min, 210 nm, tR = 4.6 min (*R*), 5.7 min (*S*)); $[\alpha]_D^{20}$ = −37.61 (>99% ee, c 0.60, $CHCl_3$).

(*S*)-2-(4-methoxyphenyl)-*N*-(1-(naphthalen-2-yl)-2-phenylethyl)propan-2-amine

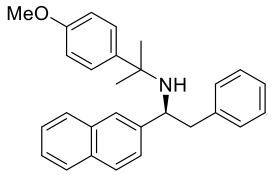

(3ga); The structure was confirmed by comparison with data of ^{1}H and ^{13}C NMR shown in literature which our group reported.[5] The enantiopurity was enhanced by recrystallization (99% ee, 24% recovery). ^{1}H NMR (500 MHz, $CDCl_3$) δ: 7.80-7.75 (3H, m), 7.59 (1H, s), 7.52 (1H, d, *J* = 8.50 Hz), 7.43-7.42 (2H, m), 7.29-7.20 (3H, m), 7.02 (2H, d, *J* = 7.37 Hz), 6.93 (2H, d, *J* = 9.07 Hz), 6.68 (2H, d, *J* = 8.50 Hz), 3.77 (3H, s), 3.73 (1H, dd, *J* = 8.79, 5.38 Hz), 2.86 (1H, dd, *J* = 13.60, 5.10 Hz), 2.75 (1H, dd, *J* = 13.60, 9.07 Hz), 1.87 (1H, s), 1.23 (3H, s), 1.07 (3H, s); ^{13}C NMR (125 MHz, $CDCl_3$) δ: 157.7, 144.9, 140.0, 138.9, 133.4, 132.6, 129.5, 128.3, 127.67, 127.65, 127.58, 126.9, 126.3, 125.8, 125.6, 125.5, 125.2, 113.1, 59.9, 56.1, 55.2, 46.7, 32.3, 28.0; HPLC analysis using Shinwa Chemical Industries Ltd. ULTRON ES-OVM column (20 mM KH_2PO_4:MeCN = 85:15, 1.0 mL/min, 210 nm, tR = 51.4 min (*S*), 47.1 min (*R*)); $[\alpha]_D^{20}$ = −81.11 (99% ee, c 0.51, $CHCl_3$).

(*S*)-2-(4-methoxyphenyl)-*N*-(1-(4-methoxyphenyl)-2-phenylethyl)propan-2-amine

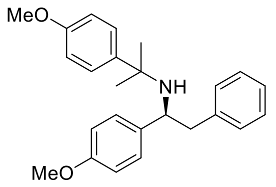

(3ha); The structure was confirmed by comparison with data of ^{1}H and ^{13}C NMR shown in literature which our group reported.[5] ^{1}H NMR (500 MHz, $CDCl_3$) δ: 7.24-7.16 (3H, m), 7.14 (2H, d, *J* = 8.25 Hz), 6.97 (2H, d, *J* = 6.19 Hz), 6.93 (2H, d, *J* = 8.94 Hz), 6.78 (2H, d, *J* = 8.25 Hz), 6.70 (2H, d, *J* = 8.94 Hz), 3.79 (6H, m), 3.50 (1H, dd, *J* = 8.25, 5.50 Hz), 2.76 (1H, dd, *J* = 6.19, 3.09 Hz), 2.66 (1H, dd, *J* = 13.75, 8.94 Hz), 1.56 (1H, s), 1.21 (3H, s), 1.07 (3H, s); ^{13}C NMR (150 MHz, $CDCl_3$) δ: 158.2, 157.7, 140.1, 139.3, 139.1, 129.5, 128.2, 128.1, 126.9, 126.2, 113.4, 113.1, 59.1, 56.0, 55.21, 55.19, 46.9, 32.4, 28.0; The ee value was evaluated after derivatization to **4ha**.

(*S*)-2-(4-methoxyphenyl)-*N*-(1-(3-methoxyphenyl)-2-phenylethyl)propan-2-amine

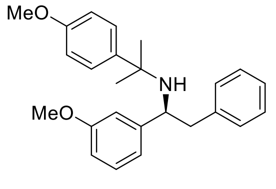

(3ia); The structure was confirmed by comparison with data of ^{1}H and ^{13}C NMR shown in literature which our group reported.[5] ^{1}H NMR (500 MHz, $CDCl_3$) δ: 7.24-7.22 (3H, m), 6.99 (2H, dd, *J* = 7.37, 1.70 Hz), 6.93 (2H, d, *J* = 9.07 Hz), 6.84-6.82 (2H, m), 6.72-6.69 (3H, m), 3.79 (6H, m), 3.53 (1H, dd, *J* = 4.72, 2.36 Hz), 2.79 (1H, dd, *J* = 13.60, 5.10 Hz), 2.66 (1H, dd, *J* = 11.34, 5.67 Hz), 1.62 (1H, s), 1.21 (3H, s), 1.09 (3H, s); ^{13}C NMR (150 MHz, $CDCl_3$) δ: 159.4, 157.7, 149.2, 140.0, 138.9, 129.5, 128.9, 128.3, 126.9, 126.3, 119.6, 113.1, 112.6, 111.9, 59.8, 56.0, 55.2, 46.8, 32.3, 27.9; HPLC analysis using Shinwa Chemical Industries Ltd. ULTRON ES-OVM column (20 mM KH_2PO_4:MeCN = 80:20, 1.0 mL/min, 210 nm, tR = 2.6 min (*R*), 2.9 min (*S*)); $[\alpha]_D^{20}$ = −34.12 (73% ee, c 1.33, $CHCl_3$).

(*S*)-2-(4-methoxyphenyl)-*N*-(1-(2-methoxyphenyl)-2-phenylethyl)propan-2-amine

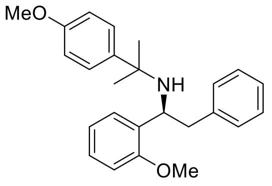

(3ja); The structure was confirmed by comparison with data of ^{1}H and ^{13}C NMR shown in literature which our group reported.[5] ^{1}H NMR (500 MHz, $CDCl_3$) δ: 7.38 (1H, s), 7.20-7.12 (4H, m), 7.09 (2H, d, *J* = 9.07 Hz), 6.98 (2H, d, *J* = 6.80 Hz), 6.86 (1H, t, *J* = 7.37 Hz), 6.77 (1H, d, *J* = 7.94 Hz), 6.68 (2H, d, *J* = 9.07 Hz), 4.06 (1H, s), 3.77 (3H, s), 2.86 (1H, dd, *J* = 13.32, 5.38 Hz), 2.61 (1H, dd, *J* = 10.20, 5.10 Hz), 1.75 (1H, s), 1.18 (3H, s), 1.09 (3H, s); ^{13}C NMR (150 MHz, $CDCl_3$) δ: 157.6, 156.3, 140.8, 139.8, 135.5, 129.5, 128.5 (broaden), 128.0, 126.98, 126.95, 125.9, 120.3, 112.9, 110.3, 55.9, 55.24, 55.18, 45.0, 31.7, 27.9; HPLC analysis using Shinwa Chemical Industries Ltd. ULTRON ES-OVM column (20 mM KH_2PO_4:MeCN = 80:20, 1.0 mL/min, 210 nm, tR = 2.9 min (*S*), 4.7 min (*R*)); $[\alpha]_D^{20}$ = −24.8 (84% ee, c 1.71, $CHCl_3$).

(*S*)-2-(4-methoxyphenyl)-*N*-(1-(4-(methylthio)phenyl)-2-phenylethyl)propan-2-

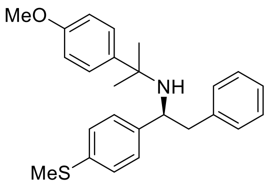

amine (3ka); Slightly yellow solid, Mp = 86-88 °C; ^{1}H NMR (500 MHz, $CDCl_3$) δ: 7.24-7.19 (3H, m), 7.17-7.14 (4H, m), 6,97 (2H, dd, *J* = 7.37, 1.70 Hz), 6.92 (2H, d, *J* = 8.50 Hz), 6.69 (2H, d, *J* = 9.07 Hz), 3.79 (3H, s), 3.52 (1H, dd, *J* = 8.79, 5.38 Hz), 2.75 (1H, dd, *J* = 13.60, 5.10 Hz), 2.65 (1H, dd, *J* = 13.60, 9.07 Hz), 2.47 (3H, s), 1.75 (1H, s), 1.21 (3H, s), 1.07 (3H, s); ^{13}C NMR (150 MHz, $CDCl_3$) δ: 157.7, 144.5, 140.0, 138.8, 135.8, 129.4, 128.3, 127.7, 126.9, 126.5, 126.3, 113.1, 59.3, 56.0, 55.2, 46.8, 32.3, 28.1, 16.1; IR (neat, cm^{-1}) 2976, 1605, 1508, 1494, 1434, 1297, 1248, 1180, 1094, 1028, 825, 700, 646, 543; HRMS (DART) calcd for $C_{25}H_{30}NOS$ $[M + H]^+$ 392.20481, found 392.20409; The ee value was evaluated after derivatization to **4ka**.

(*S*)-4-(1-((2-(4-methoxyphenyl)propan-2-yl)amino)-2-phenylethyl)-*N*,*N*-

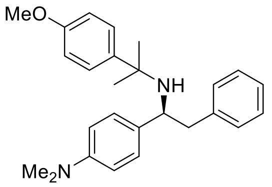

dimethylaniline (3la); The structure was confirmed by comparison with data of ^{1}H and ^{13}C NMR shown in literature which our group reported.[5] ^{1}H NMR (600 MHz, $CDCl_3$) δ: 7.23-7.20 (3H, m), 7.10 (2H, d, *J* = 8.94 Hz), 6.99 (2H, d, *J* = 6.19 Hz), 6.93 (2H, d, *J* = 8.94 Hz), 6.70 (2H, d, *J* = 8.94 Hz), 6.65 (2H, d, *J* = 8.94 Hz), 3.80 (3H, s), 3.46 (1H, d, *J* = 4.81 Hz), 2.92 (6H, s), 2.78 (1H, dd, *J* = 13.75, 5.50 Hz), 2.67 (1H, dd, *J* = 13.06, 8.94 Hz), 1.73 (1H, s), 1.22 (3H, s), 1.09 (3H, s); ^{13}C NMR (150 MHz, $CDCl_3$) δ: 157.6, 149.4, 140.2, 139.4, 129.5, 128.2, 127.8, 127.0, 126.1, 113.0, 112.4, 59.1, 56.0, 55.2, 46.9, 40.8, 32.5, 27.9; HPLC analysis using Shinwa Chemical Industries Ltd. ULTRON ES-OVM column (20 mM KH_2PO_4:MeCN = 85:15, 1.0 mL/min, 210 nm, tR = 3.1 min (*R*), 4.9 min (*S*)); $[\alpha]_D^{20}$ = −36.33 (99% ee, c 0.51, $CHCl_3$).

(*S*)-*N*-(2-(4-ethylphenyl)-1-phenylethyl)-2-(4-methoxyphenyl)propan-2-amine

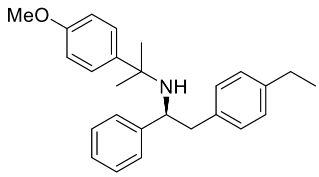

(3ab); The structure was confirmed by comparison with data of ^{1}H and ^{13}C NMR shown in literature which our group reported.[5] ^{1}H NMR (600 MHz, $CDCl_3$) δ: 7.26-7.23 (4H, m), 7.18-7.16 (1H, m), 7.07 (2H, d, *J* = 7.56 Hz), 6.92-6.90 (4H, m), 6.67 (2H, d, *J* = 8.25 Hz), 3.79 (3H, s), 3.54 (1H, dd, *J* = 4.58, 2.29 Hz), 2.75 (1H, dd, *J* = 13.40, 5.15 Hz), 2.64-2.62 (3H, m), 1.71 (1H, s), 1.24 (3H, t, *J* = 7.56 Hz), 1.20 (3H, s), 1.06 (3H, s); ^{13}C NMR (150 MHz, $CDCl_3$) δ: 157.6, 147.5, 142.2, 140.1, 136.0, 129.4, 128.0, 127.8, 127.1, 126.9, 126.3, 113.0, 59.8, 56.0, 55.2, 46.4, 32.5, 28.5, 27.9, 15.8; The ee value was evaluated after derivatization to **4ab**.

(*1SR*,*2RS*)-*N*-(2-(4-methoxyphenyl)propan-2-yl)-1,2-diphenylpropan-1-amine

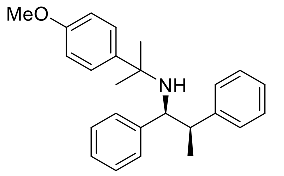

(3ac); The structure was confirmed by comparison with data of ^{1}H and ^{13}C NMR shown in literature which our group reported.[5] ^{1}H NMR (500 MHz, $CDCl_3$) δ: 7.26-7.11 (8H, m), 7.05 (2H, d, *J* = 6.87 Hz), 6.72 (2H, d, *J* = 8.94 Hz), 6.63 (2H, d, *J* = 8.25 Hz), 3.74 (3H, s), 3.21 (1H, d, *J* = 8.94 Hz), 2.64 (1H, dt, *J* = 16.50, 6.87 Hz), 1.64 (1H, s), 1.00 (3H, s), 0.85 (3H, s), 0.73 (3H, d, *J* = 6.87 Hz); ^{13}C NMR ($CDCl_3$, 150 MHz) δ: 157.6, 145.8, 144.7, 139.7, 128.4, 128.3, 128.1, 127.8, 127.1, 126.6, 126.5, 112.9, 64.5, 55.7, 55.2, 47.8, 32.4, 27.6, 19.3; HPLC analysis using Shinwa Chemical Industries Ltd. ULTRON ES-PhCD column (20 mM KH_2PO_4:MeCN = 60:40, 2.0 mL/min, 210 nm, tR = 12.4 min (*1R,2S*), 31.3 min *(1R,3S)*).

(*S*)-2-(4-methoxyphenyl)-*N*-(1-phenyl-2-(*p*-tolyl)ethyl)propan-2-amine (3ad); The

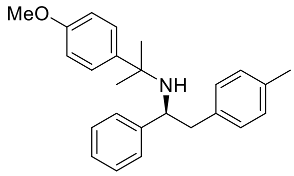

structure was confirmed by comparison with data of ^{1}H and ^{13}C NMR shown in literature which our group reported.[5] ^{1}H NMR (600 MHz, $CDCl_3$) δ: 7.25-7.21 (4H, m), 7.17-7.15 (1H, m), 7.02 (2H, d, *J* = 7.56 Hz), 6.92 (2H, d, *J* = 8.25 Hz), 6.86 (2H, d, *J* = 7.56 Hz), 6.67 (2H, d, *J* = 8.94 Hz), 3.78 (3H, s), 3.52 (1H, dd, *J* = 4.81, 2.41 Hz), 2.74 (1H, dd, *J* = 13.40, 5.15 Hz), 2.61 (1H, dd, *J* = 13.75, 8.94 Hz), 2.32 (3H, s), 1.75 (1H, s), 1.19 (3H, s), 1.05 (3H, s); ^{13}C NMR ($CDCl_3$, 150 MHz) δ: 157.6, 147.4, 140.1, 135.72, 135.67, 129.3, 128.9, 128.0, 127.1, 126.9, 126.3, 113.0, 59.7, 56.0, 55.1, 46.4, 32.4, 27.9, 21.0; HPLC analysis using Shinwa Chemical Industries Ltd. ULTRON ES-PhCD column (20 mM KH_2PO_4:MeCN = 75:25, 1.0 mL/min, 210 nm, tR = 48.1 min (*R*), 68.7 min *(S)*); $[\alpha]_D^{20}$ = −10.57 (68% ee, c 1.01, $CHCl_3$).

(*S*)-2-(4-methoxyphenyl)-*N*-(1-phenyl-2-(*m*-tolyl)ethyl)propan-2-amine (3ae); The structure was confirmed by comparison with data of ^{1}H and ^{13}C NMR shown in literature which our group reported.[5] ^{1}H NMR (500 MHz, $CDCl_3$) δ: 7.21-7.13 (4H, m), 7.11-7.04 (2H, m), 6.95 (1H, d, *J* = 7.37 Hz), 6.80 (2H, d, *J* = 8.50 Hz), 6.74 (1H, d, *J* = 7.37 Hz), 6.71 (1H, s), 6.59 (2H, d, *J* = 9.07 Hz), 3.70 (3H, s), 3.45 (1H, dd, *J* = 4.72, 2.36 Hz), 2.67 (1H, dd, *J* = 13.60, 4.53 Hz), 2.52 (1H, dd, *J* = 13.32, 9.35 Hz), 2.21 (3H, s), 1.71 (1H, s), 1.12 (3H, s), 0.97 (3H, s); ^{13}C NMR ($CDCl_3$, 125 MHz) δ: 157.6, 147.5, 140.0 138.7, 137.8, 130.2, 128.1, 128.0, 127.1, 127.0, 126.9, 126.5, 126.3, 113.0, 59.7, 56.0, 55.1, 46.8, 32.5, 27.8, 21.3; The ee value was evaluated after derivatization to **4ae**.

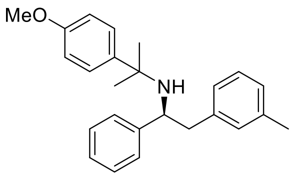

(*S*)-2-(4-methoxyphenyl)-*N*-(1-phenyl-2-(*o*-tolyl)ethyl)propan-2-amine (3af); The structure was confirmed by comparison with data of ^{1}H and ^{13}C NMR shown in literature which our group reported.[5] ^{1}H NMR (600 MHz, $CDCl_3$) δ: 7.18-7.14 (4H, m), 7.09 (1H, t, *J* = 6.87 Hz), 7.05 (1H, t, *J* = 7.22 Hz), 6.99 (2H, t, *J* = 6.53 Hz), 6.85 (1H, d, *J* = 6.87 Hz), 6.78 (2H, d, *J* = 8.94 Hz), 6.59 (2H, t, *J* = 5.84 Hz), 3.70 (3H, s), 3.44 (1H, dd, *J* = 4.81, 2.41 Hz), 2.70 (1H, dd, *J* = 13.40, 5.15 Hz), 2.59 (1H, dd, *J* = 13.75, 9.62 Hz), 1.95 (3H, s), 1.75 (1H, s), 1.13 (3H, s), 0.96 (3H, s); ^{13}C NMR ($CDCl_3$, 150 MHz) δ:157.63, 147.7, 140.0, 137.0, 136.8, 130.5, 130.4, 128.0, 126.9, 126.8, 126.5, 126.3, 125.6, 113.1; HPLC analysis using Shinwa Chemical Industries Ltd. ULTRON ES-OVM column (20 mM KH_2PO_4:MeCN = 85:15, 1.0 mL/min, 210 nm, tR = 4.4 min (*S*), 6.3 min *(R)*); $[\alpha]_D^{20}$ = −57.45 (75% ee, c 1.22, $CHCl_3$).

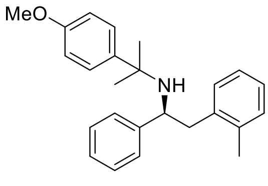

(*S*)-*N*-(2-(4-isopropylphenyl)-1-phenylethyl)-2-(4-methoxyphenyl)propan-2-amine (3ah); The structure was confirmed by comparison with data of ^{1}H and ^{13}C NMR shown in literature which our group reported.[5] ^{1}H NMR (600 MHz, $CDCl_3$) δ: 7.27-7.23 (4H, m), 7.17 (1H, t, *J* = 7.22 Hz), 7.11 (2H, d, *J* = 8.25 Hz), 6.94 (2H, d, *J* = 8.25 Hz), 6.86 (2H, d, *J* = 8.25 Hz), 6.66 (2H, d, *J* = 8.25 Hz), 3.78 (3H, s), 3.52 (1H, dd, *J* = 4.81, 2.41 Hz), 2.90 (1H, dd, *J* = 27.49, 13.75 Hz), 2.77-2.74 (1H, m), 2.62 (1H, dd, *J* = 13.40, 9.28 Hz), 1.81 (1H, s), 1.26 (6H, dd, *J* = 3.44, 1.72 Hz), 1.19 (3H, s), 1.05 (3H, s); ^{13}C NMR ($CDCl_3$, 150 MHz) δ: 157.6, 147.4, 140.1, 135.72, 135.67, 129.3, 128.9, 128.0, 127.1, 126.9, 126.3, 59.7, 56.0, 55.1, 46.4, 32.4, 27.9, 21.0; HPLC analysis using Shinwa Chemical Industries Ltd. ULTRON ES-PhCD column (20 mM KH_2PO_4:MeCN = 70:30, 1.0 mL/min, 210 nm, tR = 53.8 min (*R*), 94.3 min *(S)*); $[\alpha]_D^{20}$ = +2.39 (18% ee, c 1.39, $CHCl_3$).

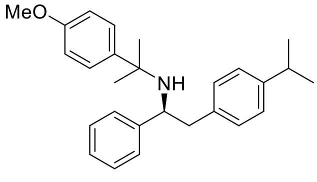

2-6. Synthetic utility

2-6-1. Gram-scale reaction (Figure 2-a)

KCH_2TMS (94.7 mg, 0.750 mmol) and KHMDS (149.6 mg, 0.750 mmol) were placed in a flame-dried 10 mL flask inside a glove box fulfilled with argon. The flask was cooled to –40 ºC, and toluene (3.0 mL) was added followed by diamine ligand **L6** (193.7 mg, 0.830 mmol) dissolved in toluene (3.0 mL) was added. Subsequently, the mixture was stirred for 30 min at the same temperature. After the flask was cooled at –78 ºC, toluene (4.0 mL) was added to the reaction mixture. Then, *p*-methoxycumylimine **1a** (2.53 g, 0.500 mmol) dissolved in toluene (10.0 mL) was successively introduced via a well-dried cannula, and the whole mixture was stirred for 18 h at the same temperature. After the reaction was quenched by adding MeOH, the mixture was extracted with DCM (10 mL x 3). The combined organic layer was dried over anhydrous Na_2SO_4. After filtration and concentration under reduced pressure, the crude product obtained was purified by column chromatography (hexane-ethyl acetate) to afford the desired amine **3aa** (3.09 g, 89% yield). The enantioselectivity was determined by HPLC (86% ee). The obtained product (3.09 g) was recrystallized in hexane-chloroform to enhance its enantiopurity (99% ee, 2.14 g, 69% recovery).

2-6-2. *In situ* preparation of imine (Figure 2-b)

p-Methoxycumylamine **S1** (337.1 mg, 2.04 mmol), aldehyde (212.2 mg, 2.00 mmol), and pellet-type MS 4 Å (1.00 g) were placed in a flame-dried 10 mL flask inside a glove box fulfilled with argon. Toluene (1.0 mL) was introduced to the flask, and the reaction mixture was gently stirred for 18 h at room temperature with preventing the pellets of MS 4 Å from being broken. The obtained solution was directly used in the next step.

KCH_2TMS (18.9 mg, 0.150 mmol) and KHMDS (29.9 mg, 0.150 mmol) were placed in another flame-dried 10 mL flask inside a glove box fulfilled with argon. The flask was cooled to –40 ºC, then diamine ligand **L6** (38.7 mg, 0.166 mmol) dissolved in toluene (1.20 mL) was added, and the chiral base mixture was stirred for 30 min at the same temperature. After the flask was cooled at –78 ºC, toluene (0.80 mL) was added, and the imine solution prepared above was successively introduced via a well-dried cannula. The flask for imine preparation was rinsed with toluene (1.0 mL), and the solution was also added in the reaction mixture. The whole mixture was stirred for 18 h at the same temperature. After the reaction was quenched by adding MeOH, the mixture was extracted with DCM (10 mL x 3). The combined organic layer was dried over anhydrous Na_2SO_4. After filtration and concentration under reduced pressure, the crude product obtained was purified by PTLC (hexane-ethyl acetate) to afford the desired amine **3aa** (559.7 mg, 81% yield). The enantioselectivity was determined by HPLC (84% ee).

2-6-3. Removal of *p*-methoxy group (Figure 2-c)

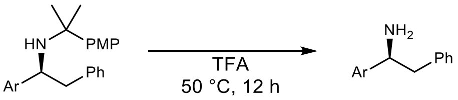

(*S*)-**3** (*S*)-**4**

p-Methoxycumylamine **3** was placed in a 10 mL flask, and TFA (2.0 mL per mmol of **3**) was subsequently added to the flask. The mixture was heated to 50 ºC and stirred for 12 h at the same temperature. Subsequently, water (1.0 mL per mmol of **3**) was added to the flask, and the mixture was stirred for 15 min at the same temperature. After cooling to room temperature, the reaction was basified with 15% NaOH aq. and extracted with DCM (20 mL x 3). The combined organic layer was dried over anhydrous Na_2SO_4. After filtration and evaporation, the obtained crude was diluted with Et_2O, and was extracted with 1N HCl aq. (20 mL x 2). The combined aqueous layer was washed with Et_2O (10 mL x 2) and then was basified with 15% NaOH aq.. The basified aqueous layer was extracted with DCM (20 mL x 3). The combined organic layer was dried over anhydrous Na_2SO_4, and concentrated under reduced vacuum after filtration to afford the desired almost pure primary amine **4**. If the purity was not enough, the product was further purified by PTLC (Et_2O-Hexane-Et_2NH).

As for **4ba**, **4ca**, **4ia**, and **4ja**, the enantiopurities could be enhanced by recrystallization (in CPME-Acetone-EtOH) of the corresponding ammonium chloride salt formed with 1N HCl in Et_2O. After a tiny amount of the obtained products was used for characterization, the remaining parts were employed for the recrystallization. The recovery yields were calculated based on the remaining parts.

(*S*)-*N*-(1,2-diphenylethyl)-2-(4-methoxyphenyl)propan-2-amine (4aa); **(*S*)-3aa** (345.5 mg, 1.00 mmol, 99% ee) was used, and **4aa** (179.2 mg, 91% yield, 99% ee) was obtained. The structure was confirmed by comparison with data of ^1H and ^{13}C NMR shown in the literature which our group reported.[5]

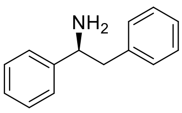

^1H NMR (600 MHz, $CDCl_3$) δ: 7.36-7.17 (10H, m), 4.19 (1H, dd, *J* = 4.58, 2.29 Hz), 3.01 (1H, dd, *J* = 13.06, 4.81 Hz), 2.82 (1H, dd, *J* = 13.75, 8.94 Hz), 1.50 (2H, s); ^{13}C NMR (150 MHz, $CDCl_3$) δ: 145.6, 139.1, 129.3, 128.4, 127.0, 126.4, 124.3, 57.5, 46.5; HPLC analysis using Daicel Chiralcel OD-H column (Hexane:^{i}PrOH:Et_2NH = 90:10:0.1, 1.0 mL/min, 254 nm, tR = 7.6 min (*R*), 11.4 min (*S*)); $[\alpha]_D^{20}$ = −0.27 (99% ee, c 1.36, $CHCl_3$).

(*S*)-1-(4-(*tert*-butyl)phenyl)-2-phenylethan-1-amine (4ba); **3ba** (324.3 mg, 0.808 mmol, 89% ee) was used, and **4ba** (132.8 mg, 65% yield, 89% ee) was obtained. The structure was confirmed by comparison with data of ^1H and ^{13}C NMR shown in the literature.[10] The enantiopurity was enhanced by recrystallization of the corresponding ammonium chloride salt (>99% ee, 31% recovery). ^1H NMR (600 MHz, $CDCl_3$) δ: 7.30-7.28 (2H, m), 7.25-7.21 (4H, m), 7.16-7.13 (3H, m), 4.10 (1H, dd, *J* = 4.58, 2.29 Hz), 2.96 (1H, dd,

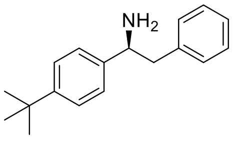

J = 13.40, 4.47 Hz), 2.73 (1H, dd, J = 13.75, 9.62 Hz), 1.63 (2H, s), 1.25 (9H, s); ^{13}C NMR (150 MHz, $CDCl_3$) δ: 150.0, 142.5, 139.3, 129.3, 128.4, 126.3, 126.1, 125.3, 57.1, 46.3, 34.5, 31.4; HPLC analysis using Daicel Chiralcel OD-H column (Hexane:^{i}PrOH:Et_2NH = 90:10:0.1, 1.0 mL/min, 254 nm, tR = 5.9 min (*R*), 6.7 min (*S*)), using Daicel Chiralcel OD-RH column (for **4ba·HCl**, 20 mM $NaB(OH)_4$/ MeCN = 60/40, 0.5 mL/min, 210 nm, tR = 79.3 min (*R*), 82.7 min (*S*)); $[\alpha]^{20}_{D}$ = +105.62 (as **4ba·HCl**, >99% ee, c 0.14, EtOH).

(*S*)-1-(4-isopropylphenyl)-2-phenylethan-1-amine (4ca); **3ca** (277.2 mg, 0.715 mmol) was used, and **4ca** (135.5 mg, 79% yield, 89% ee) was obtained. The structure was confirmed by comparison with data of ^{1}H and ^{13}C NMR shown in the literature.[10] The enantiopurity was enhanced by recrystallization of the corresponding ammonium chloride salt (>99% ee, 15% recovery). ^{1}H NMR (600 MHz, $CDCl_3$) δ: 7.30-7.28 (4H, m), 7.23-7.19 (5H, m), 4.17 (1H, dd, J = 4.58, 2.29 Hz), 3.02 (1H, dd, J = 13.40, 4.47 Hz), 2.94-2.87 (1H, m), 2.80 (1H, dd, J = 13.40, 9.28 Hz), 1.61 (s, 3H), 1.25 (6H, d, J = 6.87 Hz); ^{13}C NMR (150 MHz, $CDCl_3$) δ: 147.7, 142.8, 139.2, 129.3, 128.4, 126.4, 126.3, 57.2, 46.3, 33.7, 24.0; HPLC analysis using Daicel Chiralcel OD-H column (Hexane:^{i}PrOH:Et_2NH = 90:10:0.1, 1.0 mL/min, 254 nm, tR = 5.8 min (*R*), 6.5 min (*S*)), using Daicel Chiralcel OD-RH column (for **4ca·HCl**, 20 mM $NaB(OH)_4$/ MeCN = 60/40, 0.5 mL/min, 210 nm, tR = 57.9 min (*R*), 60.9 min (*S*)); $[\alpha]^{20}_{D}$ = +103.25 (as **4ca·HCl**, >99% ee, c 0.10, EtOH).

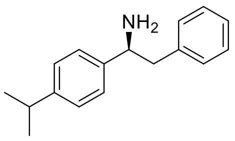

(*S*)-1-(4-ethylphenyl)-2-phenylethan-1-amine (4da); **3da** (134.5 mg, 0.360 mmol) was used, and **4da** (72.8 mg, 90% yield, 81% ee) was obtained. Colorless oil; ^{1}H NMR (600 MHz, $CDCl_3$) δ: 7.32-7.15 (9H, m), 4.20 (1H, dd, J = 8.25, 5.50 Hz), 3.04 (1H, dd, J = 13.75, 5.50 Hz), 2.91 (1H, dd, J = 13.40, 7.90 Hz), 2.62 (2H, q, J = 7.56 Hz), 1.22 (3H, t, J = 7.56 Hz); ^{13}C NMR (150 MHz, $CDCl_3$) δ:143.2, 142.4, 139.1, 129.3, 128.4, 127.9, 126.4, 126.36, 57.2, 46.2, 28.5, 15.5; IR (neat, cm^{-1})2965, 1602, 1511, 1494, 1454, 1077, 1031, 1020, 828, 700, 543; HRMS (Dart) calcd for $C_{16}H_{20}N$ $[M + H]^+$ 226.15957, found 226.15966; HPLC analysis using Daicel Chiralcel OD-H column (Hexane:^{i}PrOH:Et_2NH = 90:10:0.1, 1.0 mL/min, 254 nm, tR = 6.4 min (*R*), 7.5 min (*S*)); $[\alpha]^{20}_{D}$ = +16.38 (84% ee, c 0.24, $CHCl_3$).

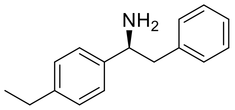

(*S*)-1-(4-methoxyphenyl)-2-phenylethan-1-amine (4ha); **3ha** (256.7 mg, 0.684 mmol) was used, and **4ca** (134.2 mg, 86% yield, 90% ee) was obtained. The structure was confirmed by comparison with data of ^{1}H and ^{13}C NMR shown in the literature.[10] ^{1}H NMR (600 MHz, $CDCl_3$) δ: 7.29-7.26 (4H, m), 7.21 (1H, t, J = 7.56 Hz), 7.16 (2H, d, J = 7.56 Hz), 6.86 (2H, d, J = 8.25 Hz), 4.15 (1H, dd, J = 4.58, 2.29 Hz), 3.80 (3H, s), 2.98 (1H, dd, J = 13.06, 4.81 Hz), 2.81 (1H, dd, J = 13.40, 8.59 Hz), 1.63 (2H, s); ^{13}C NMR (125 MHz, $CDCl_3$) δ: 158.6 139.1, 137.6, 129.3, 128.4, 127.5, 126.3, 113.7, 56.9, 55.3, 46.5; HPLC analysis using

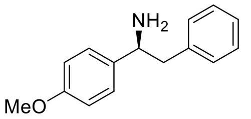

Daicel Chiralcel OD-H column (Hexane:^{i}PrOH:Et_2NH = 90:10:0.1, 1.0 mL/min, 254 nm, tR = 9.2 min (*R*), 11.7 min (*S*)); $[\alpha]_D^{20}$ = +29.02 (90% ee, c 0.10, $CHCl_3$).

(*S*)-1-(3-methoxyphenyl)-2-phenylethan-1-amine (4ia); **3ia** (256.7 mg, 0.684 mmol, 76% ee) was used, and **4ia** (76.8 mg, quant., 76% ee) was obtained. The enantiopurity was enhanced by recrystallization of the corresponding ammonium chloride salt (97% ee, 11% recovery). Colorless oil; ^{1}H NMR (600 MHz, $CDCl_3$) δ: 7.30-7.17 (6H, m), 6.94-6.93 (2H, m), 6.80 (1H, dd, *J* = 8.25, 2.06 Hz), 4.18 (1H, dd, *J* = 4.58, 2.29 Hz), 3.79 (3H, s), 3.02 (1H, dd, *J* = 13.40, 5.15 Hz), 2.84 (1H, dd, *J* = 13.40, 8.59 Hz), 1.86 (2H, s); ^{13}C NMR (125 MHz, $CDCl_3$) δ: 159.7, 146.9, 138.9, 129.4, 129.3, 128.4, 126.4, 118.8, 112.7, 111.9, 57.5, 55.2, 46.2; IR (neat, cm^{-1}) 3002, 1599, 1585, 1 485, 1454, 1434, 1254, 1074, 1043, 871, 780, 697, 511, 468; HRMS (Dart) calcd for $C_{15}H_{18}NO$ $[M + H]^+$ 228.13884, found 228.13782; HPLC analysis using Daicel Chiralcel OD-H column (Hexane:^{i}PrOH:Et_2NH = 90:10:0.1, 1.0 mL/min, 254 nm, tR = 10.2 min (*R*), 13.0 min (*S*)); $[\alpha]_D^{20}$ = +110.66 (as **4ia·HCl**, 97% ee, c 0.10, EtOH).

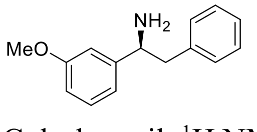

(*S*)-1-(2-methoxyphenyl)-2-phenylethan-1-amine (4ja); **3ja** (335.3 mg, 0.893 mmol, 84% ee) was used, and **4ja** (163.2 mg, 80% yield, 84% ee) was obtained. The structure was confirmed by comparison with data of ^{1}H and ^{13}C NMR shown in the literature.[10] The enantiopurity was enhanced by recrystallization of the corresponding ammonium chloride salt (>99% ee, 37% recovery). ^{1}H NMR (600 MHz, $CDCl_3$) δ: 7.26 (1H, dd, *J* = 7.56, 1.37 Hz), 7.22-7.12 (6H, m), 6.86 (1H, t, *J* = 7.90 Hz), 6.81 (1H, d, *J* = 8.25 Hz), 4.40 (1H, dd, *J* = 4.35, 2.18 Hz), 3.77 (3H, s), 3.05 (1H, dd, *J* = 13.75, 4.81 Hz), 2.71 (1H, dd, *J* = 13.06, 8.94 Hz), 1.68 (2H, s); ^{13}C NMR (150 MHz, $CDCl_3$) δ: 156.7, 139.8, 133.5, 129.4, 128.3, 127.8, 126.7, 126.1, 120.6, 110.4, 55.3, 52.2, 44.2; HPLC analysis using Daicel Chiralcel OD-H column (Hexane:^{i}PrOH:Et_2NH = 90:10:0.1, 1.0 mL/min, 254 nm, tR = 7.7 min (*R*), 8.1 min (*S*)); $[\alpha]_D^{20}$ = +90.76 (as **4ja·HCl**, >99% ee, c 0.11, EtOH).

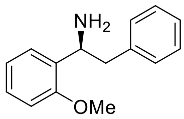

(*S*)-1-(4-(methylthio)phenyl)-2-phenylethan-1-aminee (4ka); **3ka** (308.9 mg, 0.789 mmol) was used, and **4ka** (117.9 mg, 61% yield, 80% ee) was obtained. The structure was confirmed by comparison with data of ^{1}H and ^{13}C NMR shown in the literature.[10] ^{1}H NMR (500 MHz, $CDCl_3$) δ: 7.27-7.25 (5H, m), 7.21-7.19 (3H, m), 7.14 (2H, d, *J* = 6.80 Hz), 4.14 (1H, dd, *J* = 4.53, 2.27 Hz), 2.96 (1H, dd, *J* = 13.32, 4.82 Hz), 2.78 (1H, dd, *J* = 13.32, 8.79 Hz), 2.46 (3H, s), 1.52 (2H, s); ^{13}C NMR (150 MHz, $CDCl_3$) δ: 142.7, 138.9, 136.8, 129.3, 128.4, 127.0, 126.8, 126.4, 57.1, 46.4, 16.1; HPLC analysis using Daicel Chiralcel OD-H column (Hexane:^{i}PrOH:Et_2NH = 90:10:0.1, 1.0 mL/min, 254 nm, tR = 10.7 min (*R*), 11.8 min (*S*)); $[\alpha]_D^{20}$ = +106.44 (as **4ka·HCl**, 84% ee, c 0.12, EtOH).

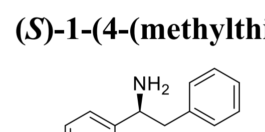

(*S*)-2-(4-ethylphenyl)-1-phenylethan-1-amine (4ab); **3ab** (167.8 mg, 0.449 mmol) was used, and **4ab** (63.4 mg, 65% yield, 62% ee) was obtained. Colorless oil; ^{1}H NMR (500 MHz, $CDCl_3$) δ: 7.38-7.07 (9H, m), 4.19 (1H, dd, *J* = 4.53, 2.27 Hz), 3.01 (1H, dd, *J* = 13.60, 5.10 Hz), 2.86 (1H, dd, *J* = 13.32, 8.79 Hz), 2.64-2.42 (4H, m), 1.24-1.19 (3H, m); ^{13}C NMR (125 MHz, $CDCl_3$) δ: 145.6, 142.2, 136.2, 129.2, 128.4, 127.9, 127.0, 126.4, 57.5, 46.0, 28.4, 15.6; IR (neat, cm^{-1}) 3025, 2963, 2929, 1602, 1514, 1492, 1452, 813, 760, 698, 554, 537; HRMS (Dart) calcd for $C_{15}H_{18}NO$ $[M + H]^+$ 226.15957, found 226.15912; HPLC analysis using Daicel Chiralcel OD-H column (Hexane:^{i}PrOH:Et_2NH = 90:10:0.1, 1.0 mL/min, 254 nm, tR = 6.6 min (*R*), 11.1 min (*S*)); $[\alpha]_D^{20}$ = +9.92 (62% ee, c 0.94, $CHCl_3$).

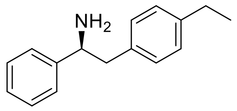

(*S*)-1-phenyl-2-(*m*-tolyl)ethan-1-amine (4ae); **3ae** (167.6 mg, 0.459 mmol) was used, and **4ae** (65.7 mg, 64% yield, 71% ee) was obtained. The structure was confirmed by comparison with data of ^{1}H and ^{13}C NMR shown in the literature.[10] ^{1}H NMR (500 MHz, $CDCl_3$) δ: 7.38-7.31 (4H, m), 7.27-7.24 (2H, m), 7.17 (1H, t, *J* = 7.37 Hz), 7.04-6.95 (3H, m), 4.20 (1H, dd, *J* = 4.53, 2.27 Hz), 3.00 (1H, dd, *J* = 13.04, 5.10 Hz), 2.84 (1H, dd, *J* = 6.52, 3.26 Hz), 2.33-2.31 (5H, m); ^{13}C NMR (125 MHz, $CDCl_3$) δ: 145.6, 139.0, 138.0, 130.1, 128.4, 128.3, 127.1, 127.0, 126.4, 126.3, 57.5, 46.4, 21.4; HPLC analysis using Daicel Chiralcel OD-H column (Hexane:^{i}PrOH:Et_2NH = 90:10:0.1, 1.0 mL/min, 254 nm, tR = 6.7 min (*R*), 12.2 min (*S*)); $[\alpha]_D^{20}$ = +16.50 (71% ee, c 0.61, $CHCl_3$).

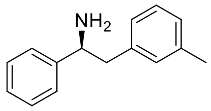

2-6-4 *N*-alkylation (Figure 2-c)

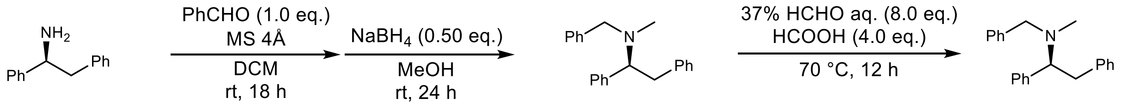

(*S*)-**4** (99% ee) **5** **6** (99% ee)

p-Methoxycumylamine **(*S*)-3aa** (197.3 mg, 1.00 mmol), benzaldehyde (106.1 mg, 1.00 mmol), MS 4 Å (1 g) were placed in a 10 mL flask, and DCM was added to the flask. The mixture was stirred for 18 h at room temperature. After MS 4 Å was removed by filtration through a Celite pad, the filtrate was concentrated and dried to afford the corresponding imine. The obtained imine was placed in 30 mL flask and was dissolved in 5.0 mL of MeOH. Subsequently, $NaBH_4$ was added to the flask, and the reaction mixture was stirred for 24h at room temperature. The mixture was concentrated under vacuum condition, and the obtained solid was dissolved in DCM and water. The two layers solution was extracted with DCM (20 mL x 3). The combined organic layer was dried over anhydrous Na_2SO_4. After filtration and concentration under reduced pressure, the crude product obtained was purified by PTLC (hexane-ethyl acetate) to afford the desired amine (239.9 mg, 83% yield).

Furthermore, amine **5** was derivatized to tertiary amine **6**. Amine **5** (71.9 mg, 0.25 mmol) was placed in a 8 mL tube, and 37% HCHO aq. (162 μL, 2.0 mmol) and formic

acid (38 μL, 4.0 mmol) added to the flask. The mixture was stirred for 12 h at 70 °C. The reaction was quenched with 15% NaOH aq., and the mixture was extracted with DCM (10 mL x 3). The combined organic layer was dried over anhydrous Na_2SO_4. After filtration and concentration under reduced pressure, the crude product obtained was purified by PTLC (hexane-ethyl acetate) to afford the desired amine **6** (67.6 mg, 90% yield).

(*S*)-*N*-Benzyl-1,2-diphenylethan-1-amine (5); The structure was confirmed by comparison with data of ^{1}H and ^{13}C NMR shown in the literature.[11] ^{1}H NMR (500 MHz, $CDCl_3$) δ: 7.35-7.33 (4H, m), 7.28-7.23 (5H, m), 7.21-7.19 (2H, m), 7.10 (4H, d, *J* = 7.37 Hz), 3.88 (1H, dd, *J* = 8.50, 5.67 Hz), 3.65 (1H, d, *J* = 13.60 Hz), 3.46 (1H, d, *J* = 13.60 Hz), 2.92 (2H, m), 1.60 (s, 1H); ^{13}C NMR (150 MHz, $CDCl_3$) δ: 143.7, 140.4, 138.8, 129.2, 128.35, 128.33, 128.2, 127.9, 127.4, 127.1, 126.7, 126.3, 63.6, 51.3, 45.3; $[\alpha]_D^{20}$ = −31.82 (c 0.55, $CHCl_3$).

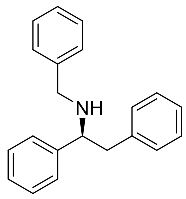

(*S*)-*N*-benzyl-*N*-methyl-1,2-diphenylethan-1-amine (6); The structure was confirmed by comparison with data of ^{1}H and ^{13}C NMR shown in the literature.[12] ^{1}H NMR (600 MHz, $CDCl_3$) δ: 7.27-7.21 (12H, m), 7.13 (1H, dd, *J* = 8.59, 5.84 Hz), 7.06 (2H, d, *J* = 6.87 Hz), 3.82 (1H, dd, *J* = 7.56, 3.78 Hz), 3.63 (1H, d, *J* = 13.75 Hz), 3.37 (1H, dd, *J* = 6.87, 3.44 Hz), 3.30 (1H, d, *J* = 13.06 Hz), 3.02 (1H, dd, *J* = 13.75, 8.25 Hz), 2.19 (3H, s); ^{13}C NMR (150 MHz, $CDCl_3$) δ: 139.9, 139.8, 139.4, 129.4, 128.8, 128.7, 128.1, 127.9, 127.8, 127.0, 126.7, 125.7, 69.5, 58.8, 38.9, 38.0; HPLC analysis using Shinwa Chemical Industries Ltd. ULTRON ES-PhCD column (20 mM KH_2PO_4:MeCN = 75:25, 1.0 mL/min, 210 nm, tR = 74.3 min (*R*), 96.9 min (*S*)); $[\alpha]_D^{20}$ = +22.50 (c 0.51, $CHCl_3$).

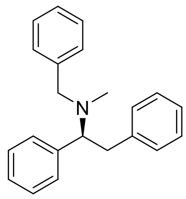

2-6-5. Transformation to 1,2,3,4-tetrahydroisoquinoline (Figure 2-d)

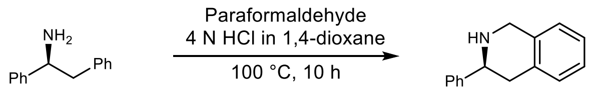

(*S*)-**4** (99% ee) **7** (99% ee)

p-Methoxycumylamine (*S*)-**3aa** (98.6 mg, 0.500 mmol) and paraformaldehyde (18.0 mg, 0.600 mmol as HCHO) were placed in a 10 mL flask, and 4 N HCl in dioxane was subsequently added. The reaction mixture was stirred for 10 h at 100 ℃. After cooling, the mixture was basified with 15% NaOH aq., and was extracted with DCM (20 mL x 3). The combined organic layer was dried over anhydrous Na_2SO_4. After filtration and concentration under reduced pressure, the crude product obtained was purified by PTLC (hexane-ethyl acetate) to afford the desired tetrahydroisoquinoline (36.1 mg, 36% yield).

(*S*)-3-phenyl-1,2,3,4-tetrahydroisoquinoline (7); The structure was confirmed by

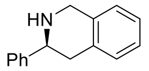

comparison with data of ^{1}H and ^{13}C NMR shown in the literature.[13] ^{1}H NMR (600 MHz, $CDCl_3$) δ: 7.44 (2H, d, J = 7.56 Hz), 7.37 (2H, m), 7.29 (1H, t, *J* = 7.56 Hz), 7.16-7.15 (m, 2H), 7.10-7.08 (m, 2H), 4.27 (1H, d, *J* = 15.81 Hz), 4.17 (1H, d, *J* = 15.81 Hz), 4.02 (dd, 1H, *J* = 8.25, 6.87 Hz), 2.99 (2H, d, *J* = 7.56 Hz), 2.04 (s, 1H); ^{13}C NMR (125 MHz, $CDCl_3$) δ: 144.3, 135.0, 134.9, 129.1, 128.6, 127.4, 126.5, 126.2, 126.1, 125.9, 58.6, 49.2, 37.7; HPLC analysis using Daicel Chiralcel OJ-3 column (Hexane:^{i}PrOH = 98:2, 1.0 mL/min, 254 nm, tR = 34.5 min (*S*), 44.7 min (*R*)); $[\alpha]_D^{20}$ = −123.02 (98% ee, c 0.35, $CHCl_3$).

2-7. Mechanistic studies

2-7-1. General procedure of nonlinear effect

Experimental procedure (Supplementary Figures 1, 2)

KCH$_2$TMS (6.3 mg, 5.0 x 10^{-2} mmol) and KHMDS (10.0 mg, 5.0 x 10^{-2} mmol) were placed in a flame-dried 10 mL flask inside a glove box fulfilled with argon. The flask was cooled to –40 ºC, then diamine ligand **L6** (19.4 mg, 8.3 x 10^{-2} mmol) in toluene (0.20 mL) was added, and the chiral base mixture was stirred for 30 min at the same temperature. After the flask was cooled at –78 ºC, *p*-methoxycumylimine **1a** (2.53 g, 10.0 mmol) dissolved in toluene (0.80 mL) was successively introduced via a well-dried cannula, and the whole mixture was stirred for 18 h at the same temperature. After the reaction was quenched by adding water, the mixture was extracted with DCM (10 mL x 3). The combined organic layer was dried over anhydrous Na$_2$SO$_4$. After filtration and concentration under reduced pressure, the crude product obtained was purified by PTLC (hexane-ethyl acetate) to afford the desired adduct **3**.

2-7-2. NMR experiments

Preparation of samples (Supplementary Figures 3-5)

KCH$_2$TMS (6.3 mg, 5.0 x 10^{-2} mmol) KHMDS (10.0 mg, 5.0 x 10^{-2} mmol) were placed in a flame-dried 10 mL flask inside a glove box fulfilled with argon. The flask was cooled to –40 ºC, then diamine ligand **L2** (12.8 mg, 5.5 x 10^{-2} mmol) in toluene-d$_8$ (0.20 mL) was added, and the mixture was stirred for 30 min at the same temperature. After the flask was cooled to –78 ºC, the mixture was diluted with toluene-d8 (0.80 mL) and was successively transferred into a screw-cap NMR tube via a well-dried cannular. Subsequently, NMR measurement was conducted with keeping –78 ºC.

2-7-3. Effect of the piperazine part

General procedure for ligand structure activity (Supplementary Table 4)

KCH$_2$SiMe$_3$ (6.3 mg, 5.0 x 10^{-2} mmol) and KHMDS (10.0 mg, 5.0 x 10^{-2} mmol) were placed in a flame-dried 10 mL flask inside a glove box fulfilled with argon. After the flask was cooled to the corresponding temperature, the corresponding amine ligand **L** (5.5 x 10^{-2} mmol) in toluene (0.40 mL) was added, and the mixture was stirred at –40 °C for 30 minutes. Subsequently, the flask was cooled to –78 °C, and the corresponding imine **1a** (126.7 mg, 0.50 mmol) dissolved in toluene (0.60 mL) was successively introduced into the flask via a well-dried cannula. After the whole mixture was stirred for 18 h at the same temperature, the reaction was quenched by adding a few drops of MeOH, and the obtained mixture was extracted with DCM (10 mL x 3), then combined organic layer was dried over anhydrous Na$_2$SO$_4$. After filtration and concentration under reduced pressure, the obtained crude product was purified by PTLC (hexane-ethyl acetate) to afford the desired amine **3aa**. The enantioselectivities were determined by HPLC.

2-7-4. Investigation of backward reaction

Experimental procedure (Supplementary Figure 6)

KCH_2SiMe_3 (6.3 mg, 5.0 x 10^{-2} mmol) and KHMDS (10.0 mg, 5.0 x 10^{-2} mmol) were placed in a flame-dried 10 mL flask inside a glove box fulfilled with argon. After the flask was cooled to the corresponding temperature, the corresponding amine ligand **L** (5.5 x 10^{-2} mmol) in toluene (0.40 mL) was added, and the mixture was stirred at –40 °C for 30 minutes. Subsequently, the flask was cooled to –78 °C, and the corresponding imine **3aa** (126.7 mg, 0.50 mmol, 99% ee) dissolved in toluene (0.60 mL) was successively introduced into the flask via a well-dried cannula. After the whole mixture was stirred for 18 h at the same temperature, the reaction was quenched by adding a few drops of MeOH, and the obtained mixture was extracted with DCM (10 mL x 3), then combined organic layer was dried over anhydrous Na_2SO_4. After filtration and concentration under reduced pressure, the obtained crude product was purified by PTLC (hexane-ethyl acetate) to afford the desired amine **3aa** (93% yield). The enantiopurity was determined by HPLC as 99% ee.

3. References

1. W. Clegg, B. Conway, D. V. Graham, E. Hevia, A. R. Kennedy, R. E. Mulvey, L. Russo and D. S. Wright, *Chem. Eur. J.*, 2009, **15**, 7074.
2. Y. Yamashita, I. Sato, H. Suzuki and S. Kobayashi, *Chem. Asian J.*, 2015, **10**, 2143.
3. B. E. Rossiter, M. Eguchi, G. Miao, N. M. Swingle, A. E. Hernández, D. Vickers, E. Fluckiger, R. G. Patterson and K.V. Reddy, *Tetrahedron*, 1993, **49**, 965.
4. R. Shirai, K. Aoki, D. Sato, Daisaku, H.-D. Kim, M. Murakata, T. Yasukata and K. Koga, *Chem. Pharm. Bull.* 1994, **42**, 690.
5. Y. Yamashita, H. Suzuki, I. Sato, T. Hirata and S. Kobayashi, *Angew. Chem. Int. Ed.*, 2018, **57**, 6896.
6. K. Bojaryn, C. Hoffmann, F. R. Struth and C. Hirschhäuser, *Synlett*, 2018, **29,** 1092.
7. H.-J. Choi, M.-O. Kwak and H. Song, *Synthetic Communications*, 1997, **27**, 1273.
8. V. Kurteva and M. Lyapova, *Cent. Eur. J. Chem.*, 2004, **2**, 686.
9. C. K. De, E. G. Klauber and D. Seidel, *J. Am. Chem. Soc.*, 2009, **131**, 17060.
10. G. Liu, P. J. Walsh and J. Mao, *Org. Lett.*, 2019, **21**, 8514.
11. P. Arigala, V. S. Sadu, I.-T. Hwang, J.-S. Hwang, C.-U. Kim, K.-I. Lee, *Adv. Synth. Catal.*, 2015, **357**, 2027.
12. L.-G. Xie and D. J. Dixon, *Chem. Sci.*, 2017, **8**, 7492.

4. NMR and HPLC charts

Supplementary Figure 7 ^{1}H NMR of **1d**

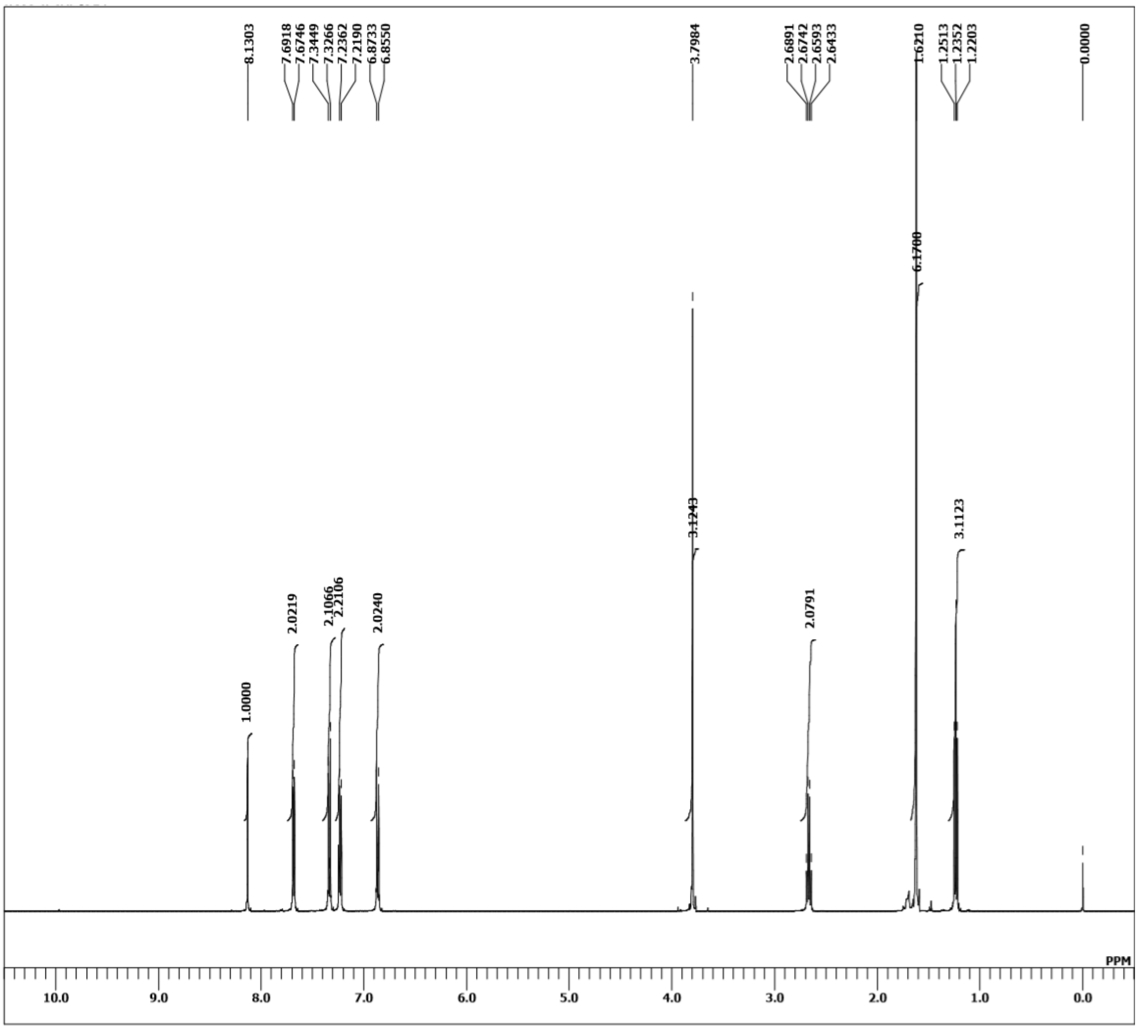

Supplementary Figure 8 ^{13}C NMR of **1d**

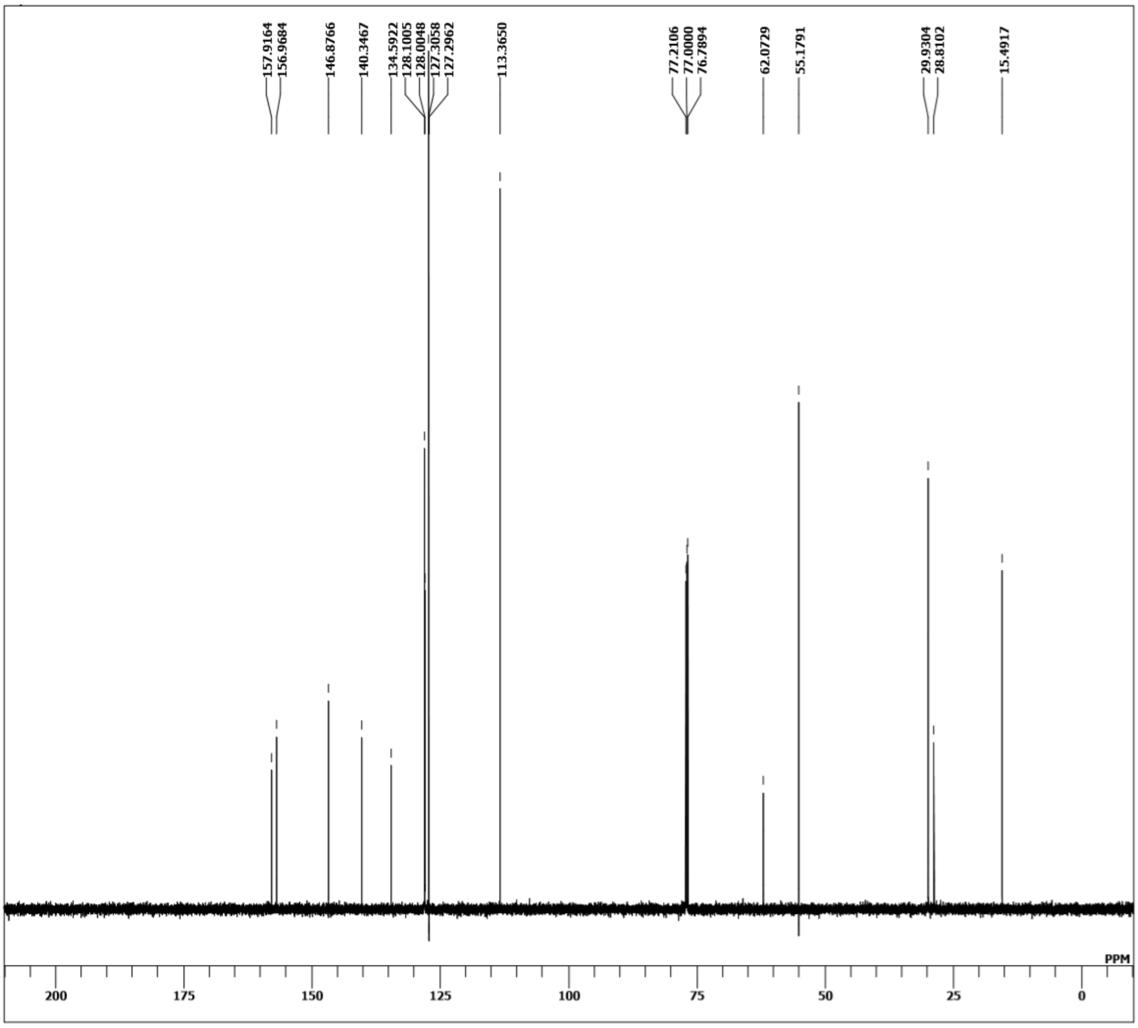

Supplementary Figure 9 ^{1}H NMR of **1e**

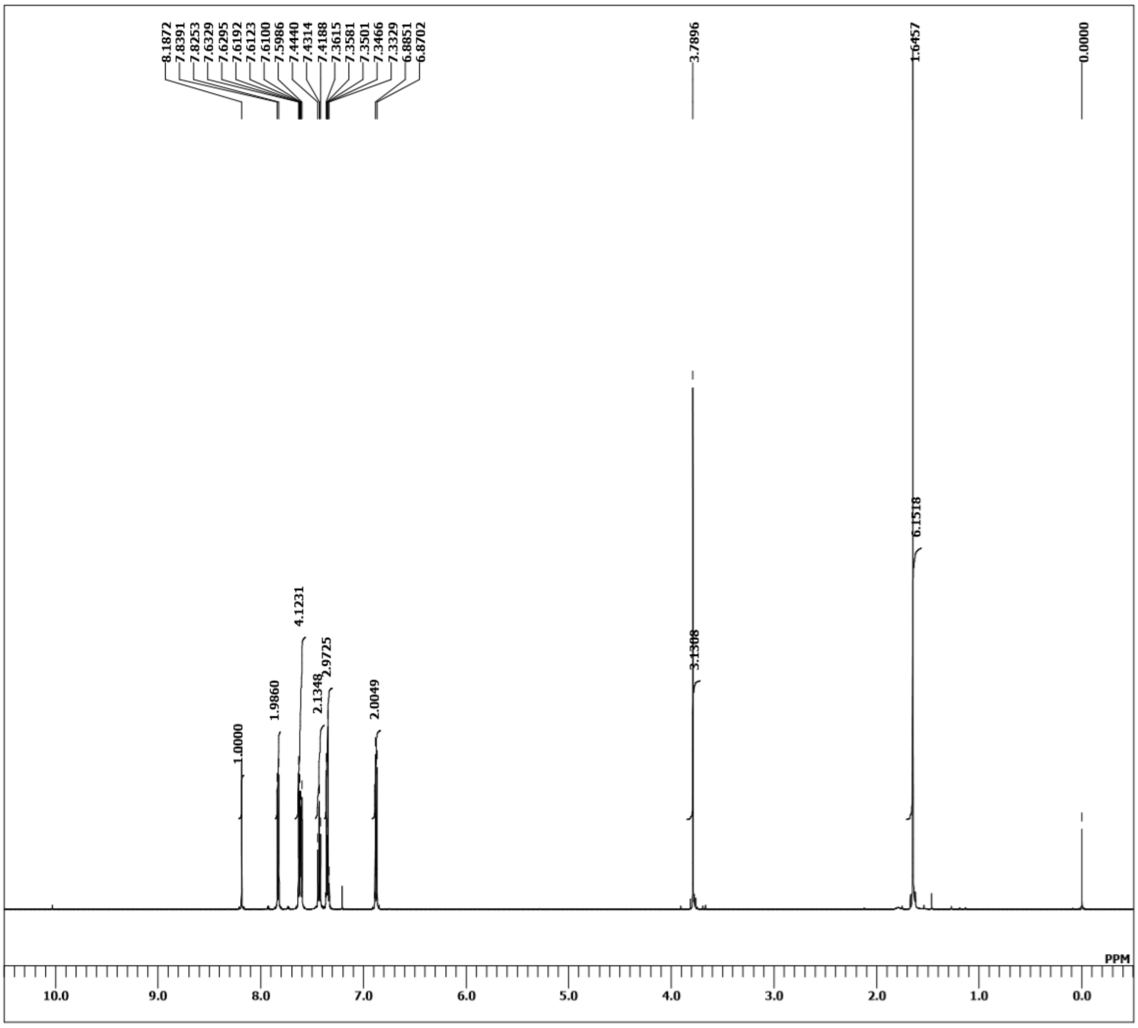

Supplementary Figure 10 ^{13}C NMR of **1e**

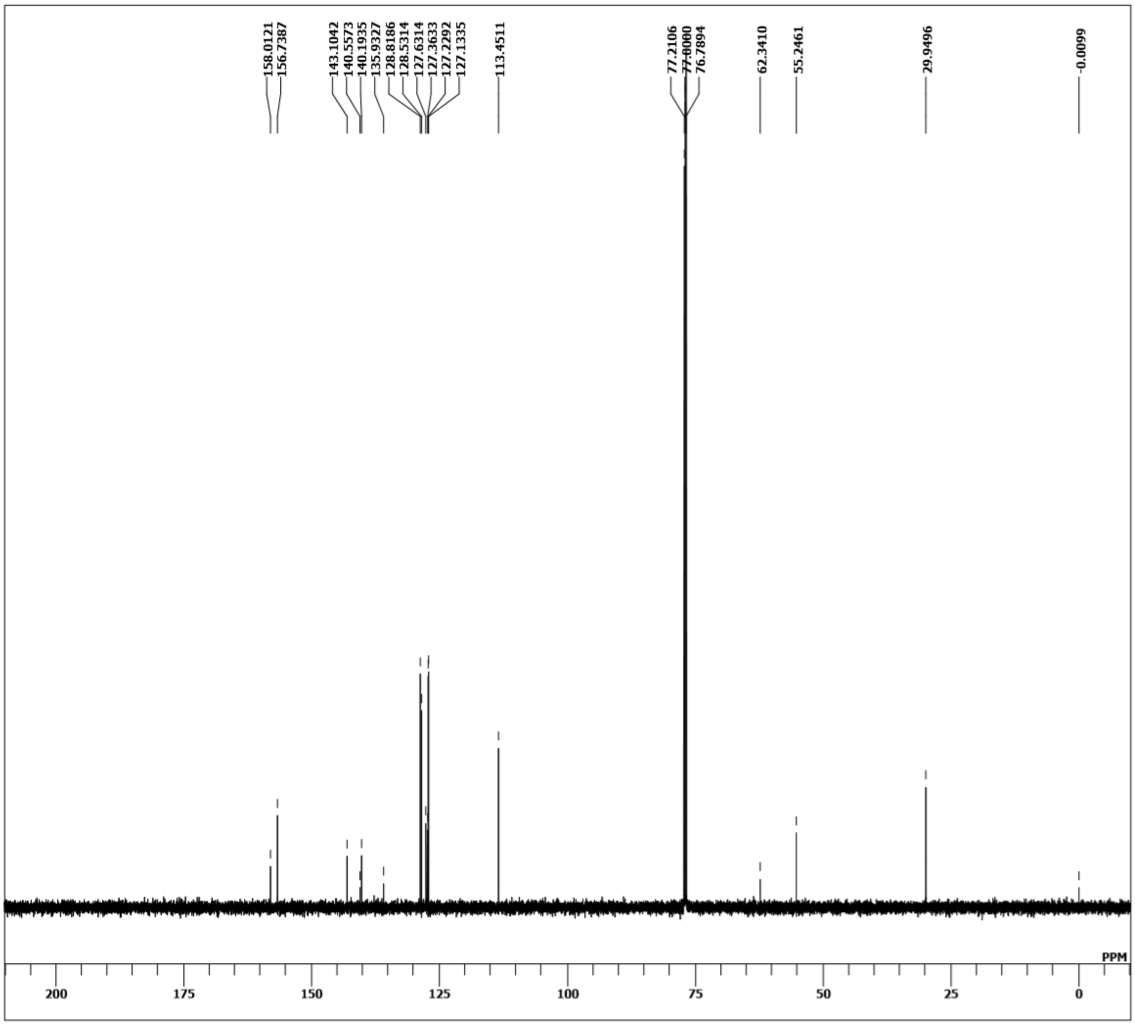

Supplementary Figure 11 ^{1}H NMR of **1f**

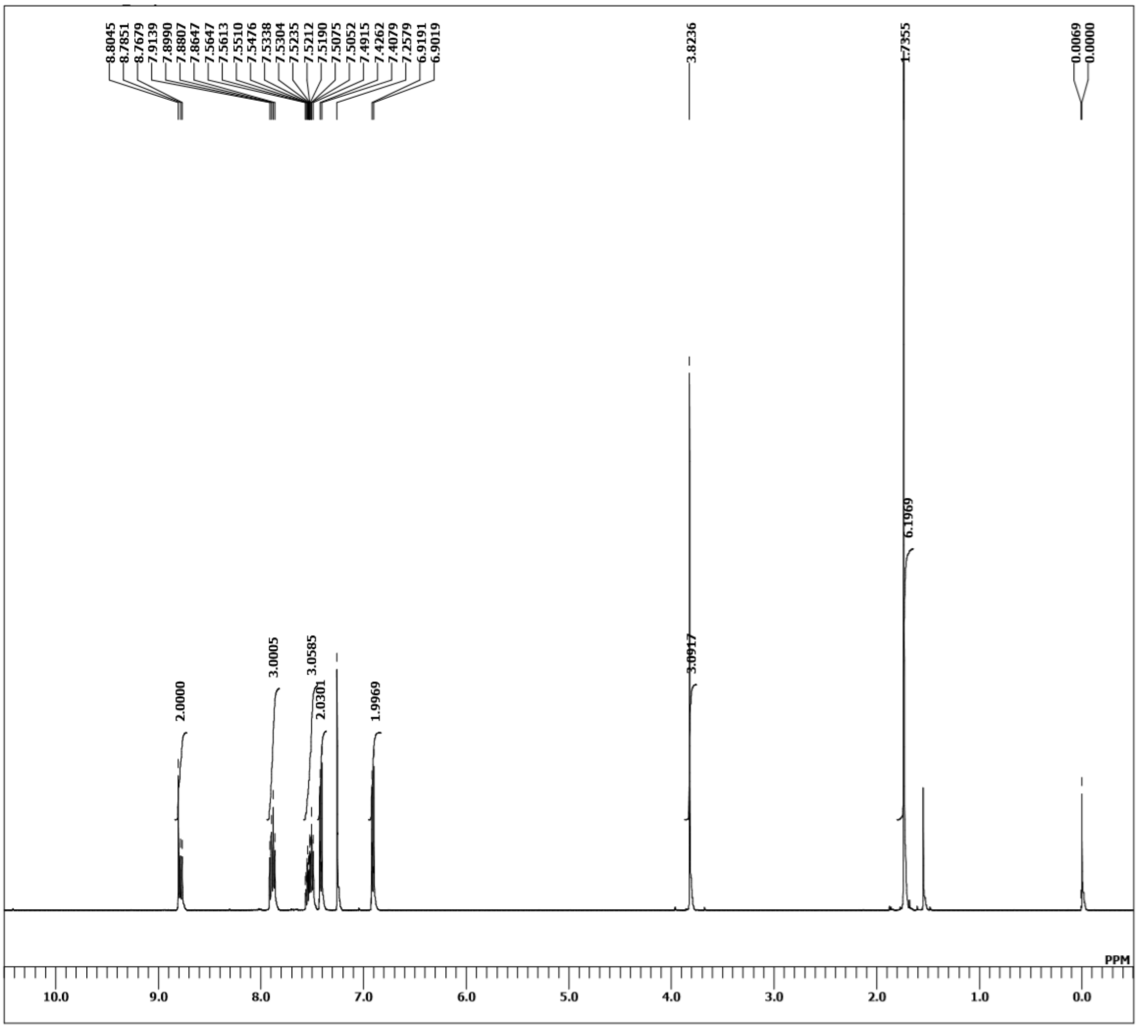

Supplementary Figure 12 ^{13}C NMR of **1f**

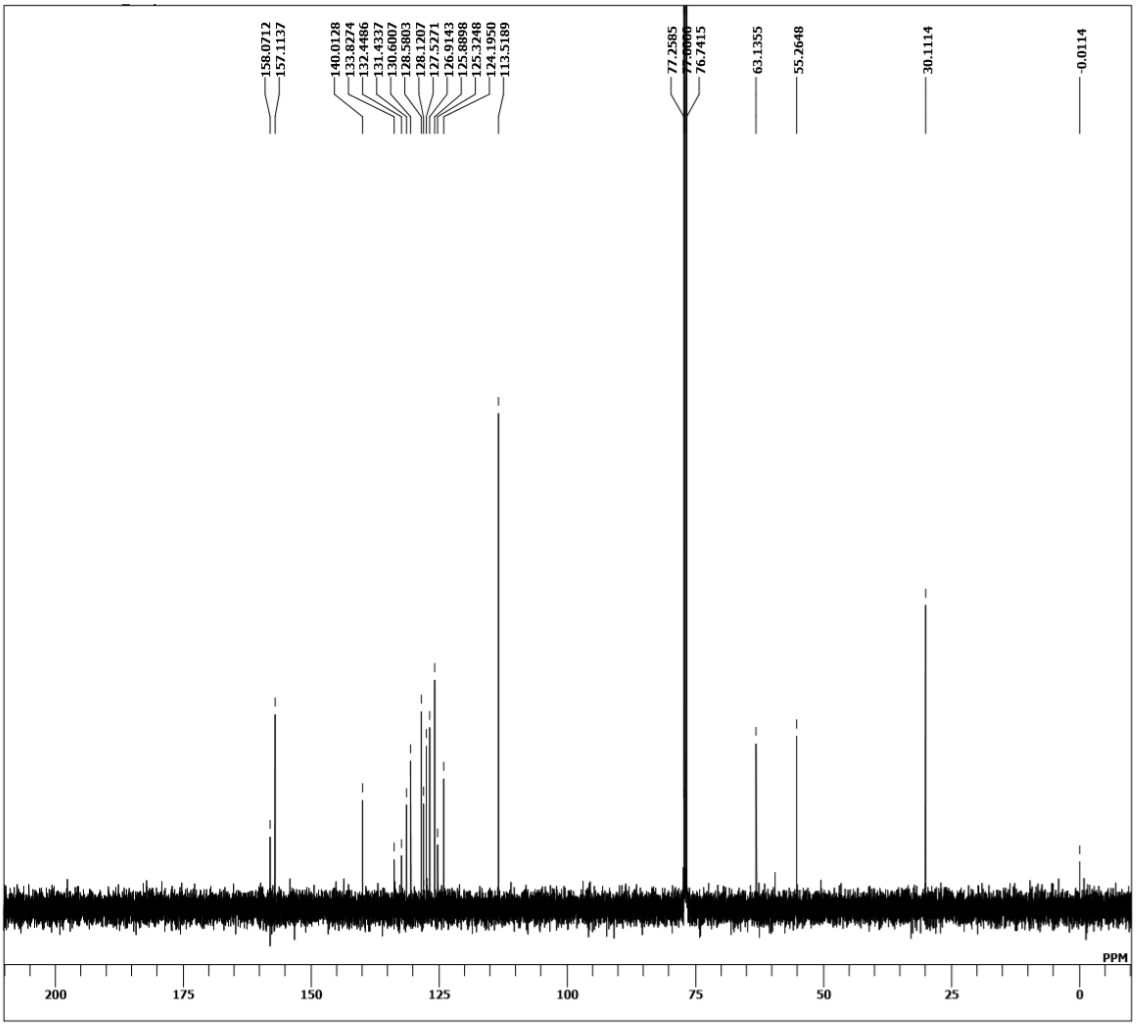

Supplementary Figure 13 ^{1}H NMR of **1k**

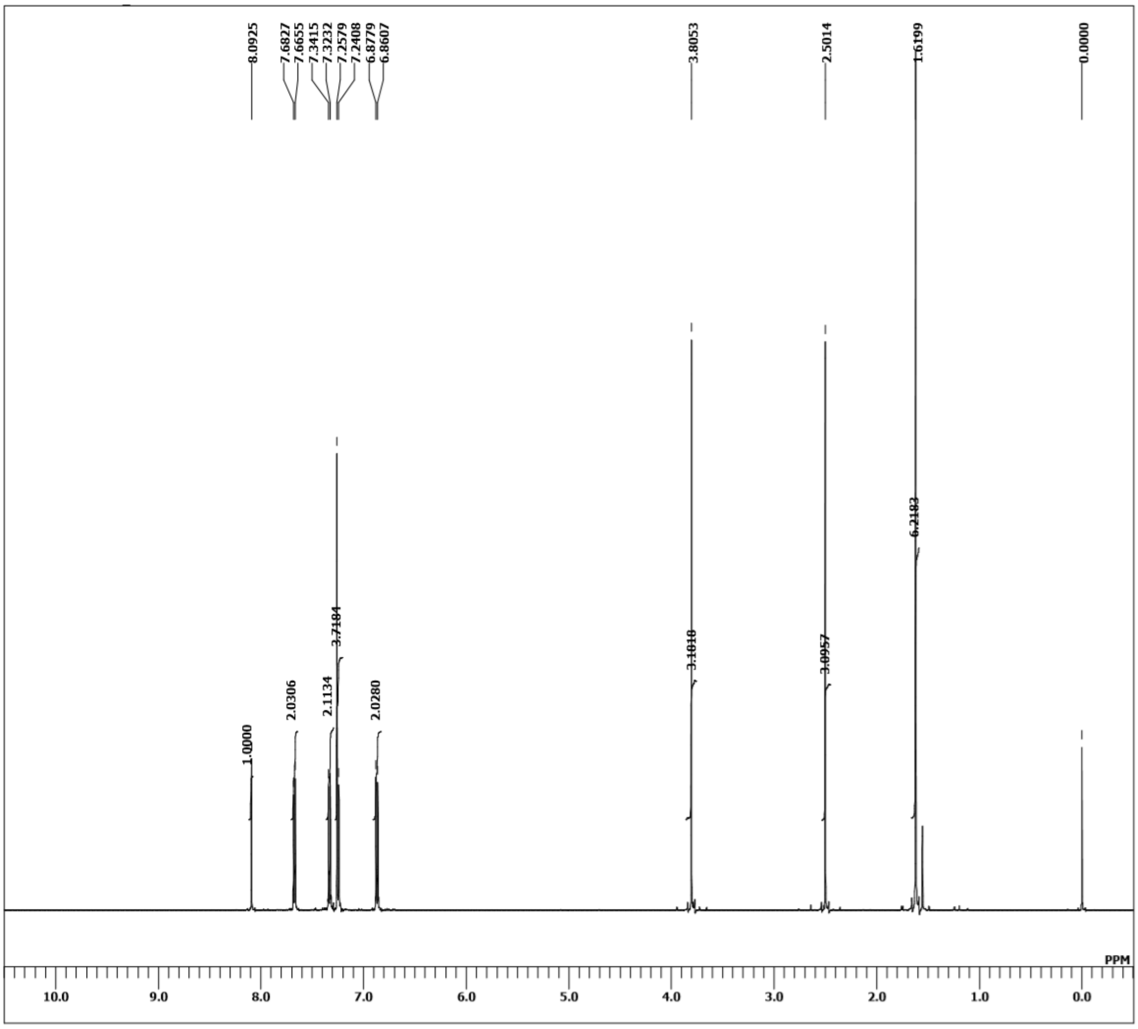

Supplementary Figure 14 ^{13}C NMR of **1k**

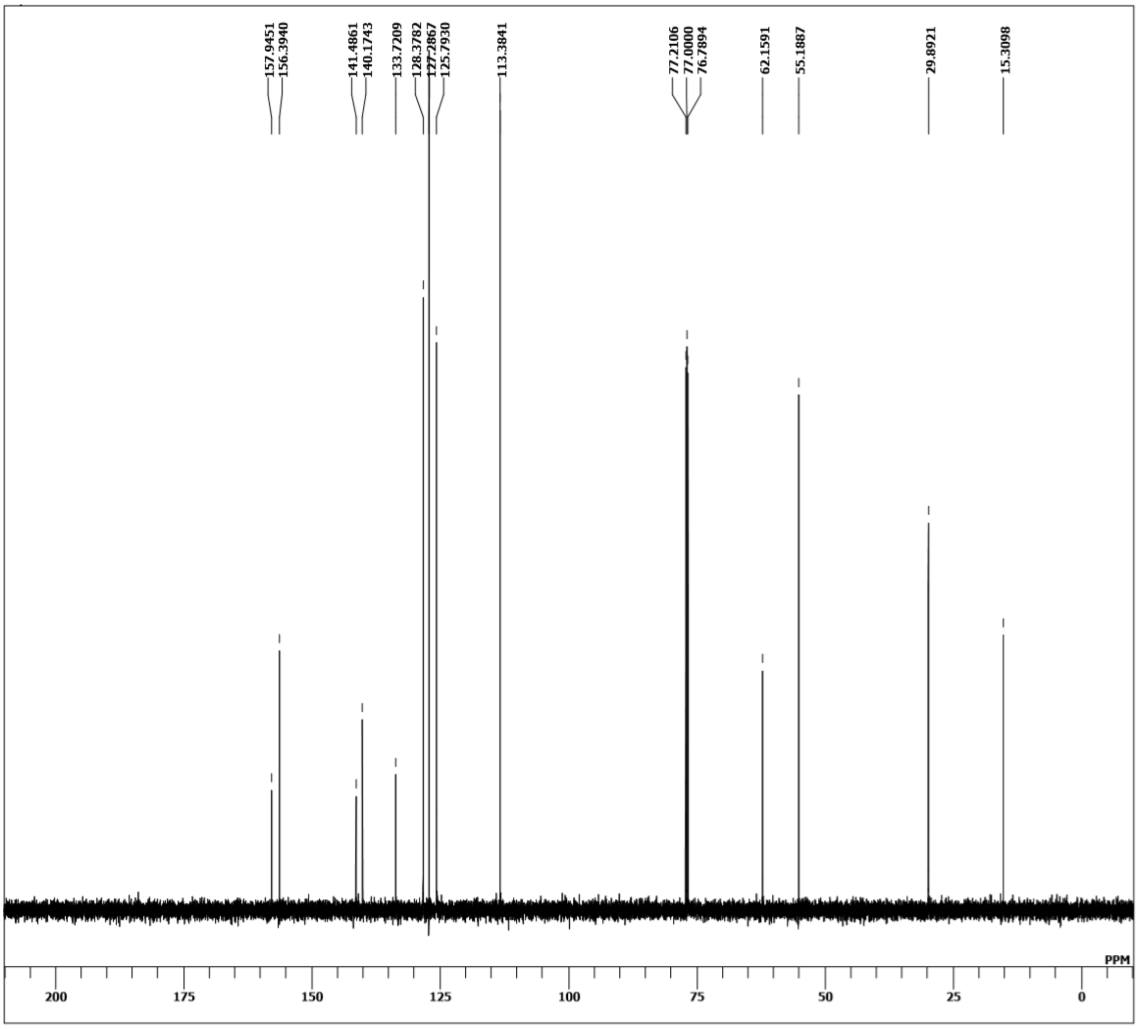

Supplementary Figure 15 ^{1}H NMR of **L4**

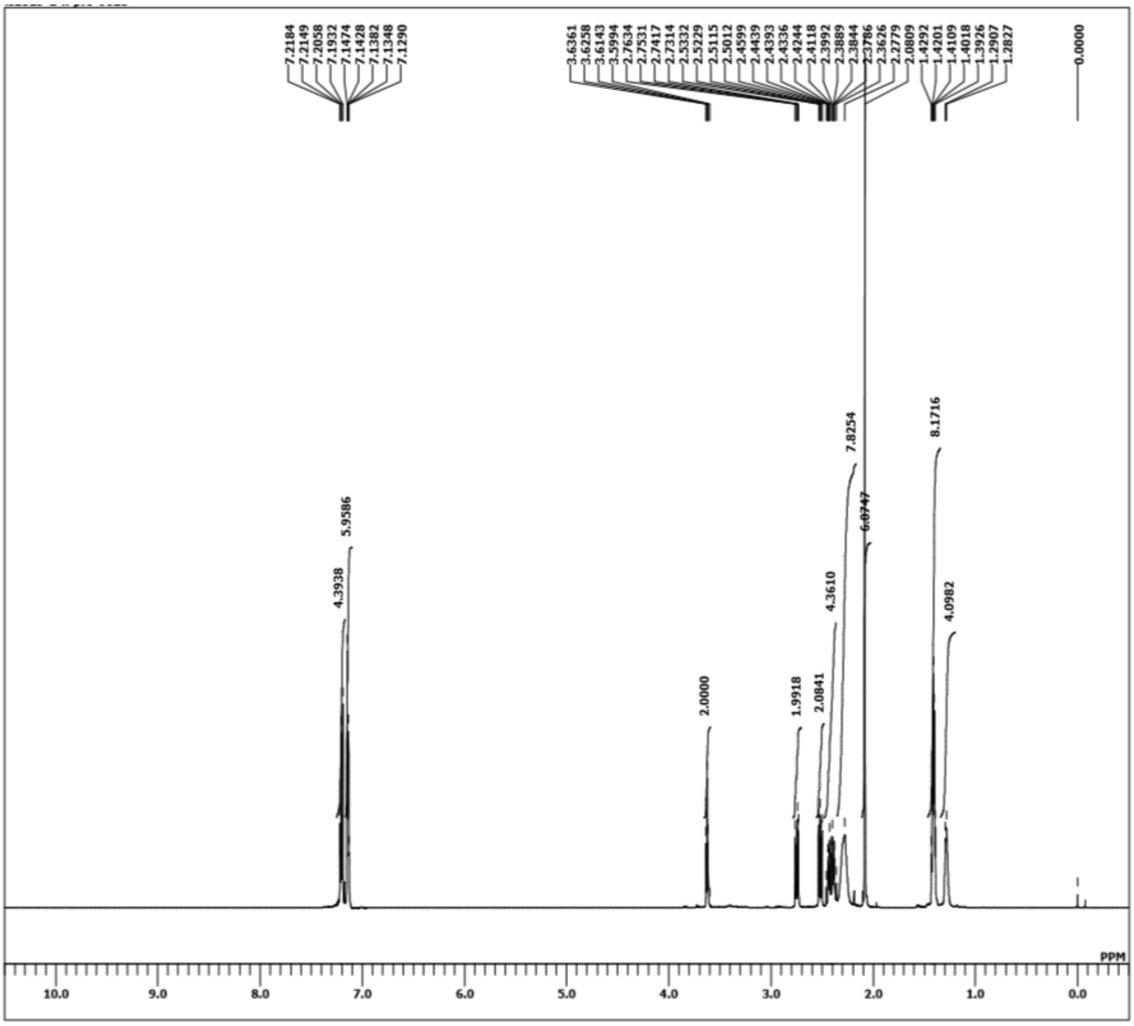

Supplementary Figure 16 ^{13}C NMR of **L4**

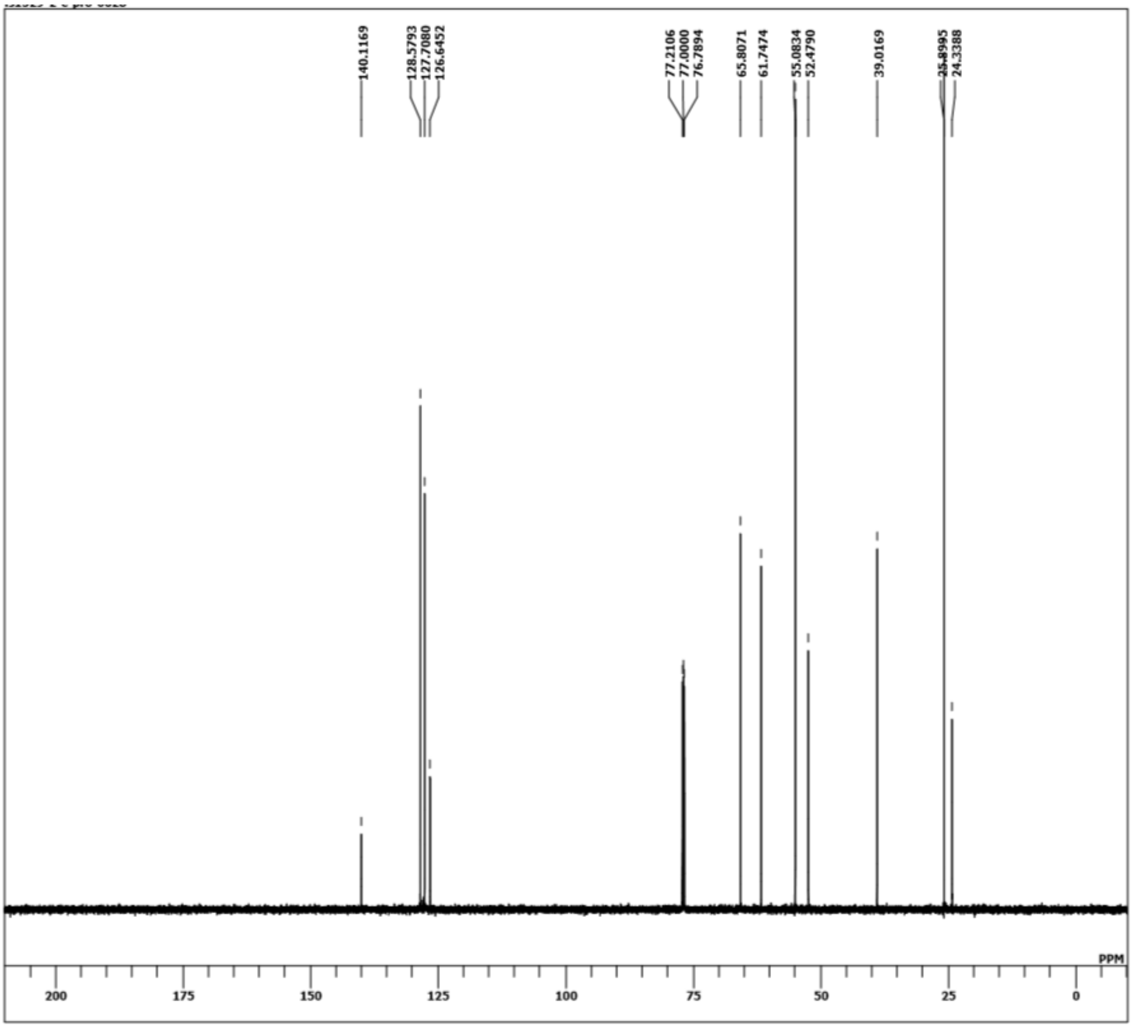

Supplementary Figure 17 ^{1}H NMR of **L5**

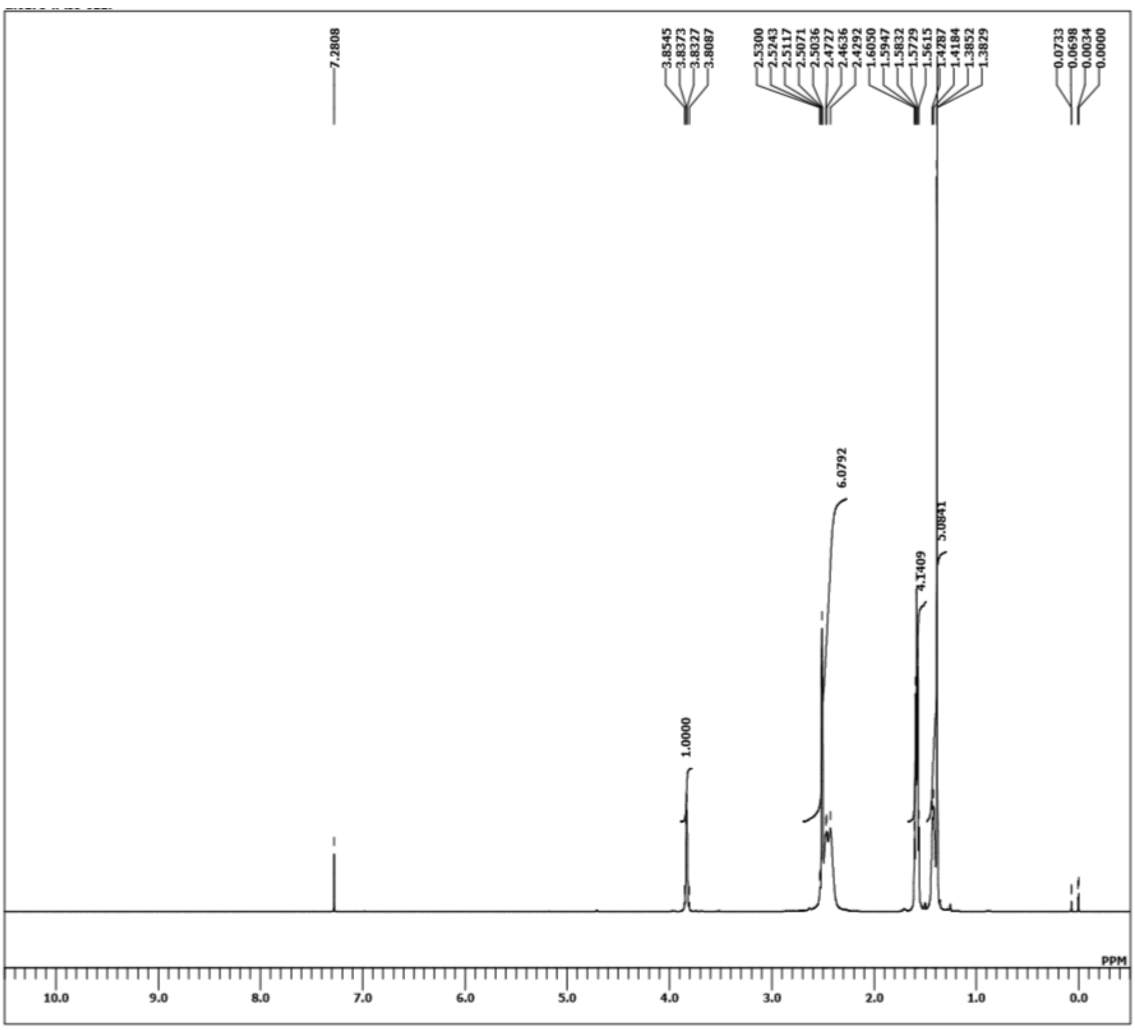

Supplementary Figure 18 ^{13}C NMR of **L5**

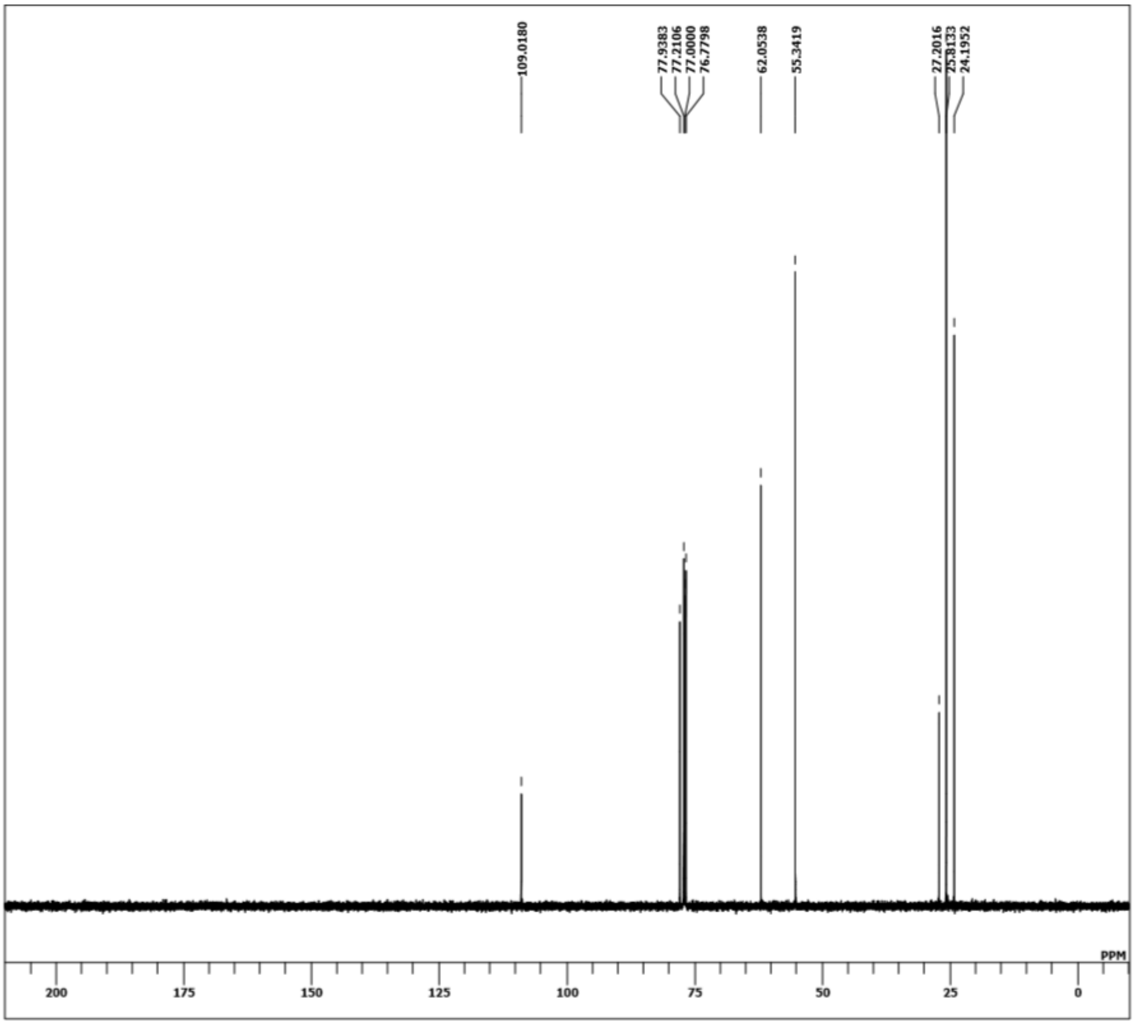

Supplementary Figure 19 ^{1}H NMR of **L6**

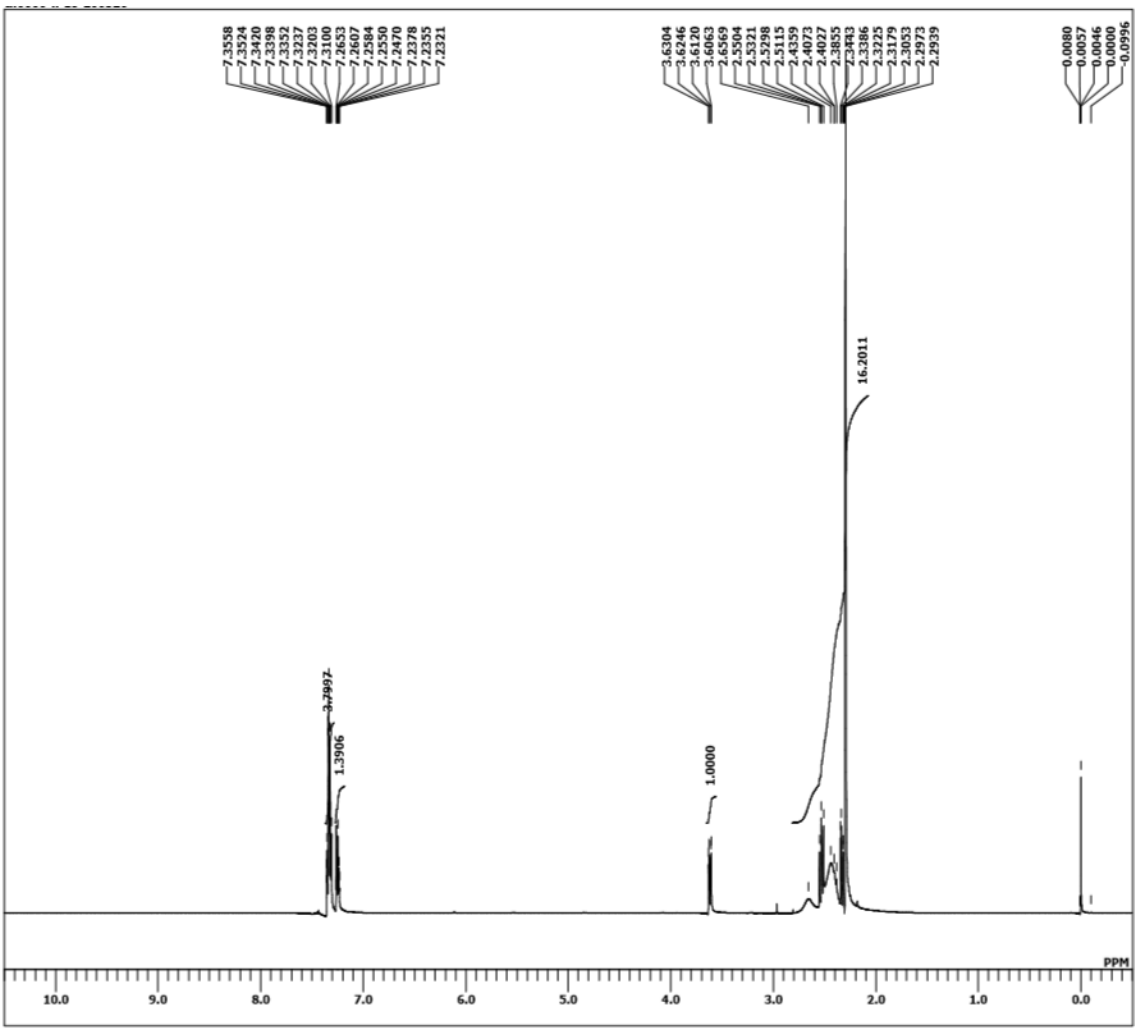

Supplementary Figure 20 ^{13}C NMR of **L6**

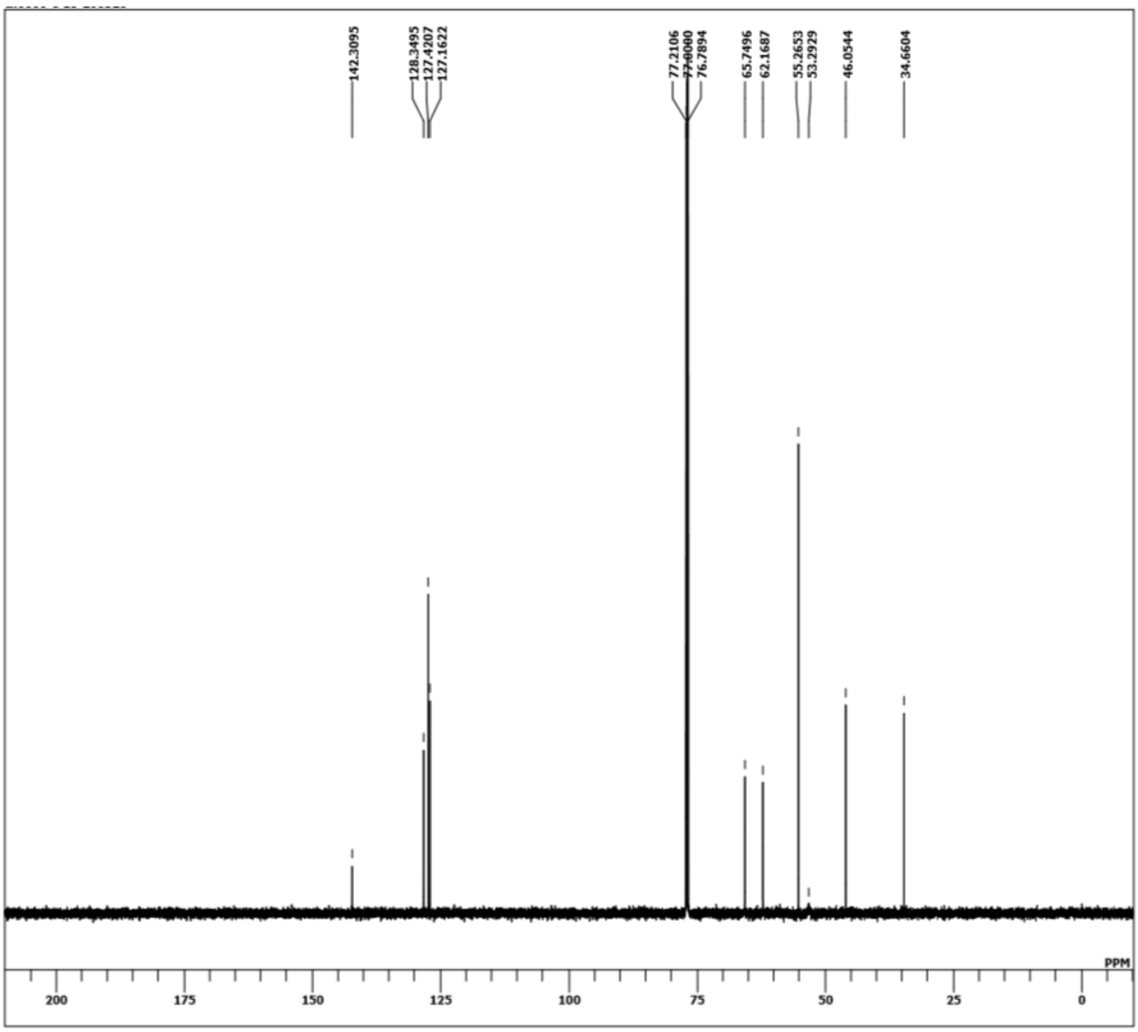

Supplementary Figure 21 ^{1}H NMR of **L10**

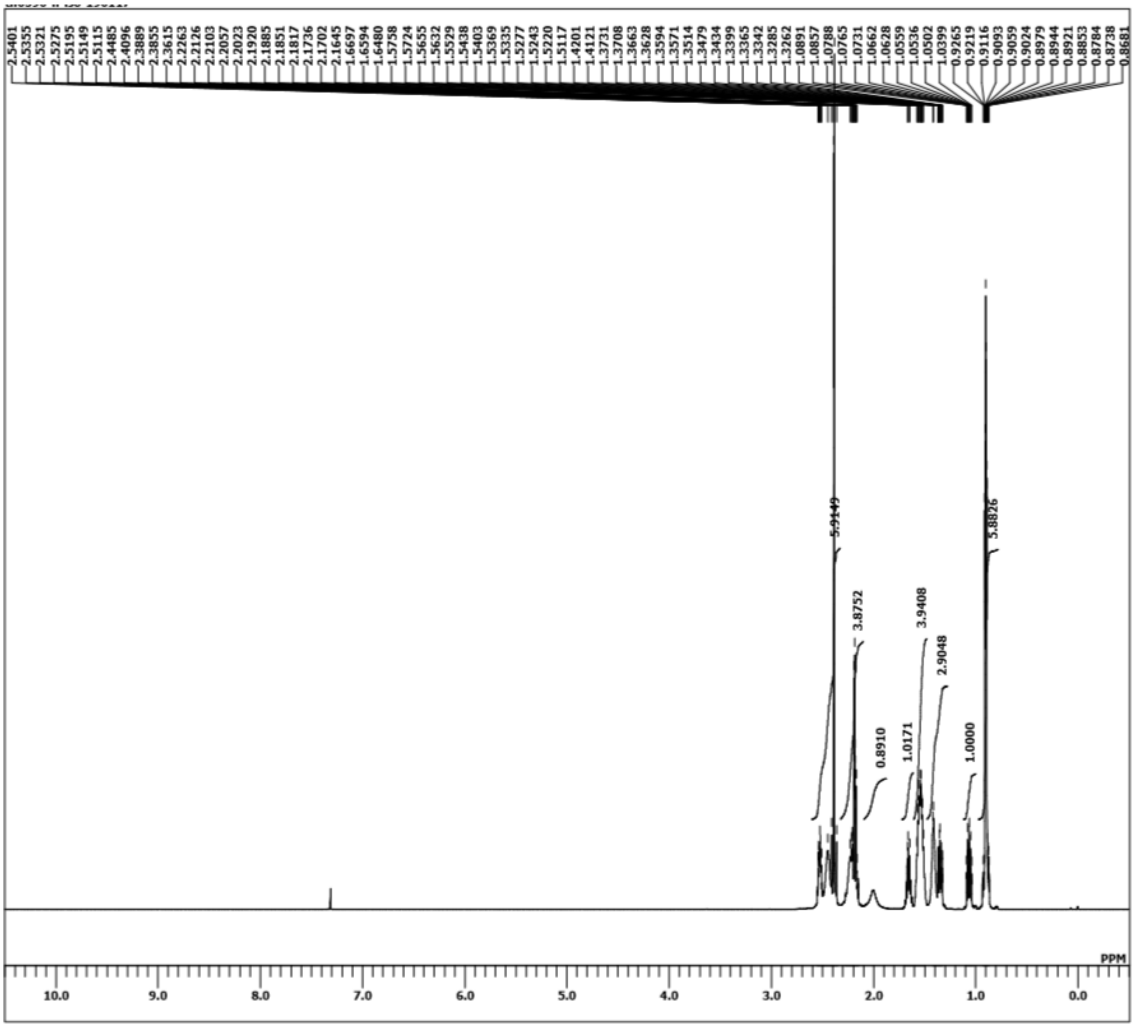

Supplementary Figure 22 ^{13}C NMR of **L10**

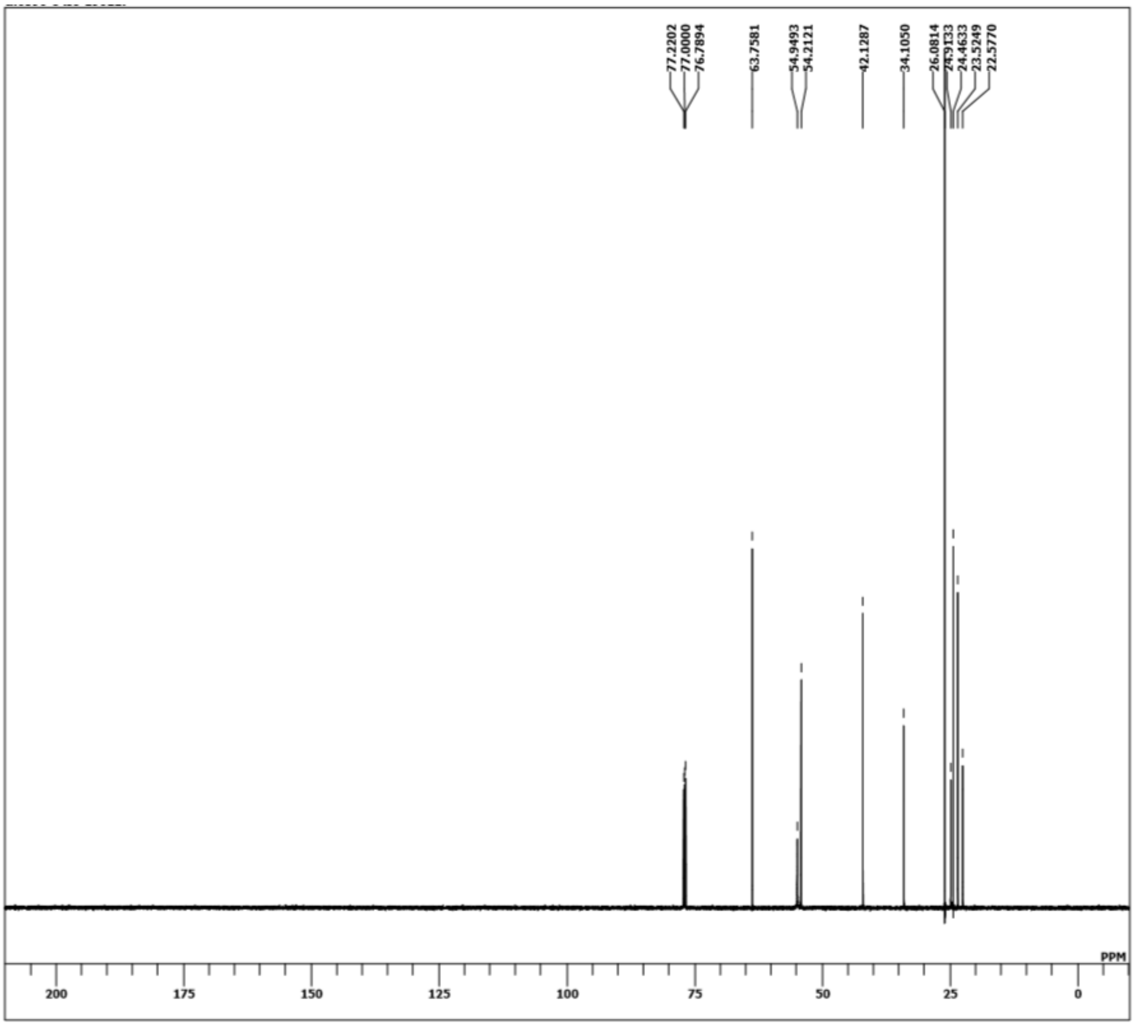

Supplementary Figure 23 ^{1}H NMR of **L12**

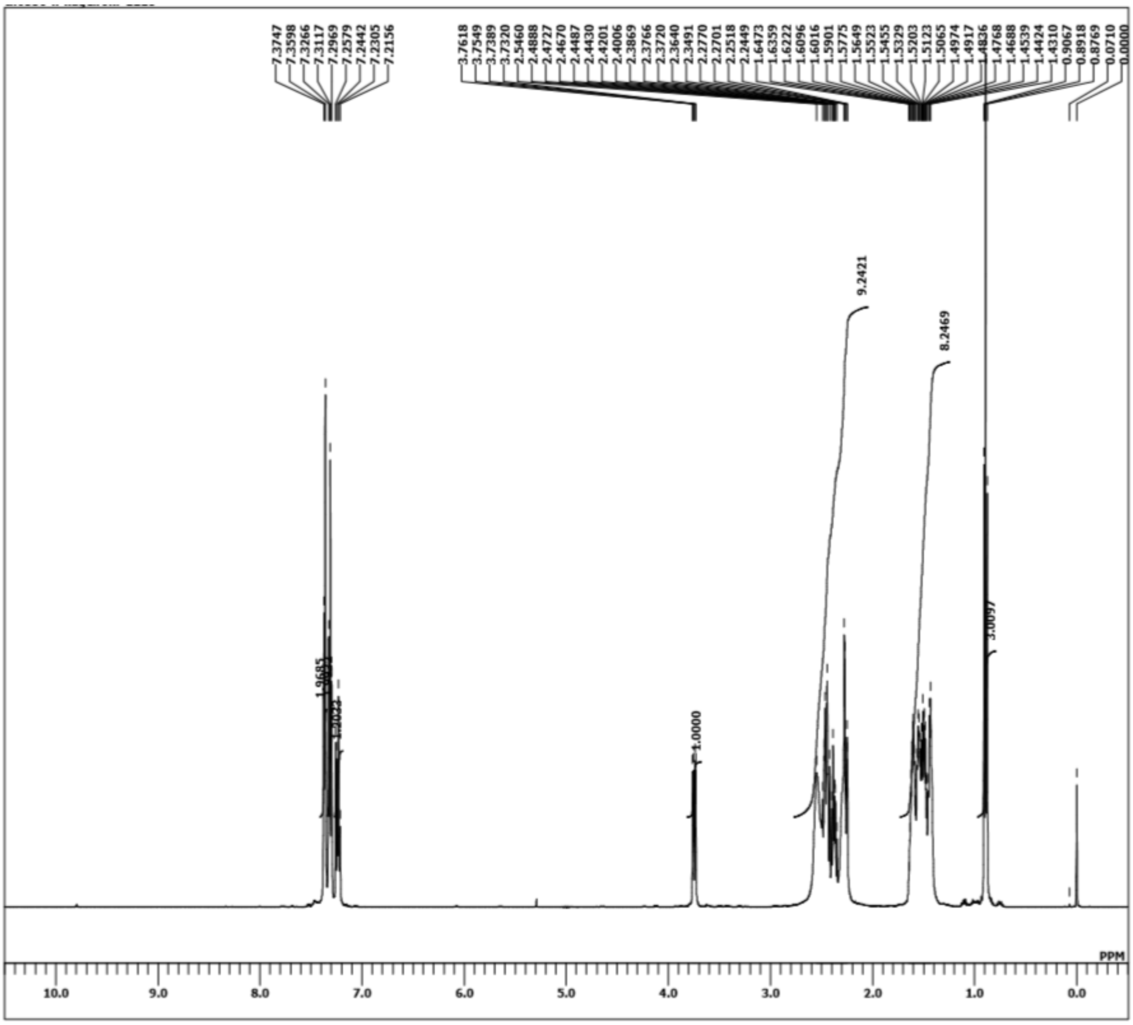

Supplementary Figure 24 ^{13}C NMR of **L12**

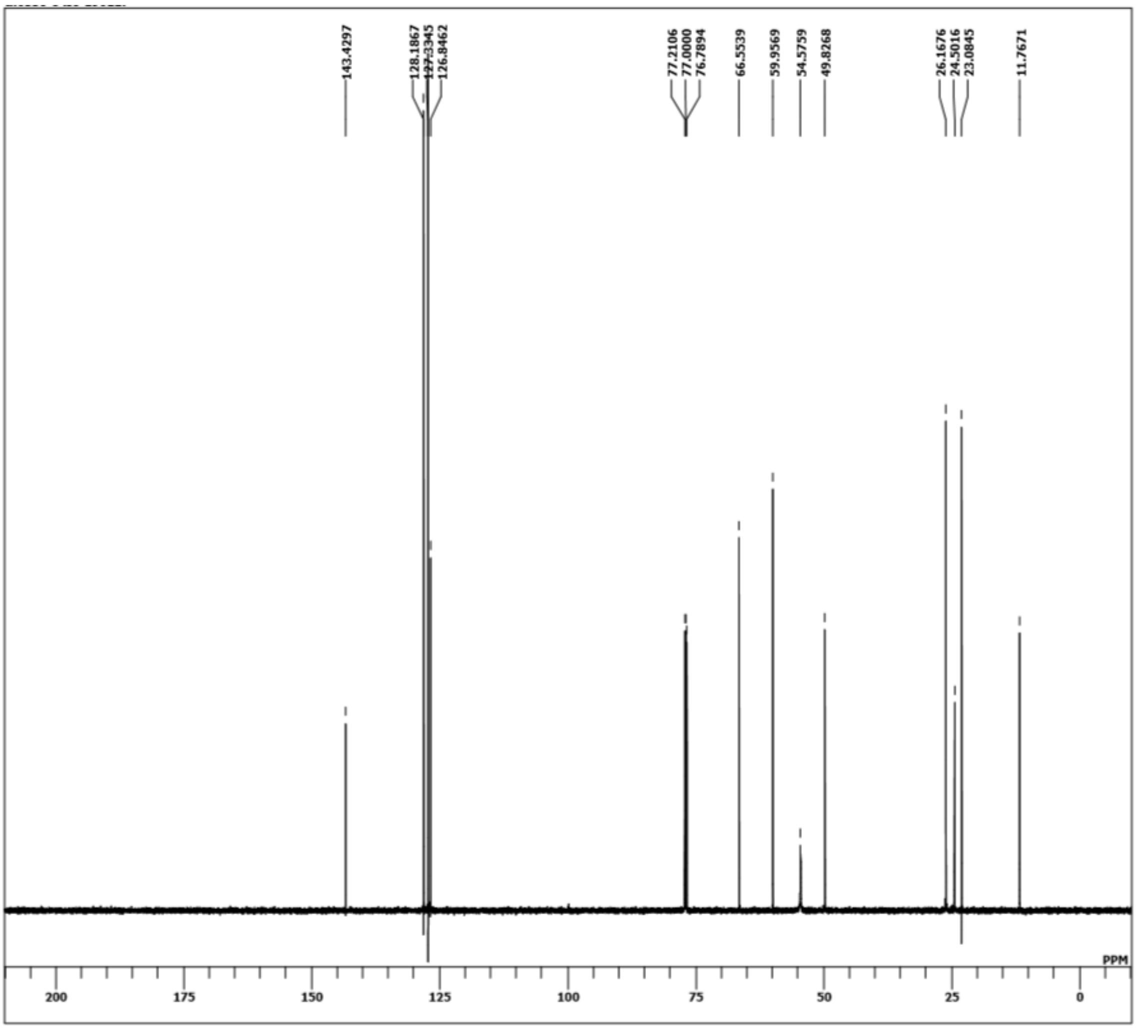

Supplementary Figure 25 ^{1}H NMR of **L17**

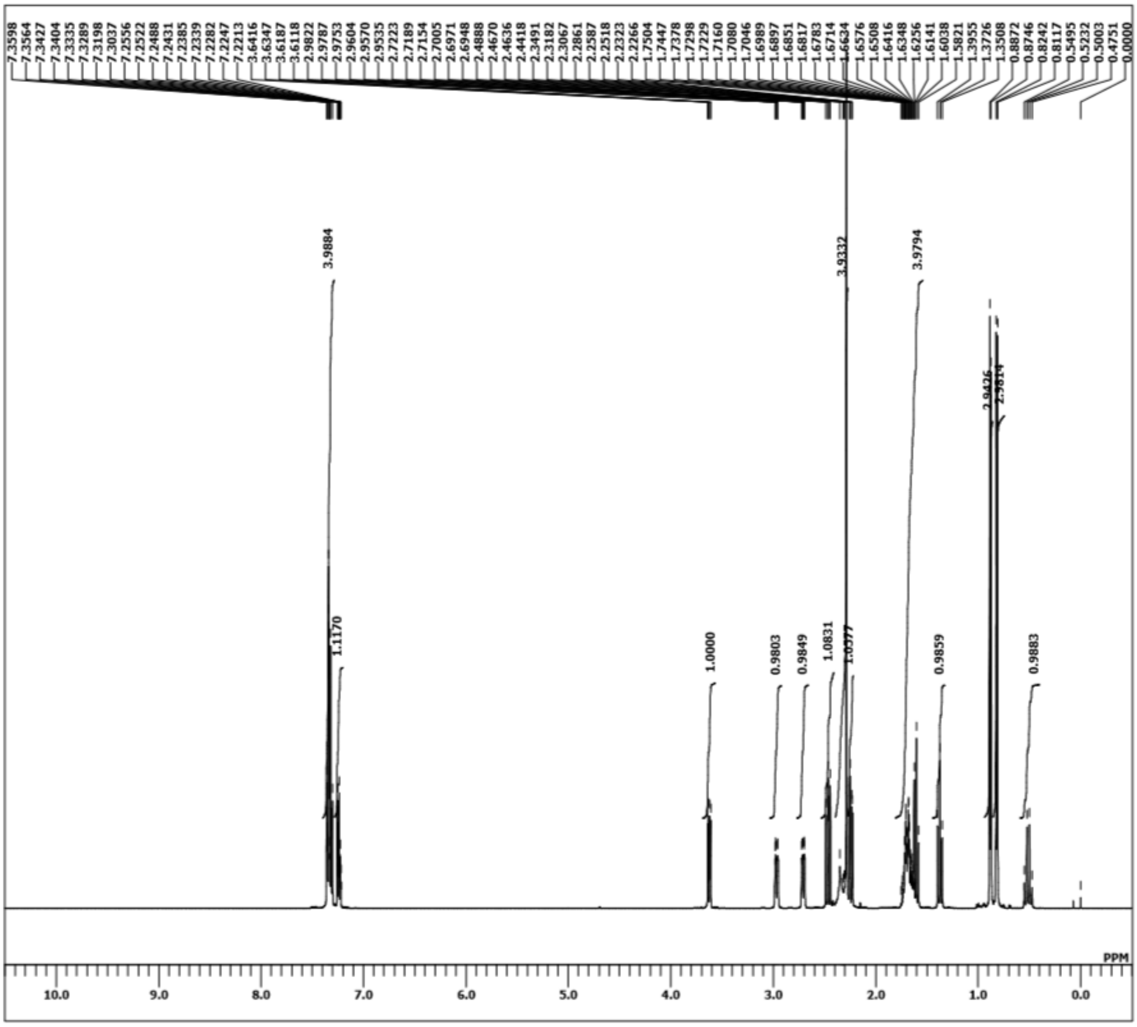

Supplementary Figure 26 ^{13}C NMR of **L17**

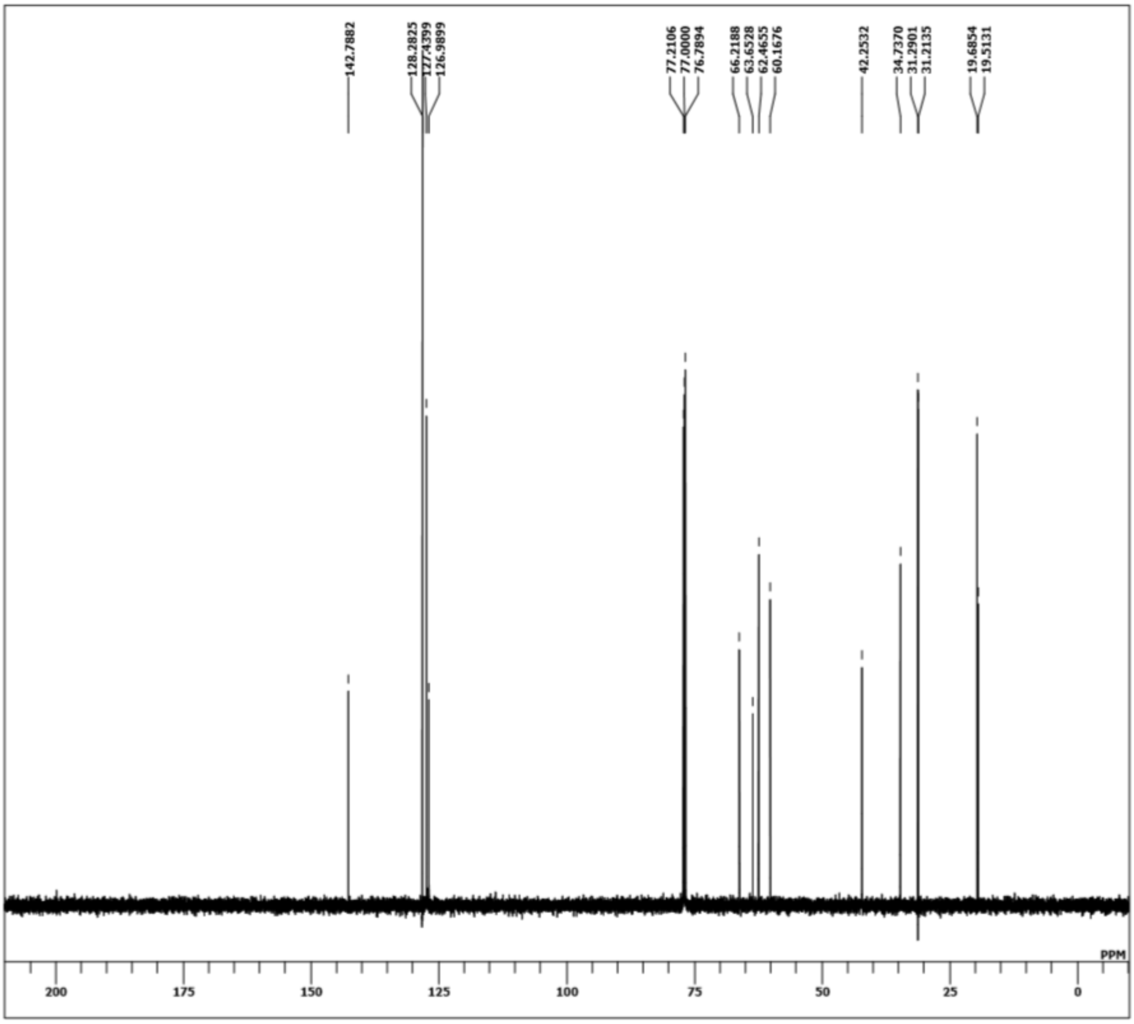

Supplementary Figure 26 ^{1}H NMR of **L19**

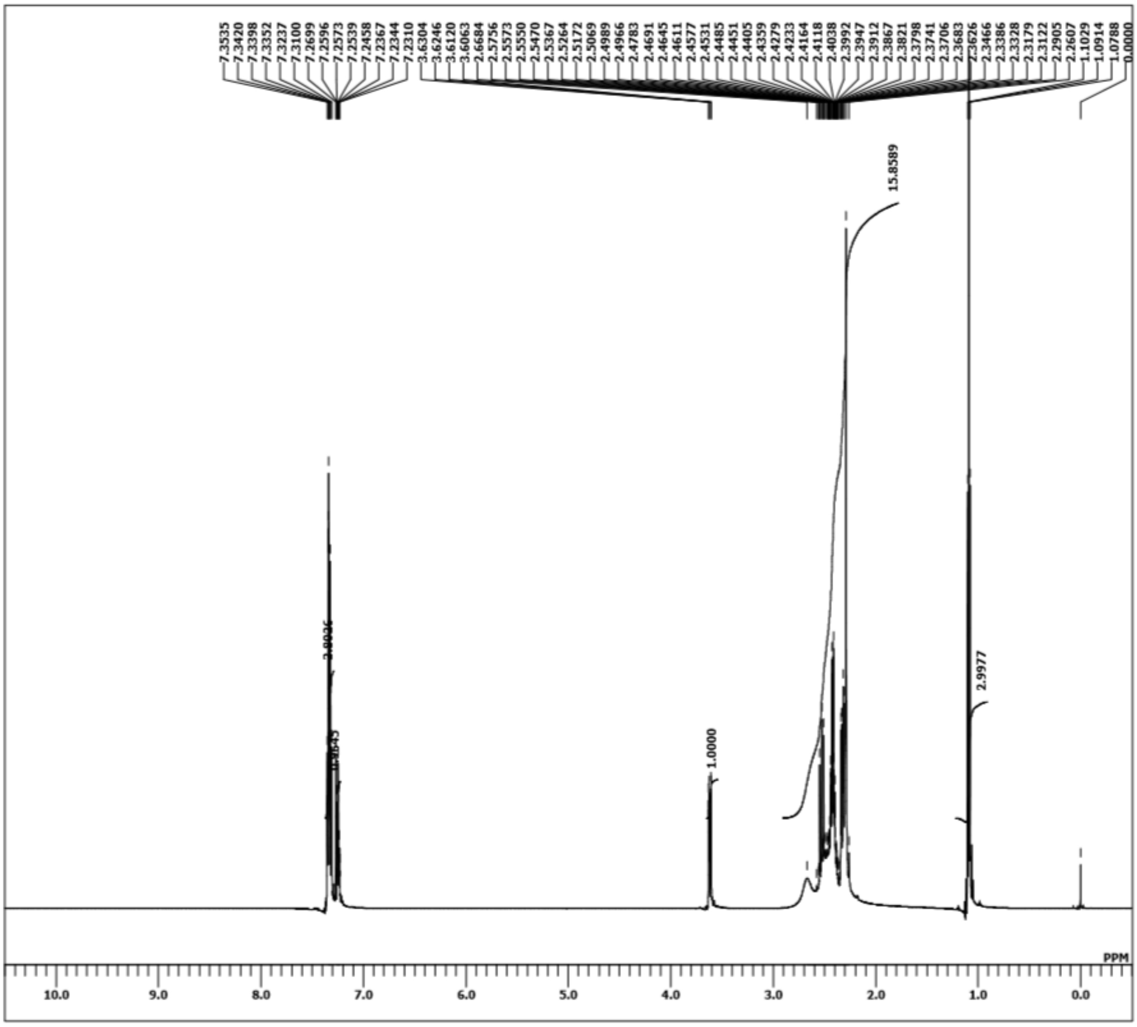

Supplementary Figure 27 ^{13}C NMR of **L19**

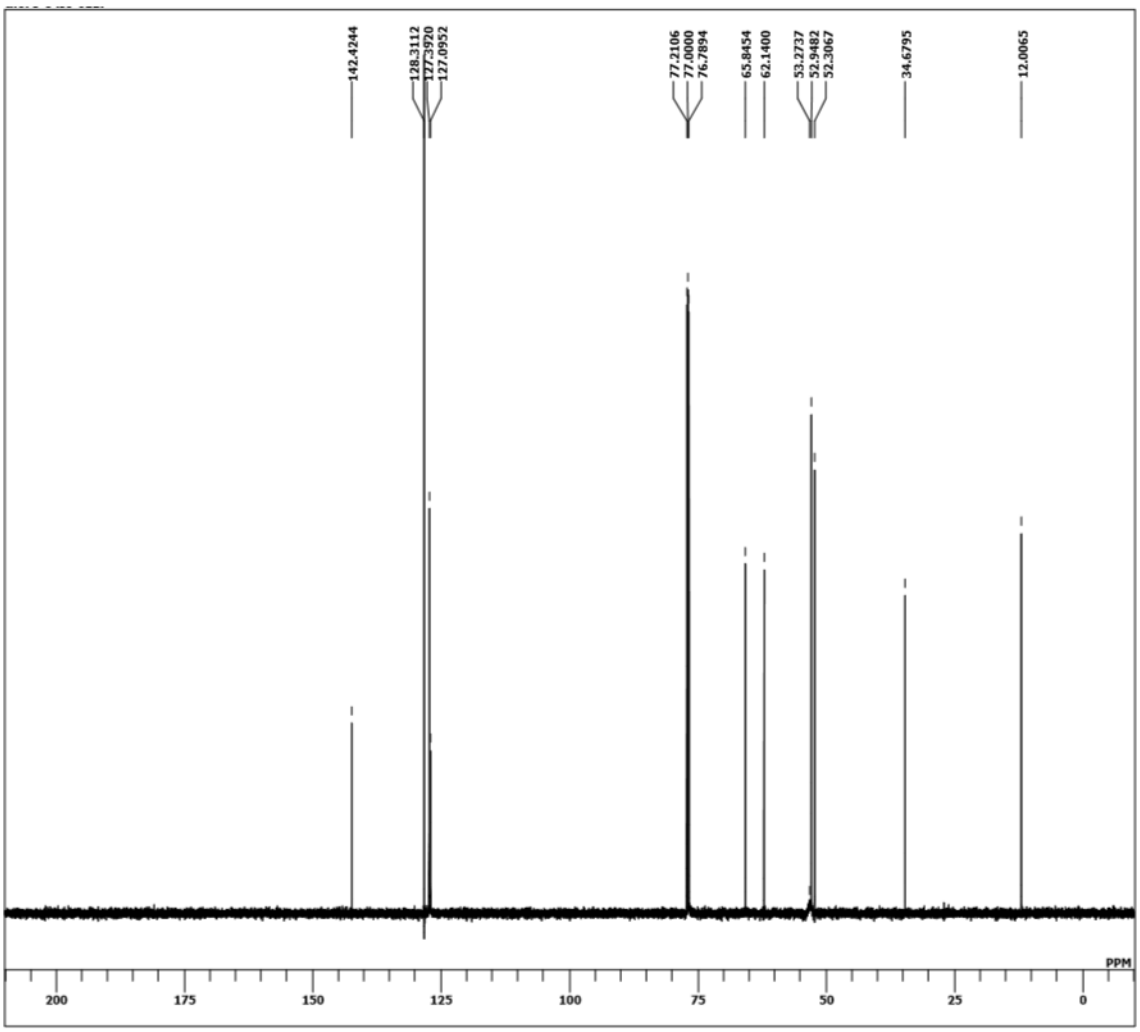

Supplementary Figure 28 ^{1}H NMR of **L20**

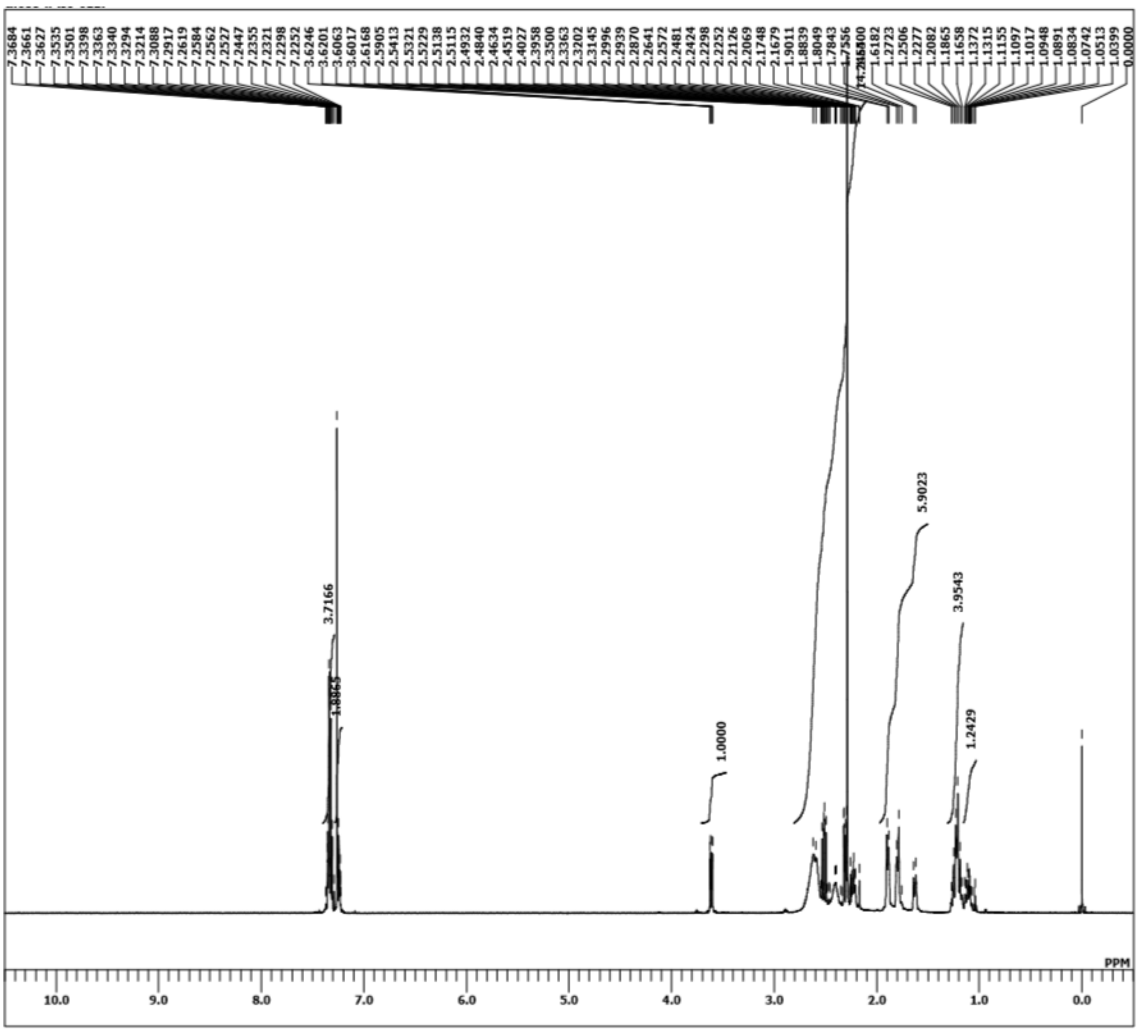

Supplementary Figure 29 ^{13}C NMR of **L20**

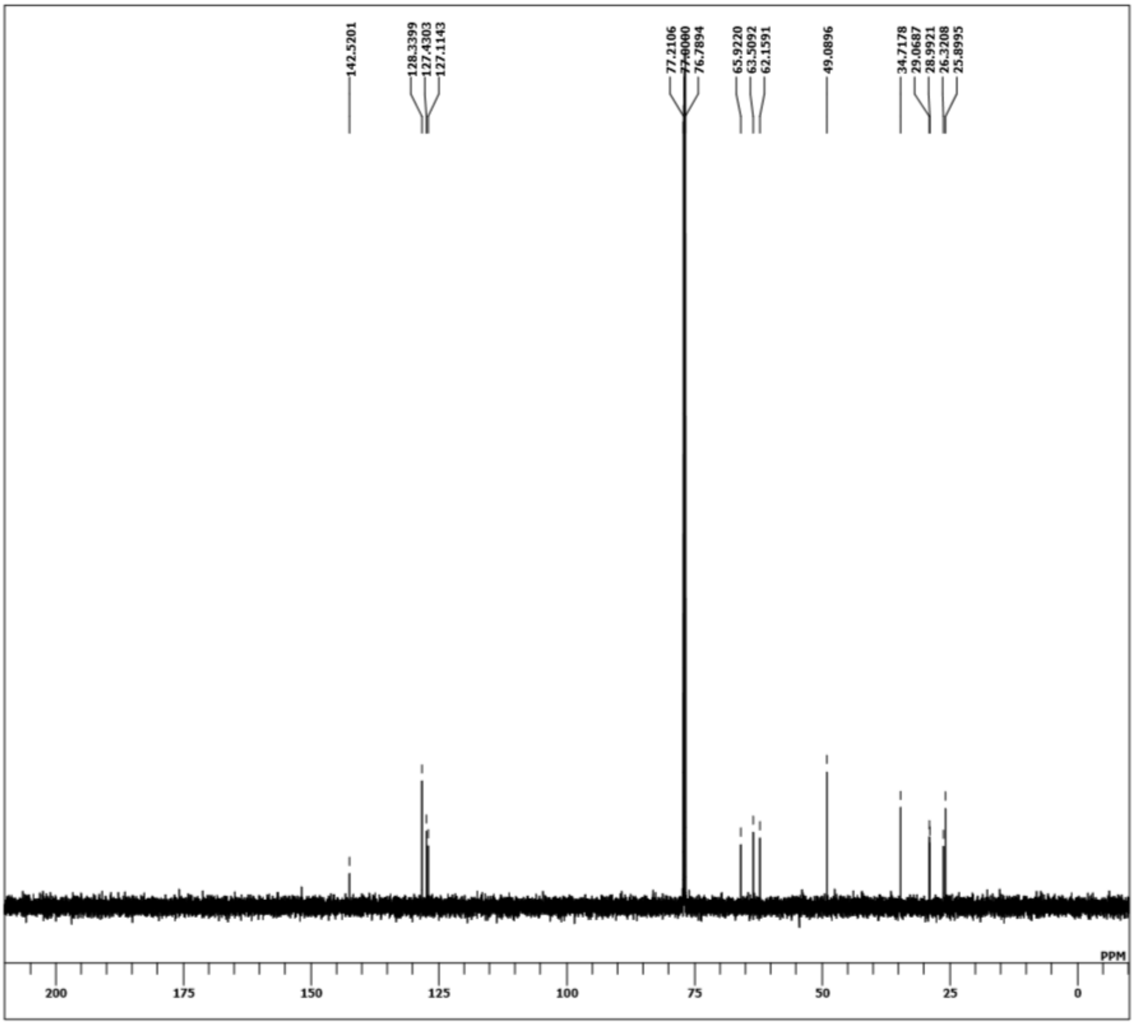

Supplementary Figure 30 ^{1}H NMR of **L21**

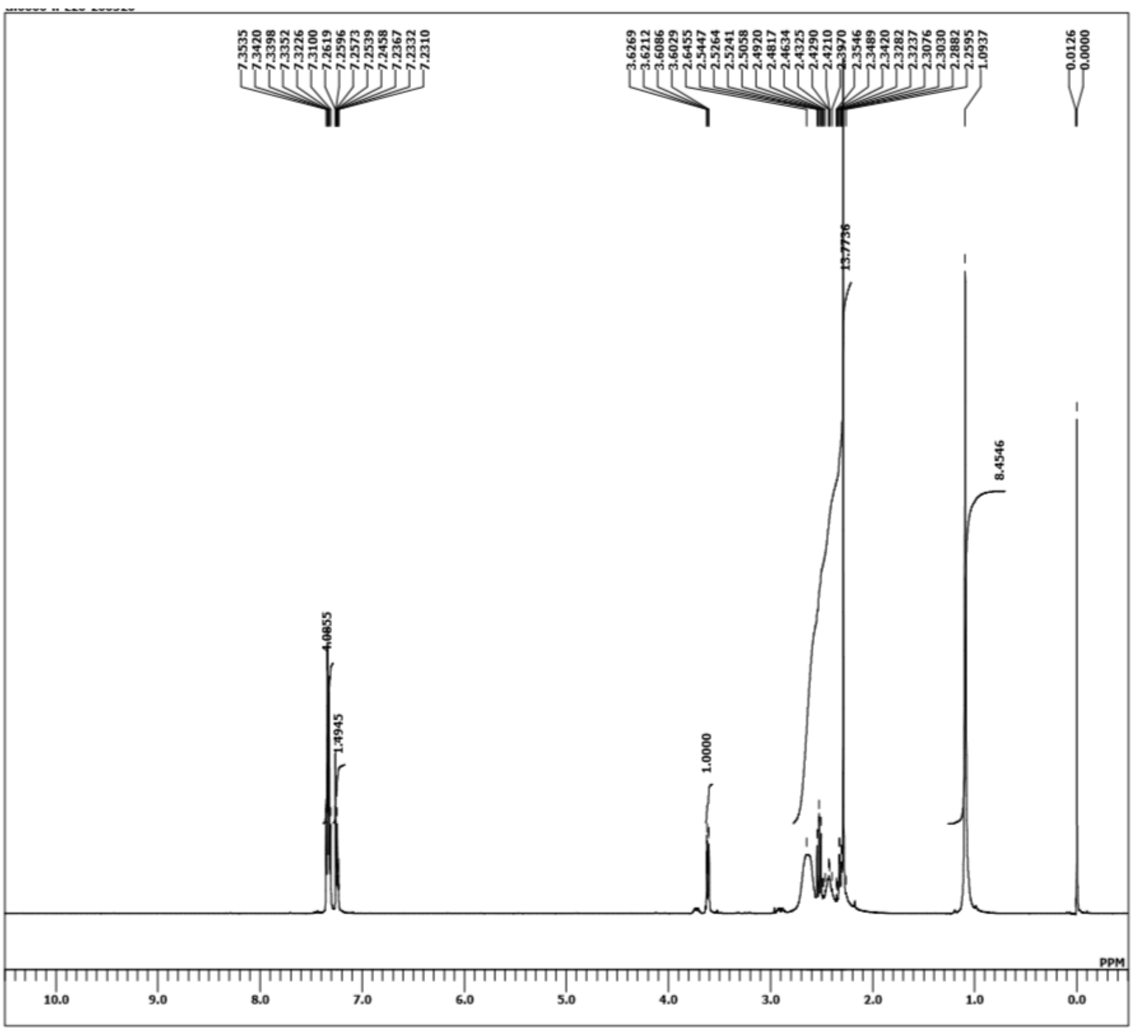

Supplementary Figure 31 ^{13}C NMR of **L21**

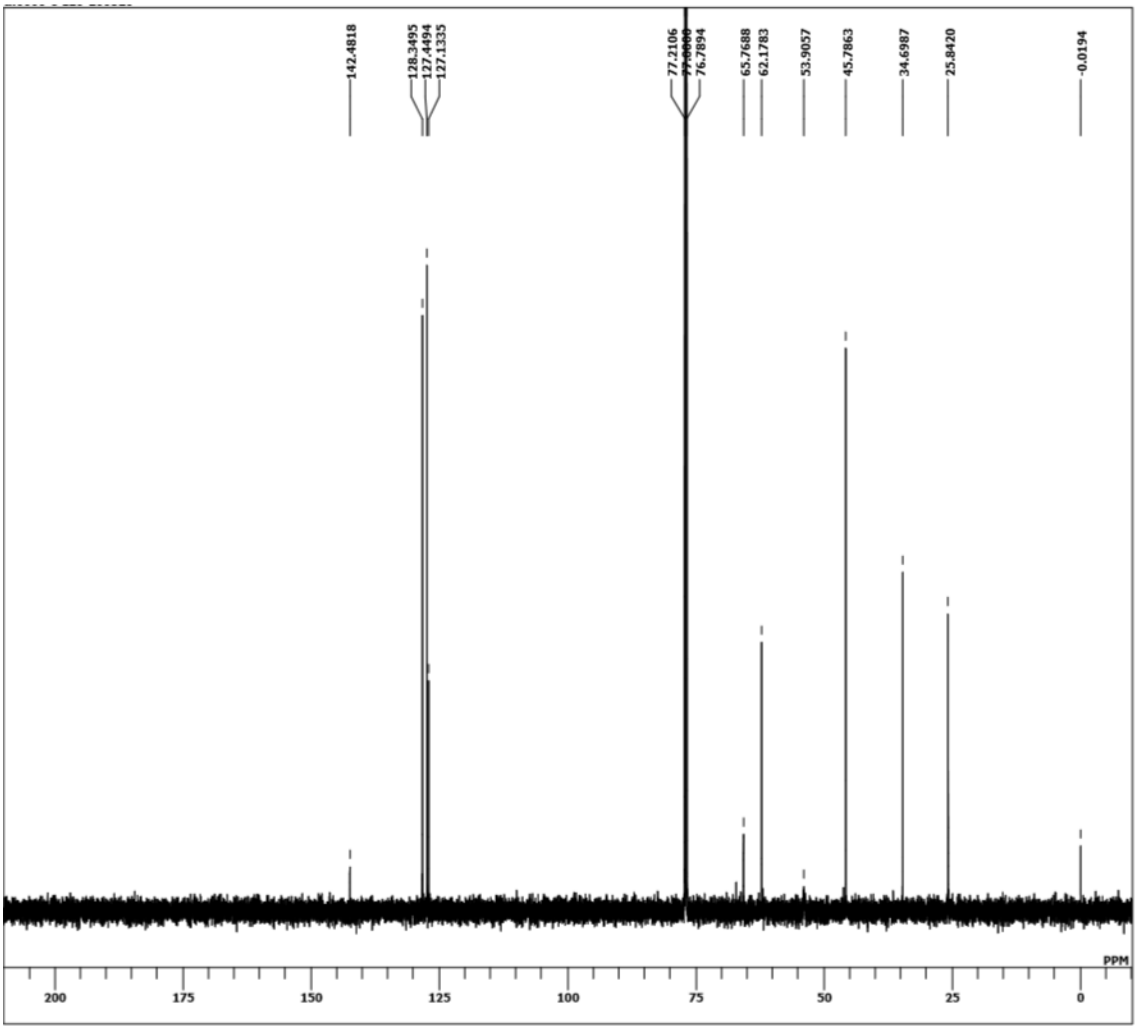

Supplementary Figure 32 ^{1}H NMR of **L22**

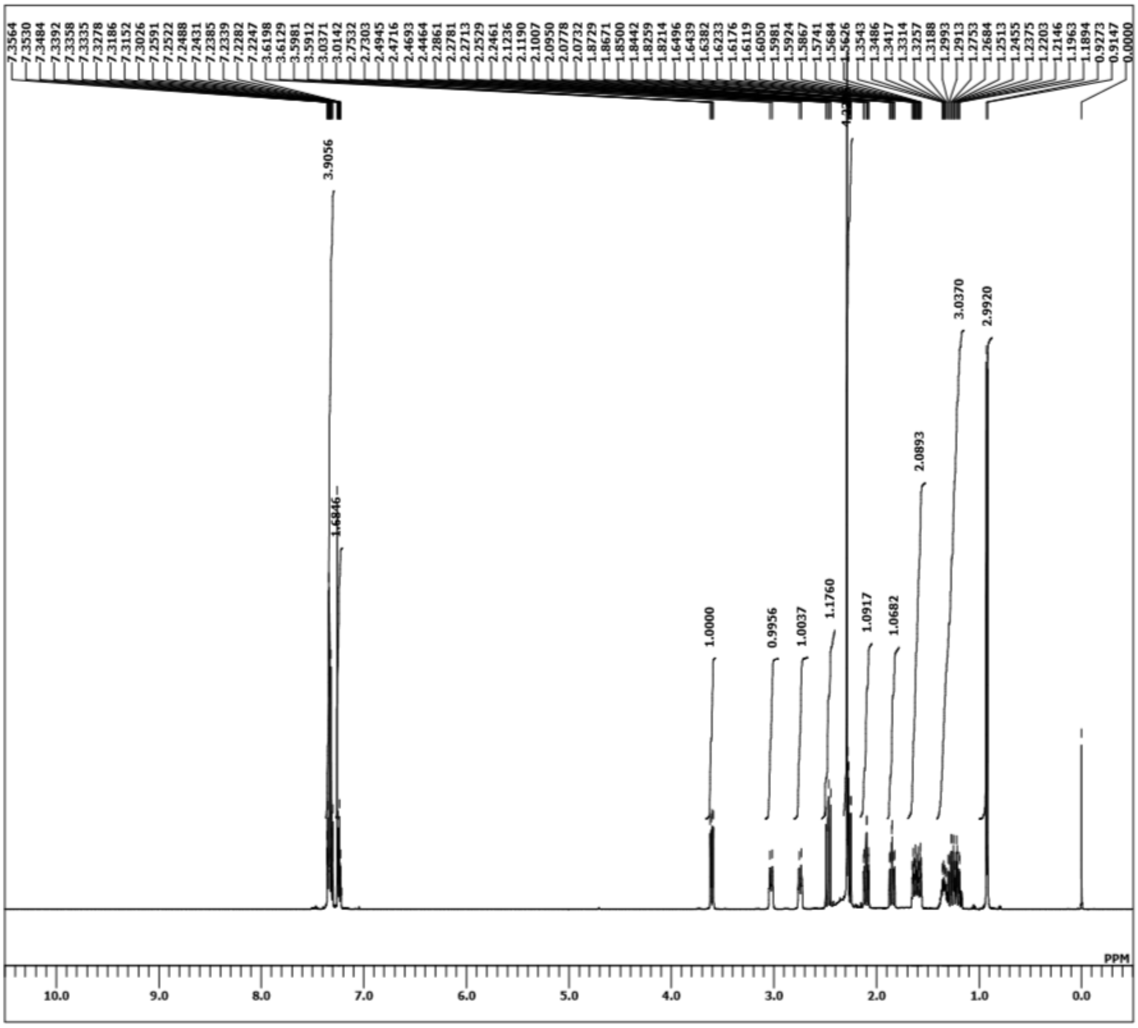

Supplementary Figure 33 ^{13}C NMR of **L22**

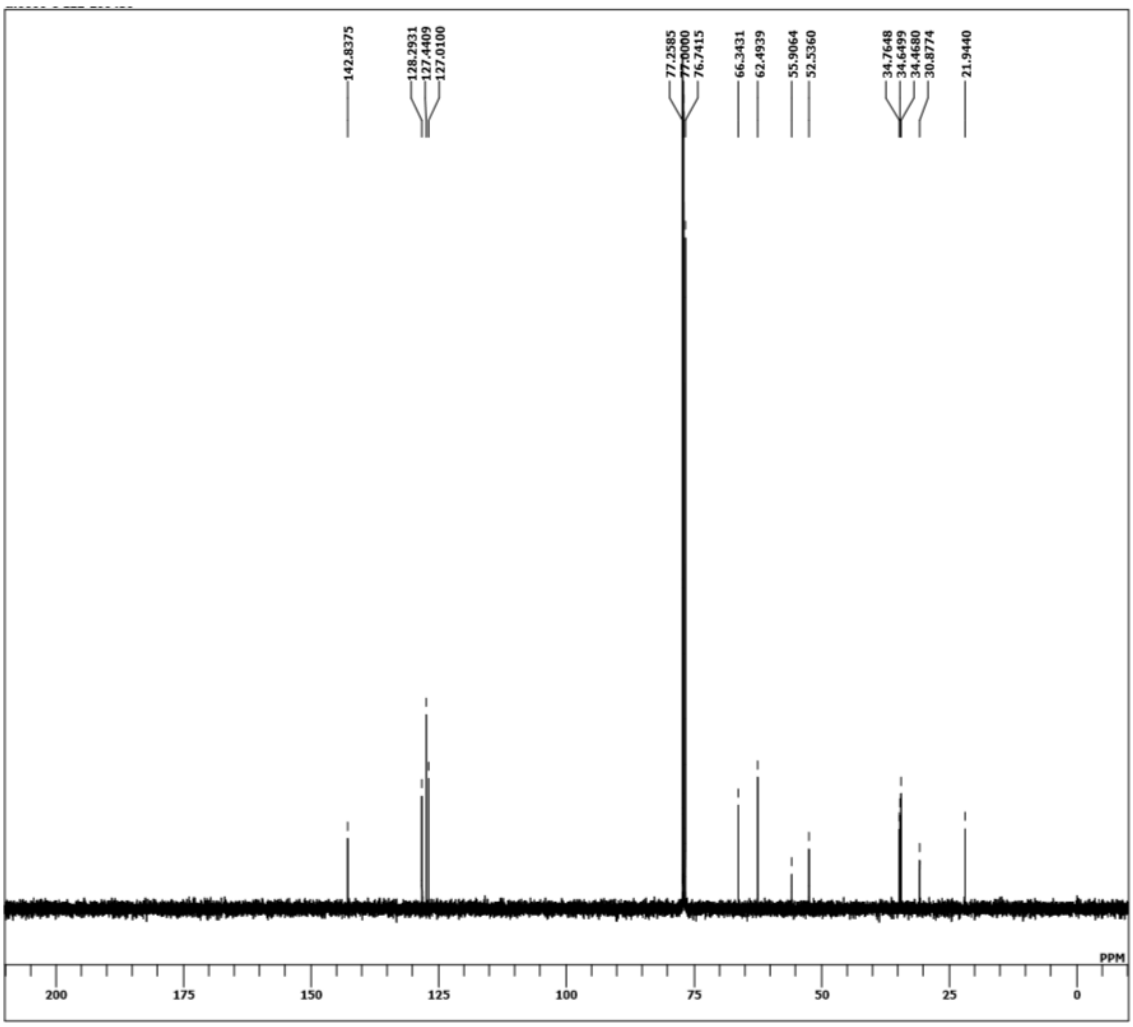

Supplementary Figure 34 ^{1}H NMR of **3aa**

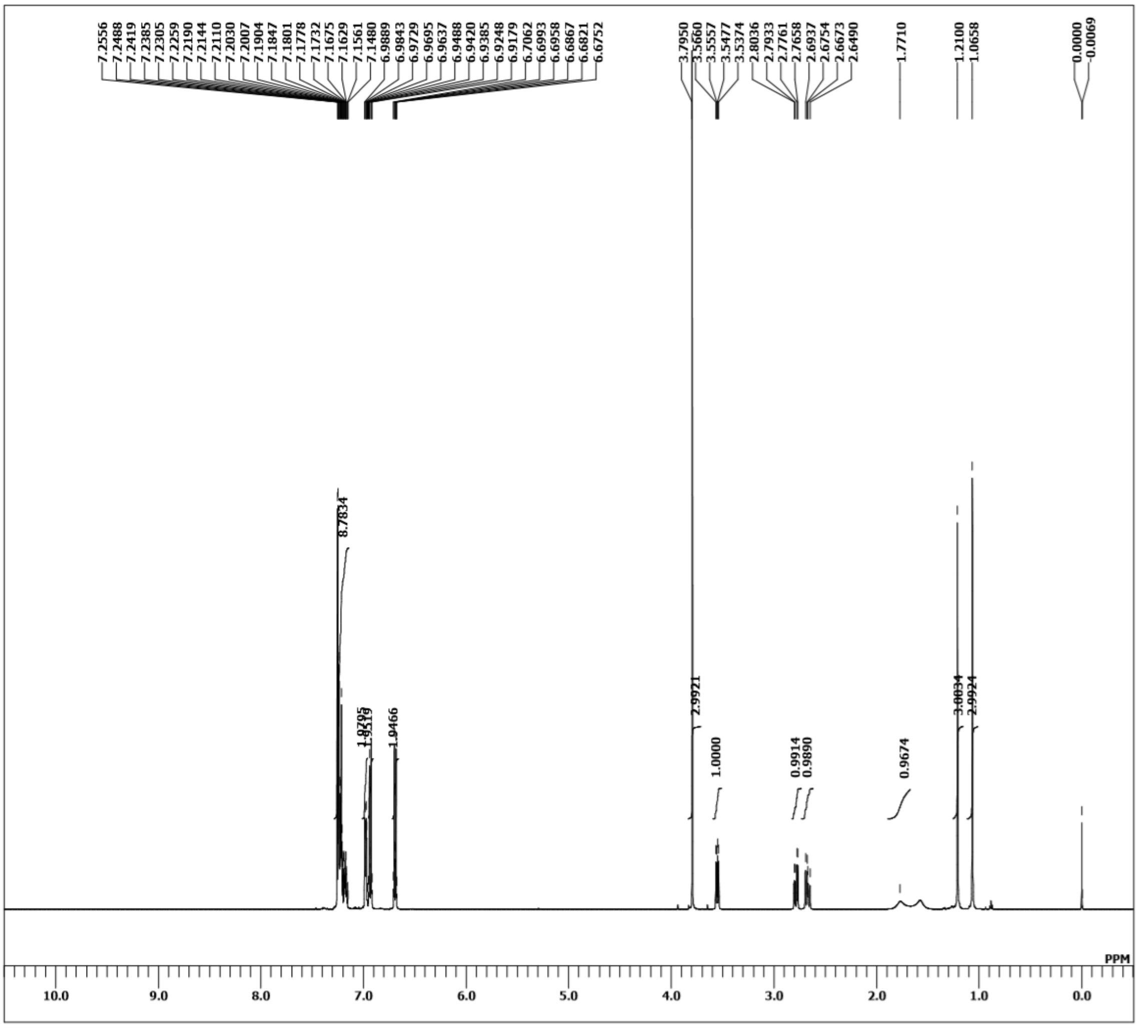

Supplementary Figure 35 ^{13}C NMR of **3aa**

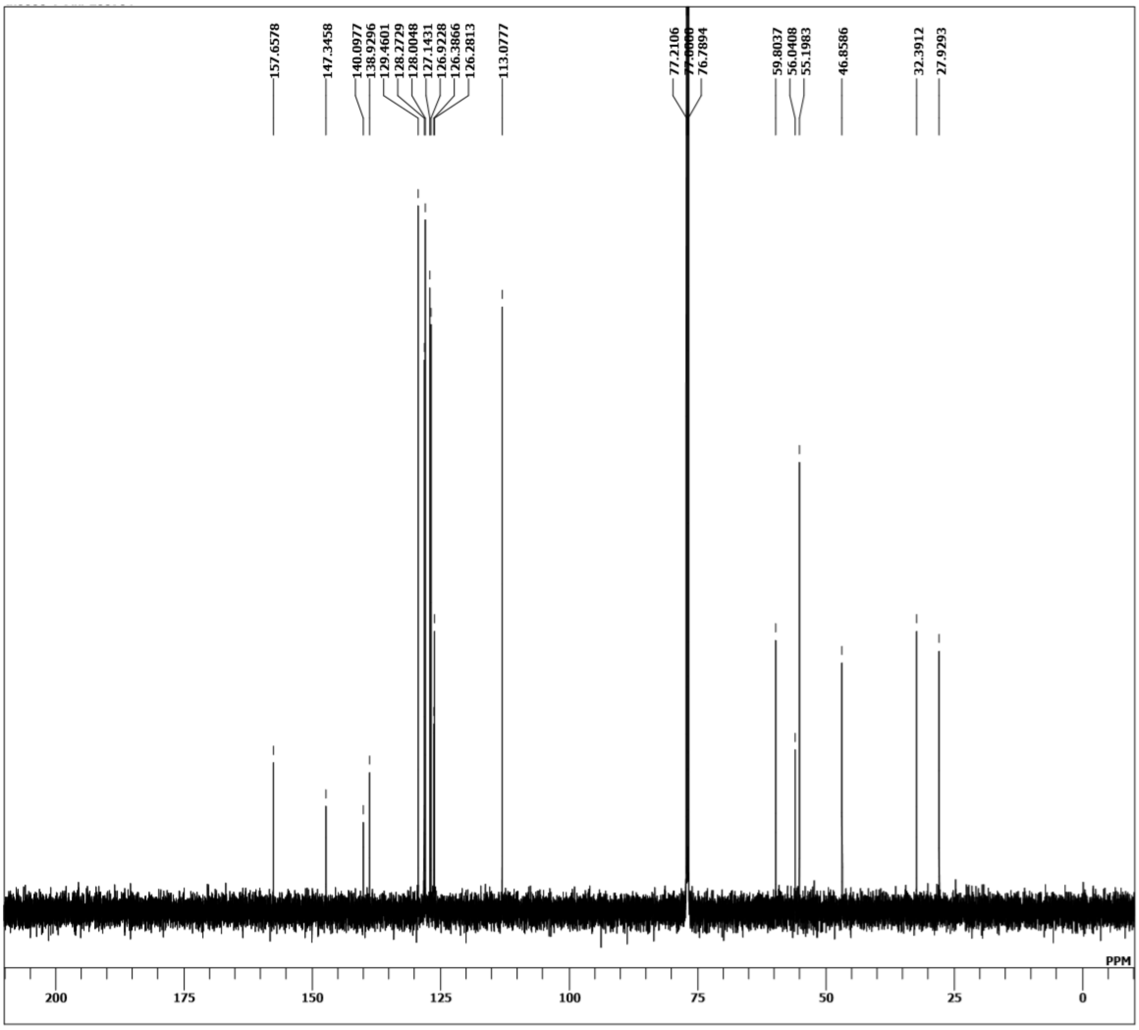

Supplementary Figure 36 HPLC analysis of **3aa**

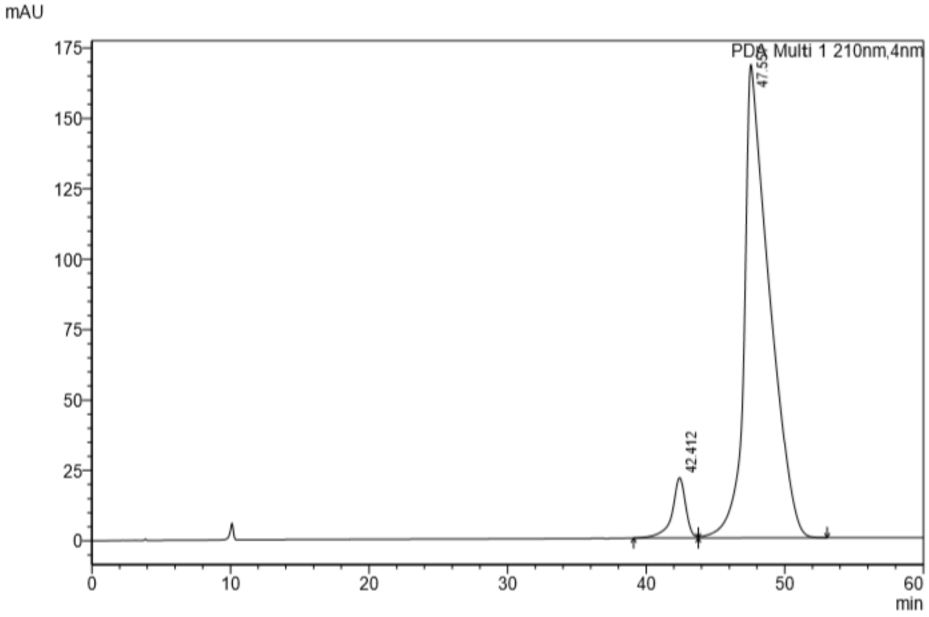

Peak Table

PDA Ch1 210nm

Peak#	Ret. Time	Area	Height	Area%
1	42.412	1458317	21365	6.422
2	47.557	21249731	167995	93.578
‡Œv		22708048	189360	100.000

Supplementary Figure 37 HPLC analysis of **3aa** (after recrystallization)

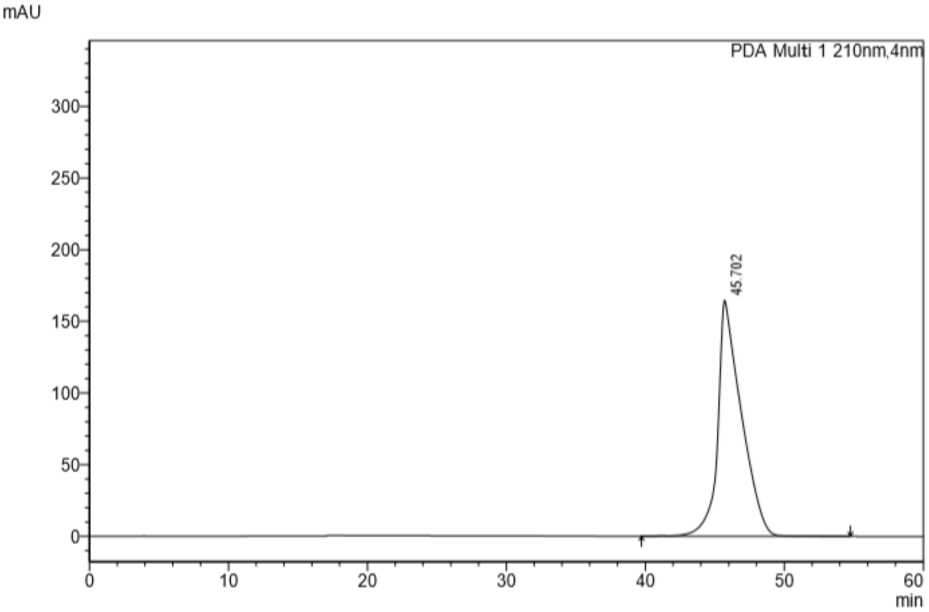

Peak Table

PDA Ch1 210nm

Peak#	Ret. Time	Area	Height	Area%
1	45.702	19478481	164534	100.000
‡Œv		19478481	164534	100.000

Supplementary Figure 38 HPLC analysis of **3aa** (racemic)

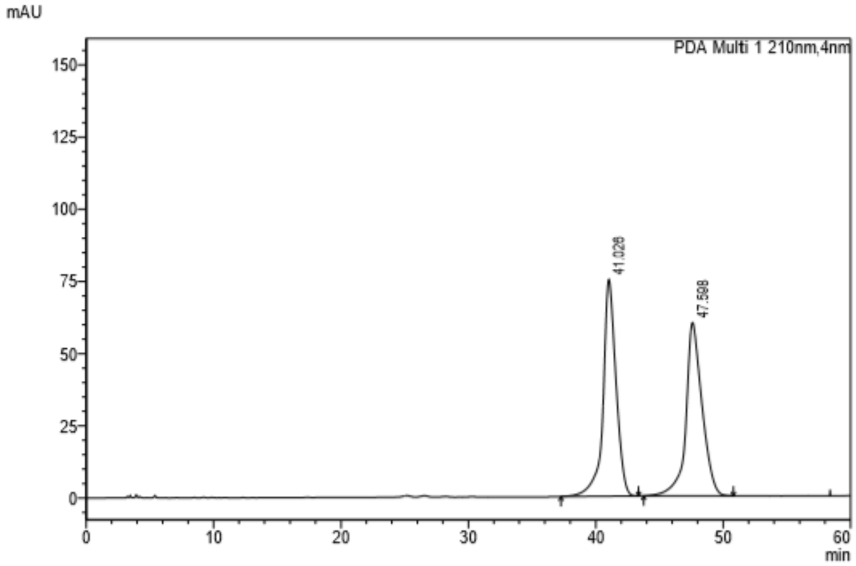

Peak Table

PDA Ch1 210nm

Peak#	Ret. Time	Area	Height	Area%
1	41.026	5329856	75148	50.110
2	47.598	5306558	60089	49.890
Total		10636414	135238	100.000

Supplementary Figure 39 ^{1}H NMR of **3ba**

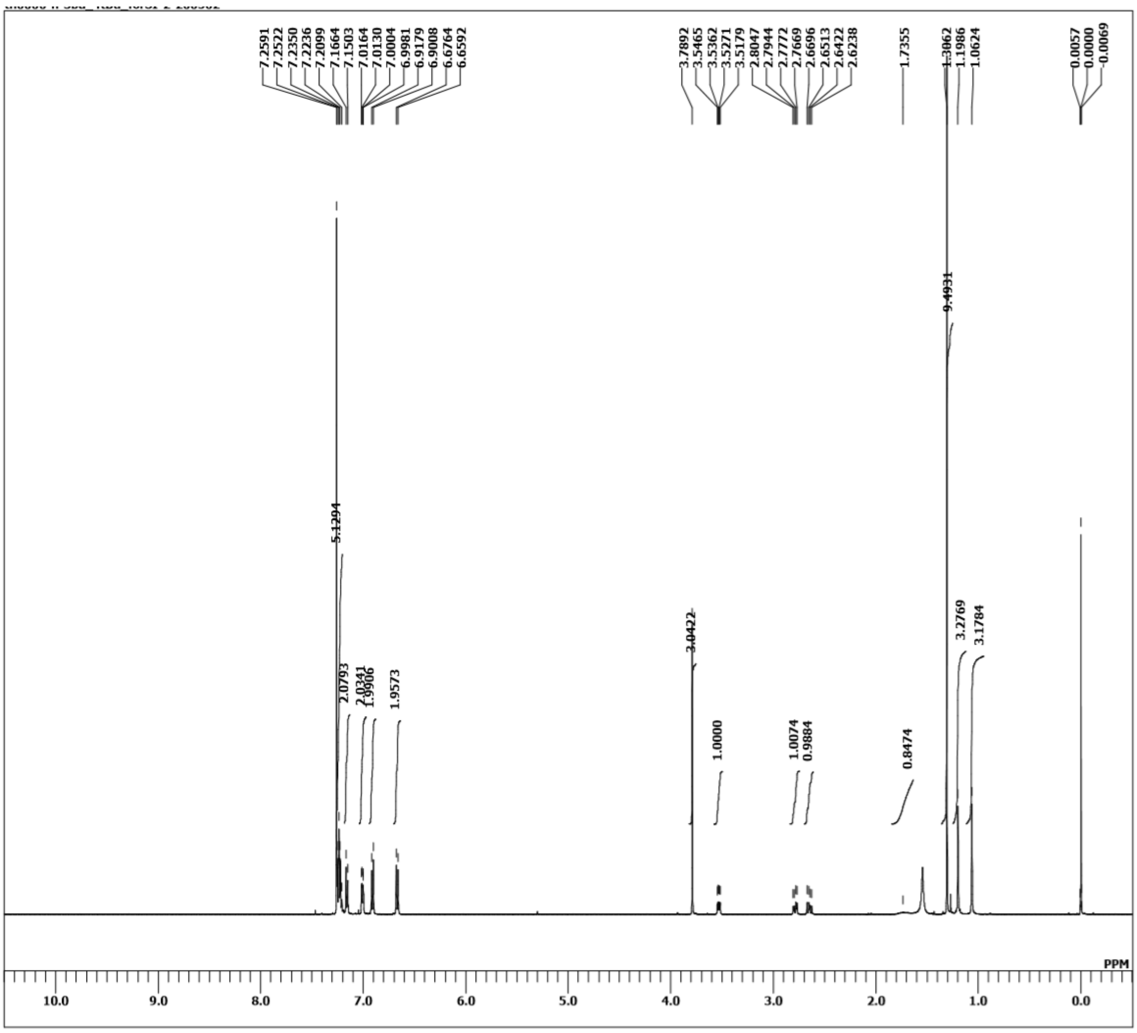

Supplementary Figure 40 ^{13}C NMR of **3ba**

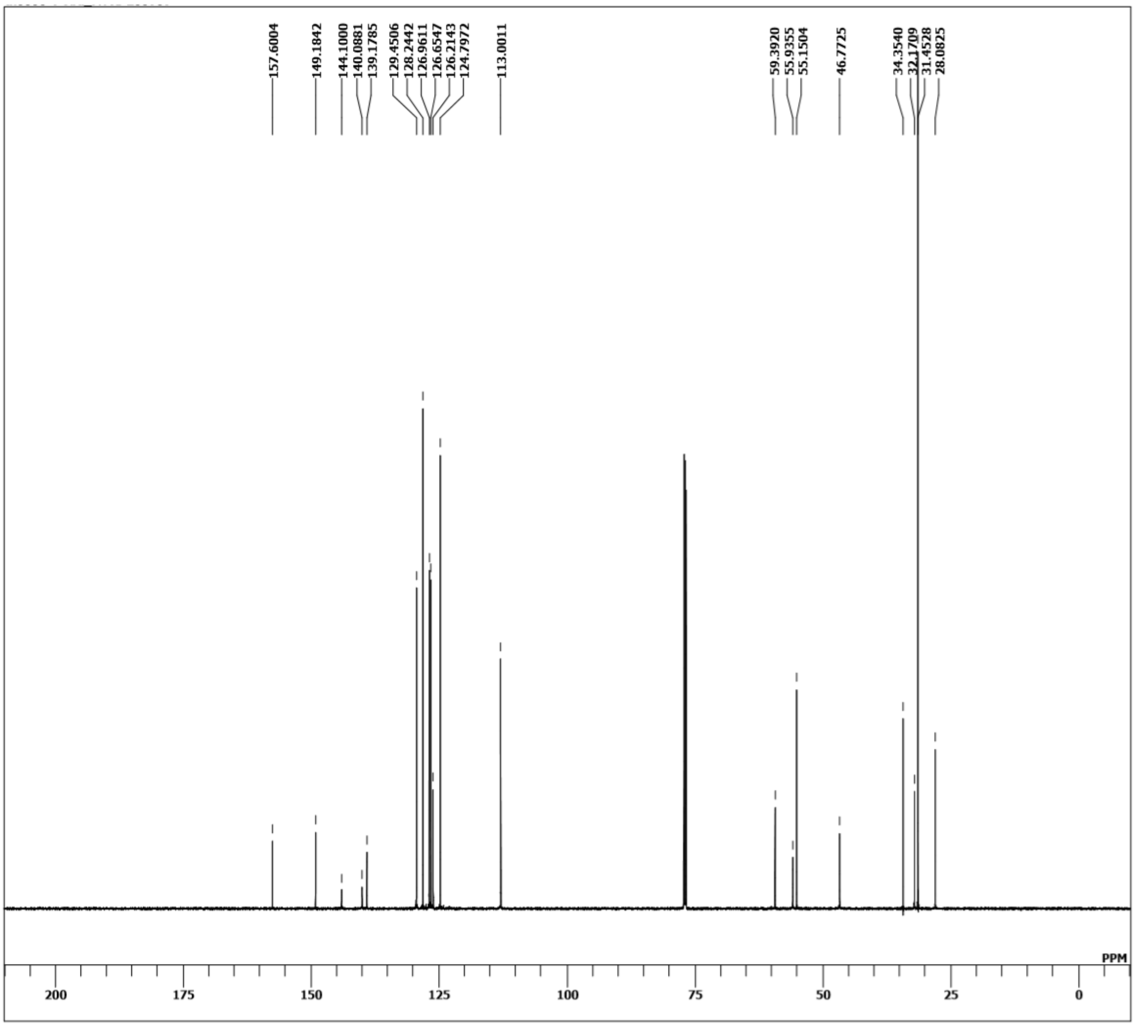

Supplementary Figure 41 HPLC analysis of **3ba**

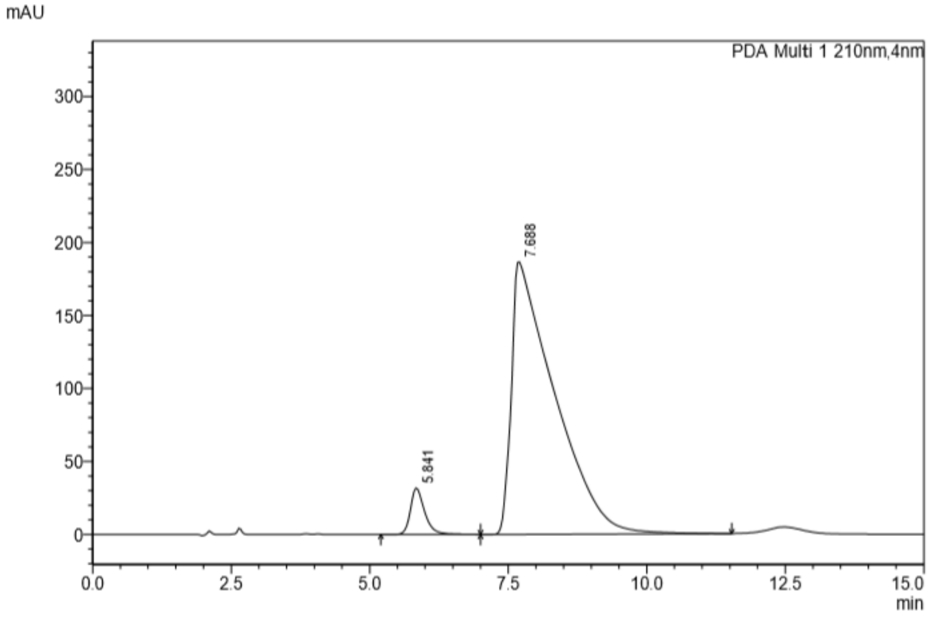

Peak Table

PDA Ch1 210nm

Peak#	Ret. Time	Area	Height	Area%
1	5.841	571956	31860	5.346
2	7.688	10127002	186563	94.654
‡Œv		10698958	218423	100.000

Supplementary Figure 42 HPLC analysis of **3ba** (racemic)

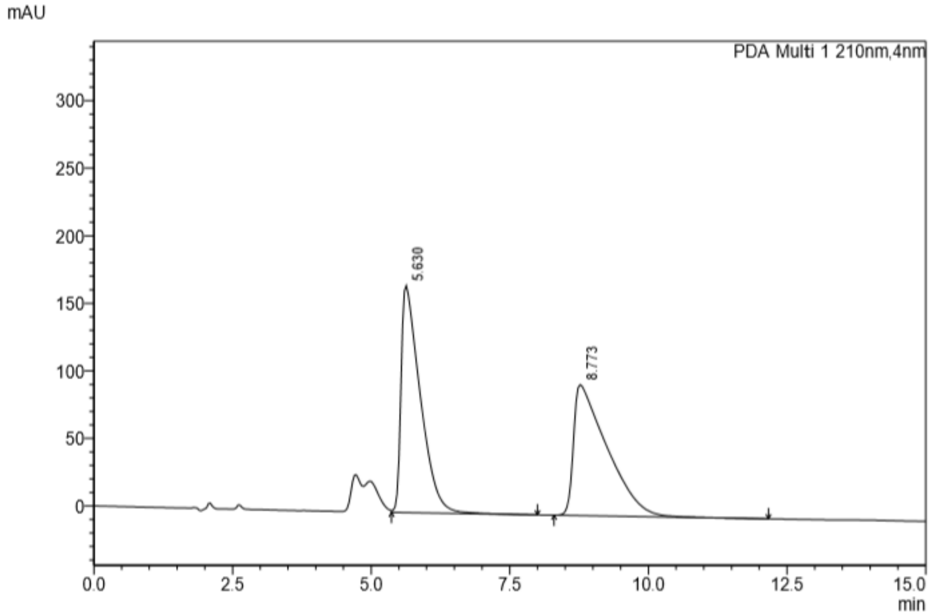

Peak Table

PDA Ch1 210nm

Peak#	Ret. Time	Area	Height	Area%
1	5.630	4068272	168050	50.022
2	8.773	4064733	96864	49.978
‡Œv		8133005	264914	100.000

Supplementary Figure 43 ^{1}H NMR of **3ca**

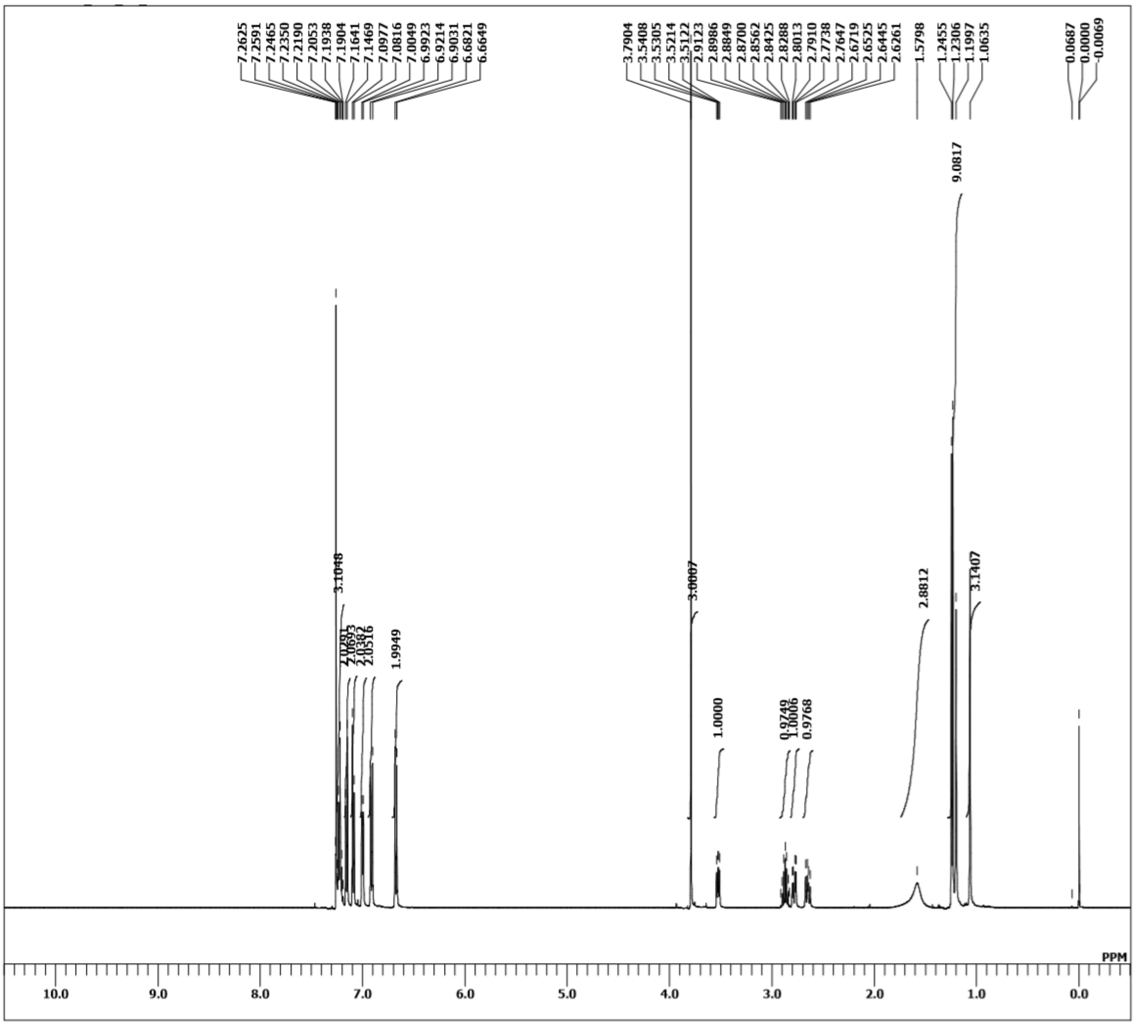

Supplementary Figure 44 ^{13}C NMR of **3ca**

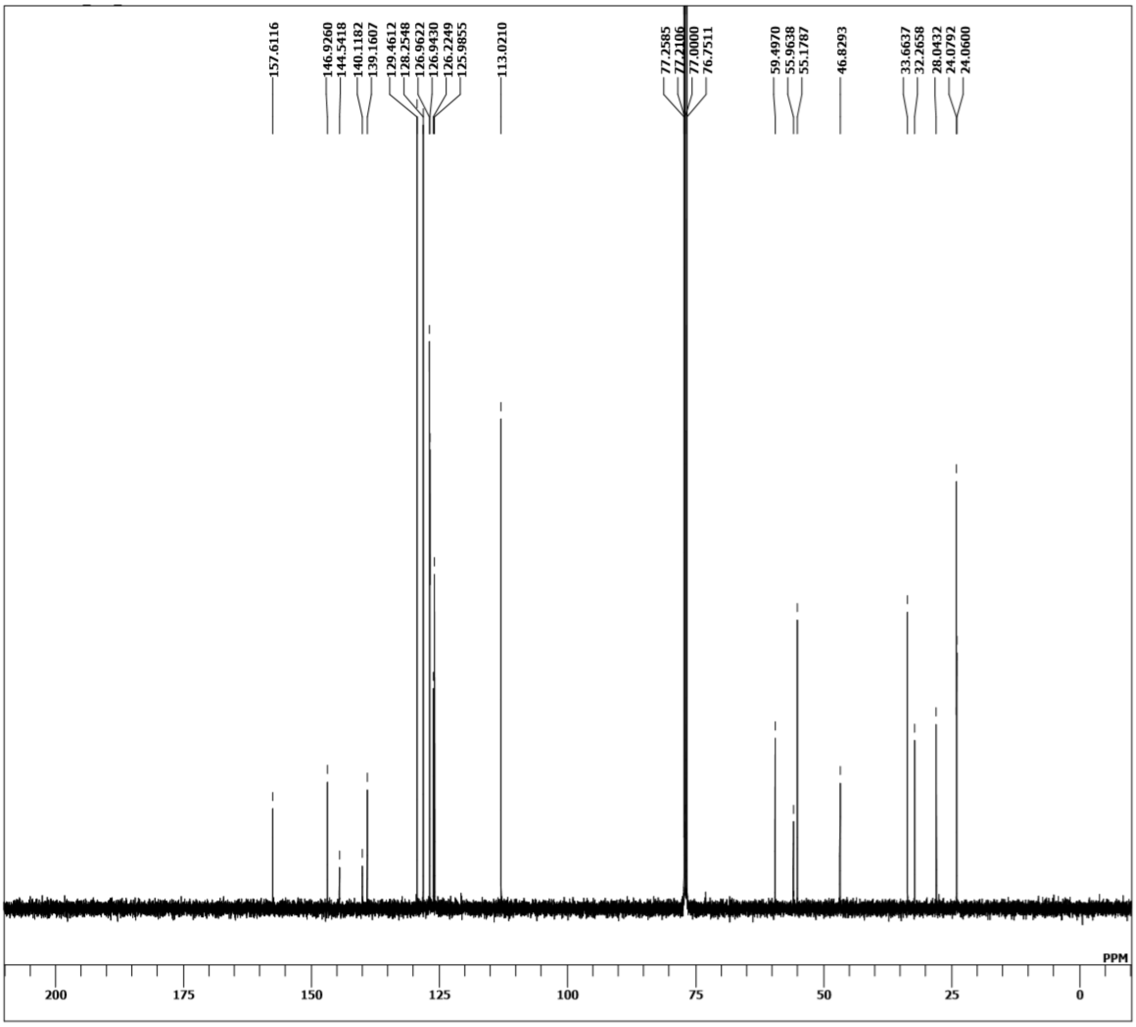

Supplementary Figure 45 ^{1}H NMR of **3da**

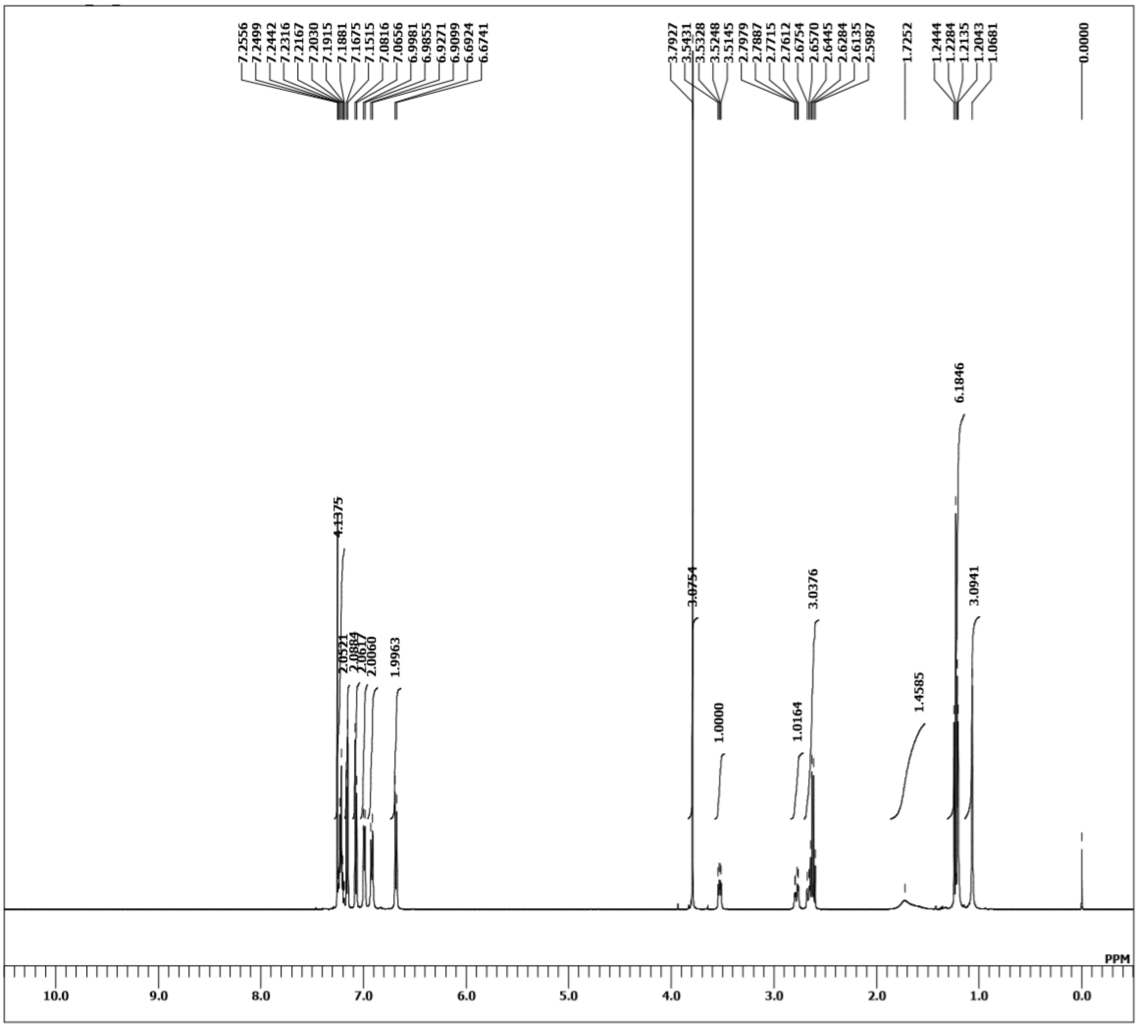

Supplementary Figure 46 ^{13}C NMR of **3da**

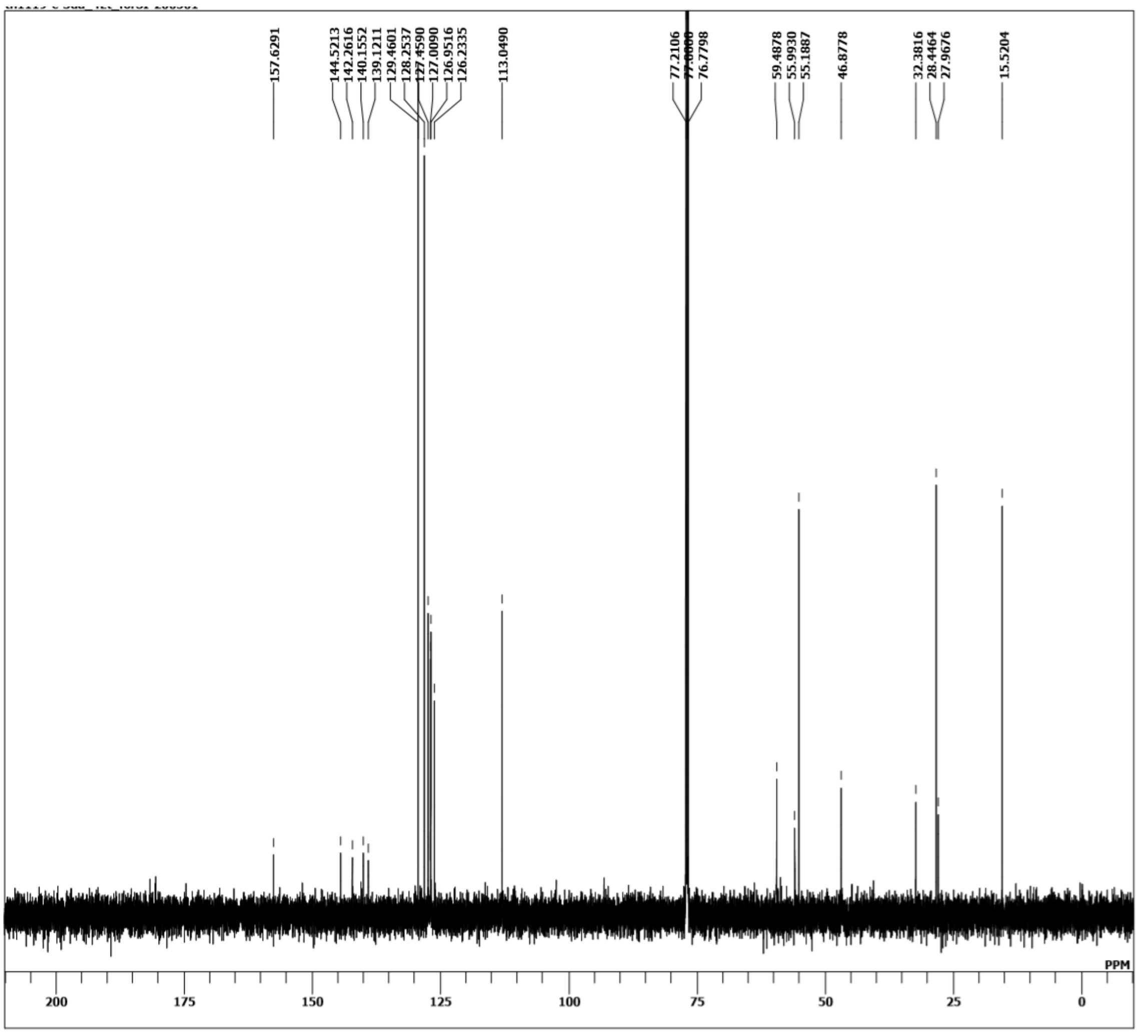

Supplementary Figure 47 ^{1}H NMR of **3ea**

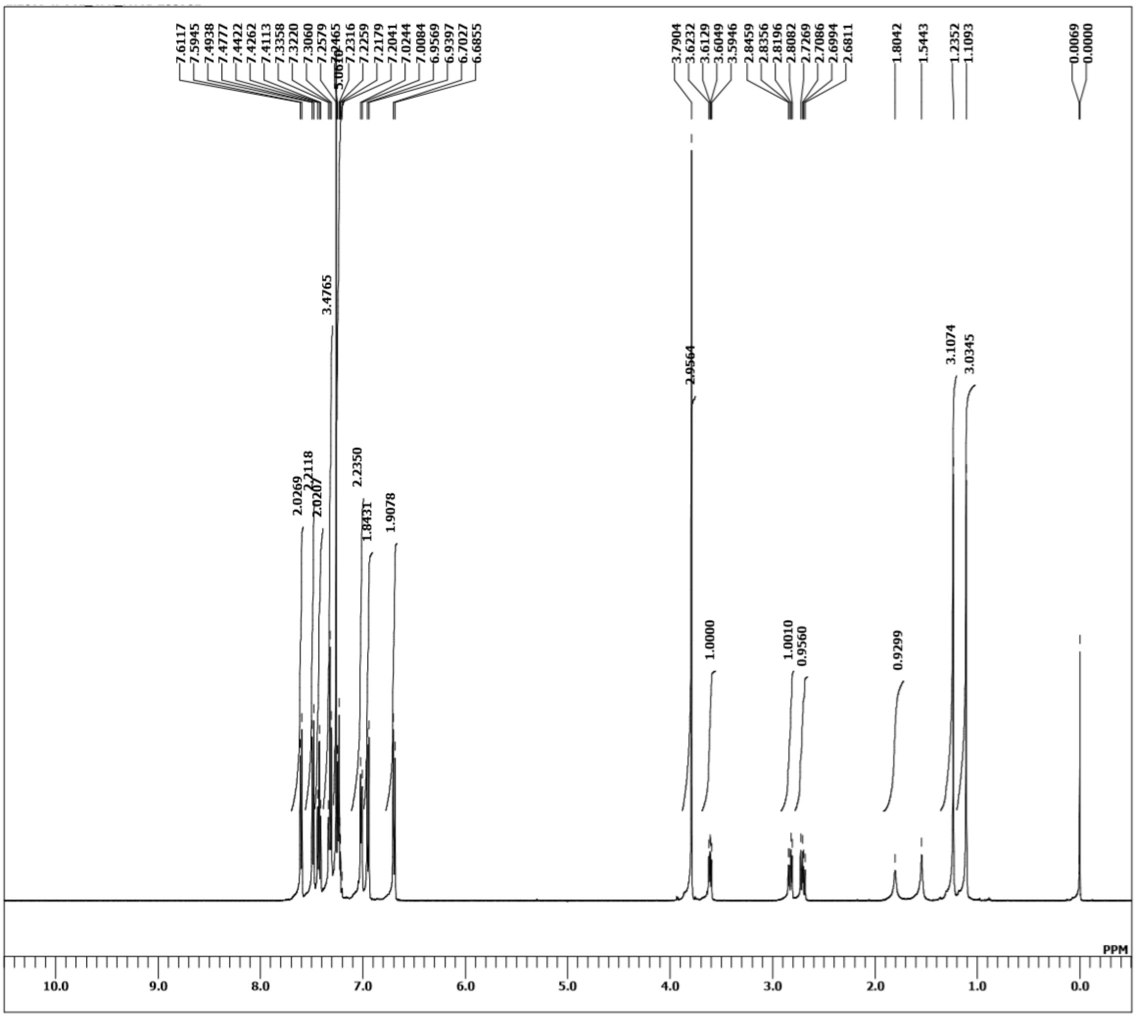

Supplementary Figure 48 ^{13}C NMR of **3ea**

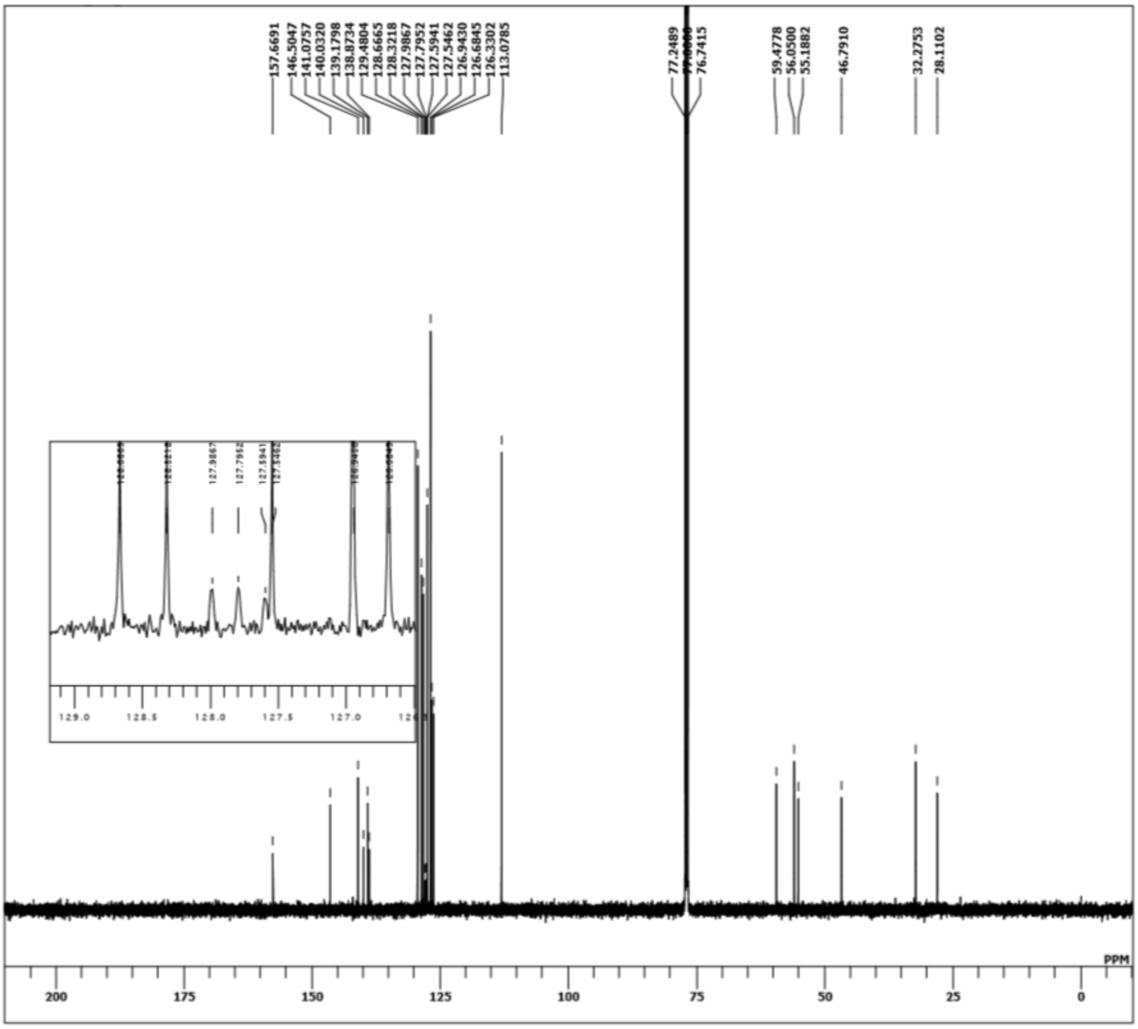

Supplementary Figure 49 HPLC analysis of **3ea**

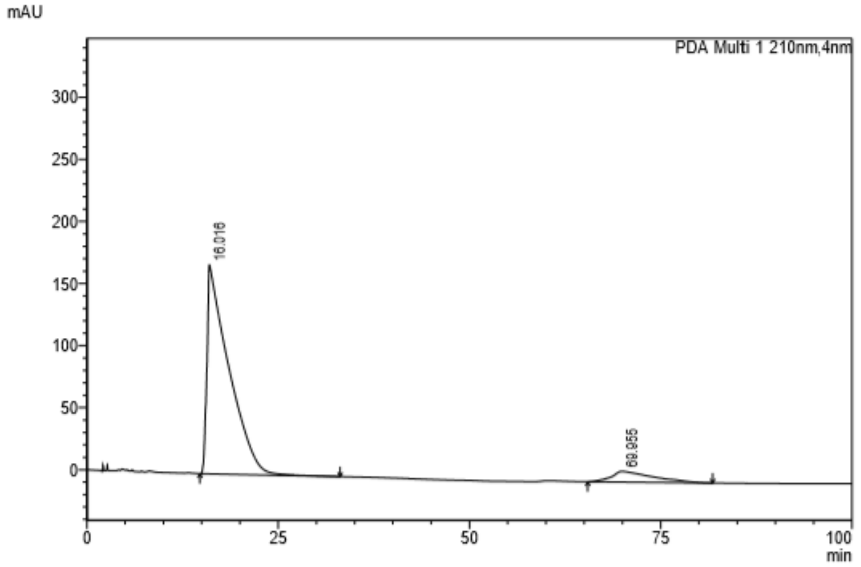

Peak Table

PDA Ch1 210nm

Peak#	Ret. Time	Area	Height	Area%
1	16.016	33427896	168177	91.182
2	69.955	3232739	8684	8.818
ΣŒv		36660635	176861	100.000

Supplementary Figure 50 HPLC analysis of **3ea** (after recrystallization)

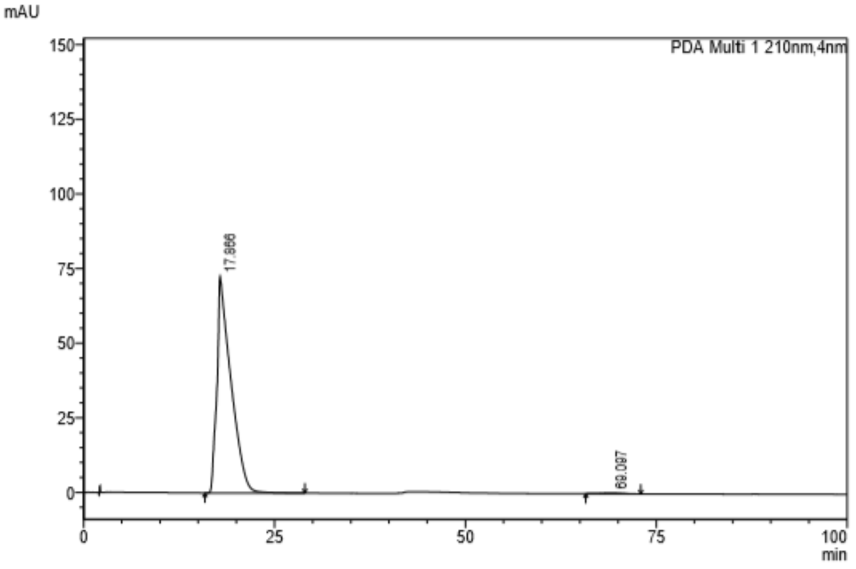

Peak Table

PDA Ch1 210nm

Peak#	Ret. Time	Area	Height	Area%
1	17.866	9279065	72528	99.446
2	69.097	51667	280	0.554
ΣŒv		9330731	72808	100.000

Supplementary Figure 51 HPLC analysis of **3ea** (racemic)

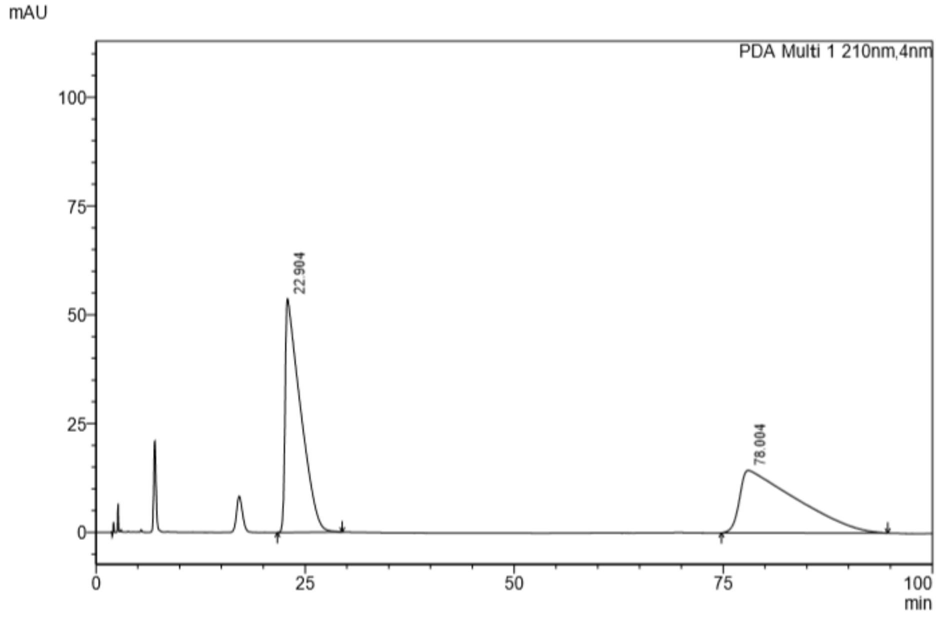

Peak Table

PDA Ch1 210nm

Peak#	Ret. Time	Area	Height	Area%
1	22.904	6851070	53714	50.289
2	78.004	6772318	14364	49.711
‡Œv		13623387	68078	100.000

Supplementary Figure 52 ^{1}H NMR of **3fa**

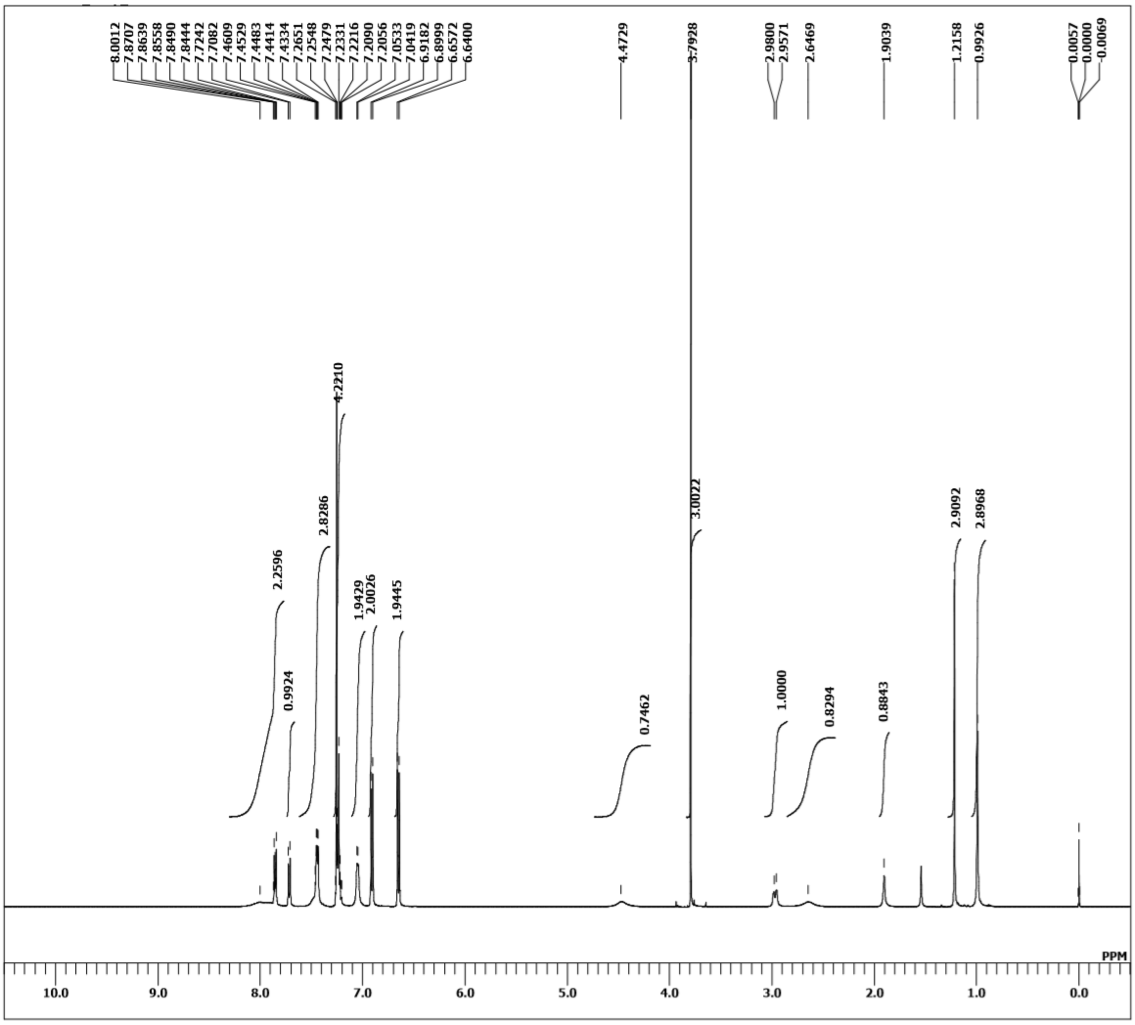

Supplementary Figure 53 ^{13}C NMR of **3fa**

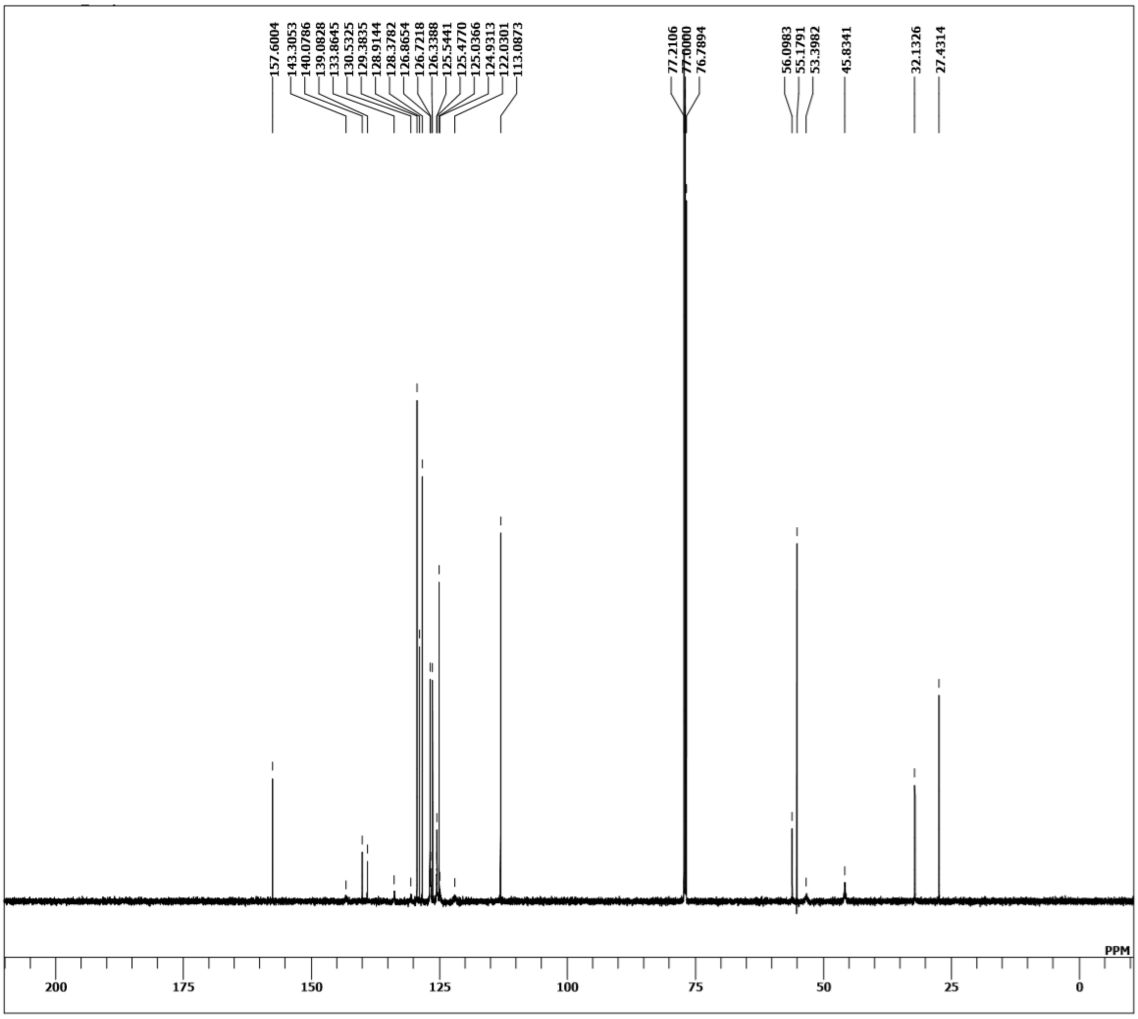

Supplementary Figure 54 HPLC analysis of **3fa**

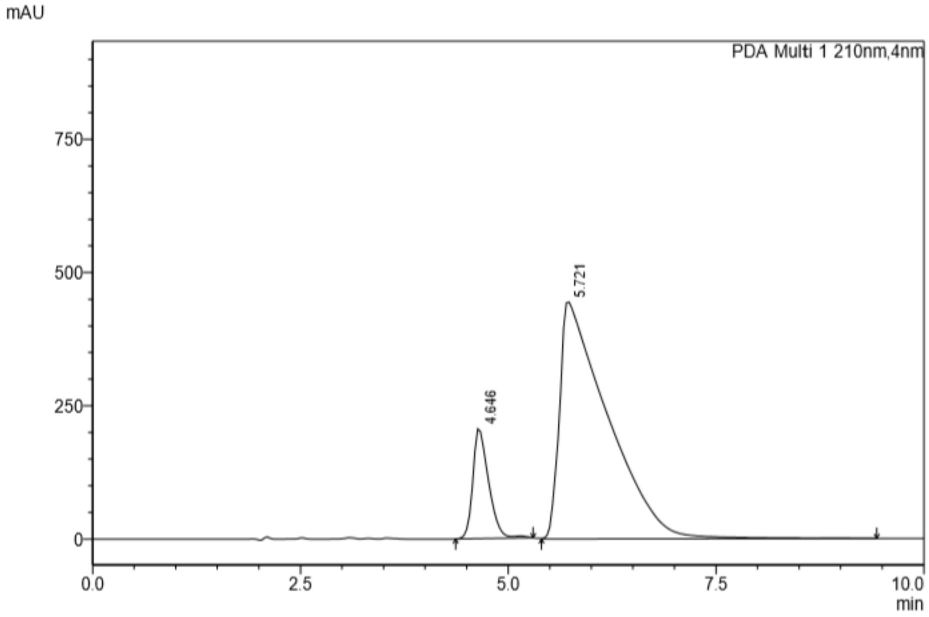

Peak Table

PDA Ch1 210nm

Peak#	Ret. Time	Area	Height	Area%
1	4.646	2746380	206019	13.309
2	5.721	17889339	444239	86.691
‡Œv		20635719	650258	100.000

Supplementary Figure 55 HPLC analysis of **3fa** (after recrystallization)

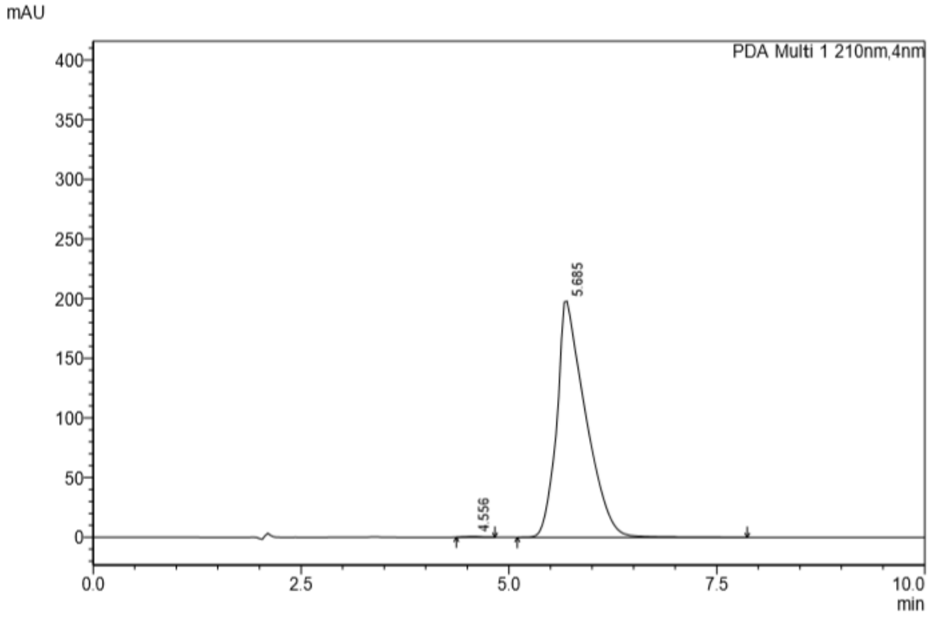

Peak Table

PDA Ch1 210nm

Peak#	Ret. Time	Area	Height	Area%
1	4.556	7259	676	0.152
2	5.685	4776451	198009	99.848
‡Œv		4783709	198685	100.000

Supplementary Figure 56 HPLC analysis of **3fa** (racemic)

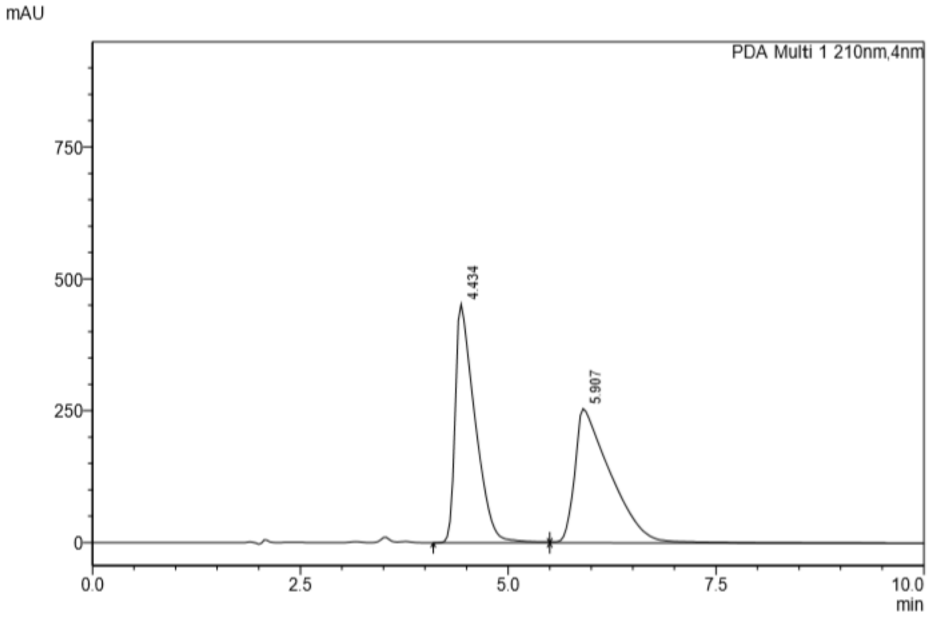

Peak Table

PDA Ch1 210nm

Peak#	Ret. Time	Area	Height	Area%
1	4.434	7533460	452232	49.624
2	5.907	7647585	254771	50.376
‡Œv		15181045	707003	100.000

Supplementary Figure 57 ^{1}H NMR of **3ga**

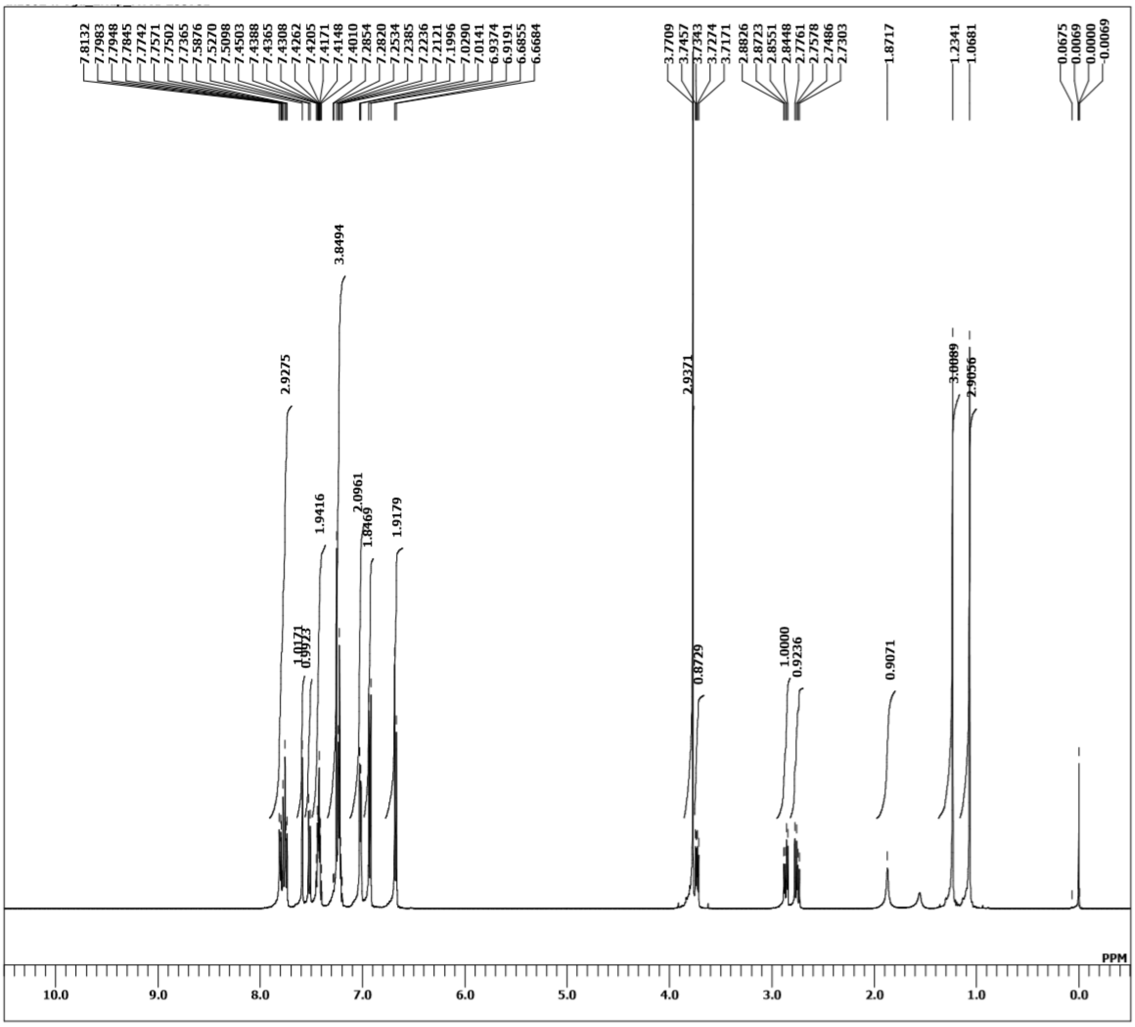

Supplementary Figure 58 ^{13}C NMR of **3ga**

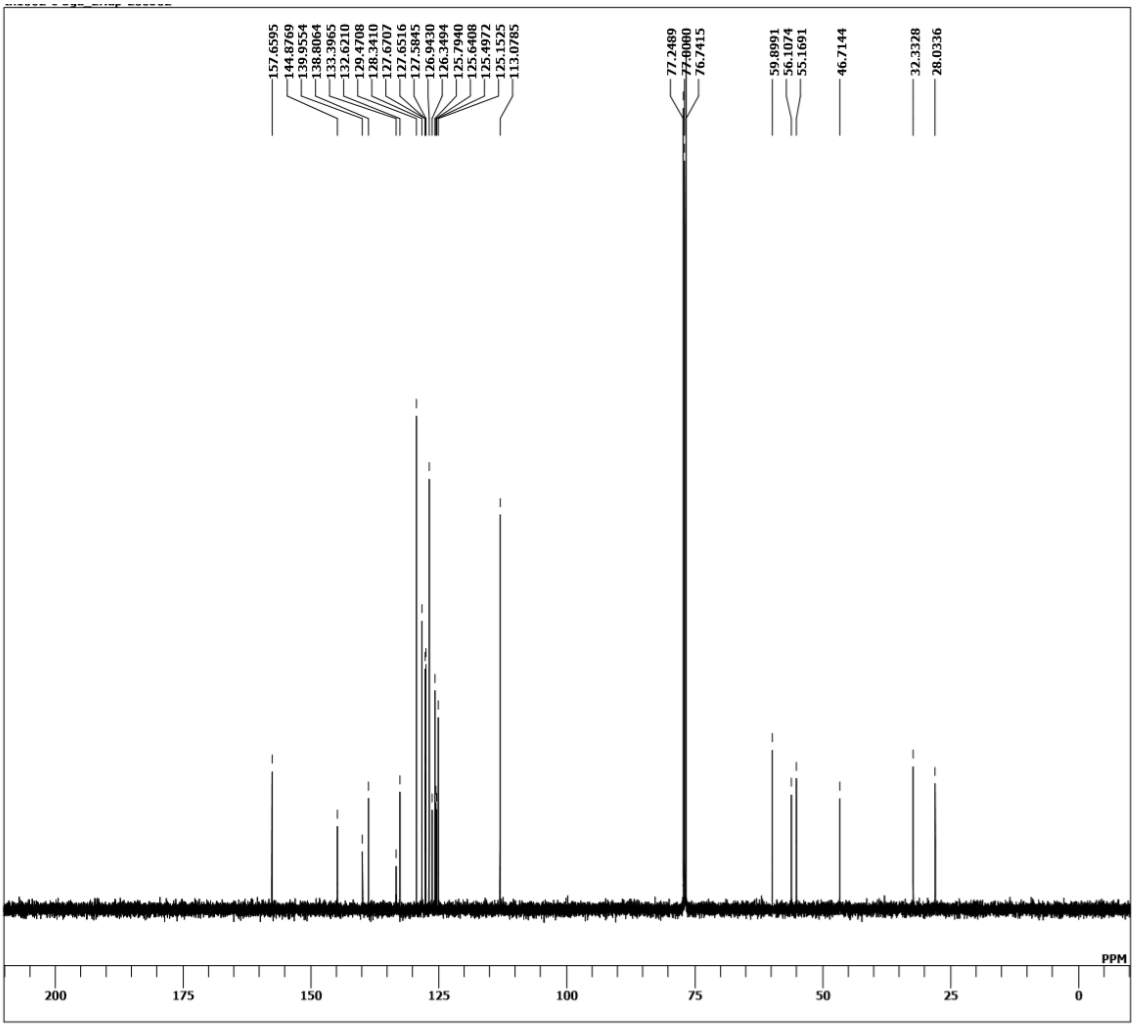

Supplementary Figure 59 HPLC analysis of **3ga**

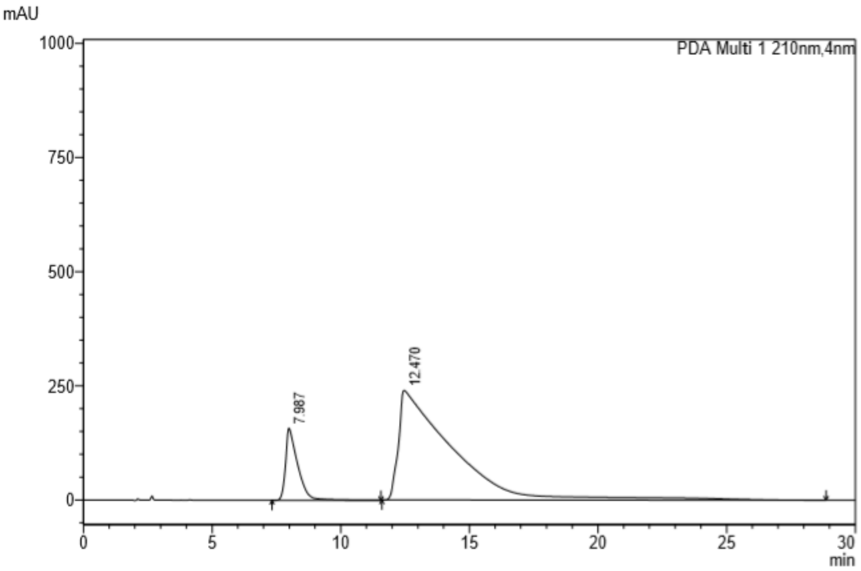

Peak Table

PDA Ch1 210nm

Peak#	Ret. Time	Area	Height	Area%
1	7.987	5287972	157451	13.198
2	12.470	34777848	239782	86.802
±Œv		40065821	397233	100.000

Supplementary Figure 60 HPLC analysis of **3ga** (after recrystallization)

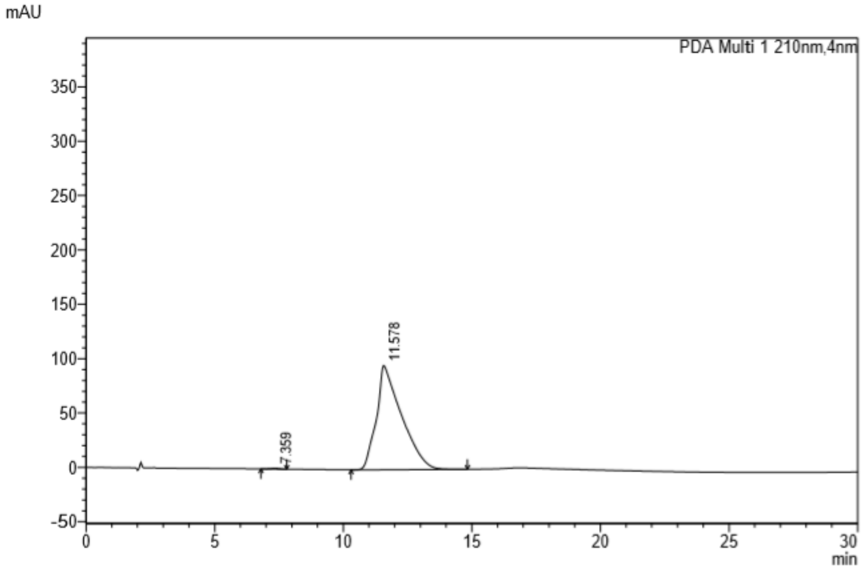

Peak Table

PDA Ch1 210nm

Peak#	Ret. Time	Area	Height	Area%
1	7.359	20221	860	0.309
2	11.578	6513778	95738	99.691
±Œv		6533998	96598	100.000

Supplementary Figure 61 HPLC analysis of **3ga** (racemic)

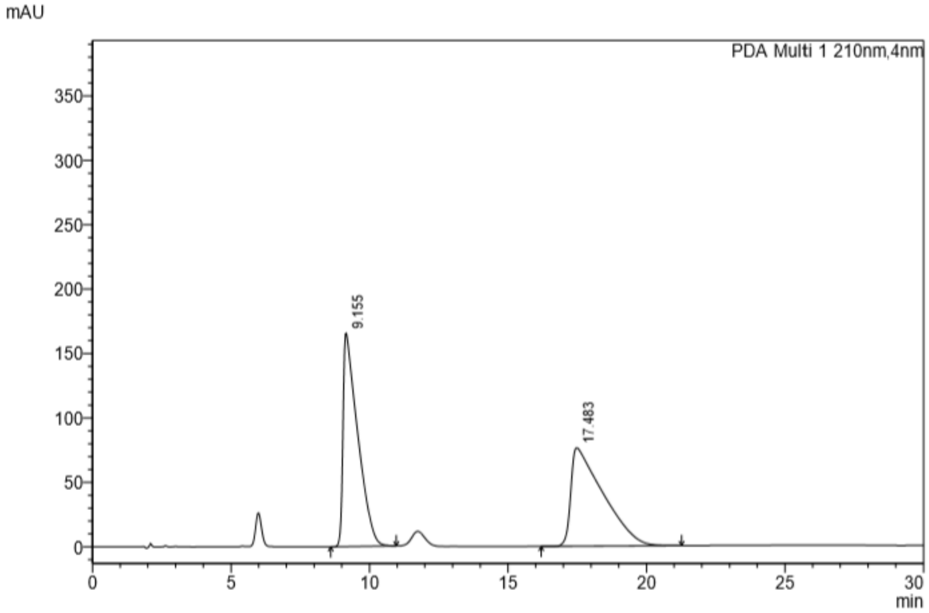

Peak Table

PDA Ch1 210nm

Peak#	Ret. Time	Area	Height	Area%
1	9.155	6110175	165664	49.838
2	17.483	6149967	76267	50.162
‡Œv		12260142	241930	100.000

Supplementary Figure 62 ^{1}H NMR of **3ha**

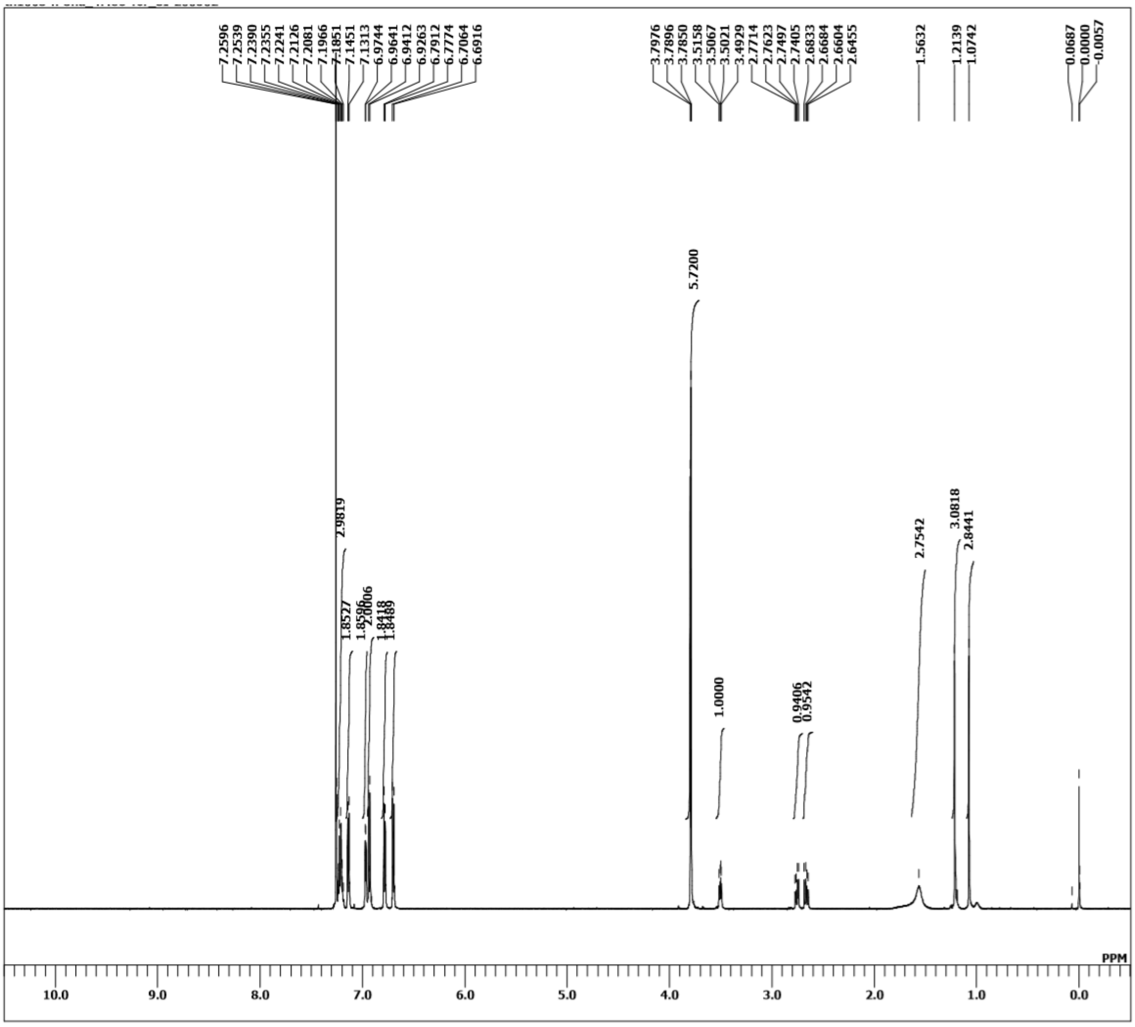

Supplementary Figure 63 ^{13}C NMR of **3ha**

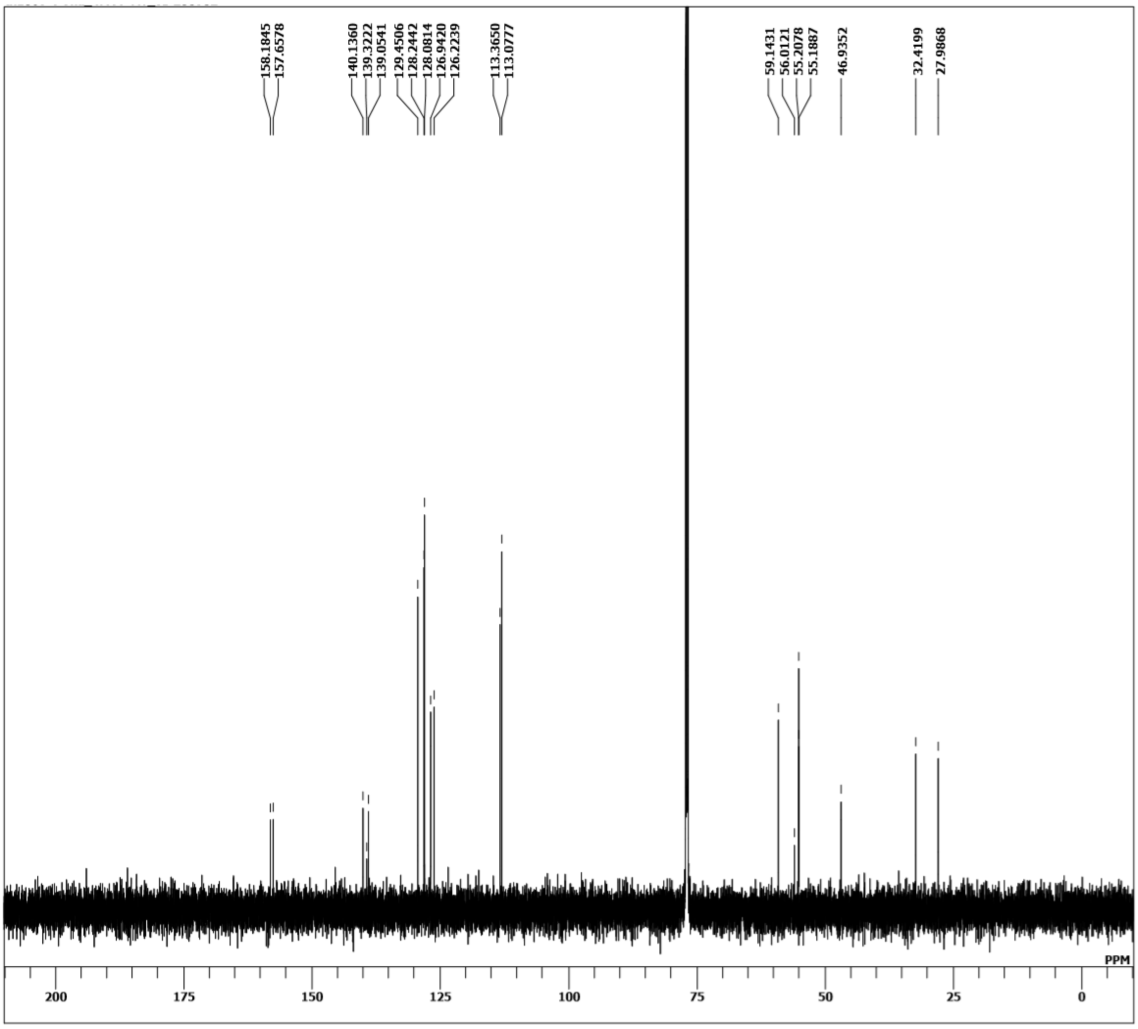

Supplementary Figure 64 ^{1}H NMR of **3ia**

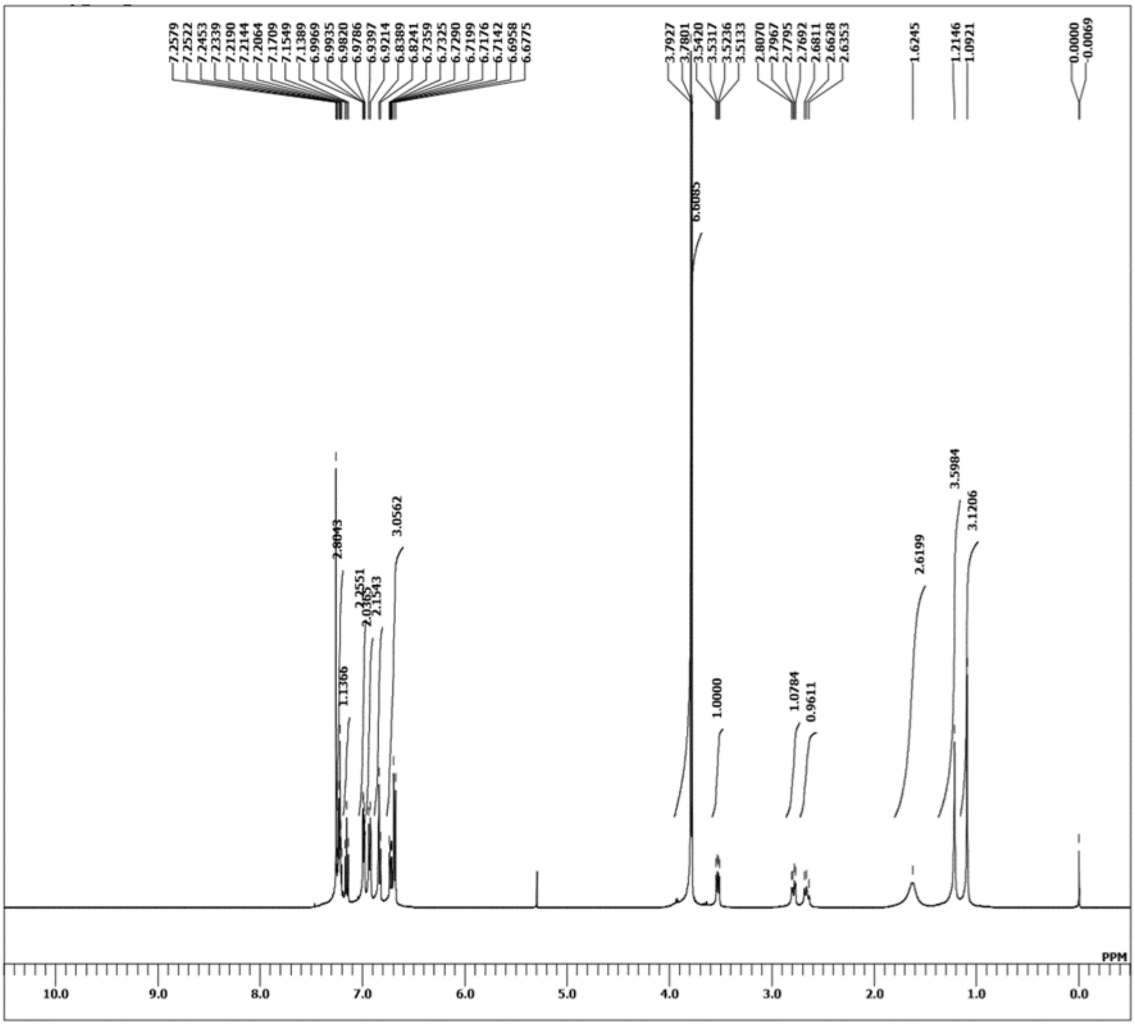

Supplementary Figure 65 ^{13}C NMR of **3ia**

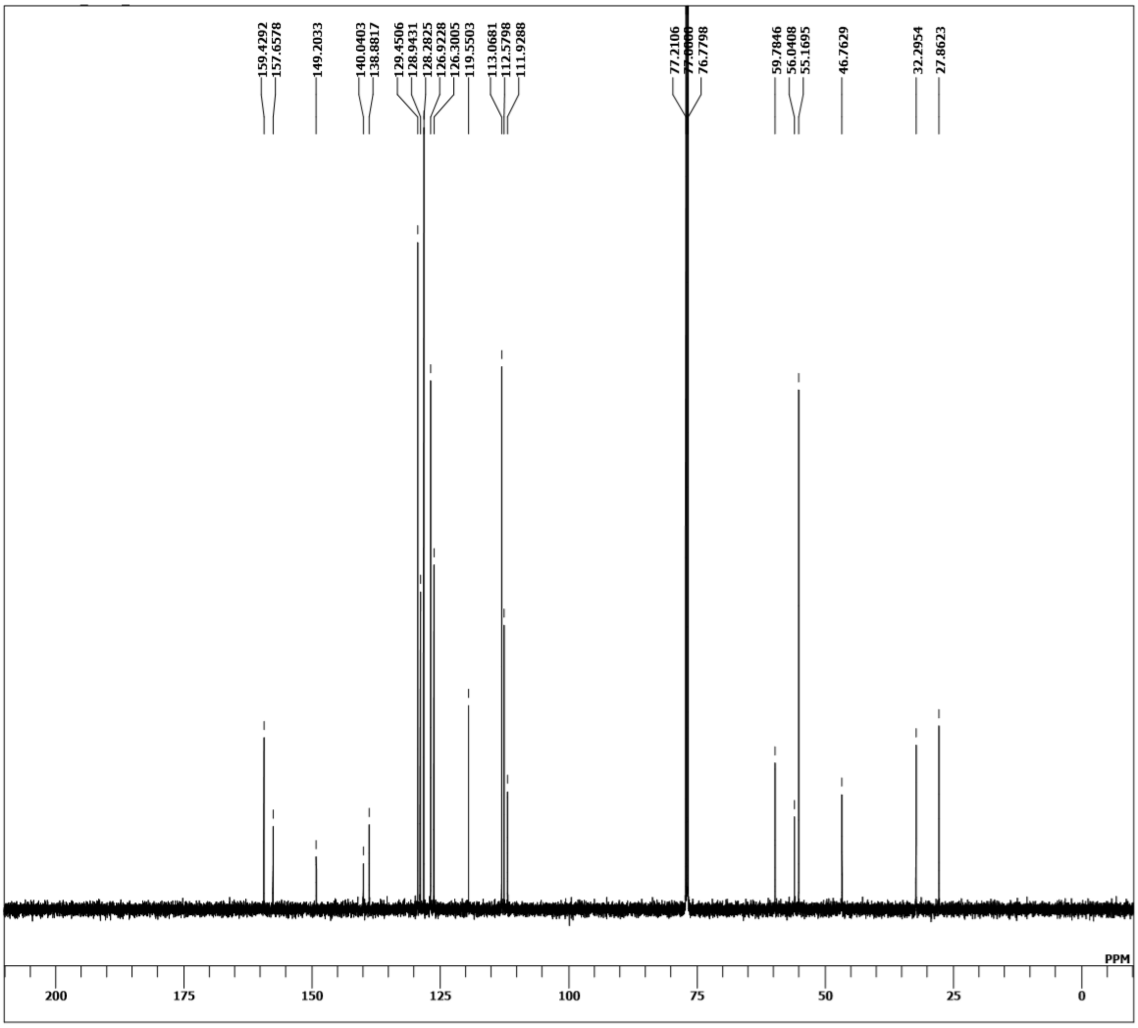

Supplementary Figure 66 HPLC analysis of **3ia**

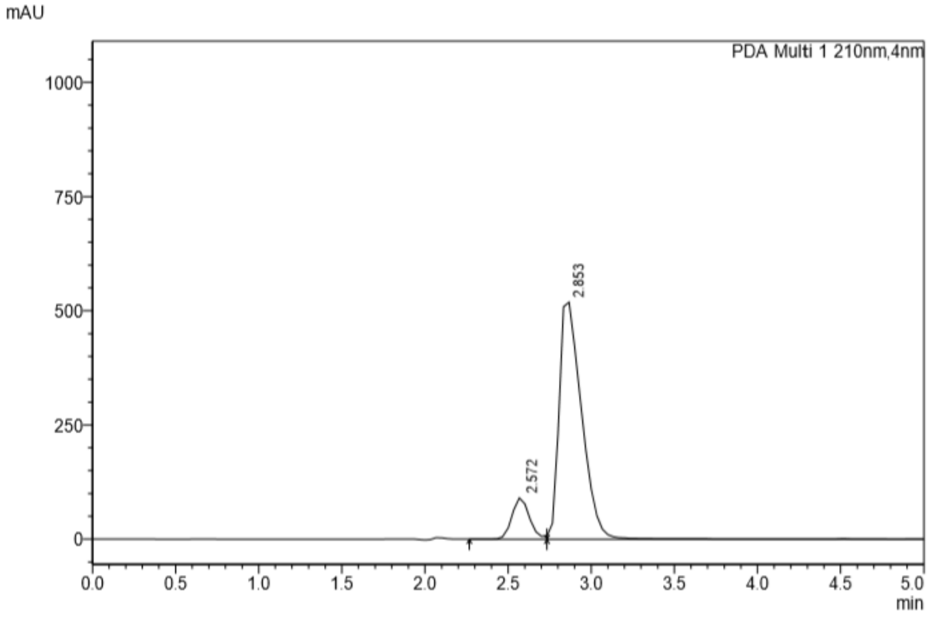

Peak Table

PDA Ch1 210nm

Peak#	Ret. Time	Area	Height	Area%
1	2.572	669690	90339	12.034
2	2.853	4895497	518938	87.966
‡Œv		5565187	609277	100.000

Supplementary Figure 67 HPLC analysis of **3ia** (racemic)

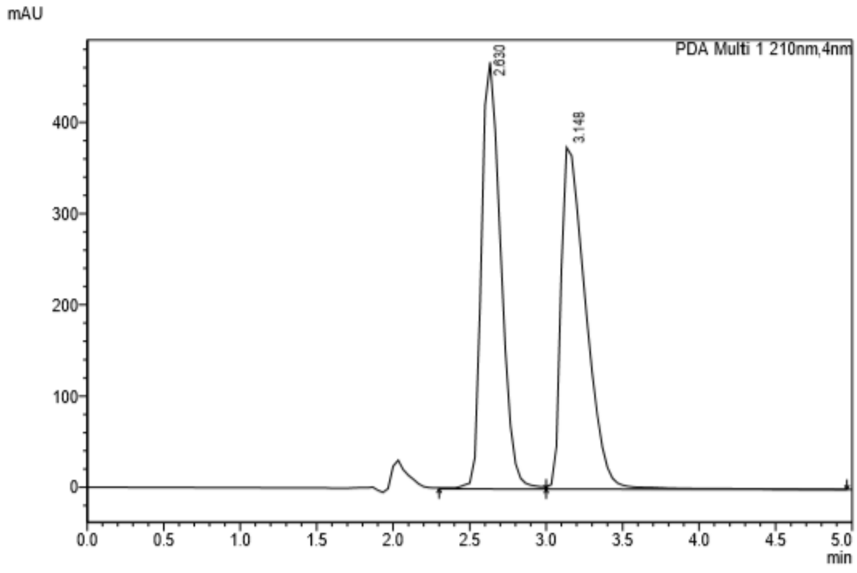

Peak Table

PDA Ch1 210nm

Peak#	Ret. Time	Area	Height	Area%
1	2.630	4164062	467794	49.635
2	3.148	4225323	374313	50.365
‡Œv		8389385	842107	100.000

Supplementary Figure 68 ^{1}H NMR of **3ja**

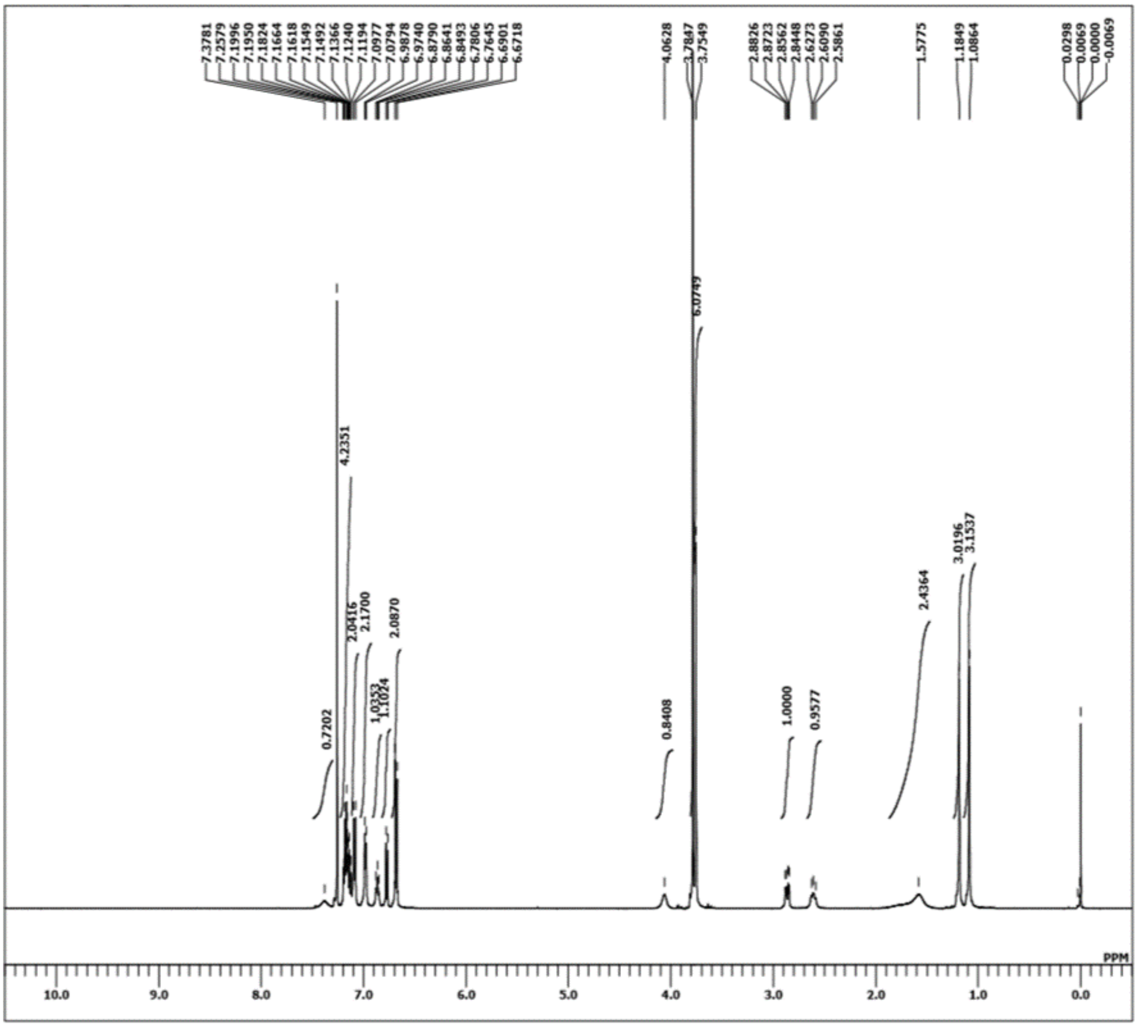

Supplementary Figure 69 ^{13}C NMR of **3ja**

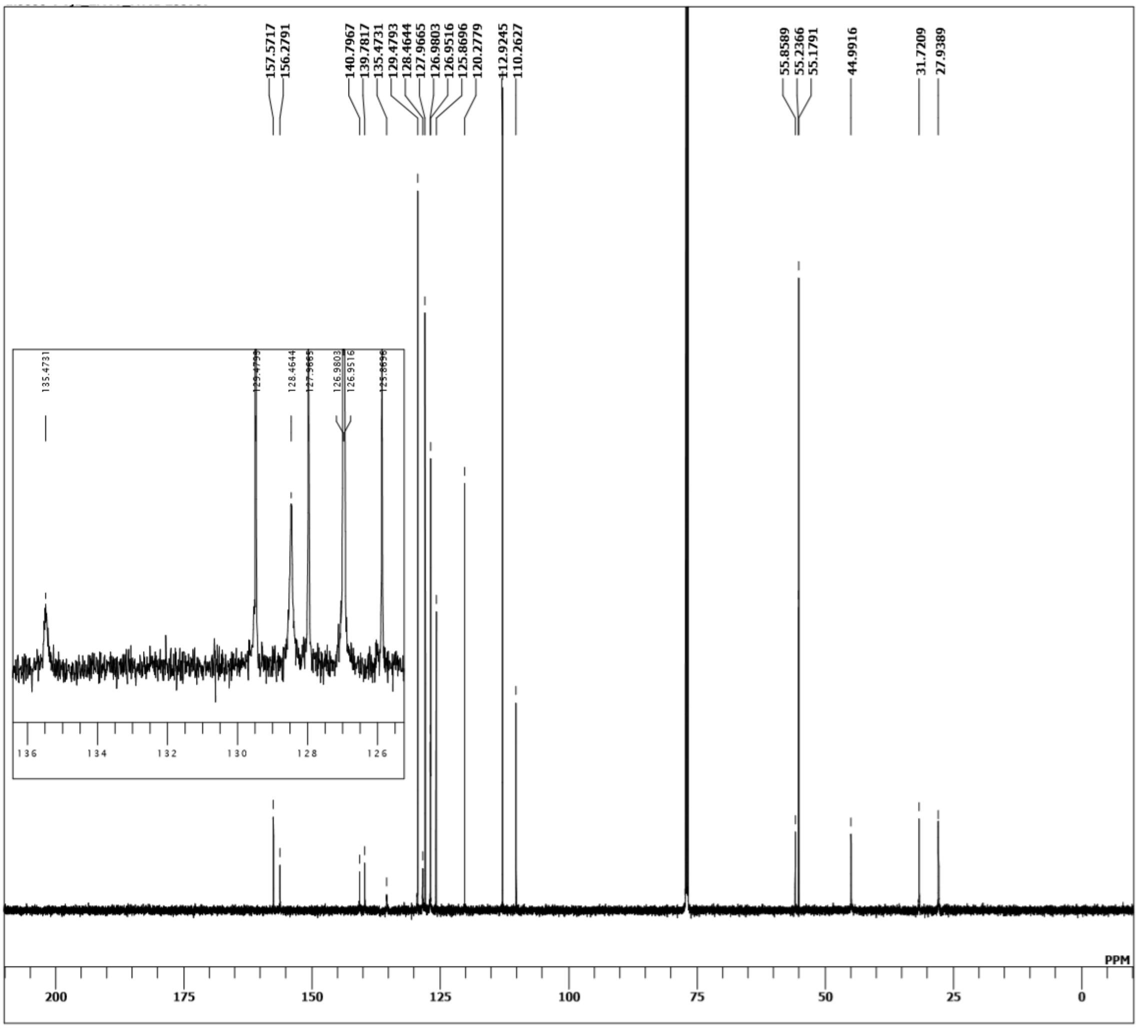

Supplementary Figure 70 HPLC analysis of **3ja**

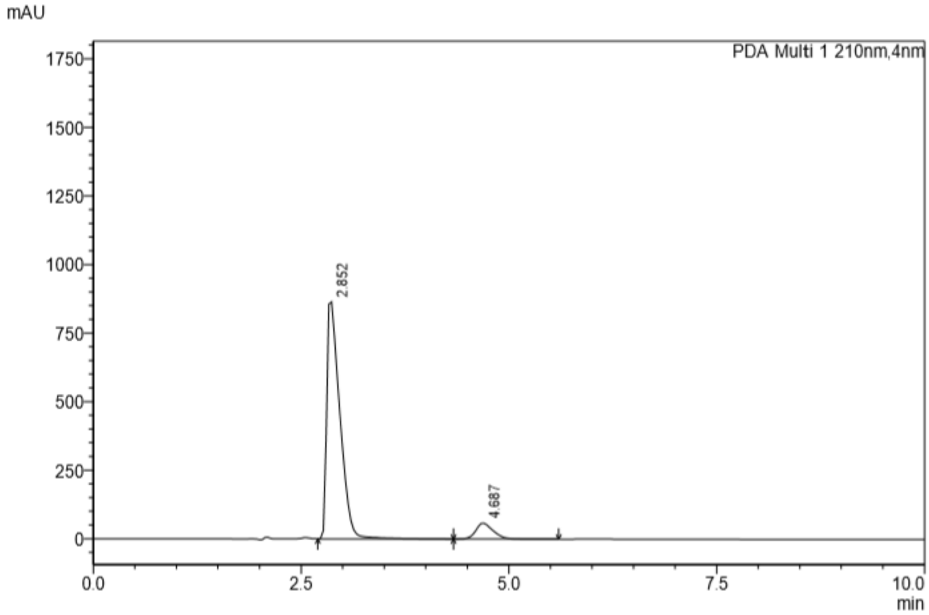

Peak Table

PDA Ch1 210nm

Peak#	Ret. Time	Area	Height	Area%
1	2.852	9594749	864355	91.885
2	4.687	847398	57856	8.115
‡Œv		10442148	922211	100.000

Supplementary Figure 71 HPLC analysis of **3ja** (racemic)

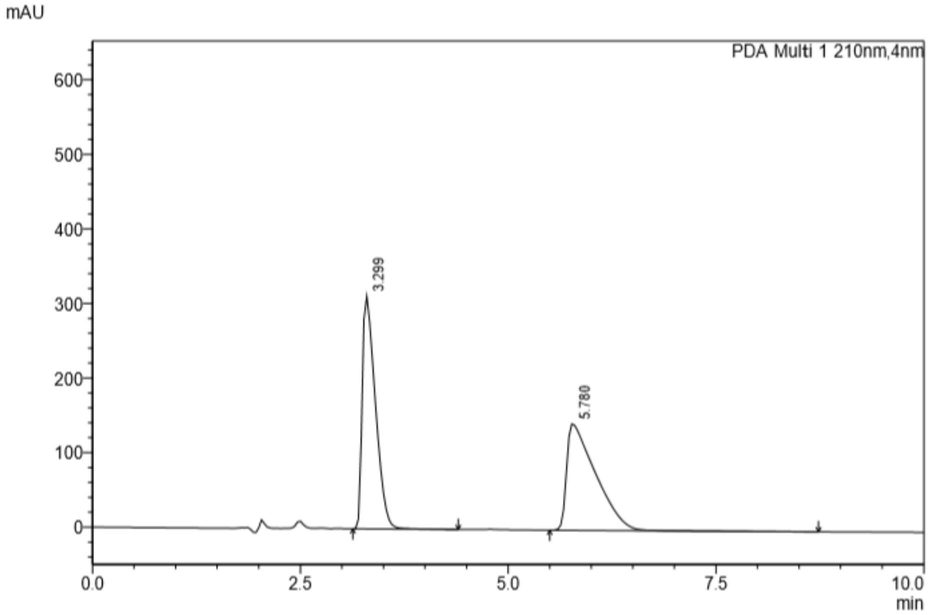

Peak Table

PDA Ch1 210nm

Peak#	Ret. Time	Area	Height	Area%
1	3.299	3410816	312179	49.732
2	5.780	3447518	142853	50.268
‡Œv		6858334	455032	100.000

Supplementary Figure 72 ^{1}H NMR of **3ka**

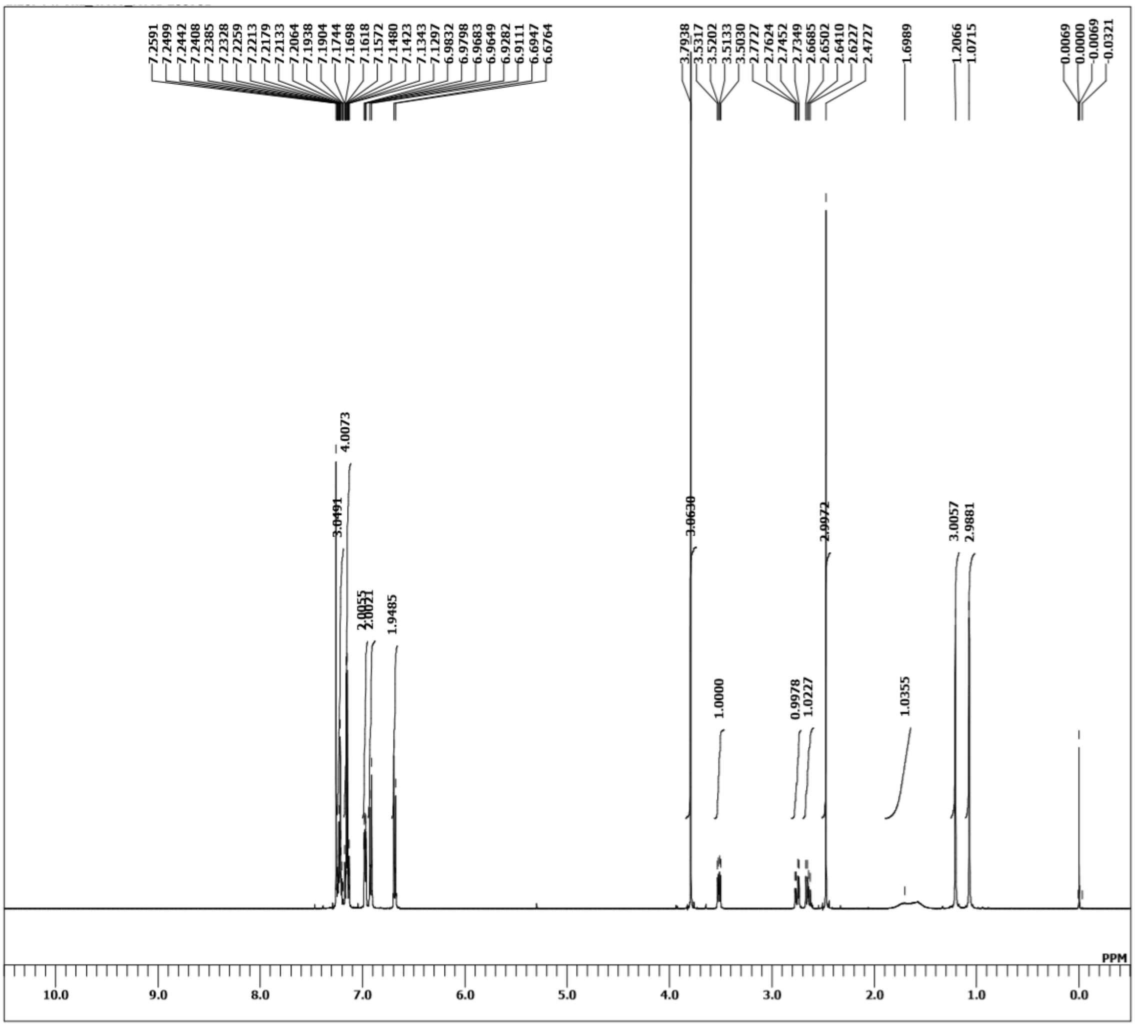

Supplementary Figure 73 ^{13}C NMR of **3ka**

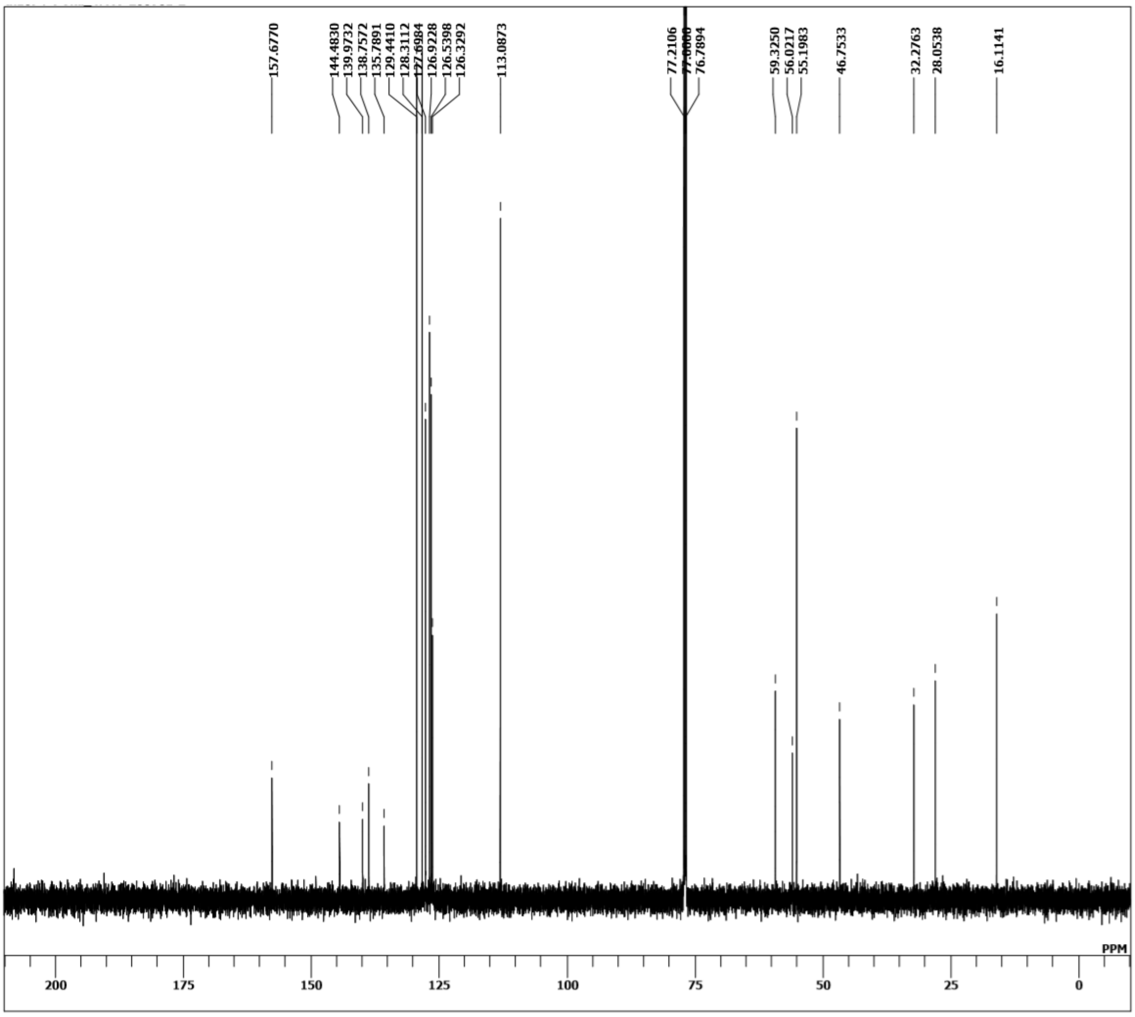

Supplementary Figure 74 ^{1}H NMR of **3la**

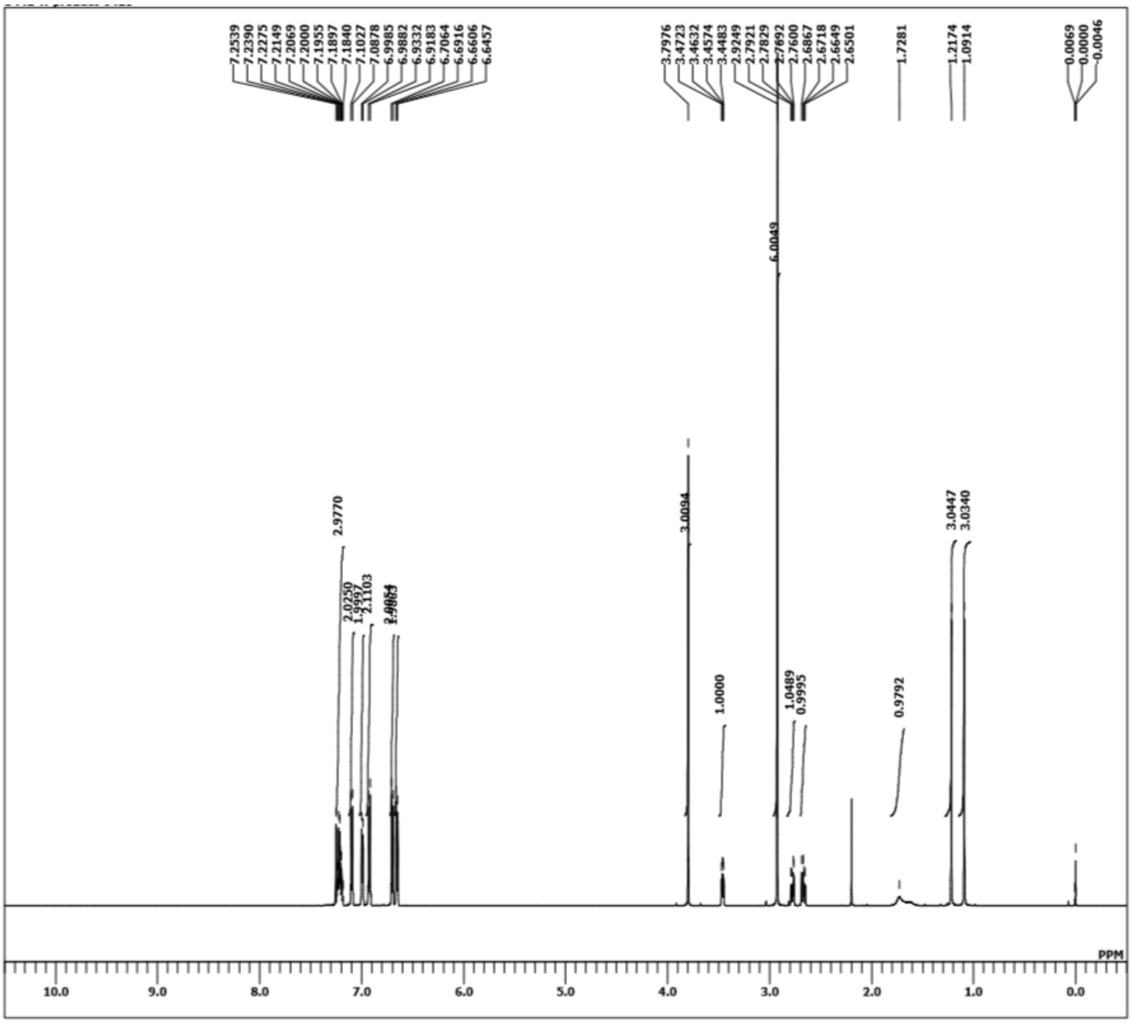

Supplementary Figure 75 ^{13}C NMR of **3la**

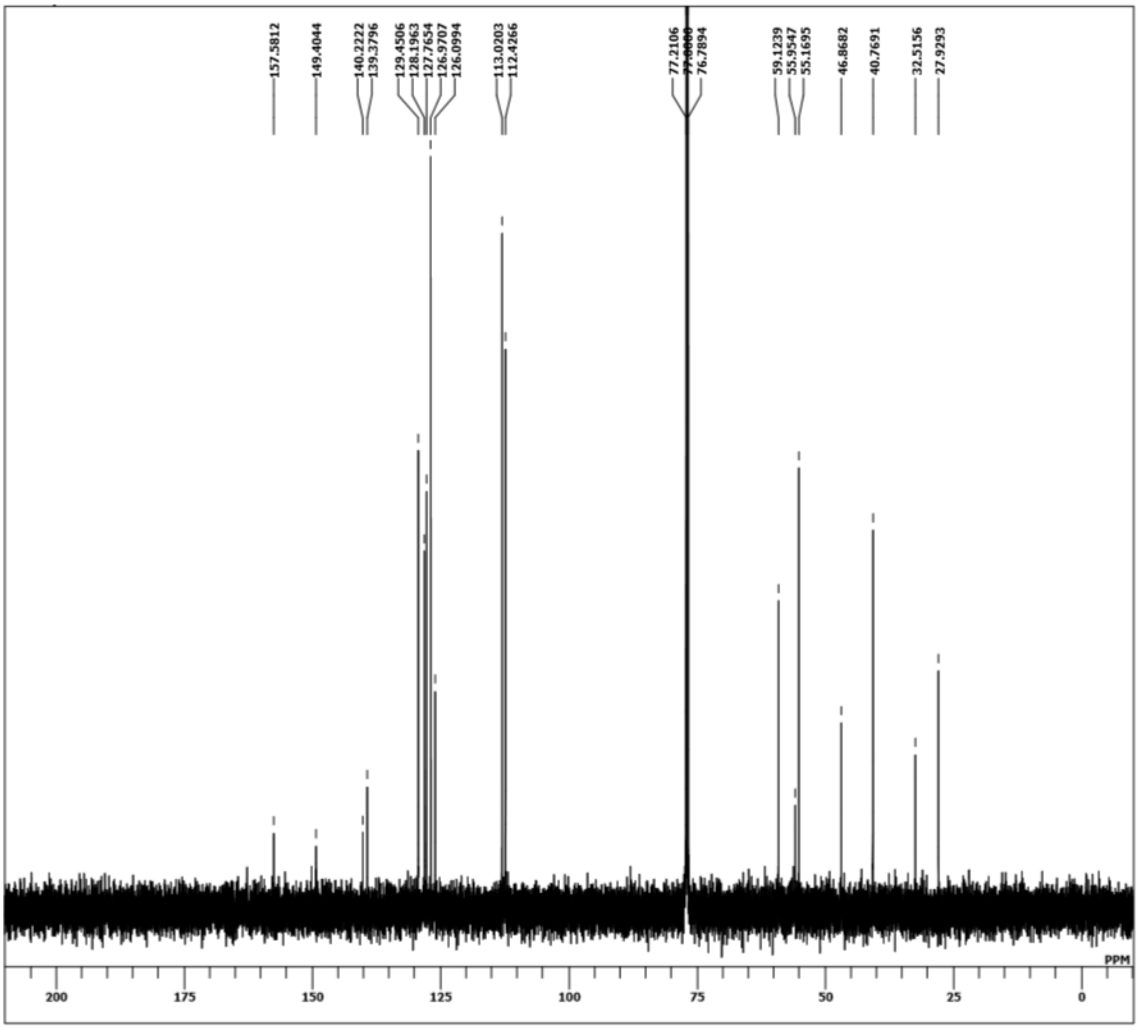

Supplementary Figure 76 HPLC analysis of **3la**

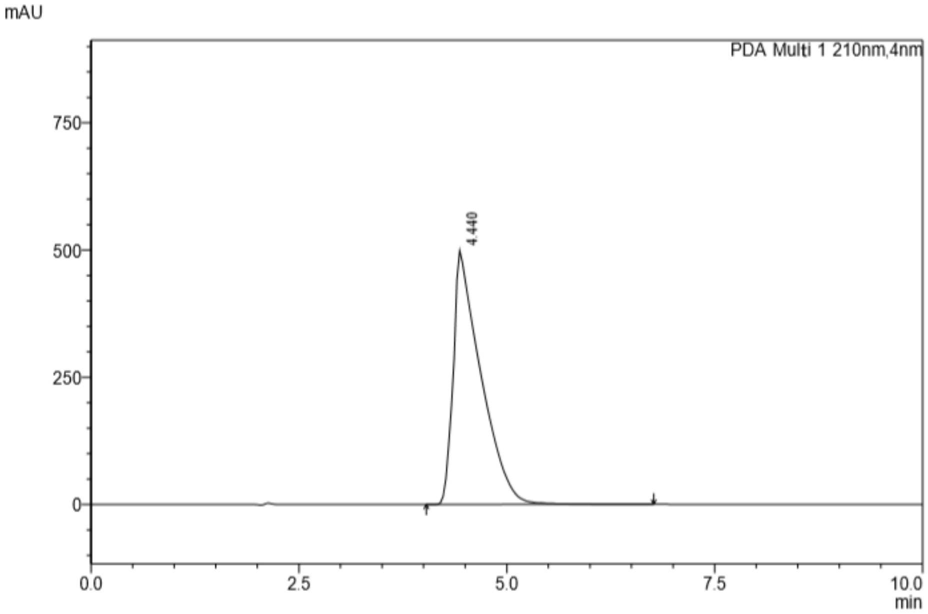

Peak Table

PDA Ch1 210nm

Peak#	Ret. Time	Area	Height	Area%
1	4.440	11564744	498939	100.000
±Œv		11564744	498939	100.000

Supplementary Figure 77 HPLC analysis of **3la** (racemic)

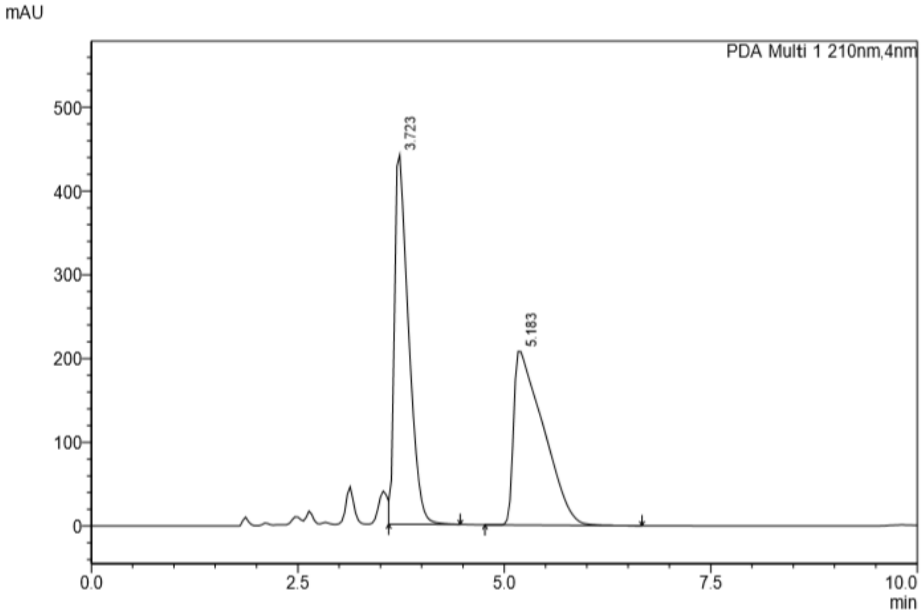

Peak Table

PDA Ch1 210nm

Peak#	Ret. Time	Area	Height	Area%
1	3.723	5245909	440696	50.112
2	5.183	5222507	207199	49.888
±Œv		10468416	647895	100.000

Supplementary Figure 78 ^{1}H NMR of **3ab**

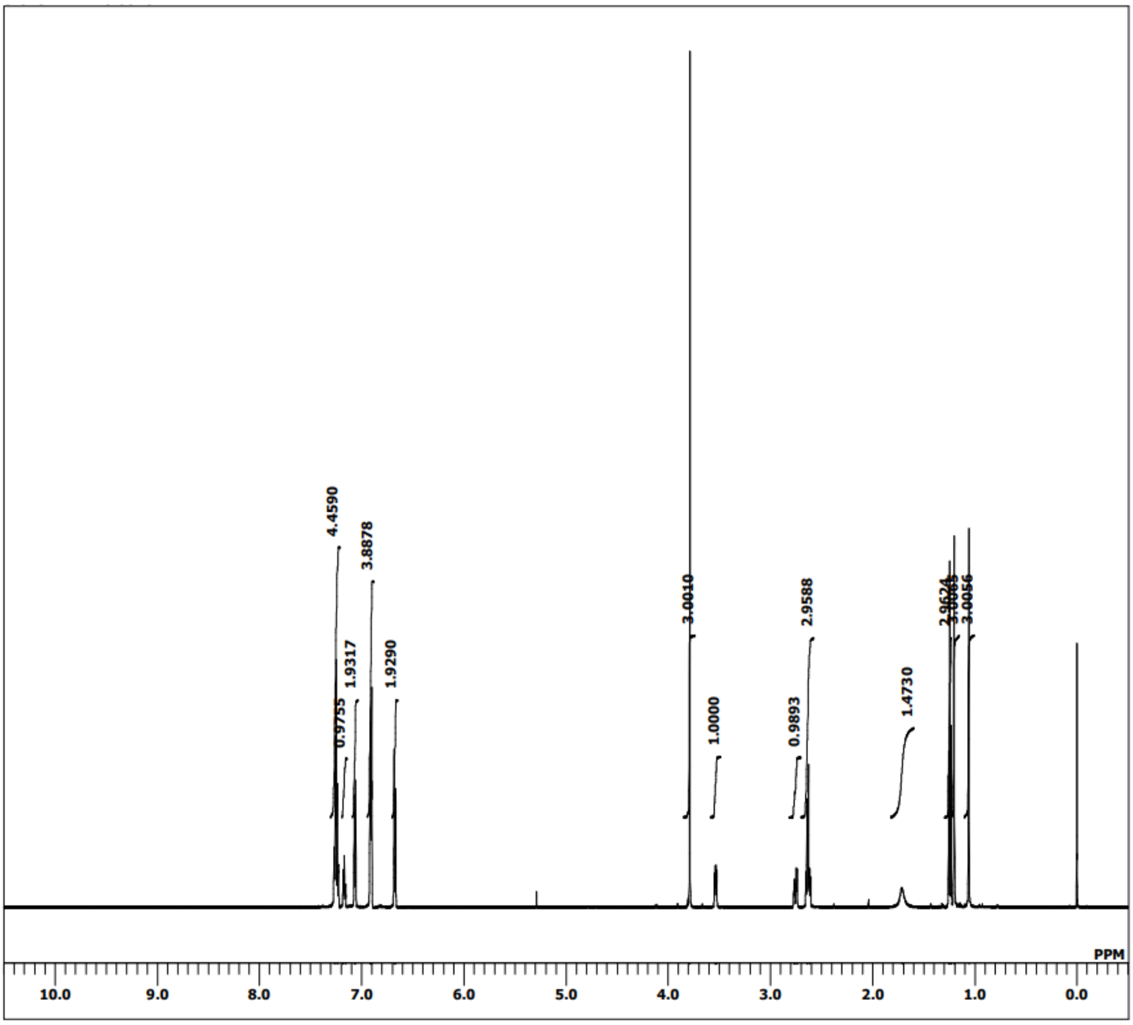

Supplementary Figure 79 ^{13}C NMR of **3ab**

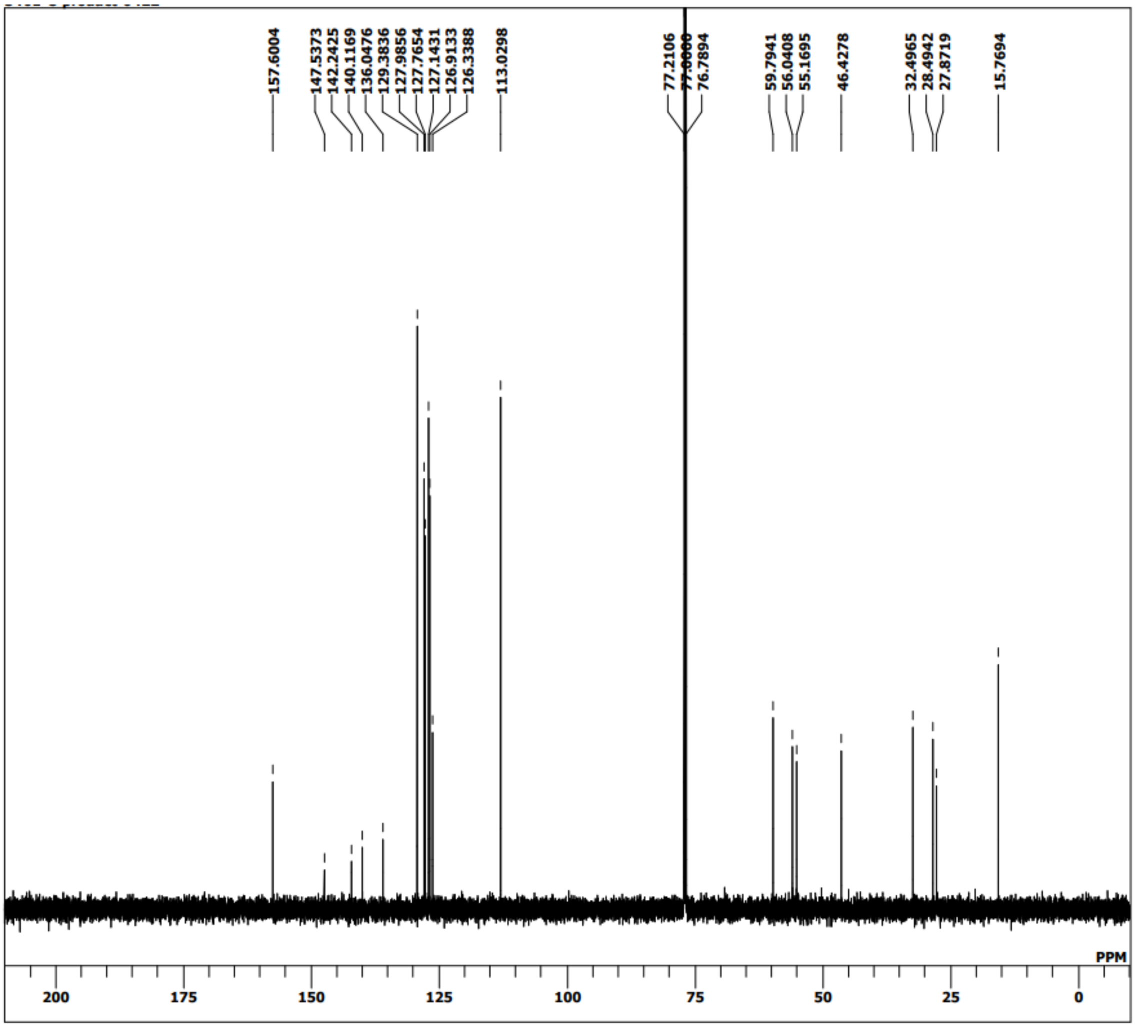

Supplementary Figure 80 ^{1}H NMR of **3ac** (crude)

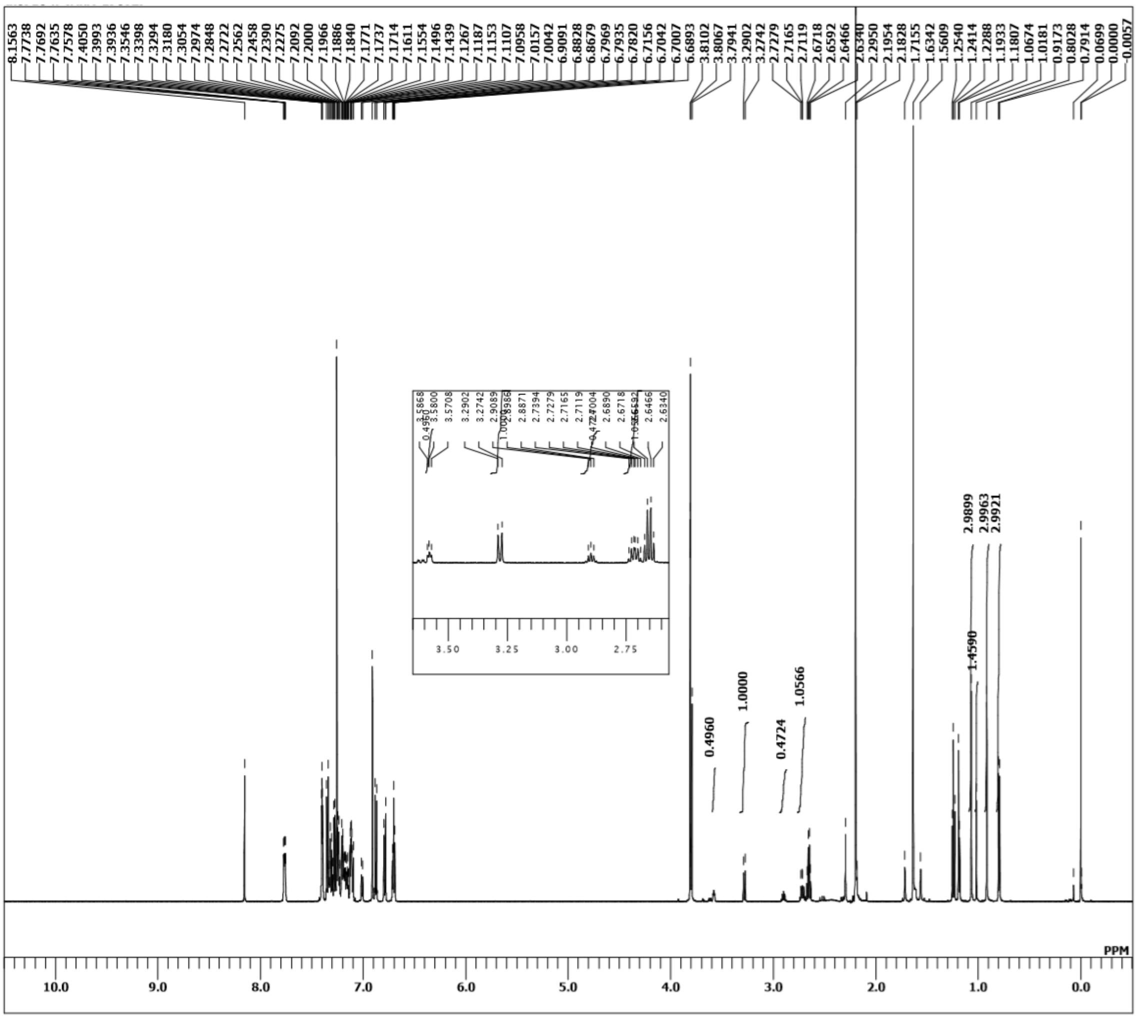

Supplementary Figure 81 ^{1}H NMR of **3ac**

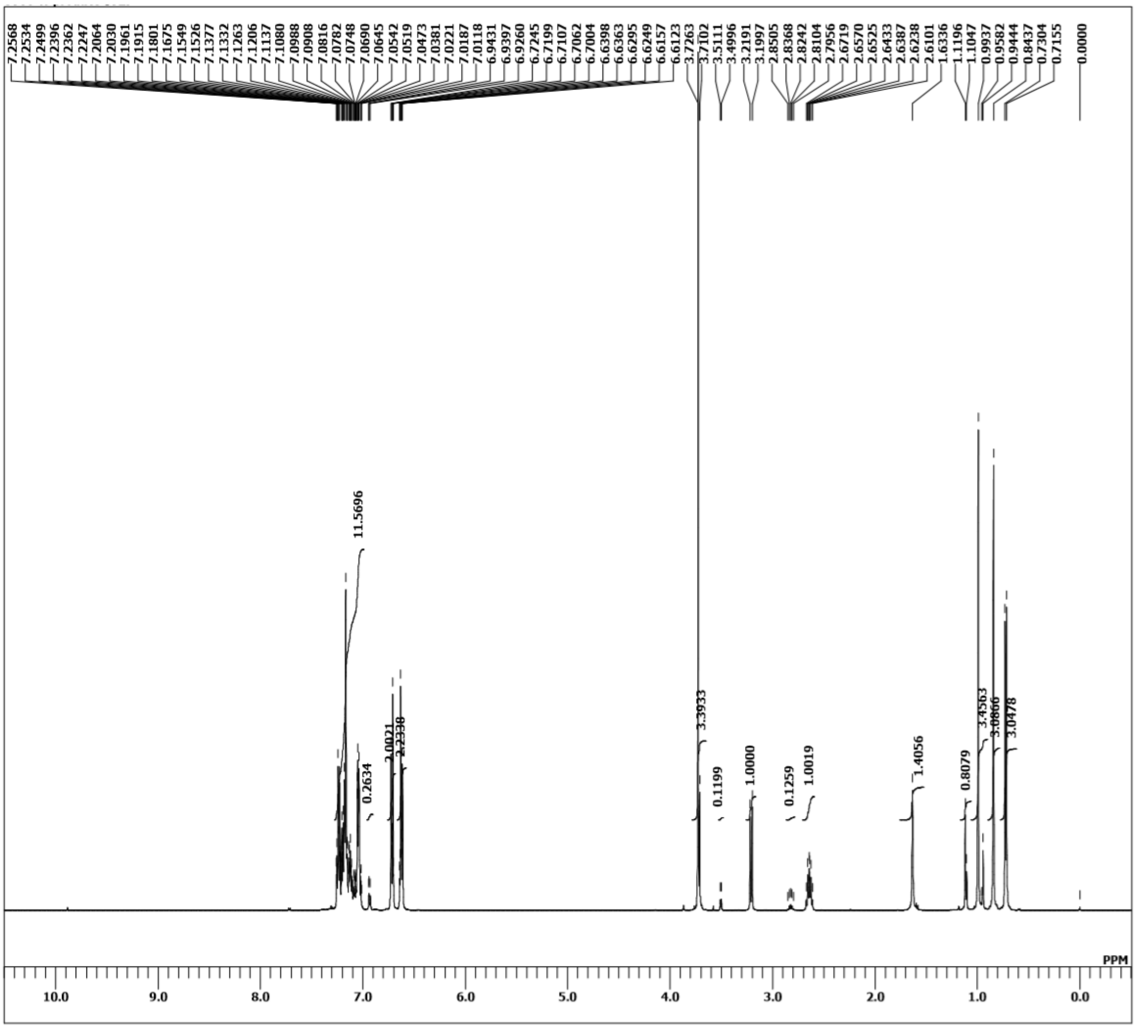

Supplementary Figure 82 ^{13}C NMR of **3ac**

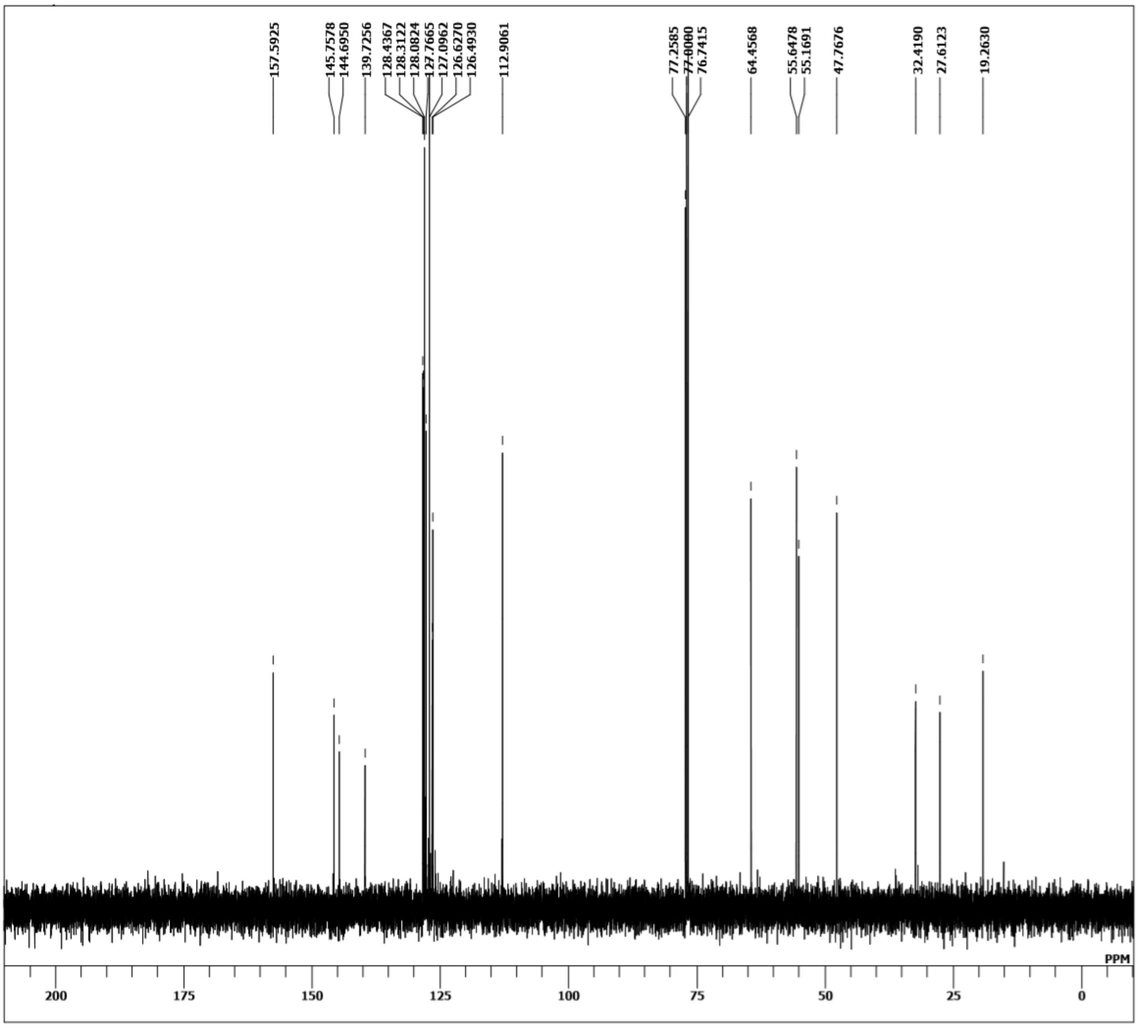

Supplementary Figure 83 HPLC analysis of **3ac**

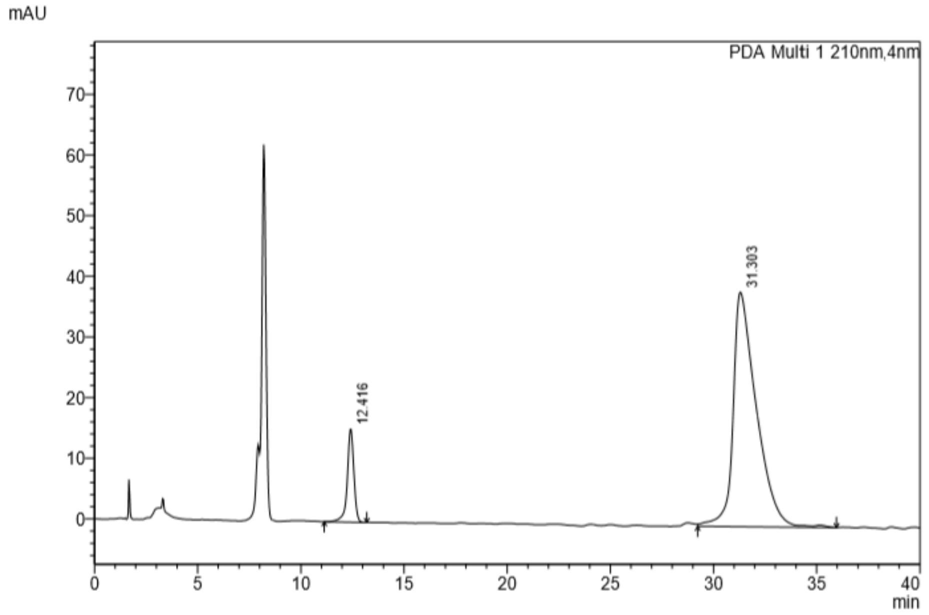

Peak Table

PDA Ch1 210nm

Peak#	Ret. Time	Area	Height	Area%
1	12.416	344193	15307	10.140
2	31.303	3050338	38664	89.860
‡Œv		3394531	53972	100.000

Supplementary Figure 84 HPLC analysis of **3ac** (racemic)

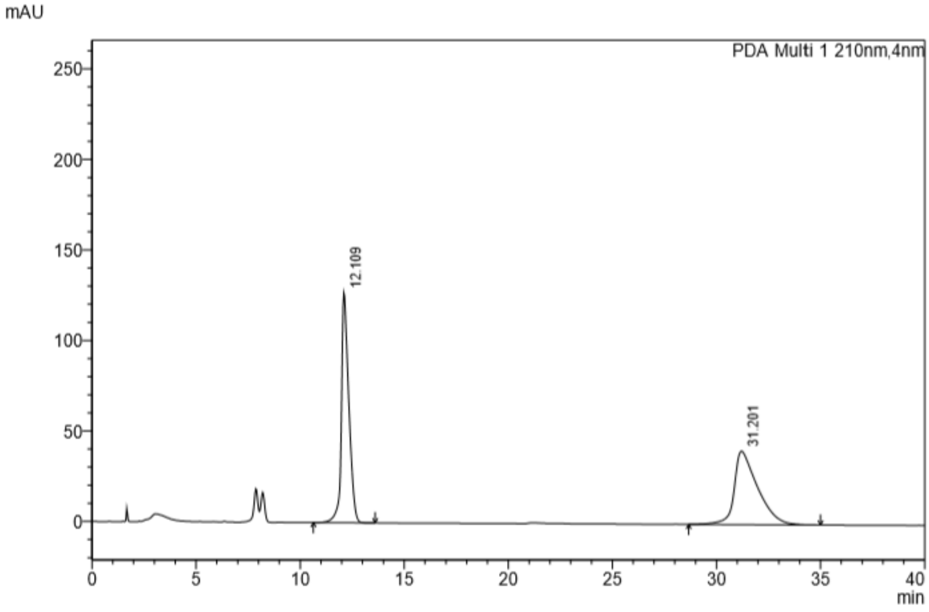

Peak Table

PDA Ch1 210nm

Peak#	Ret. Time	Area	Height	Area%
1	12.109	3165824	127123	49.937
2	31.201	3173786	40571	50.063
‡Œv		6339610	167693	100.000

Supplementary Figure 85 ^{1}H NMR of **3ad**

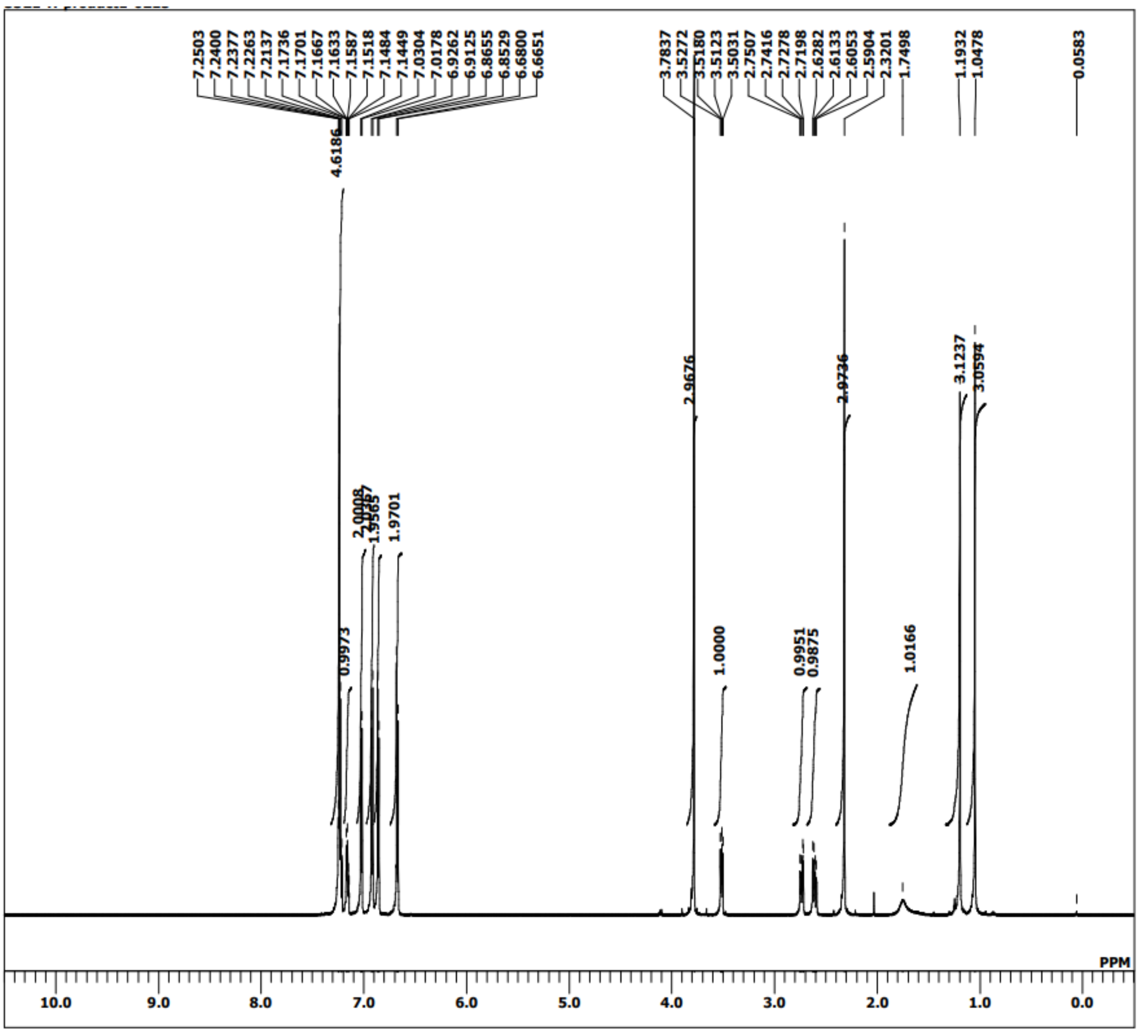

Supplementary Figure 86 ^{13}C NMR of **3ad**

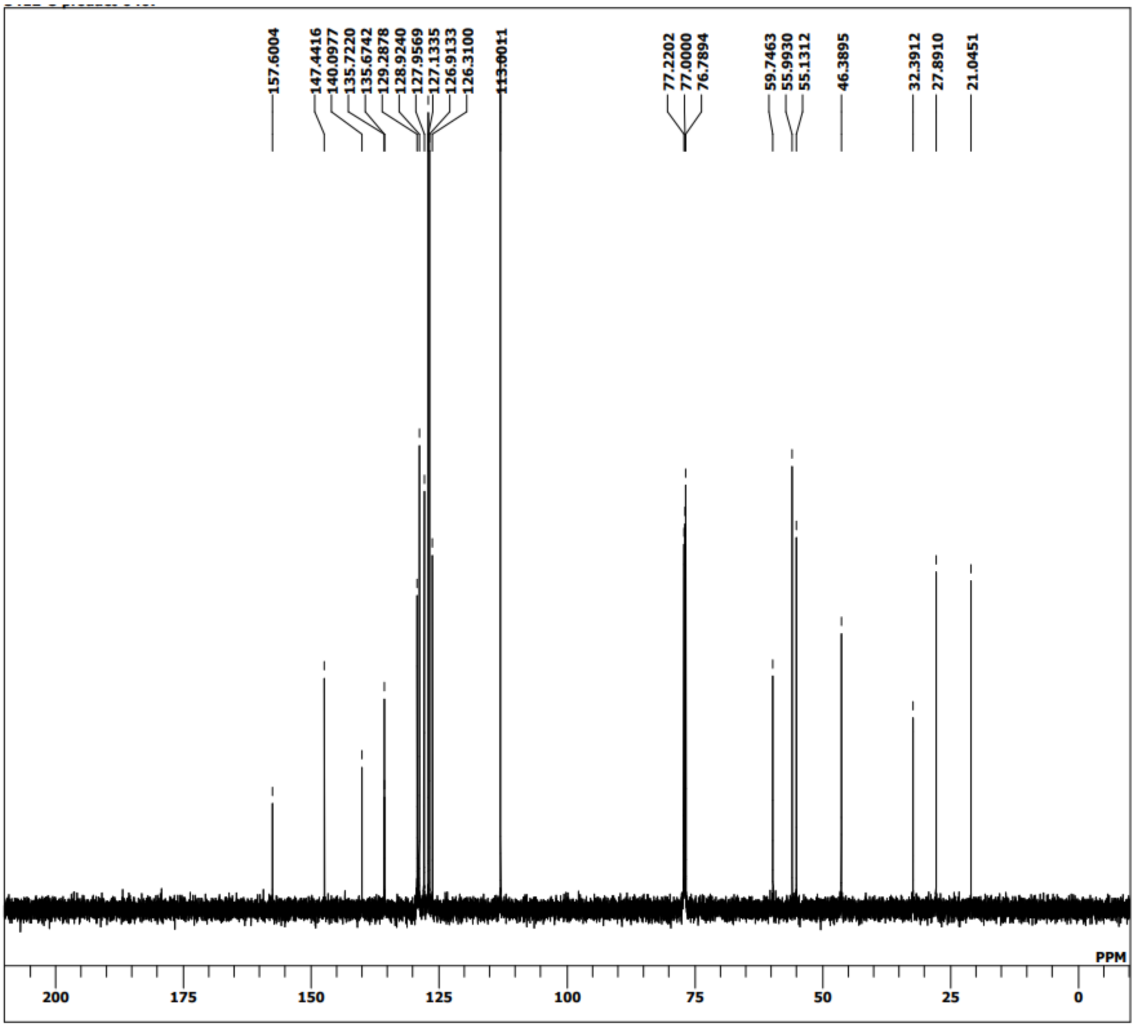

Supplementary Figure 87 HPLC analysis of **3ad**

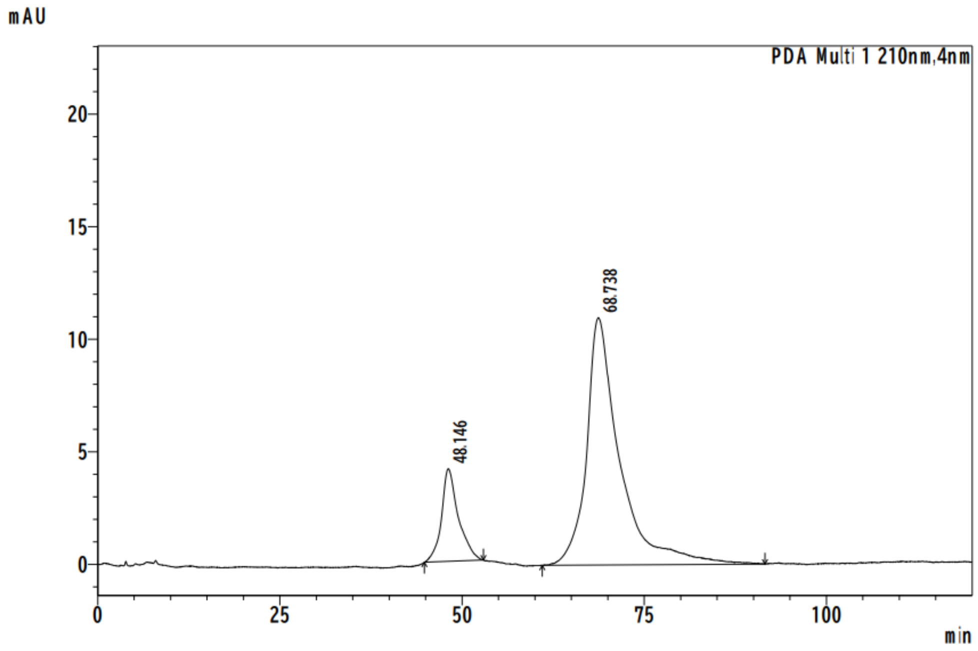

Peak Table

PDA Ch1 210nm

Peak#	Ret. Time	Area	Height	Area%
1	48.146	663022	4118	16.026
2	68.738	3474193	10985	83.974
‡Œv		4137215	15102	100.000

Supplementary Figure 88 HPLC analysis of **3ad** (racemic)

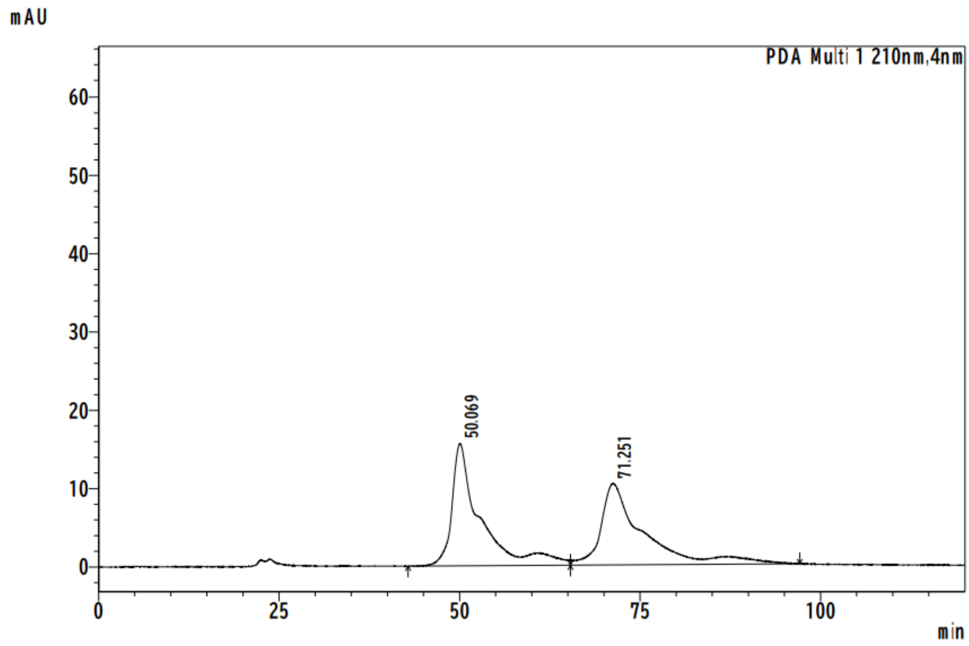

Peak Table

PDA Ch1 210nm

Peak#	Ret. Time	Area	Height	Area%
1	50.069	4251528	15639	50.189
2	71.251	4219505	10396	49.811
‡Œv		8471034	26035	100.000

Supplementary Figure 89 ^{1}H NMR of **3ae**

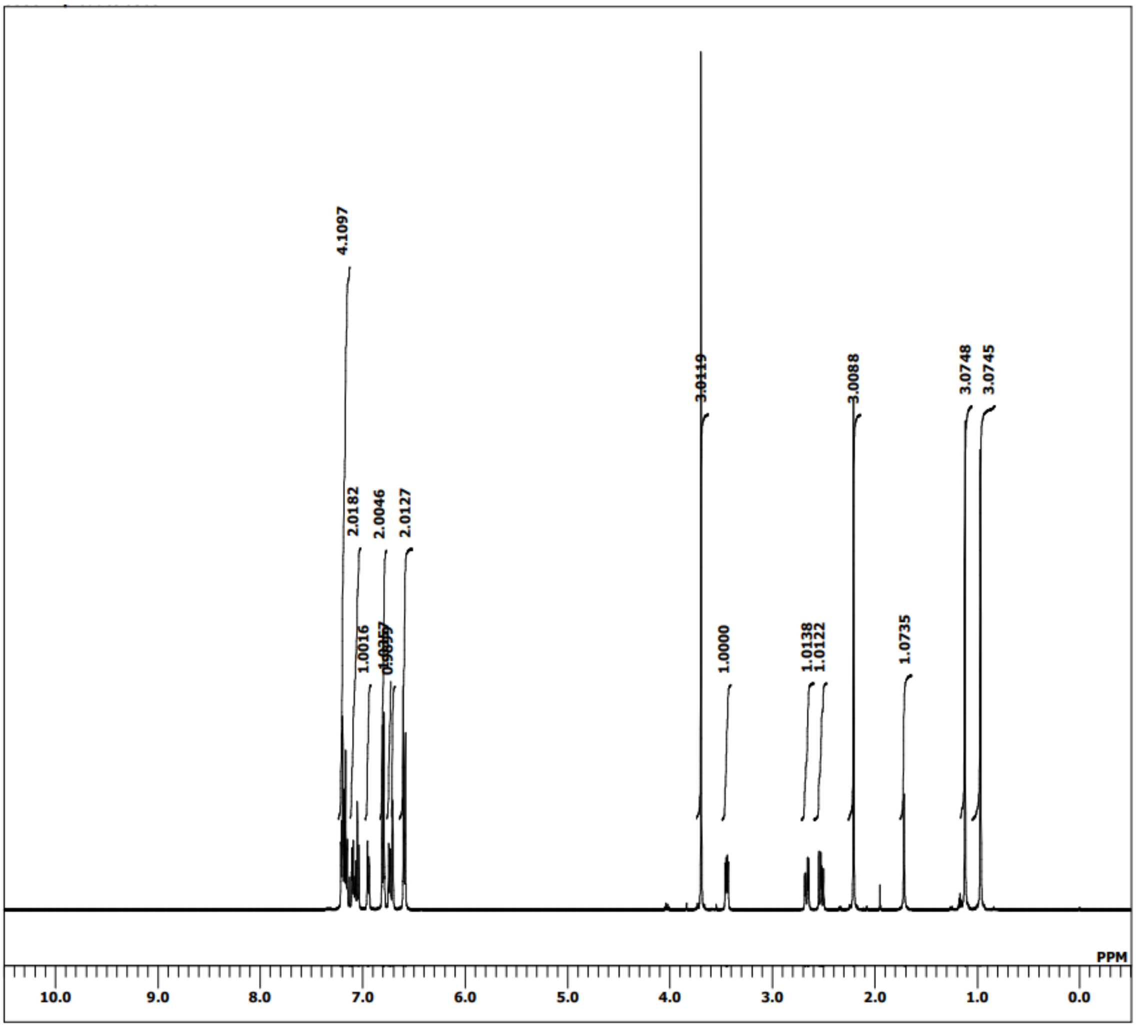

Supplementary Figure 90 ^{13}C NMR of **3ae**

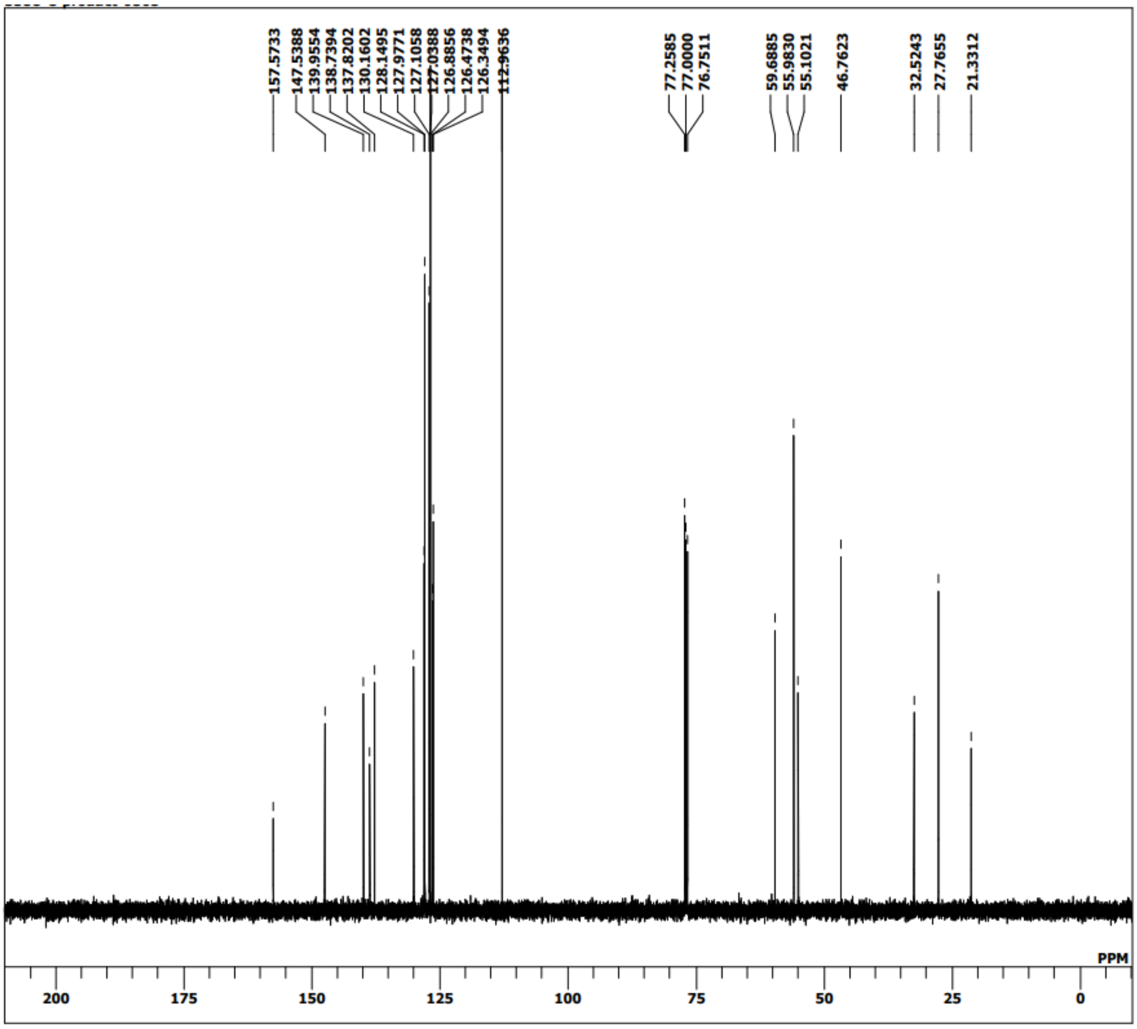

Supplementary Figure 91 ^{1}H NMR of **3af**

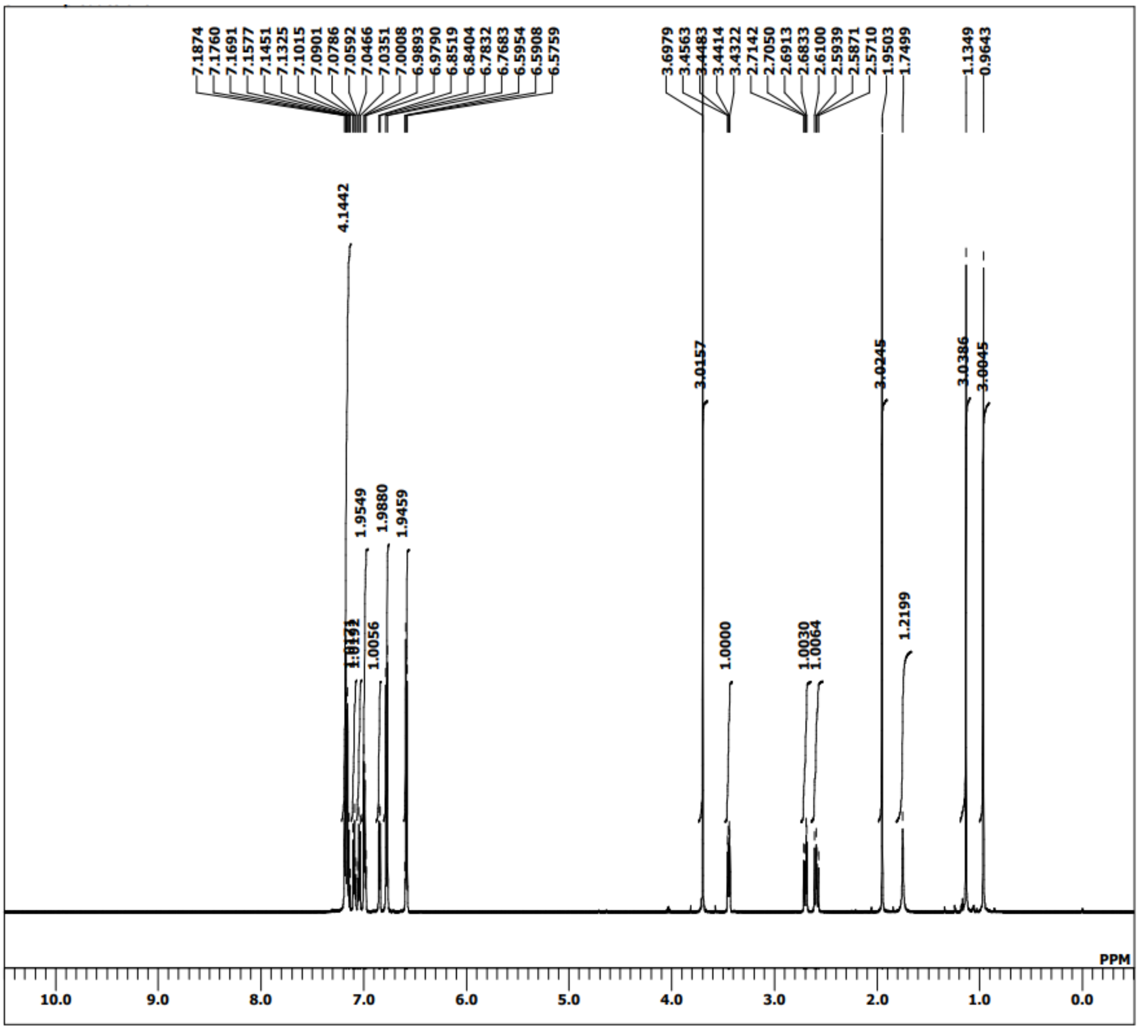

Supplementary Figure 92 ^{13}C NMR of **3af**

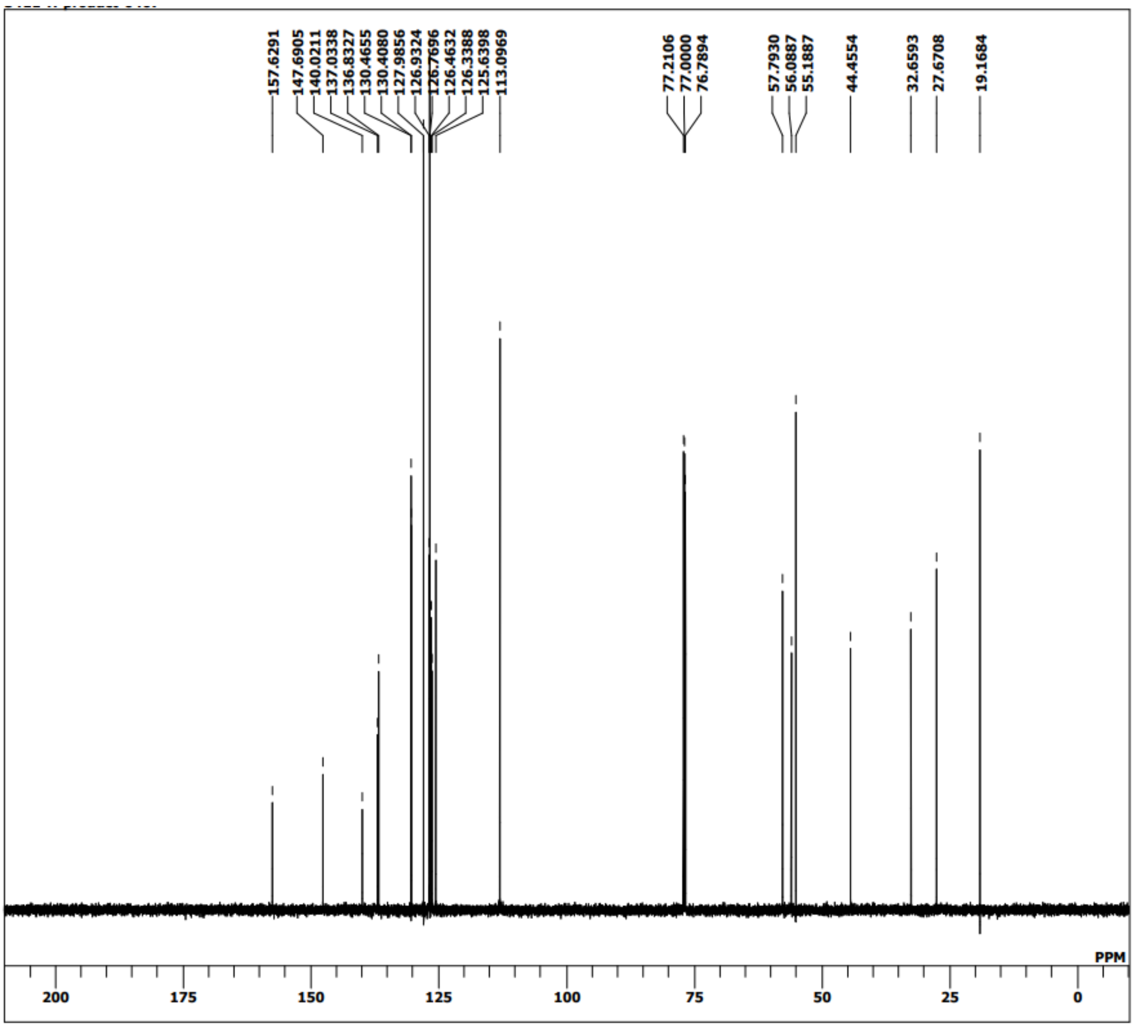

Supplementary Figure 93 HPLC analysis of **3af**

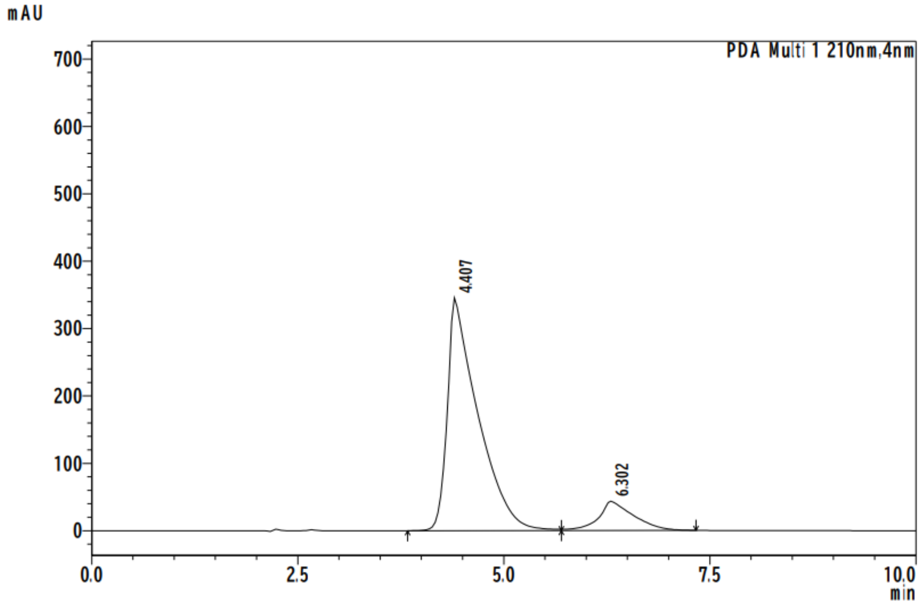

Peak Table

PDA Ch1 210nm

Peak#	Ret. Time	Area	Height	Area%
1	4.407	8947992	345717	87.554
2	6.302	1271982	43494	12.446
‡Œv		10219974	389210	100.000

Supplementary Figure 94 HPLC analysis of **3af** (racemic)

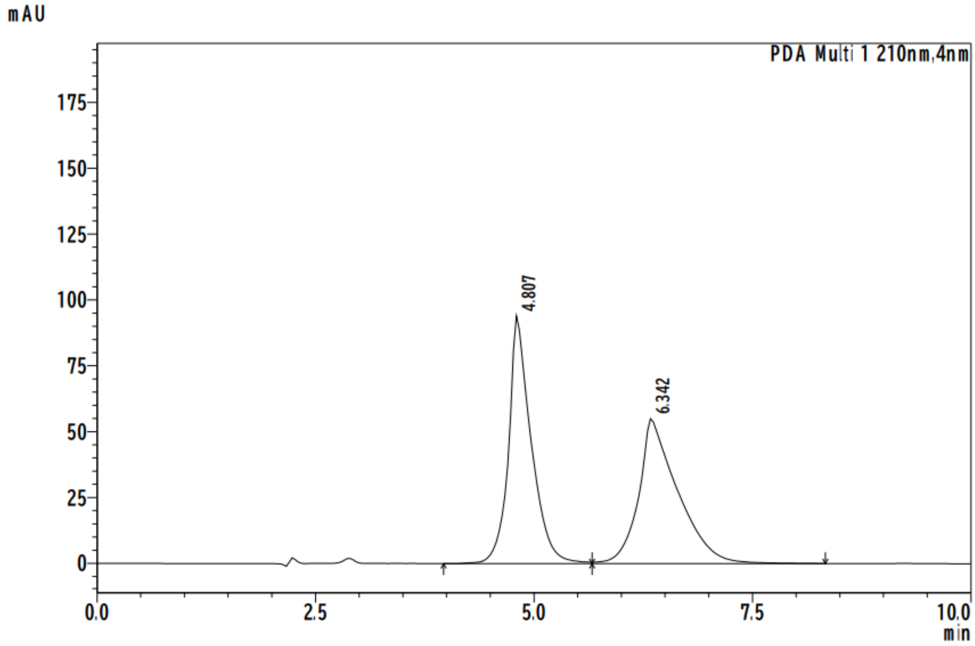

Peak Table

PDA Ch1 210nm

Peak#	Ret. Time	Area	Height	Area%
1	4.807	1694744	94026	49.824
2	6.342	1706735	55049	50.176
‡Œv		3401479	149075	100.000

Supplementary Figure 95 ^{1}H NMR of **3ah**

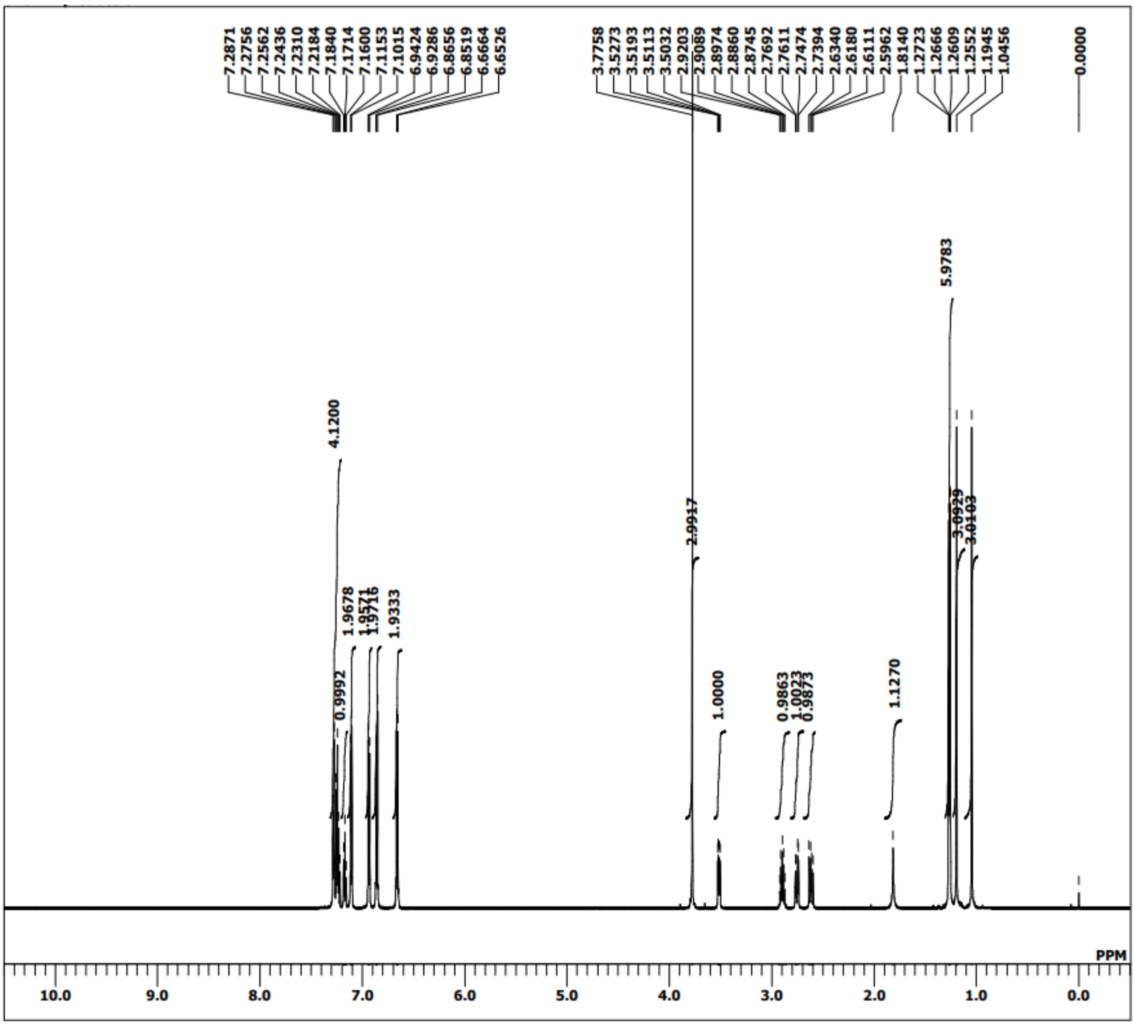

Supplementary Figure 96 ^{13}C NMR of **3ah**

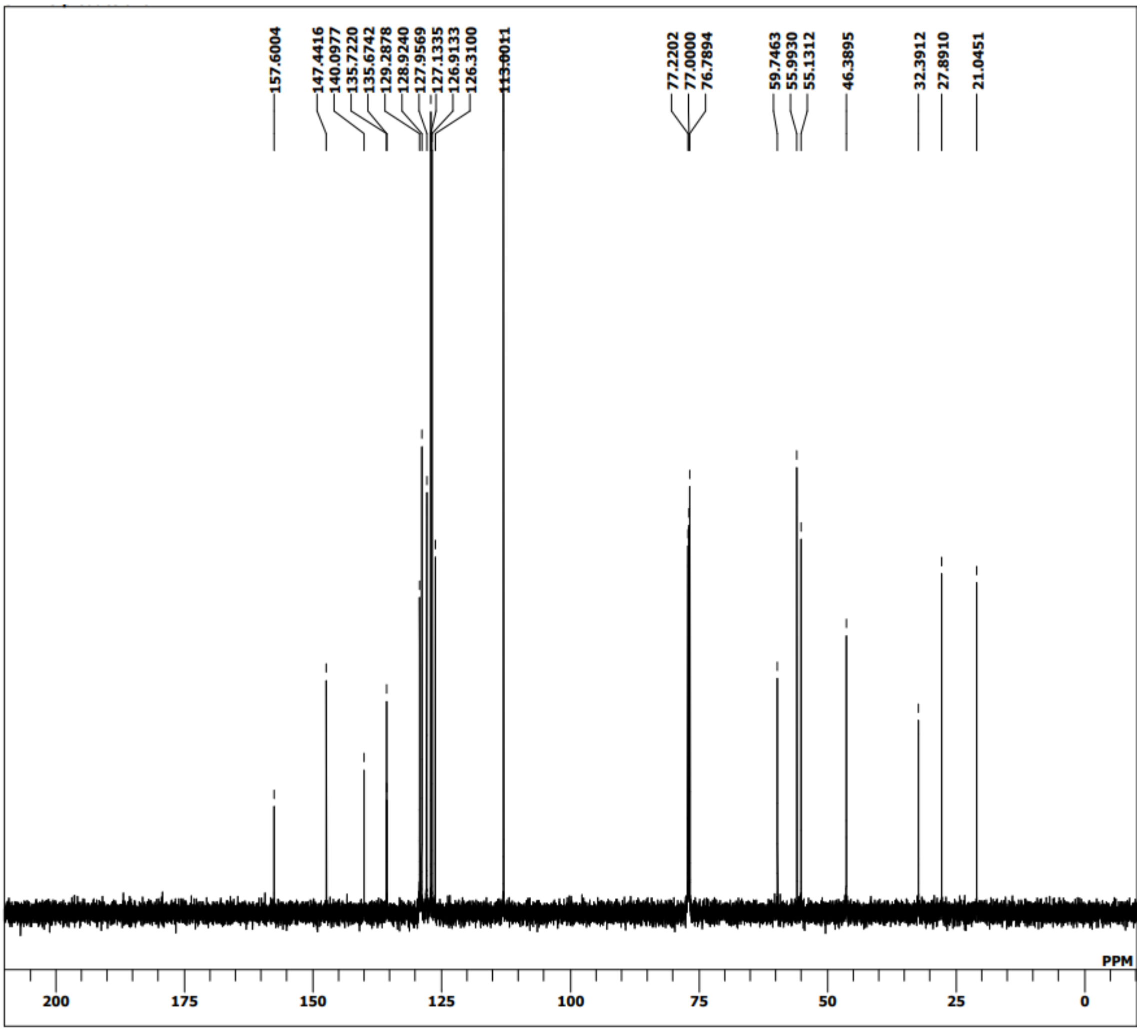

Supplementary Figure 97 HPLC analysis of **3ah**

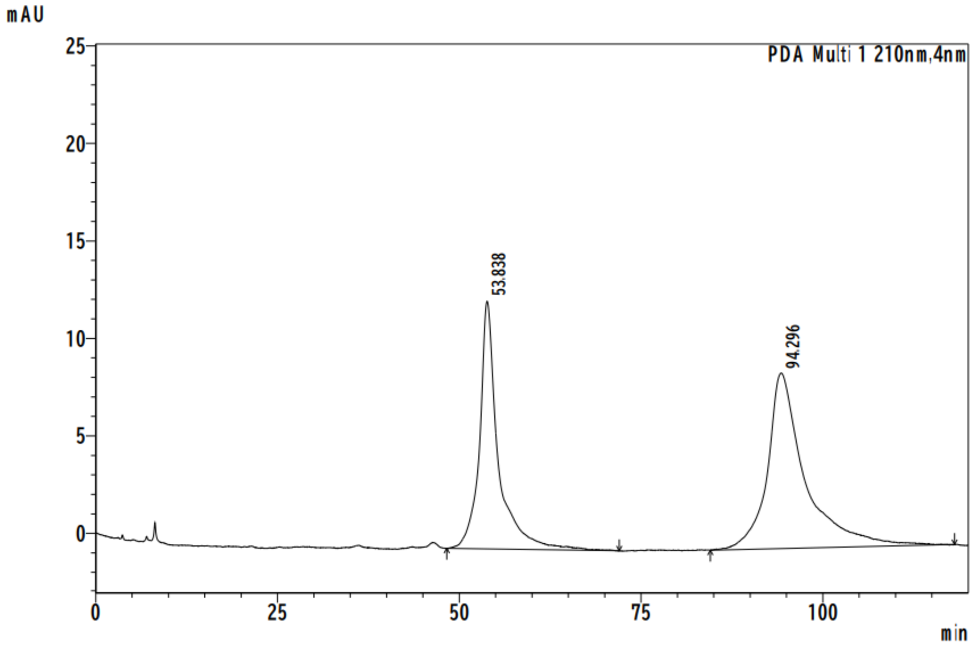

Peak Table

PDA Ch1 210nm

Peak#	Ret. Time	Area	Height	Area%
1	53.838	2277959	12705	41.189
2	94.296	3252531	9002	58.811
‡Œv		5530490	21707	100.000

Supplementary Figure 98 HPLC analysis of **3ah** (racemic)

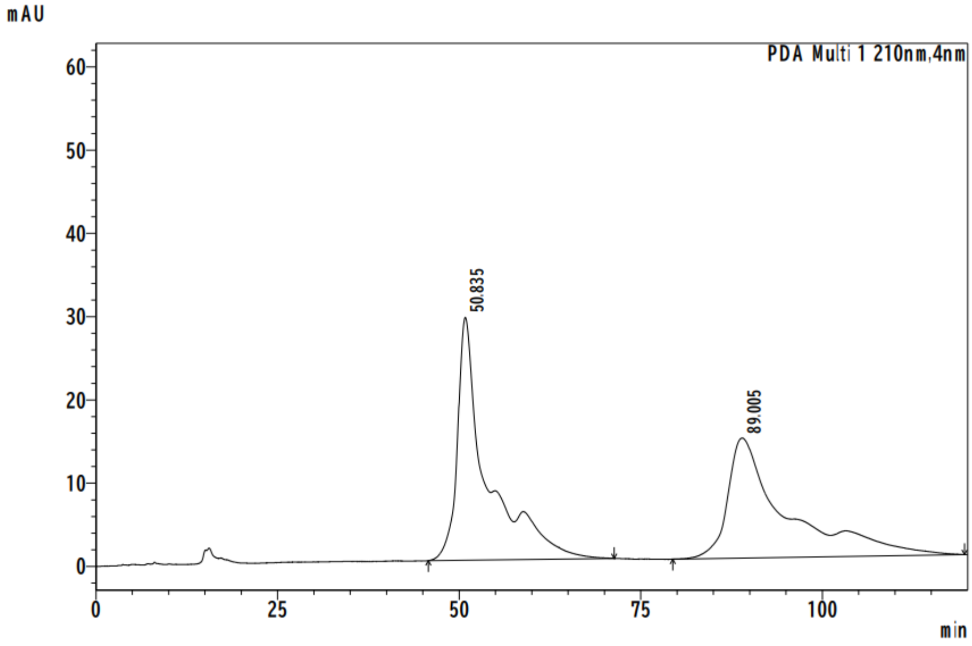

Peak Table

PDA Ch1 210nm

Peak#	Ret. Time	Area	Height	Area%
1	50.835	8273398	29161	50.722
2	89.005	8037793	14414	49.278
‡Œv		16311190	43575	100.000

Supplementary Figure 99 ^{1}H NMR of **4aa**

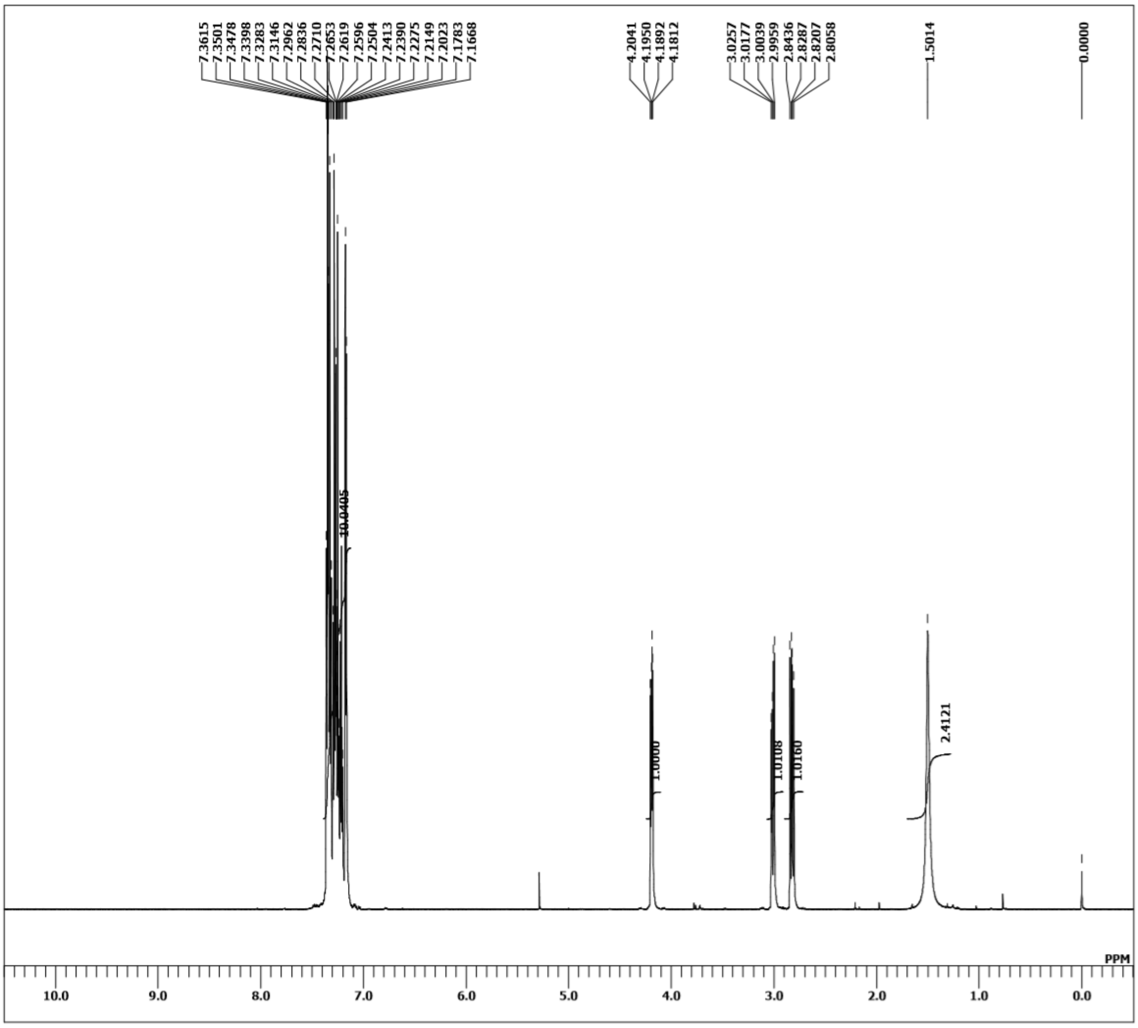

Supplementary Figure 100 ^{13}C NMR of **4aa**

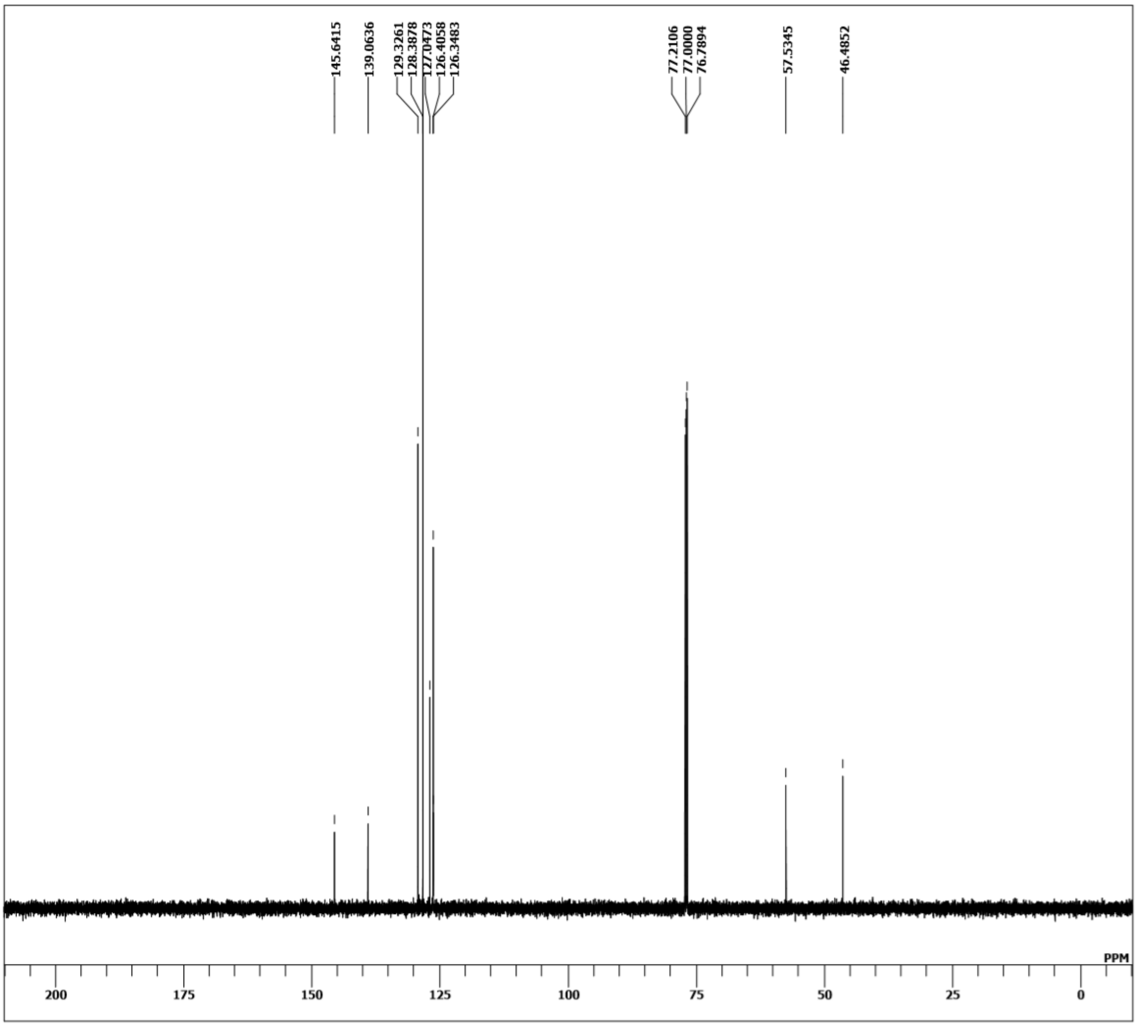

Supplementary Figure 101 HPLC analysis of **4aa**

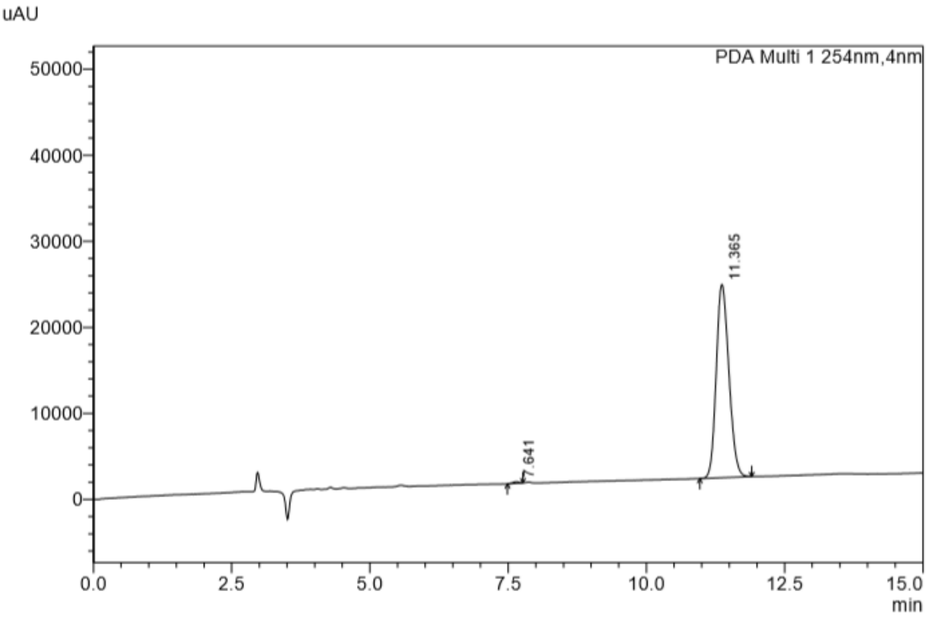

Peak Table

PDA Ch1 254nm

Peak#	Ret. Time	Area	Height	Area%
1	7.641	1487	179	0.415
2	11.365	357113	22447	99.585
Total		358600	22626	100.000

Supplementary Figure 102 HPLC analysis of **4aa** (racemic)

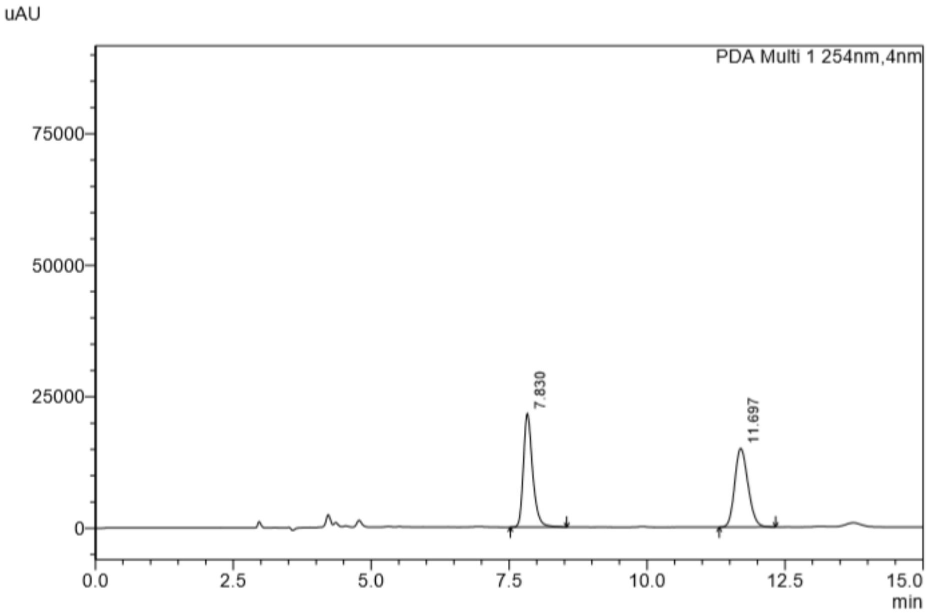

Peak Table

PDA Ch1 254nm

Peak#	Ret. Time	Area	Height	Area%
1	7.830	252276	21612	50.027
2	11.697	252009	14990	49.973
Total		504285	36602	100.000

Supplementary Figure 103 ^{1}H NMR of **4ba**

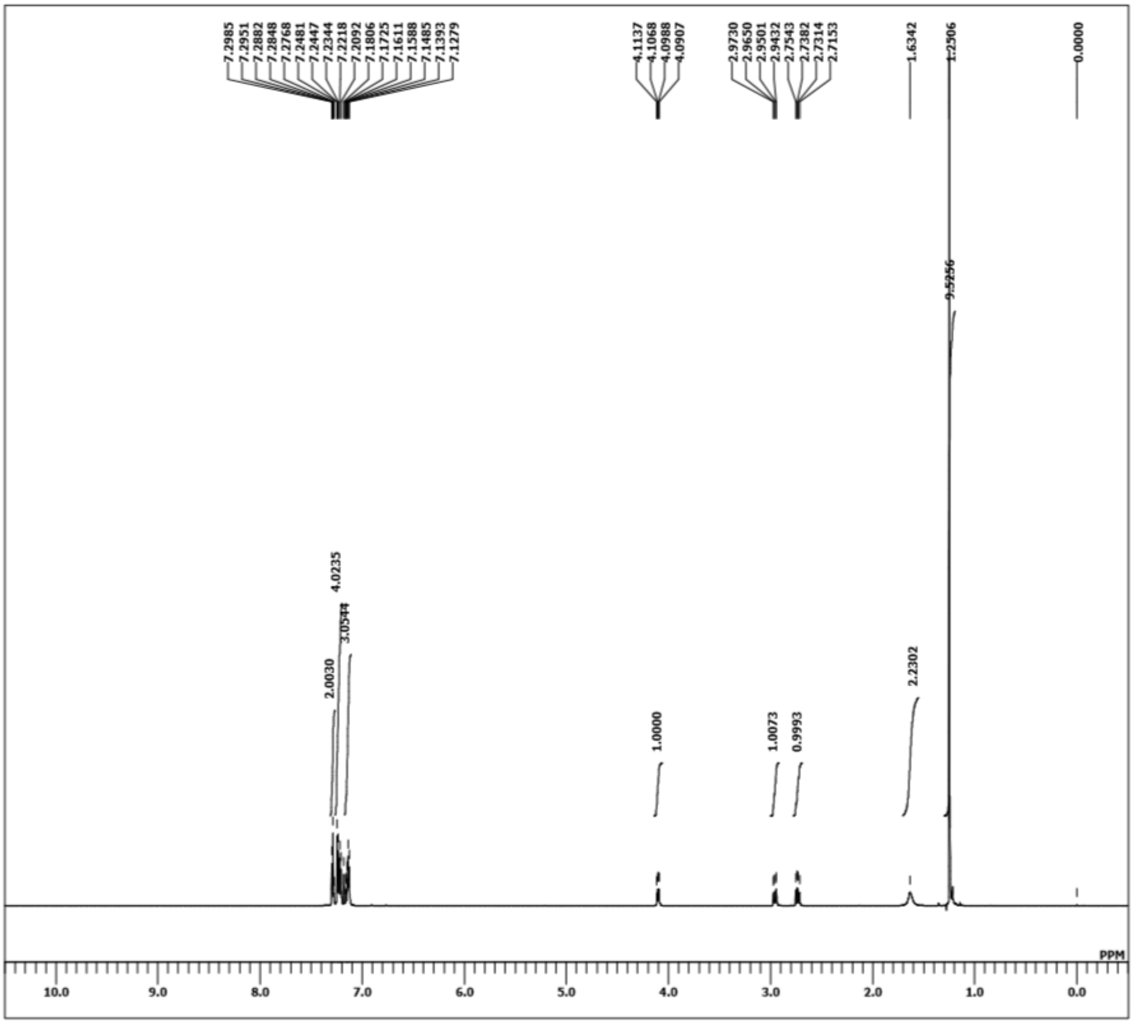

Supplementary Figure 104 ^{13}C NMR of **4ba**

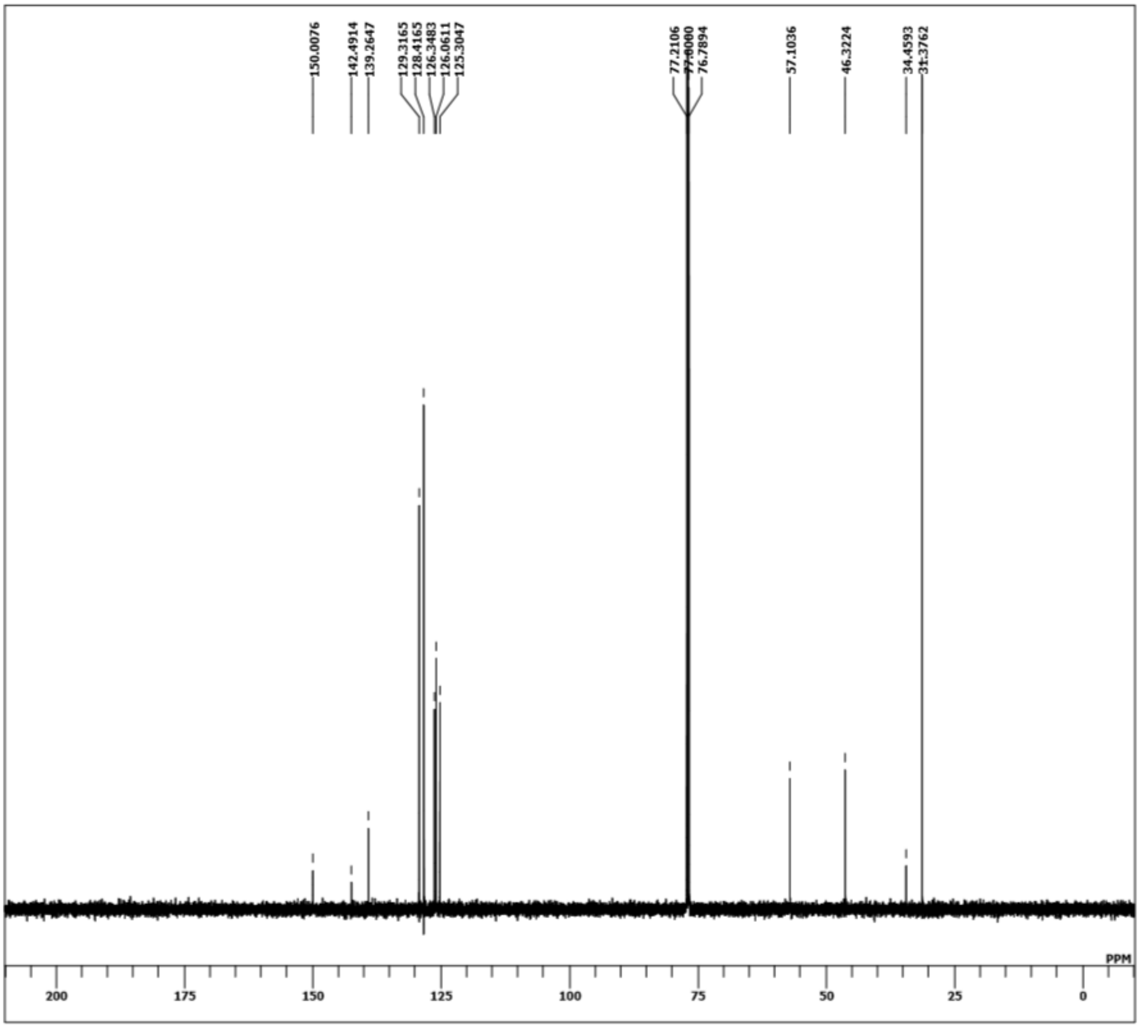

Supplementary Figure 105 HPLC analysis of **4ba** (Daicel Chiralpak OD-H column)

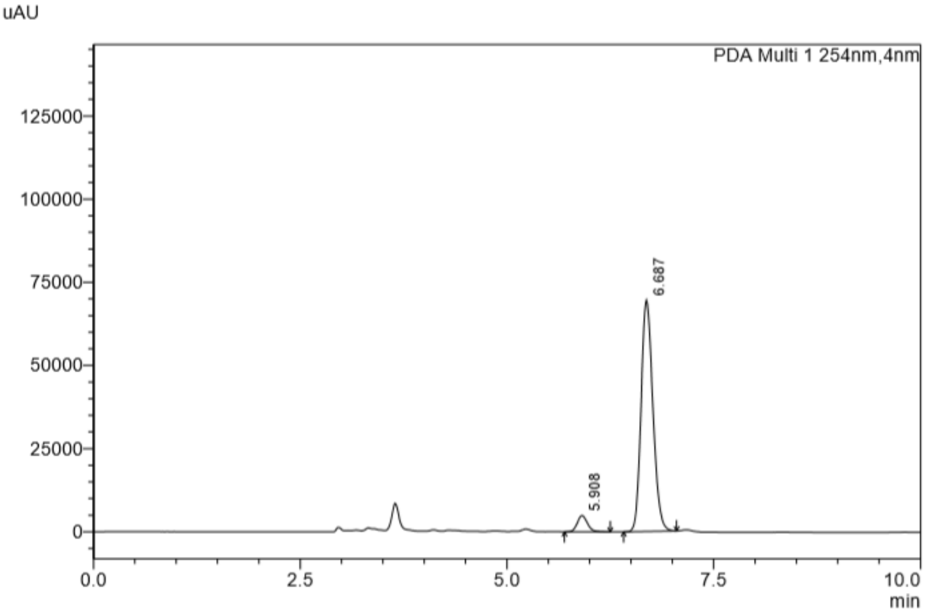

Peak Table

PDA Ch1 254nm

Peak#	Ret. Time	Area	Height	Area%
1	5.908	41677	4879	5.679
2	6.687	692164	69586	94.321
Total		733841	74466	100.000

Supplementary Figure 106 HPLC analysis of **4ba** (racemic, Daicel Chiralpak OD-H column)

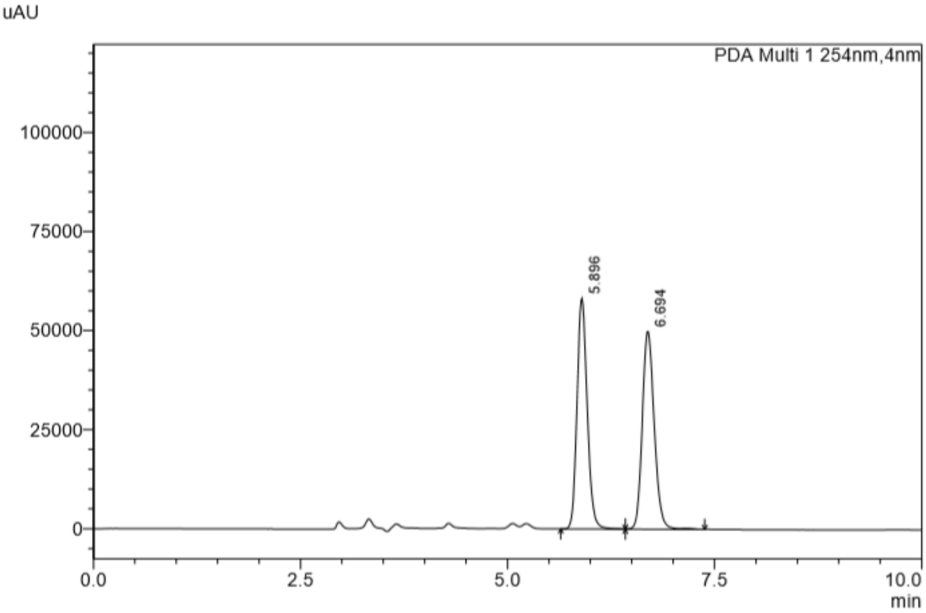

Peak Table

PDA Ch1 254nm

Peak#	Ret. Time	Area	Height	Area%
1	5.896	499603	58210	49.884
2	6.694	501923	49874	50.116
Total		1001526	108085	100.000

Supplementary Figure 107 HPLC analysis of **4ba·HCl** (after recrystallization, Daicel Chiralpak OD-RH column)

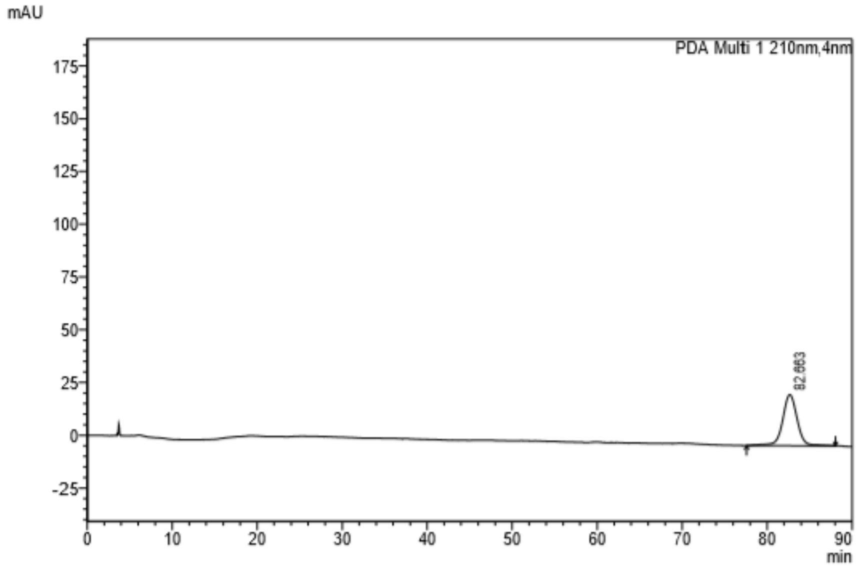

Peak Table

PDA Ch1 210nm

Peak#	Ret. Time	Area	Height	Area%
1	82.663	2873126	24247	100.000
Total		2873126	24247	100.000

Supplementary Figure 108 HPLC analysis of **4ba** (racemic, Daicel Chiralpak OD-RH column)

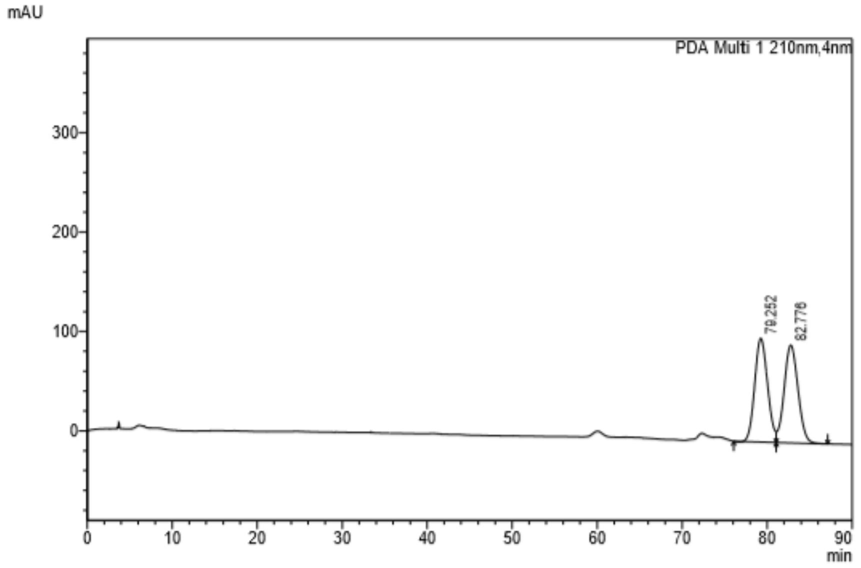

Peak Table

PDA Ch1 210nm

Peak#	Ret. Time	Area	Height	Area%
1	79.252	10976523	104445	49.610
2	82.776	11148950	98506	50.390
Total		22125473	202951	100.000

Supplementary Figure 109 ^{1}H NMR of **4ca**

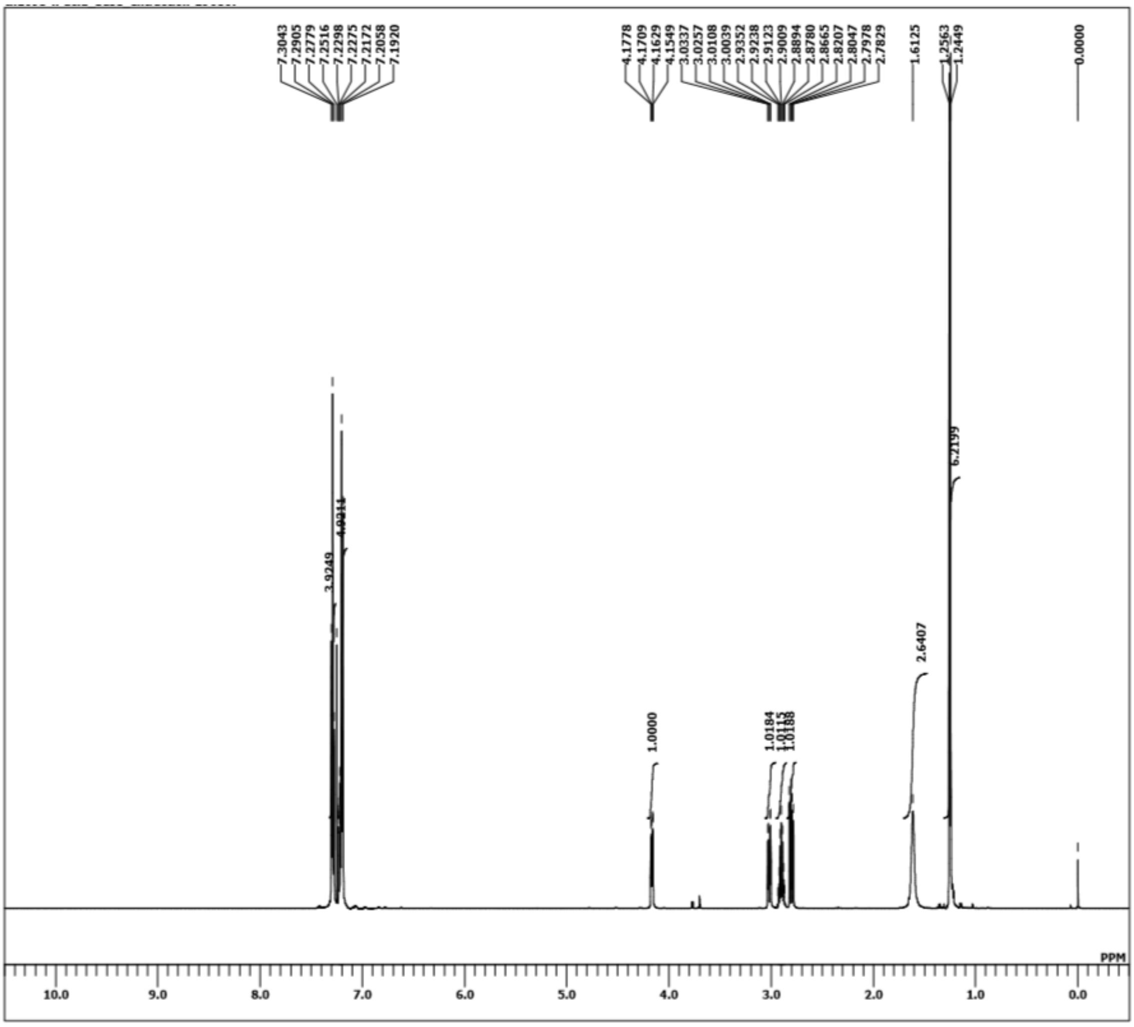

Supplementary Figure 110 ^{13}C NMR of **4ca**

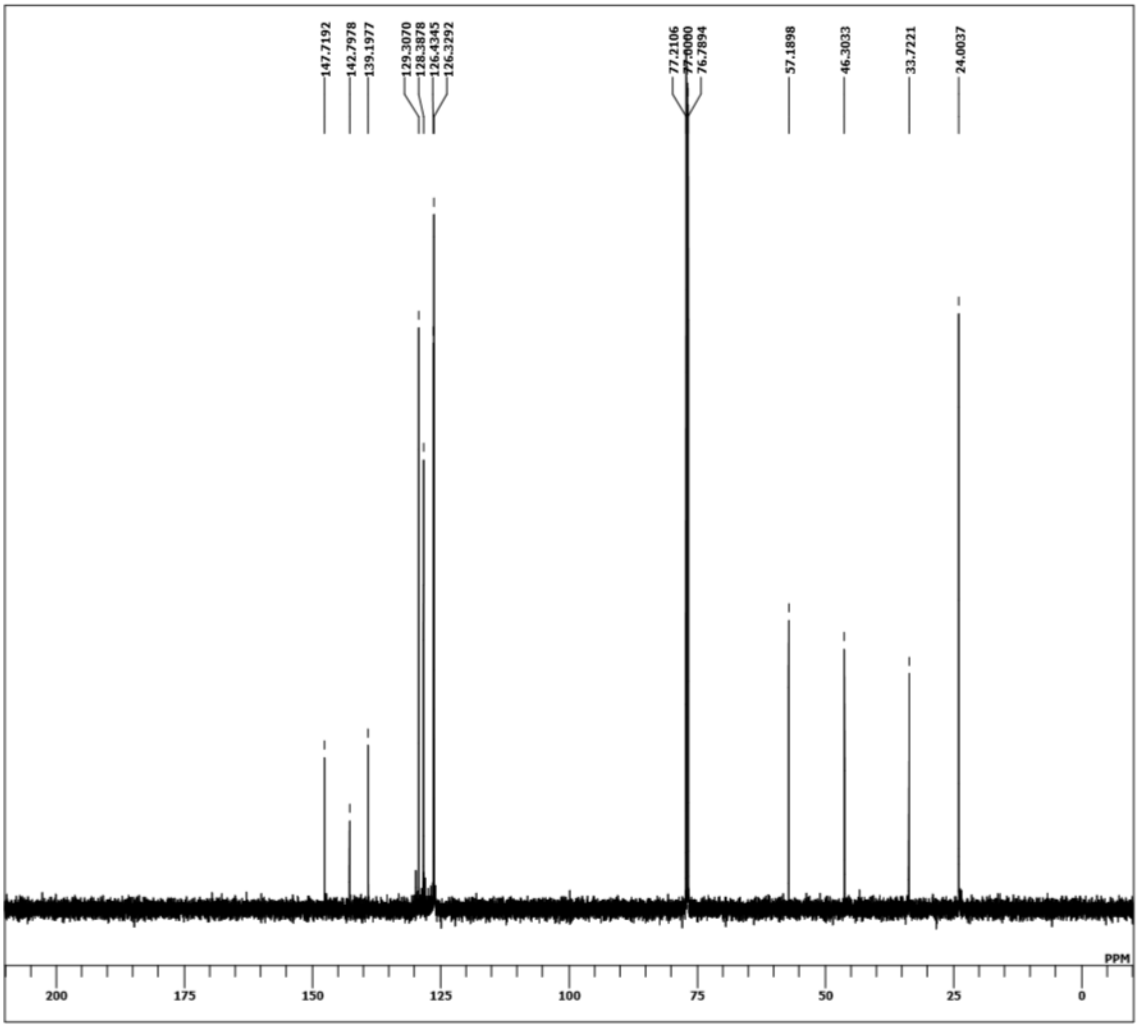

Supplementary Figure 111 HPLC analysis of **4ca** (Daicel Chiralpak OD-H column)

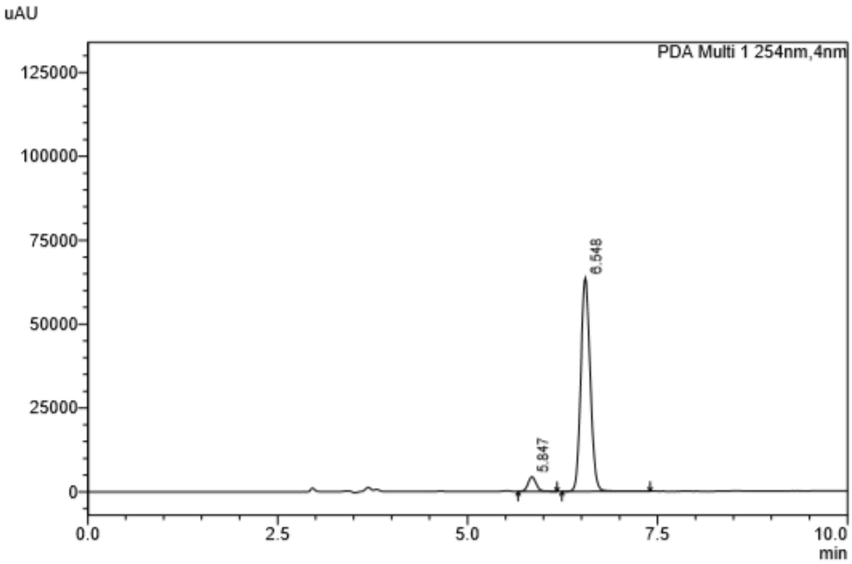

Peak Table

PDA Ch1 254nm

Peak#	Ret. Time	Area	Height	Area%
1	5.847	32147	4342	5.545
2	6.548	547577	63660	94.455
Total		579724	68002	100.000

Supplementary Figure 112 HPLC analysis of **4ca** (racemic, Daicel Chiralpak OD-H column)

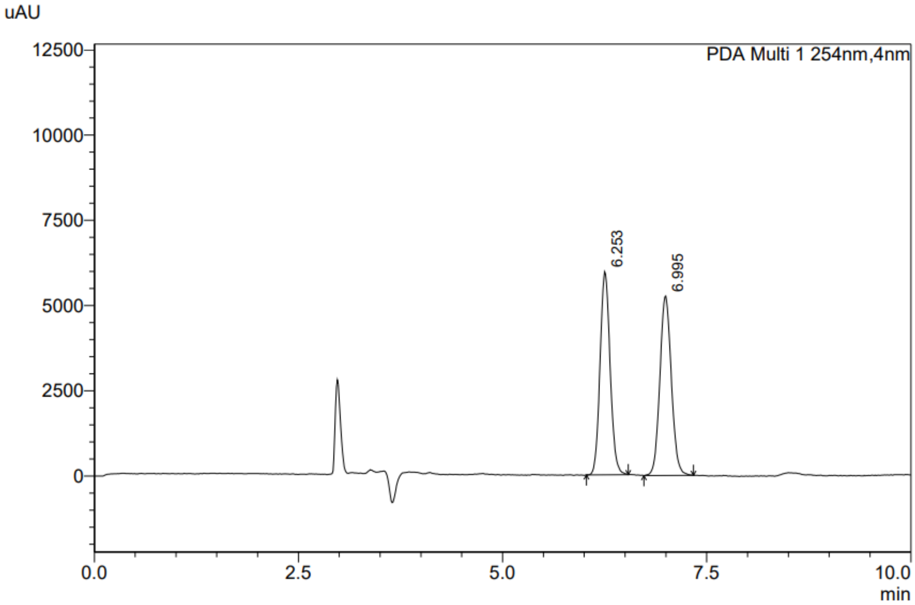

Peak Table

PDA Ch1 254nm

Peak#	Ret. Time	Area	Height	Area%
1	6.253	51035	5957	49.738
2	6.995	51572	5264	50.262
Total		102606	11221	100.000

Supplementary Figure 113 HPLC analysis of **4ca·HCl** (after recrystallization, Daicel Chiralpak OD-RH column)

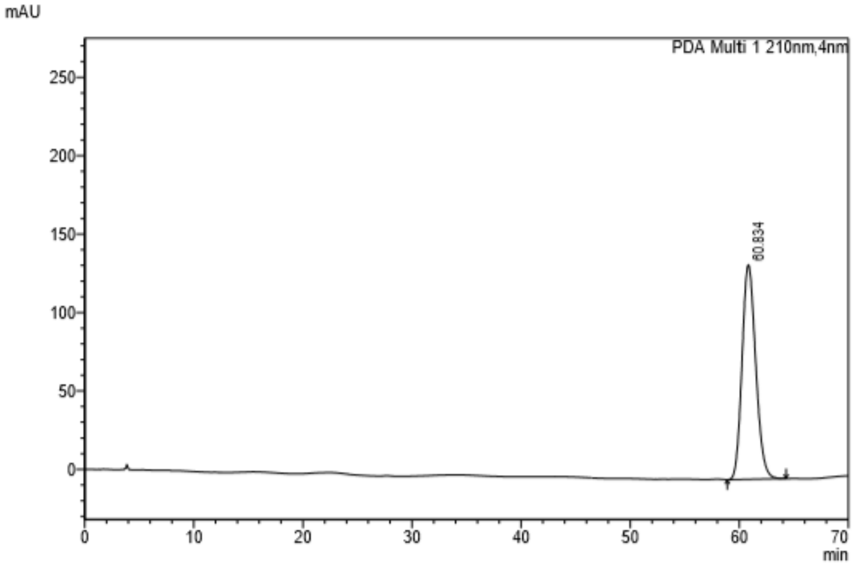

Peak Table

PDA Ch1 210nm

Peak#	Ret. Time	Area	Height	Area%
1	60.834	11945141	136781	100.000
ΊŒv		11945141	136781	100.000

Supplementary Figure 114 HPLC analysis of **4ca·HCl** (racemic, Daicel Chiralpak OD-RH column)

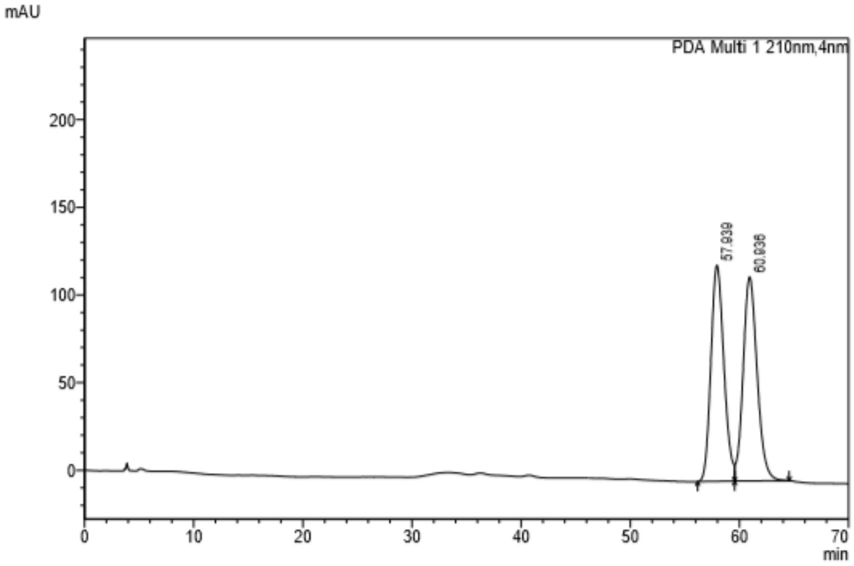

Peak Table

PDA Ch1 210nm

Peak#	Ret. Time	Area	Height	Area%
1	57.939	10199215	123266	49.523
2	60.936	10395771	116517	50.477
ΊŒv		20594986	239783	100.000

Supplementary Figure 115 ^{1}H NMR of **4da**

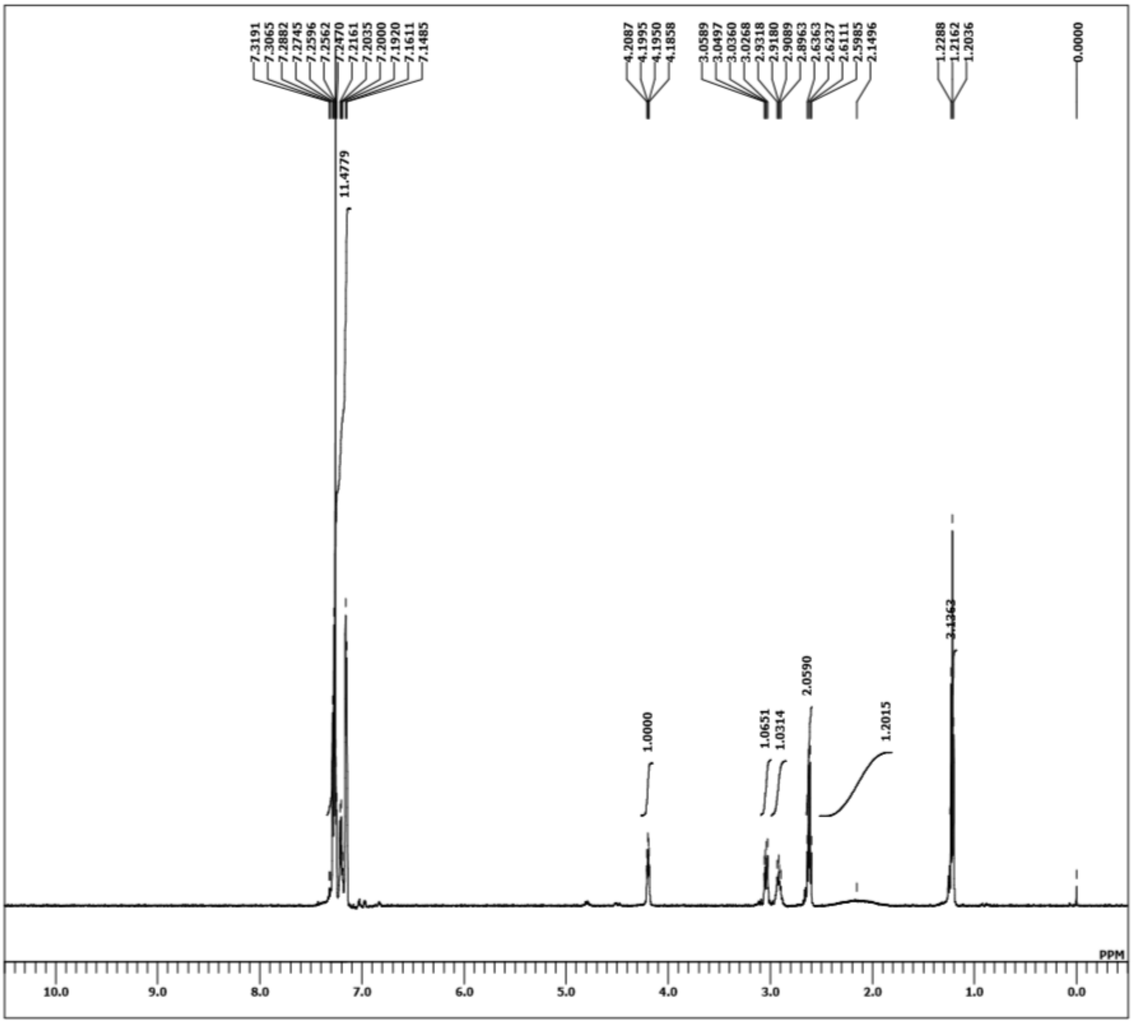

Supplementary Figure 116 ^{13}C NMR of **4da**

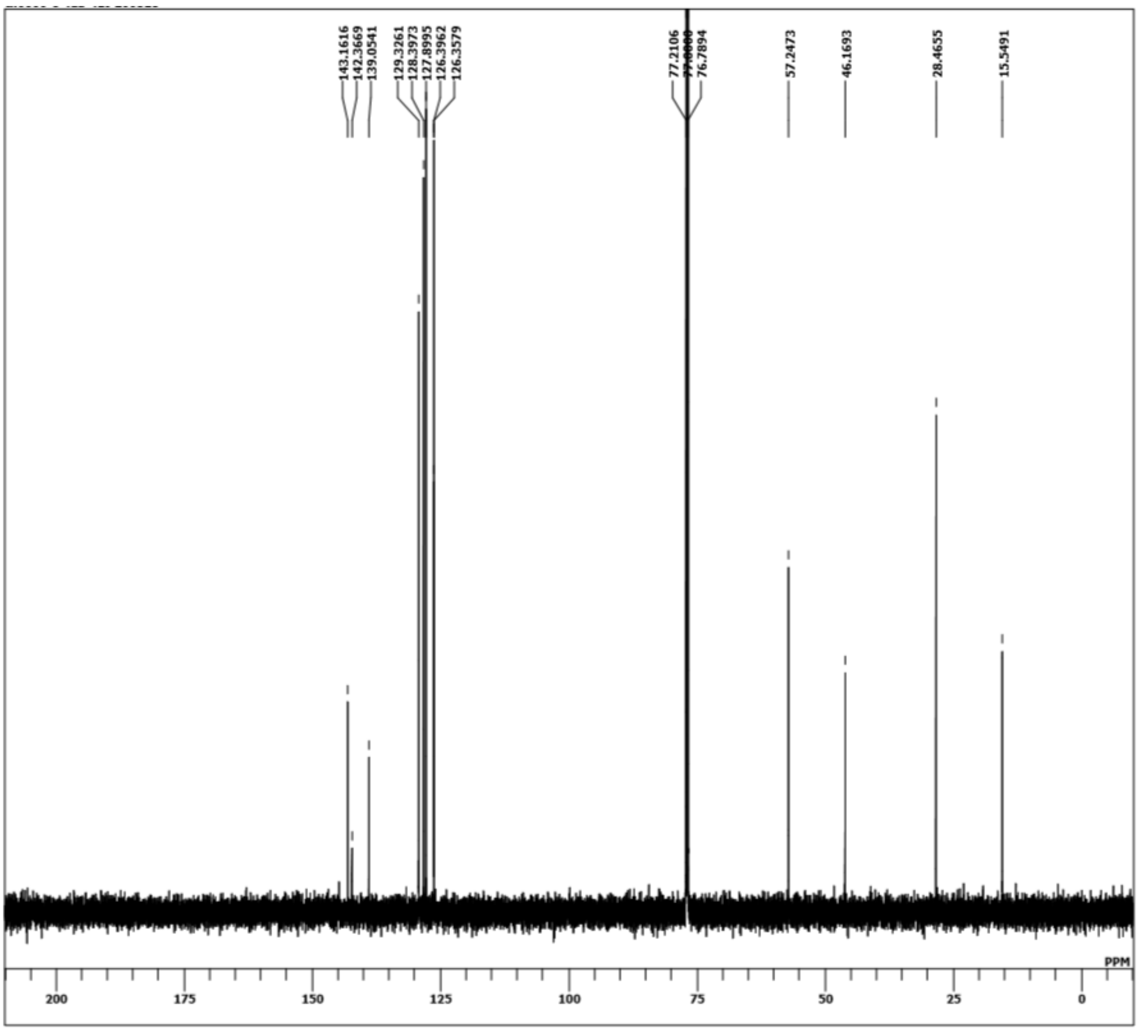

Supplementary Figure 117 HPLC analysis of **4da**

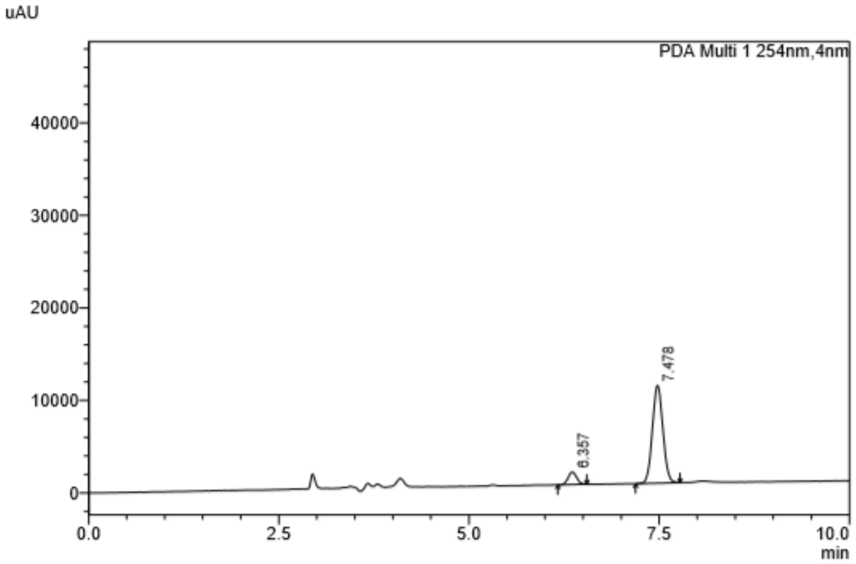

Peak Table

PDA Ch1 254nm

Peak#	Ret. Time	Area	Height	Area%
1	6.357	10701	1356	9.509
2	7.478	101839	10561	90.491
Total		112540	11916	100.000

Supplementary Figure 118 HPLC analysis of **4da** (racemic)

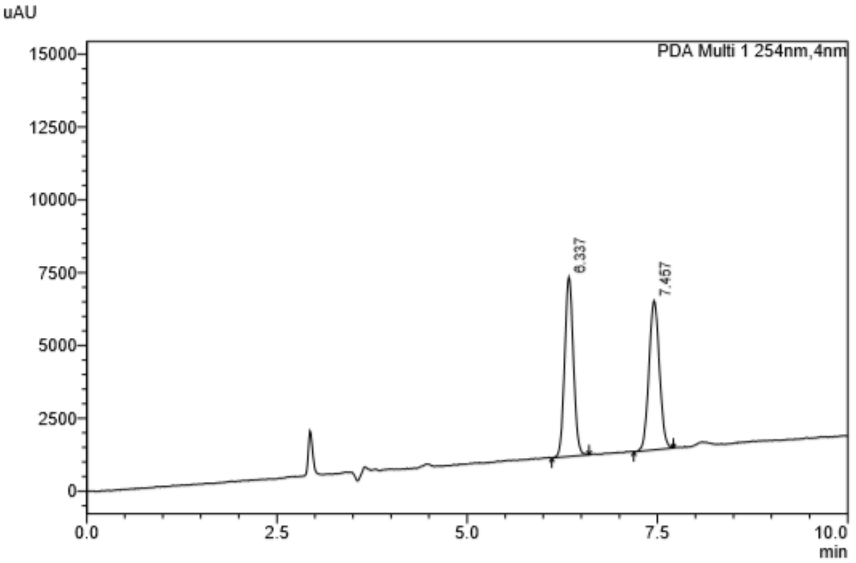

Peak Table

PDA Ch1 254nm

Peak#	Ret. Time	Area	Height	Area%
1	6.337	49518	6164	50.121
2	7.457	49279	5126	49.879
Total		98797	11290	100.000

Supplementary Figure 119 ^{1}H NMR of **4ha**

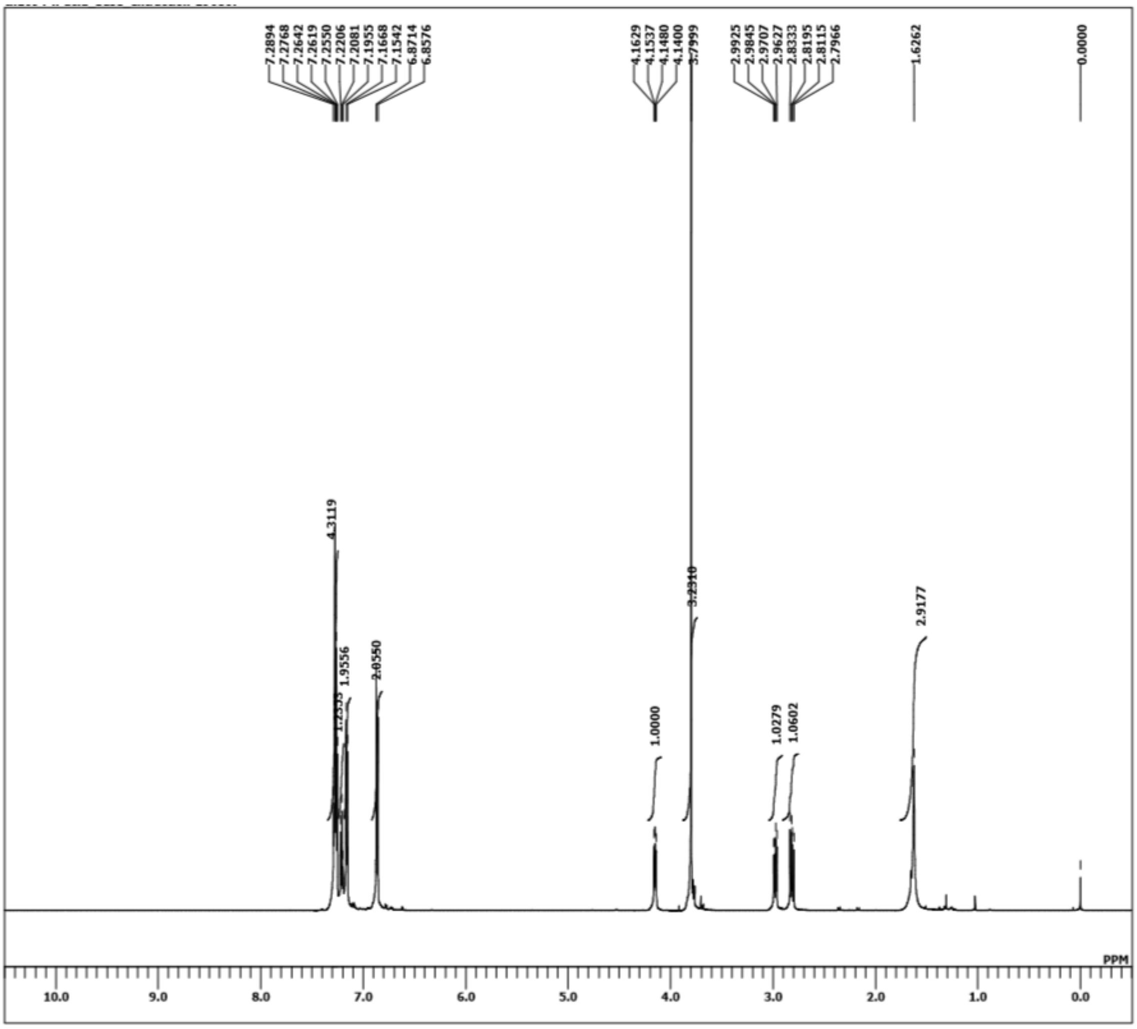

Supplementary Figure 120 ^{13}C NMR of **4ha**

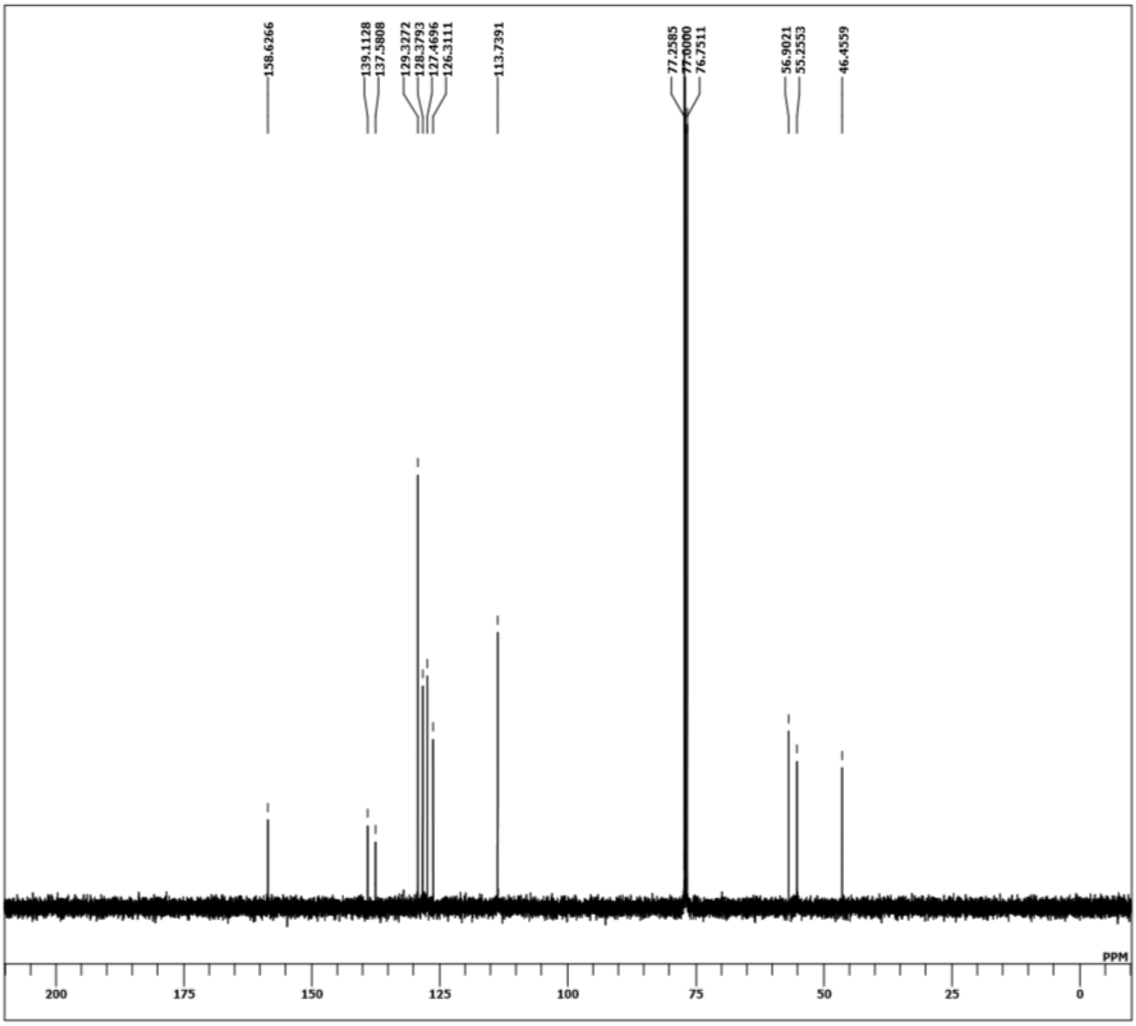

Supplementary Figure 122 HPLC analysis of **4ha**

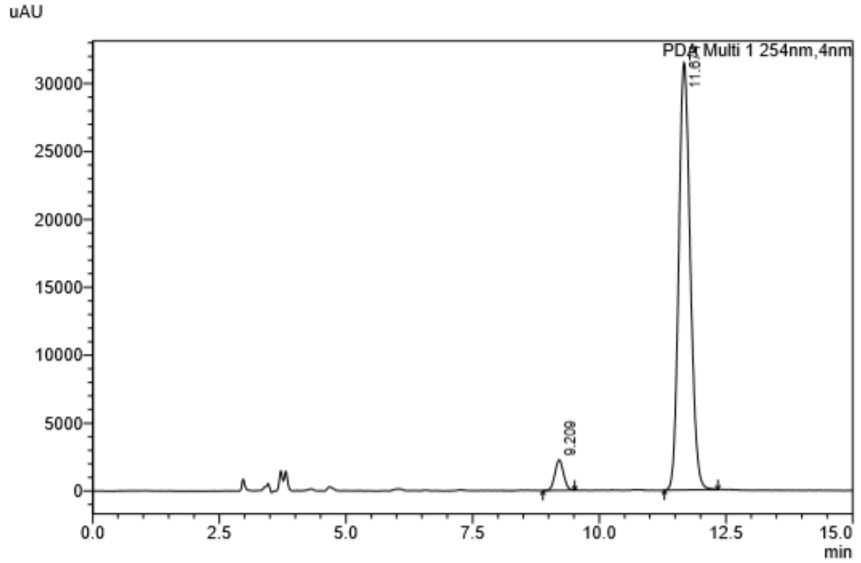

Peak Table

PDA Ch1 254nm

Peak#	Ret. Time	Area	Height	Area%
1	9.209	26961	2249	5.140
2	11.674	497590	31483	94.860
Total		524551	33732	100.000

Supplementary Figure 123 HPLC analysis of **4ha** (racemic)

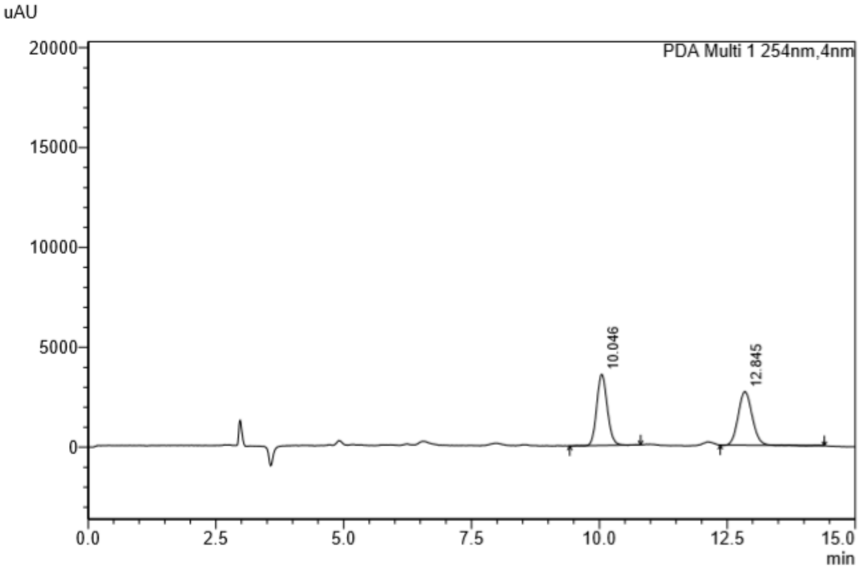

Peak Table

PDA Ch1 254nm

Peak#	Ret. Time	Area	Height	Area%
1	10.046	50602	3555	49.632
2	12.845	51354	2689	50.368
Total		101956	6245	100.000

Supplementary Figure 124 ^{1}H NMR of **4ia**

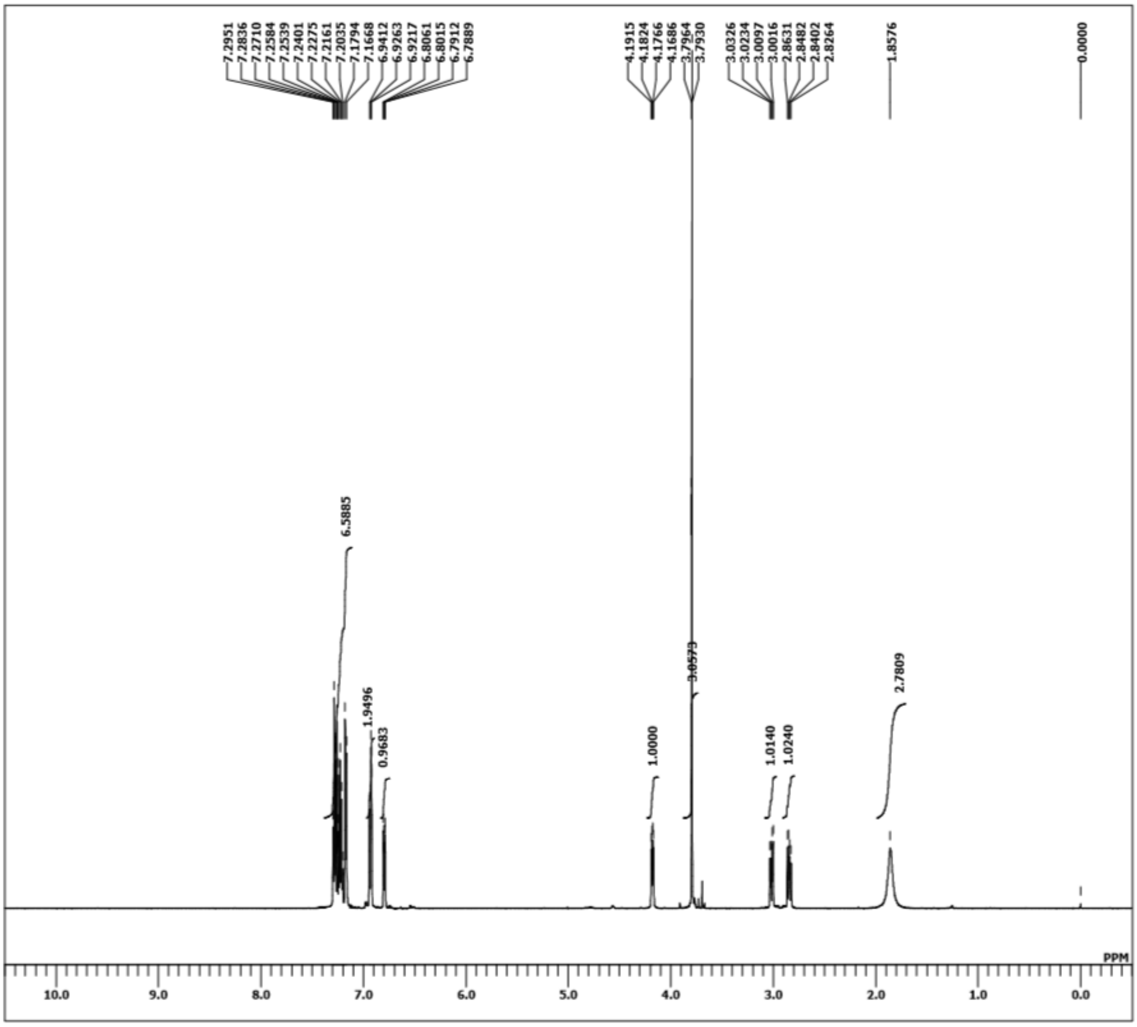

Supplementary Figure 125 ^{13}C NMR of **4ia**

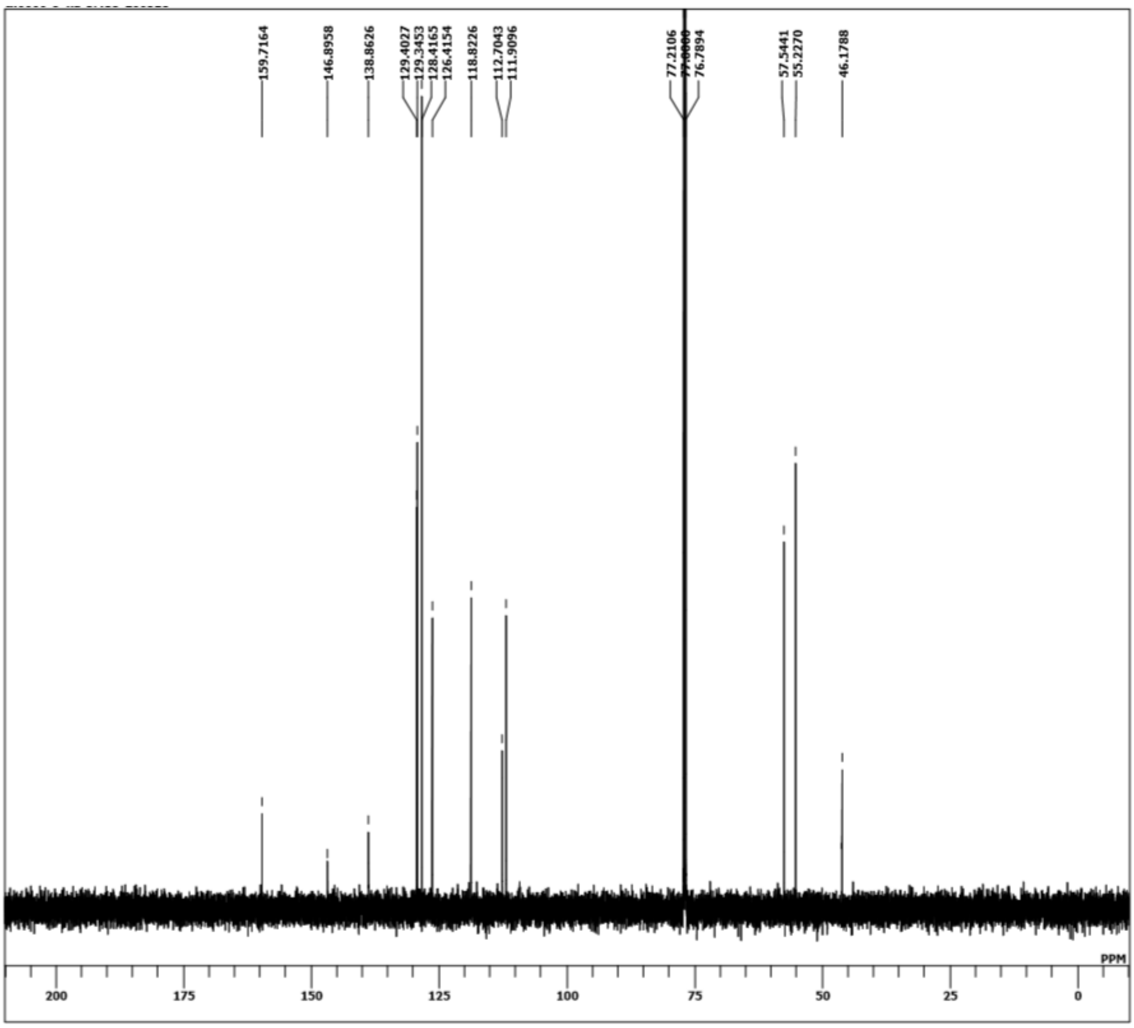

Supplementary Figure 126 HPLC analysis of **4ia**

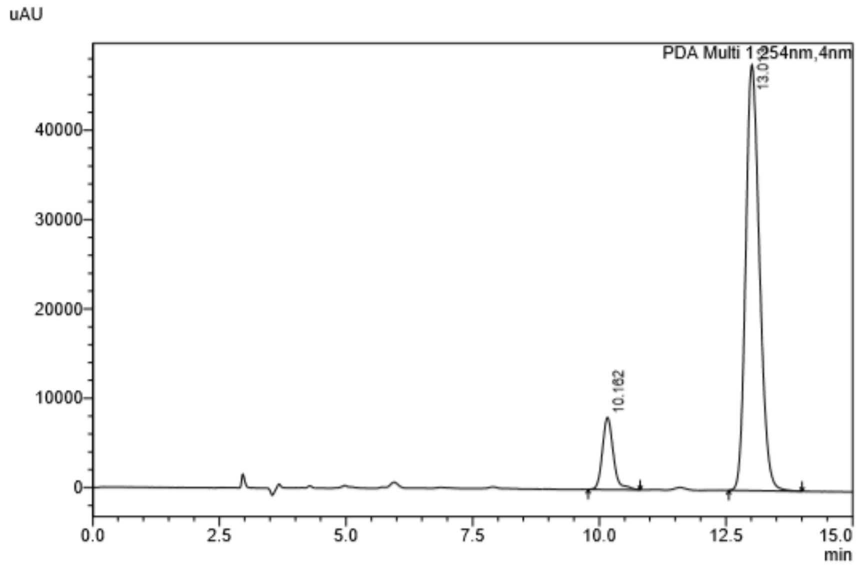

Peak Table

PDA Ch1 254nm

Peak#	Ret. Time	Area	Height	Area%
1	10.162	121239	8060	11.819
2	13.013	904531	47668	88.181
Total		1025770	55728	100.000

Supplementary Figure 127 HPLC analysis of **4ia** (racemic)

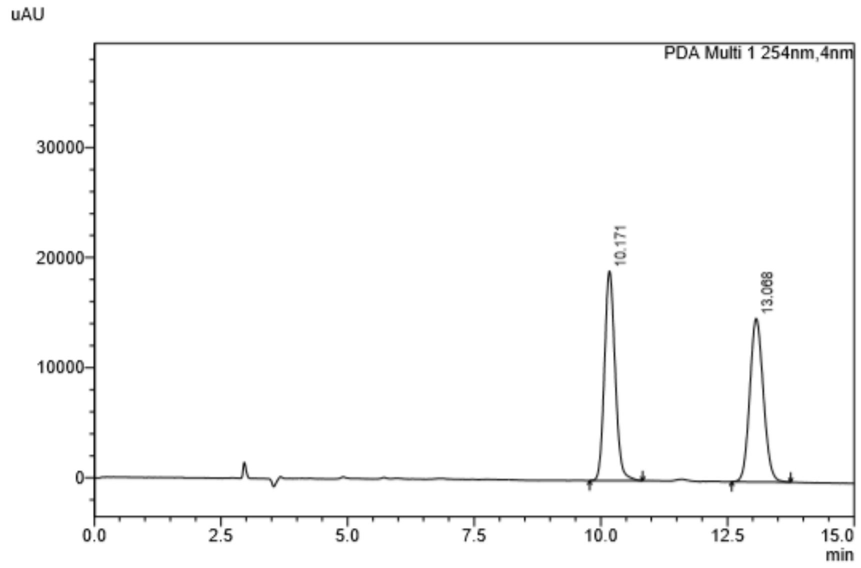

Peak Table

PDA Ch1 254nm

Peak#	Ret. Time	Area	Height	Area%
1	10.171	278969	19002	50.179
2	13.068	276980	14836	49.821
Total		555949	33837	100.000

Supplementary Figure 128 HPLC analysis of **4ia** (after recrystallization)

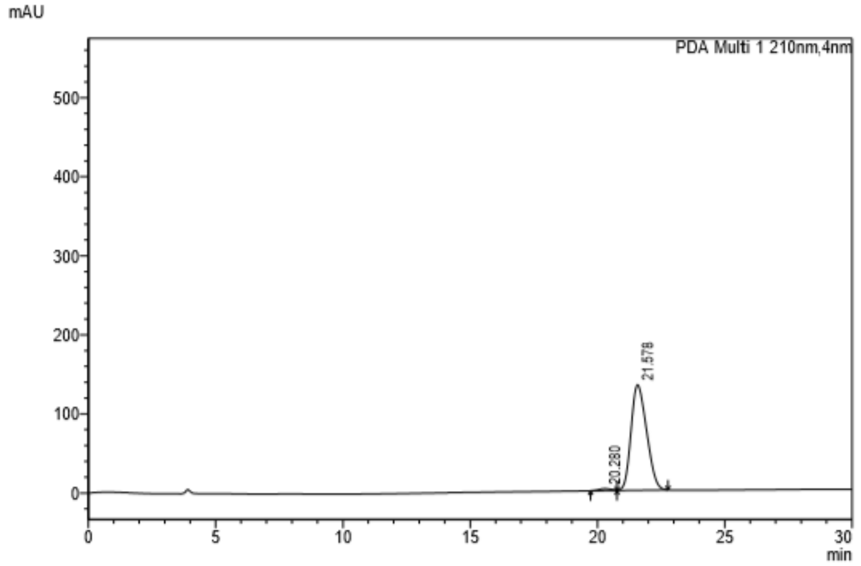

Peak Table

PDA Ch1 210nm

Peak#	Ret. Time	Area	Height	Area%
1	20.280	89973	2455	1.551
2	21.578	5710810	133374	98.449
±Œv		5800783	135828	100.000

Supplementary Figure 129 HPLC analysis of **4ia** (racemic)

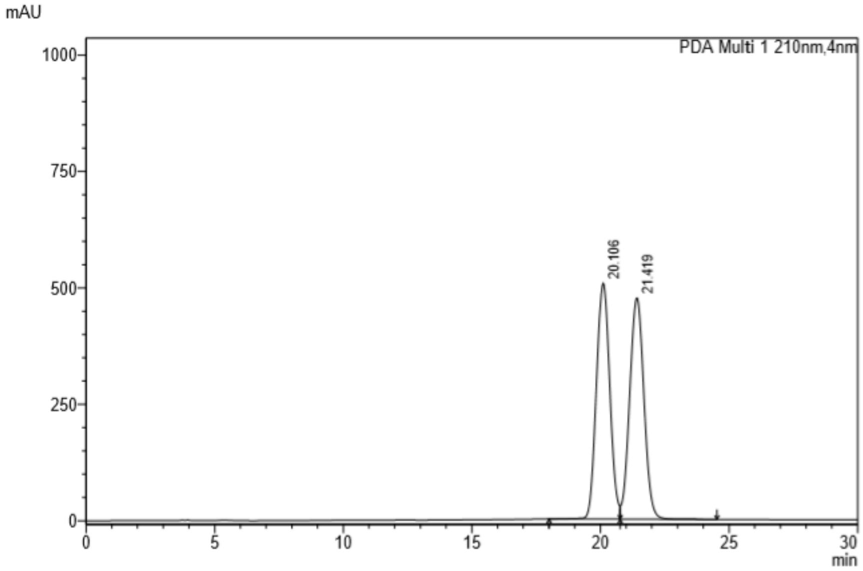

Peak Table

PDA Ch1 210nm

Peak#	Ret. Time	Area	Height	Area%
1	20.106	18264617	506543	49.748
2	21.419	18449834	474963	50.252
±Œv		36714451	981506	100.000

Supplementary Figure 130 ^{1}H NMR of **4ja**

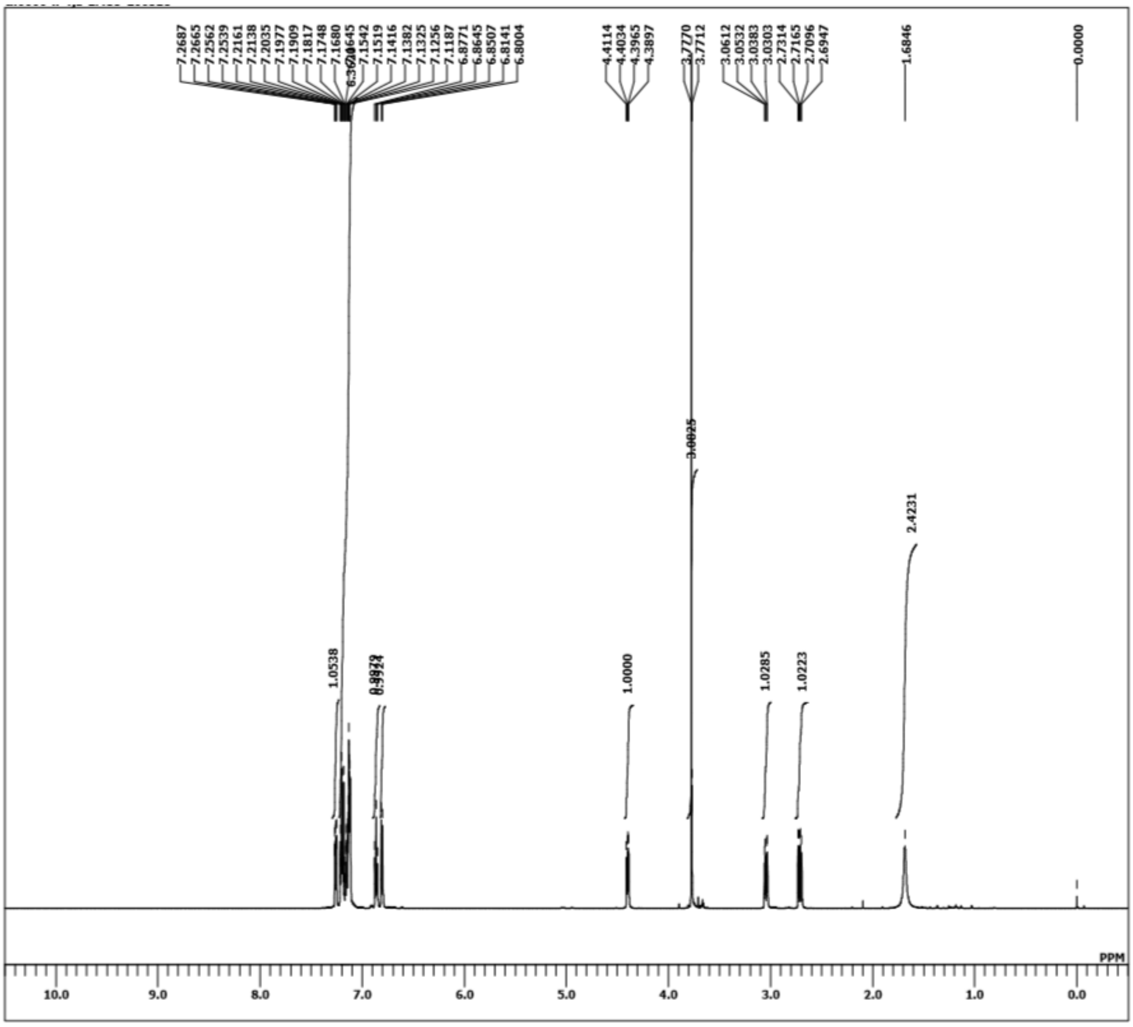

Supplementary Figure 131 ^{13}C NMR of **4ja**

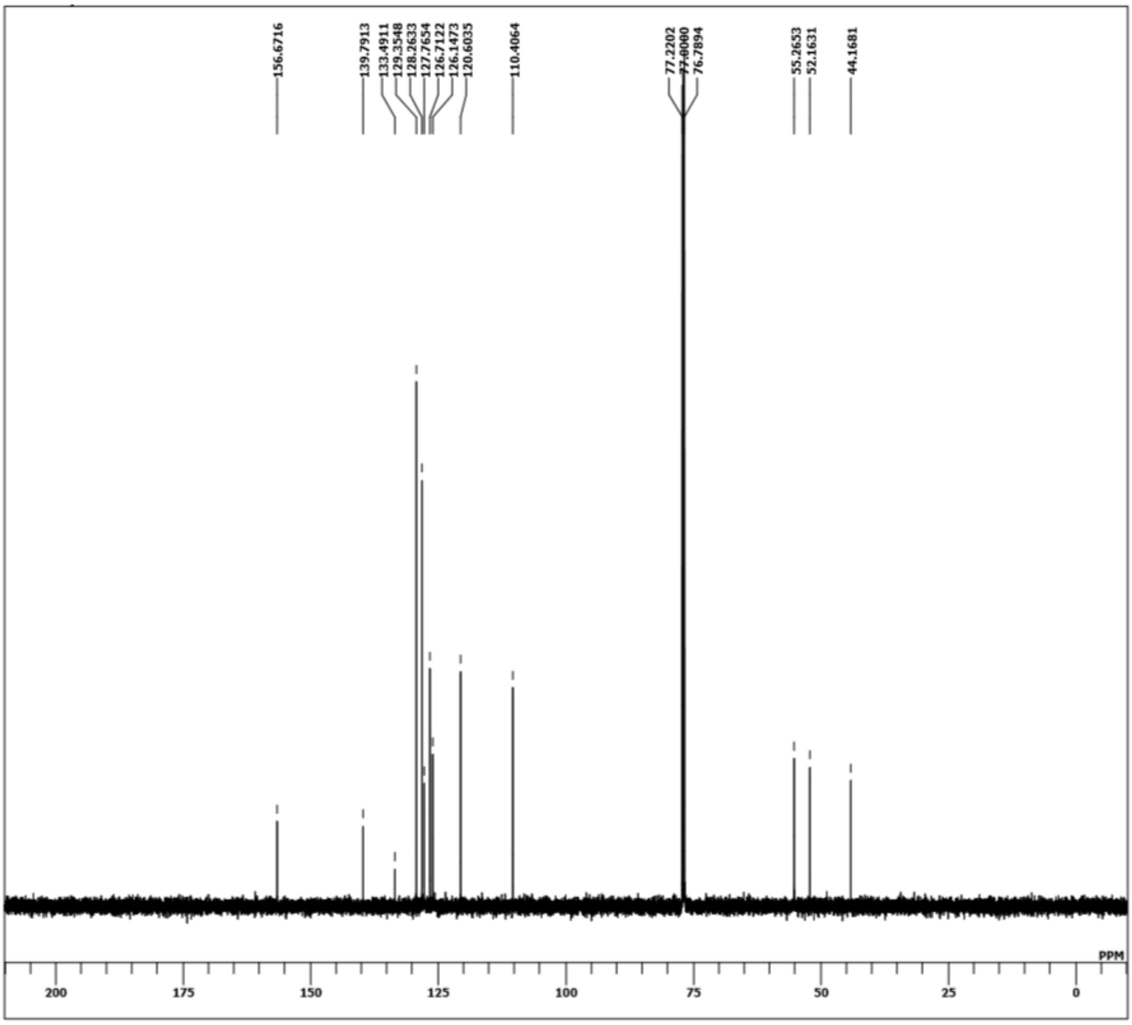

Supplementary Figure 132 HPLC analysis of **4ja**

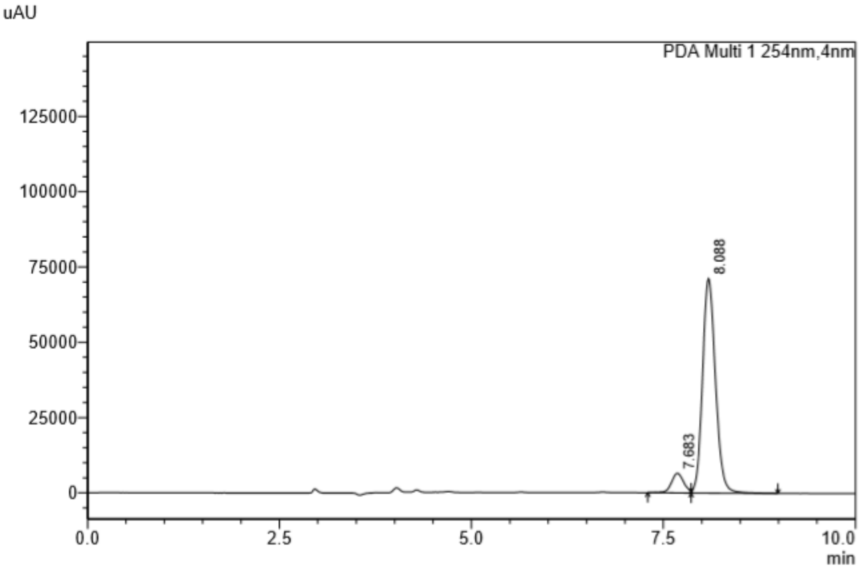

Peak Table

PDA Ch1 254nm

Peak#	Ret. Time	Area	Height	Area%
1	7.683	72136	6477	7.961
2	8.088	834004	71260	92.039
Total		906140	77737	100.000

Supplementary Figure 133 HPLC analysis of **4ja** (after recrystallization)

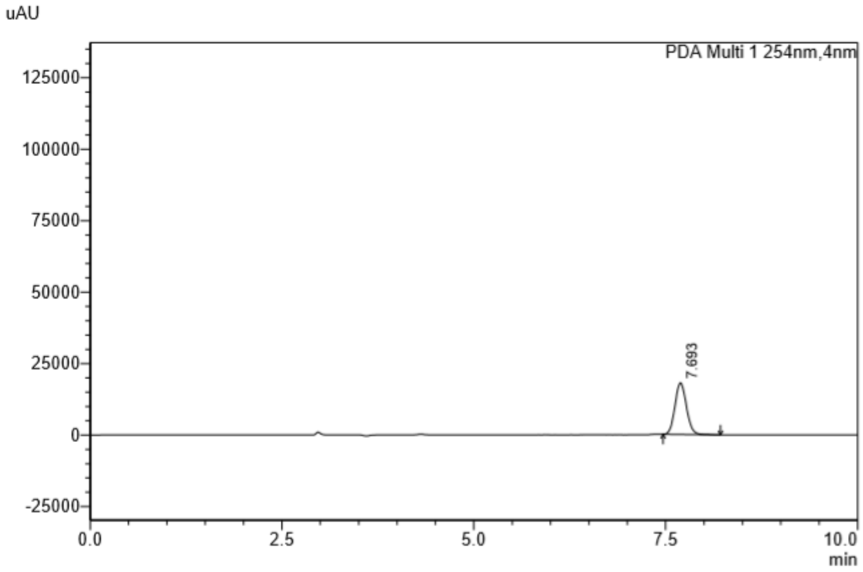

Peak Table

PDA Ch1 254nm

Peak#	Ret. Time	Area	Height	Area%
1	7.693	188464	18072	100.000
Total		188464	18072	100.000

Supplementary Figure 134 HPLC analysis of **4ja** (racemic)

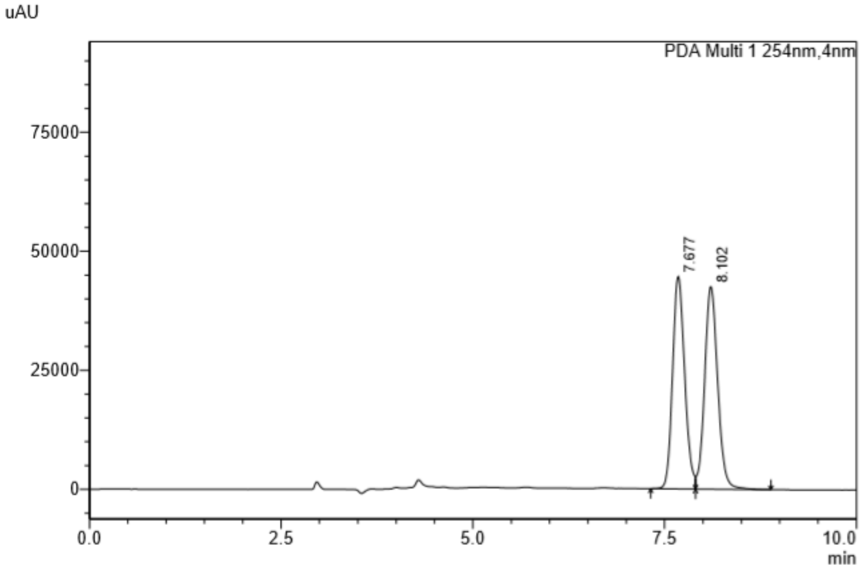

Peak Table

PDA Ch1 254nm

Peak#	Ret. Time	Area	Height	Area%
1	7.677	489374	44622	49.316
2	8.102	502955	42533	50.684
Total		992329	87155	100.000

Supplementary Figure 135 ^{1}H NMR of **4ka**

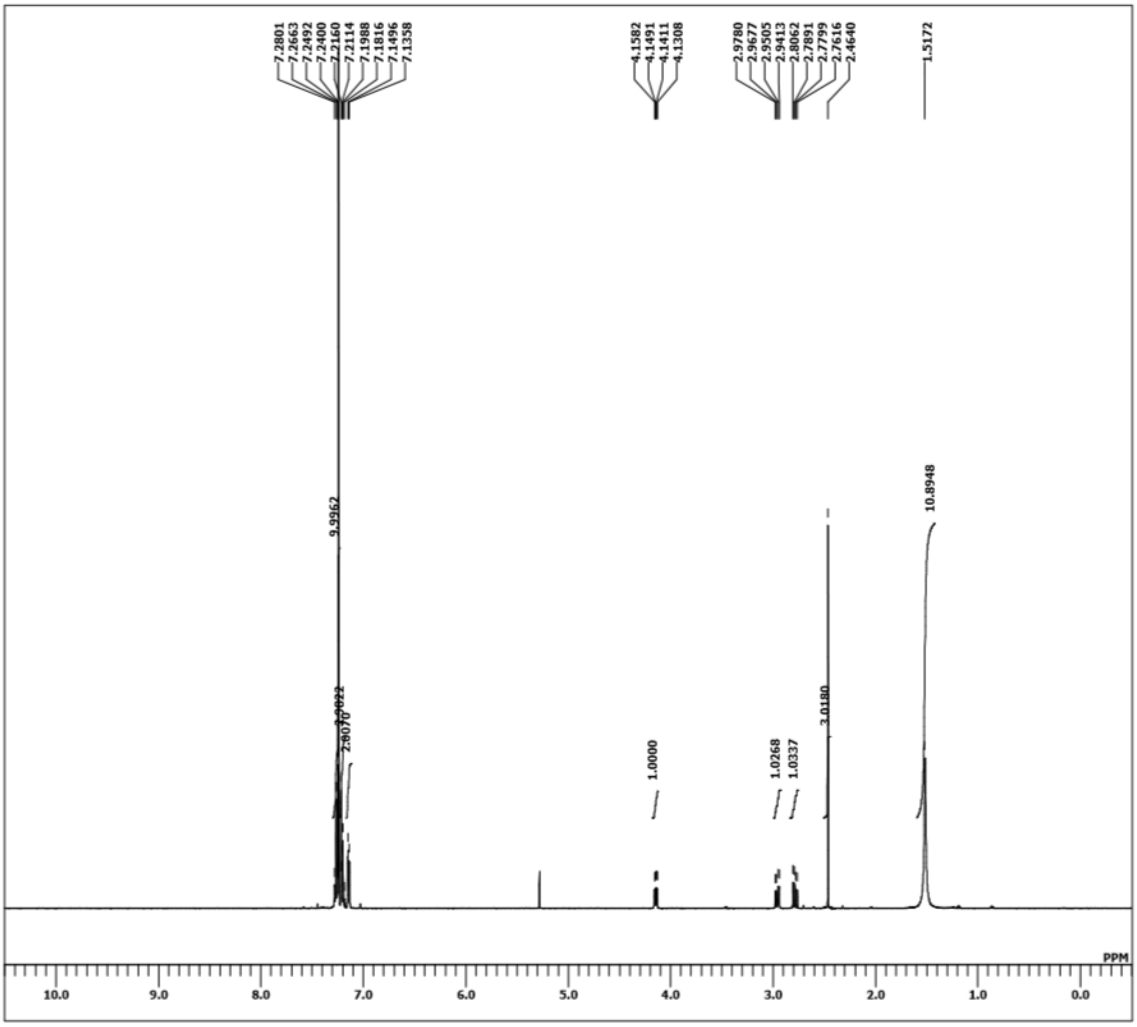

Supplementary Figure 136 ^{13}C NMR of **4ka**

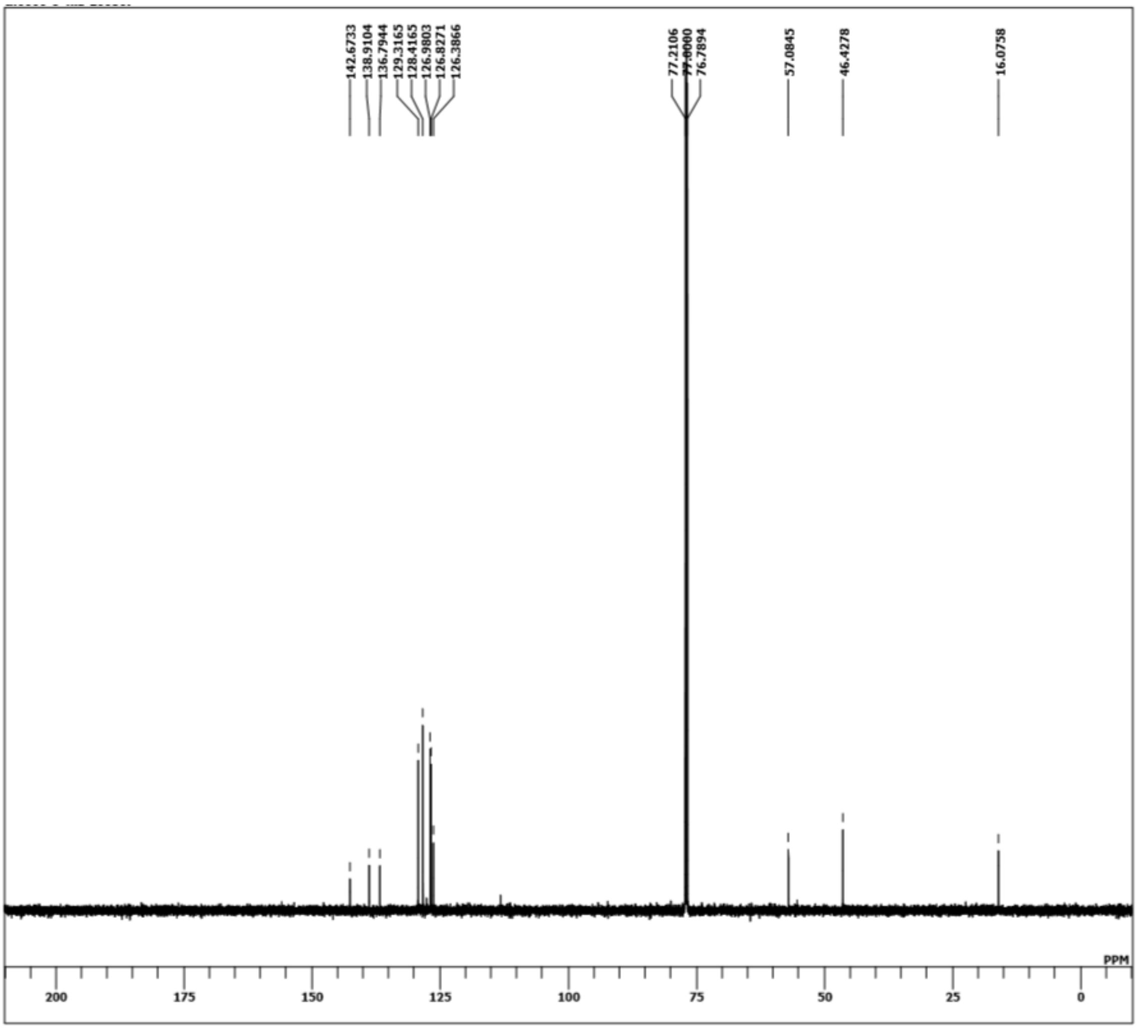

Supplementary Figure 137 HPLC analysis of **4ka**

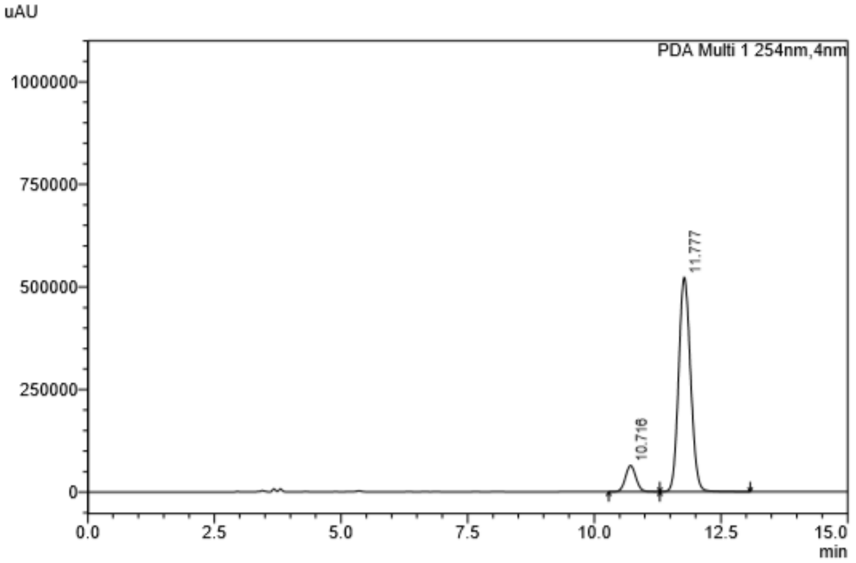

Peak Table

PDA Ch1 254nm

Peak#	Ret. Time	Area	Height	Area%
1	10.716	934128	63999	9.815
2	11.777	8582873	522895	90.185
Total		9517001	586894	100.000

Supplementary Figure 138 HPLC analysis of **4ka** (racemic)

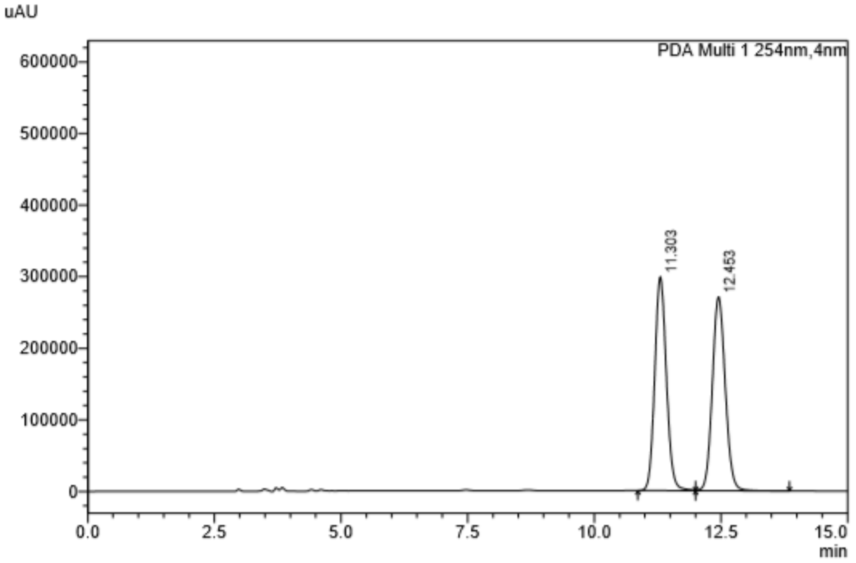

Peak Table

PDA Ch1 254nm

Peak#	Ret. Time	Area	Height	Area%
1	11.303	4788694	298512	49.816
2	12.453	4824012	270699	50.184
Total		9612706	569212	100.000

Supplementary Figure 139 ^{1}H NMR of **4ab**

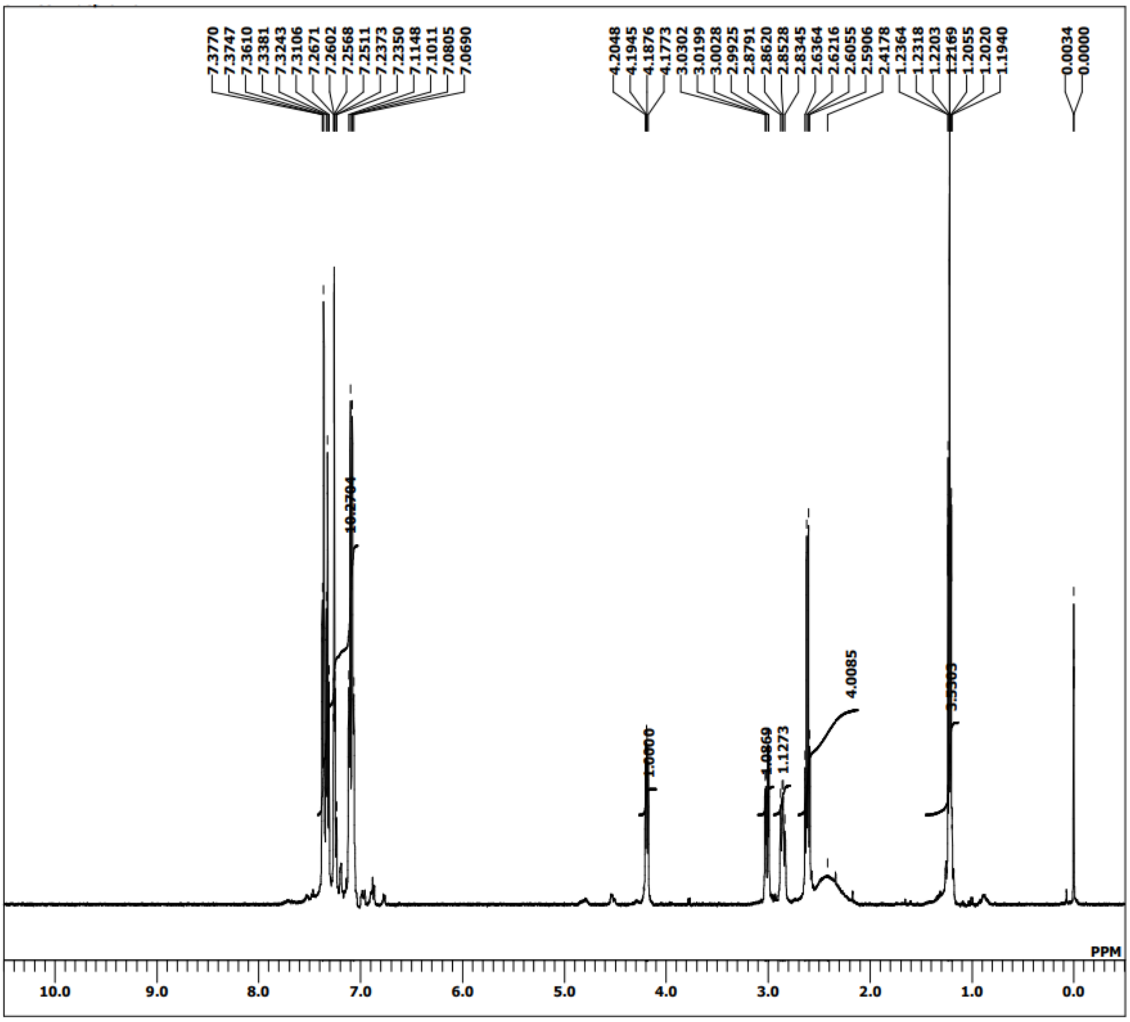

Supplementary Figure 140 ^{13}C NMR of **4ab**

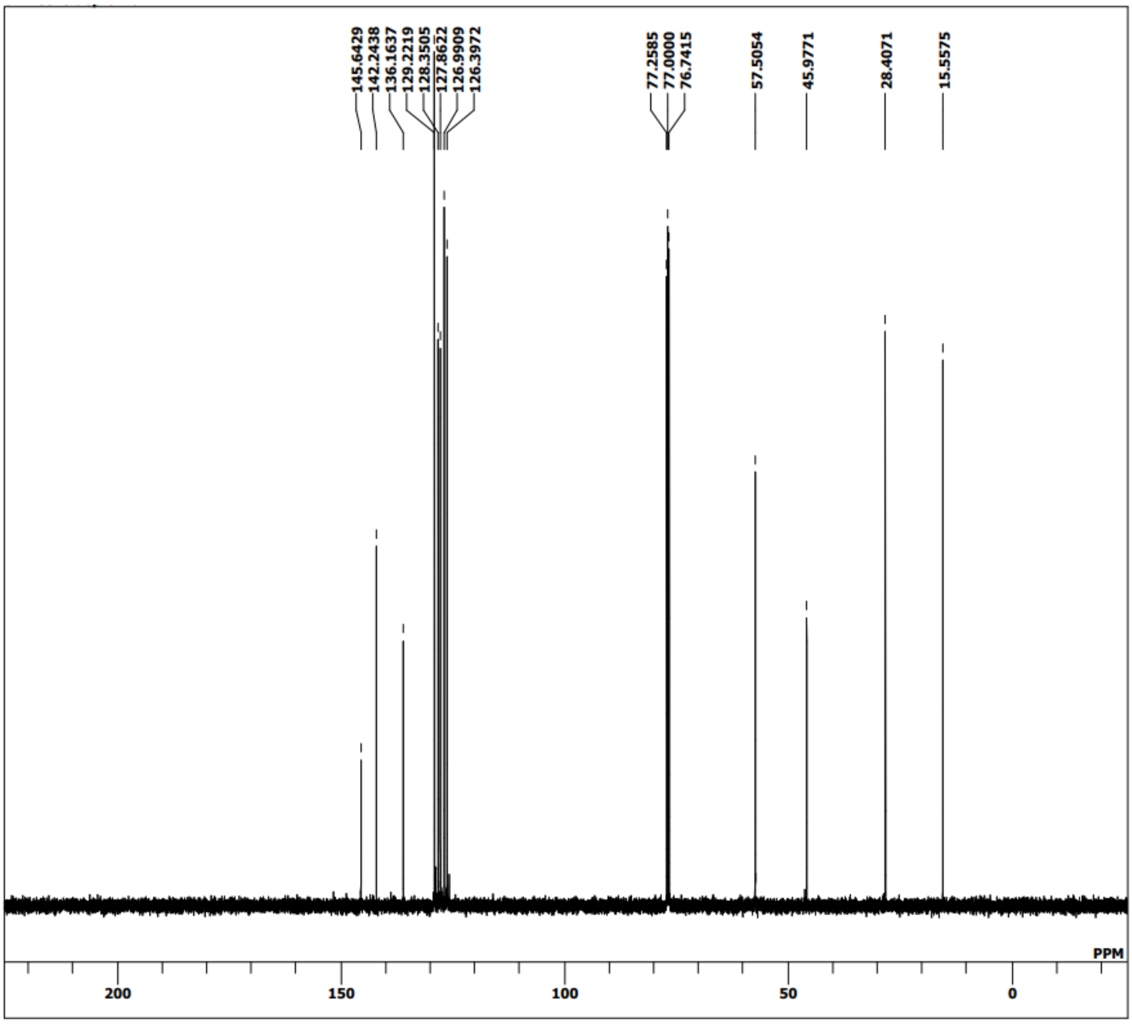

Supplementary Figure 141 HPLC analysis of **4ab**

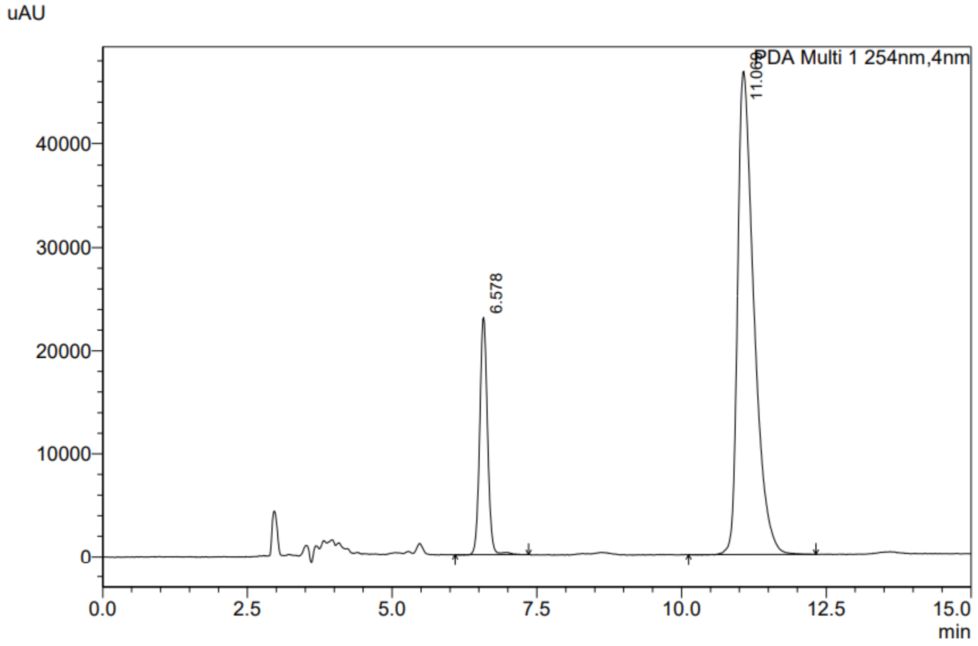

Peak Table

PDA Ch1 254nm

Peak#	Ret. Time	Area	Height	Area%
1	6.578	215221	22984	18.964
2	11.069	919662	46736	81.036
Total		1134883	69720	100.000

Supplementary Figure 142 HPLC analysis of **4ab** (racemic)

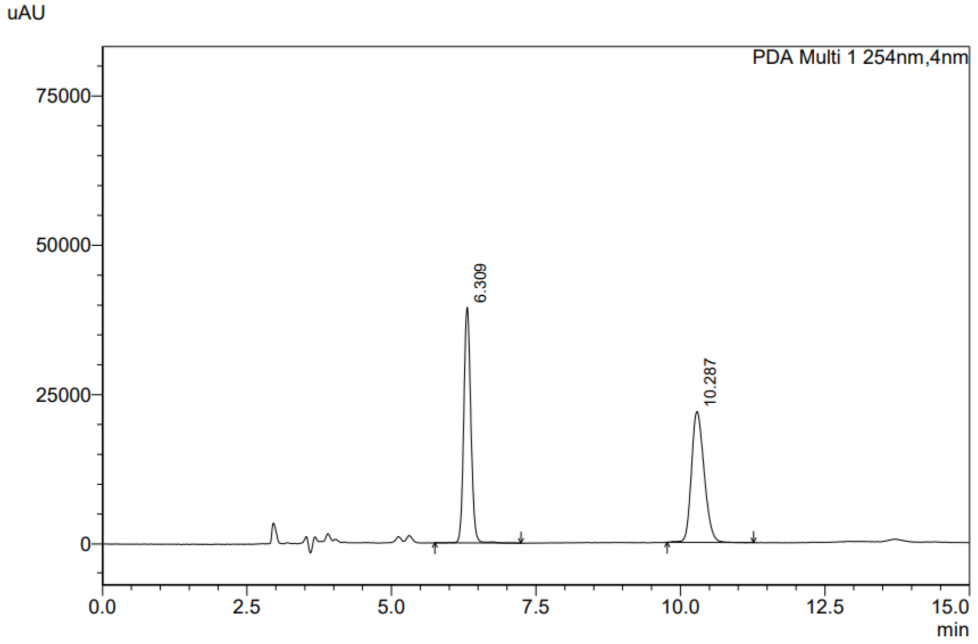

Peak Table

PDA Ch1 254nm

Peak#	Ret. Time	Area	Height	Area%
1	6.309	333715	39396	49.939
2	10.287	334524	21889	50.061
Total		668238	61285	100.000

Supplementary Figure 143 ^{1}H NMR of **4ae**

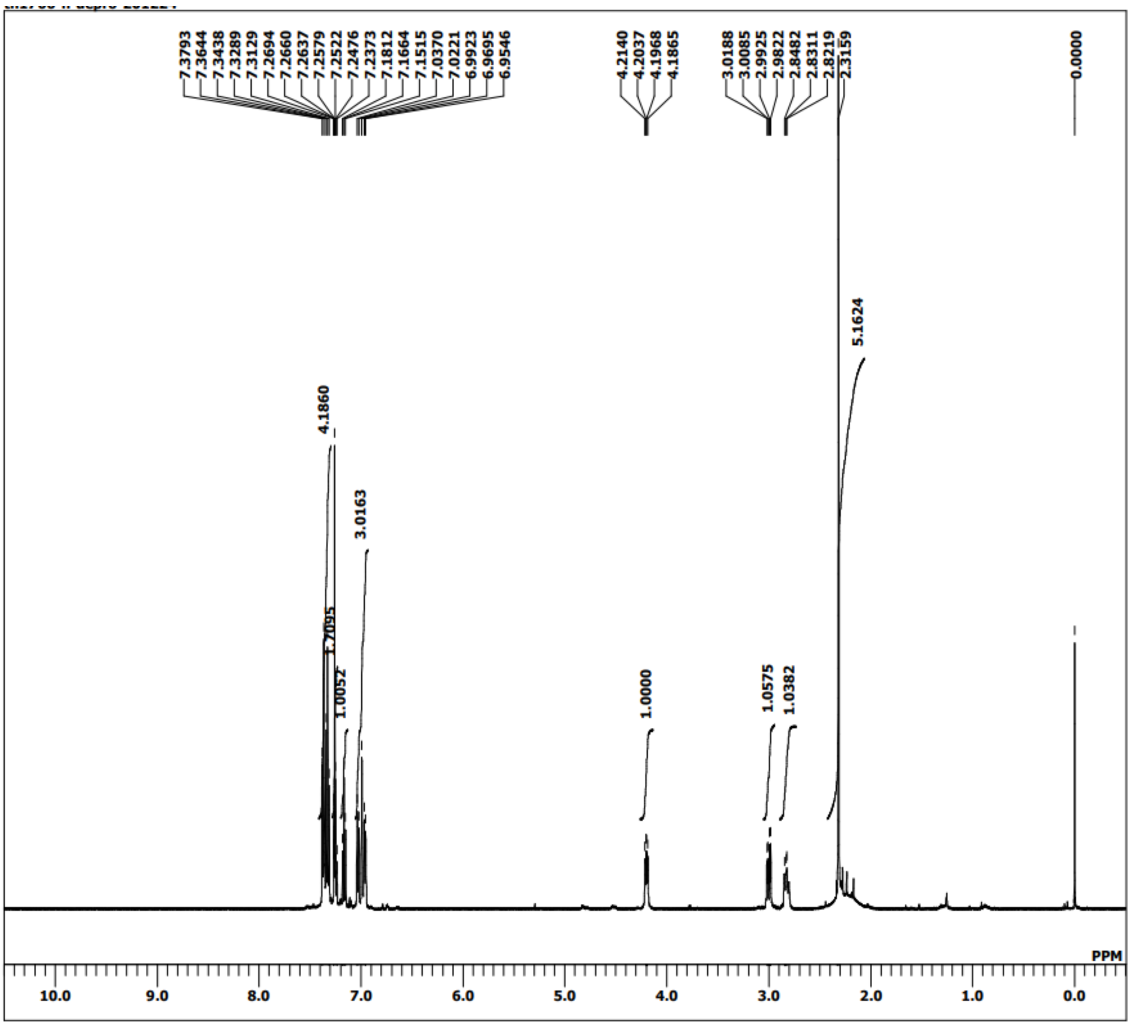

Supplementary Figure 144 ^{13}C NMR of **4ae**

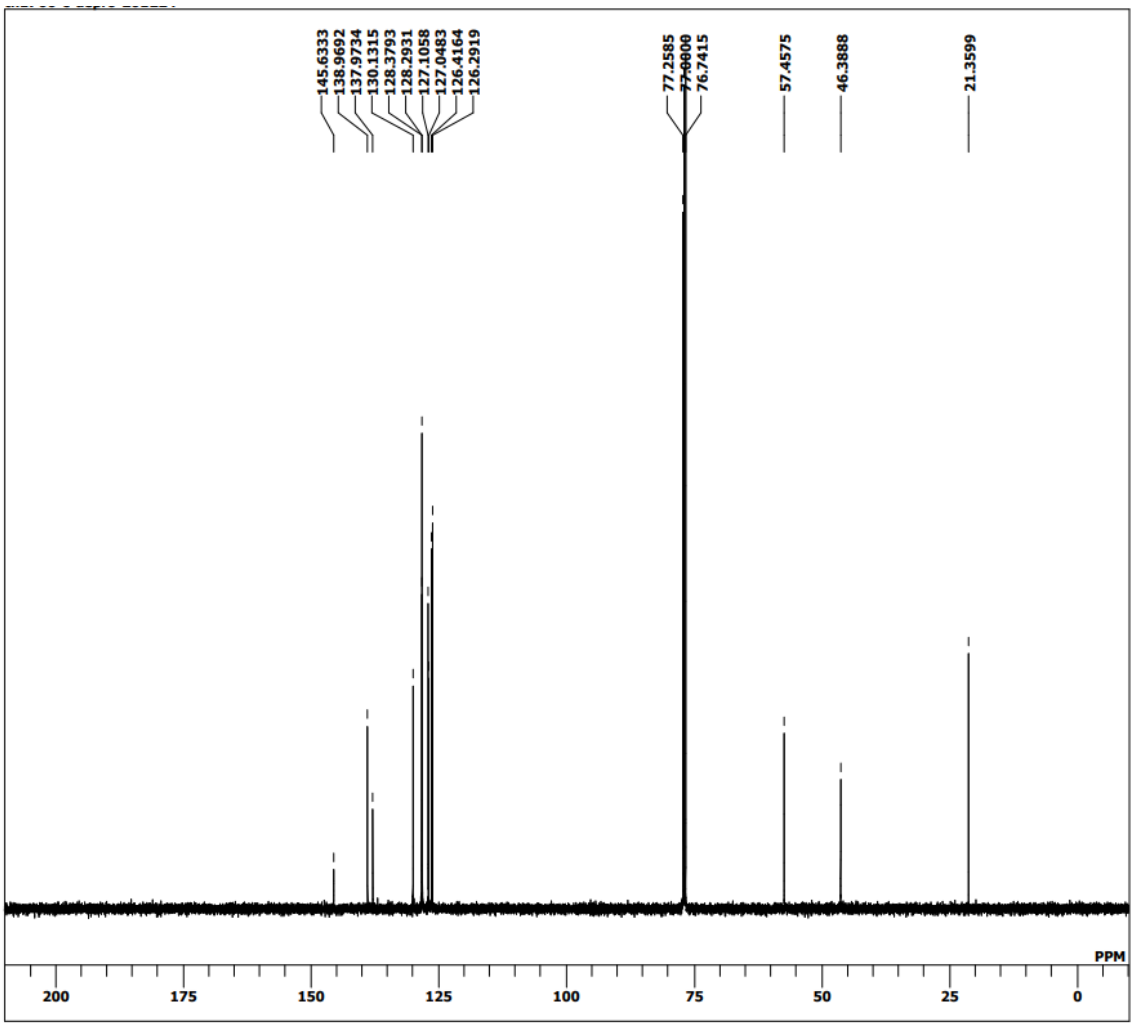

Supplementary Figure 145 HPLC analysis of **4ae**

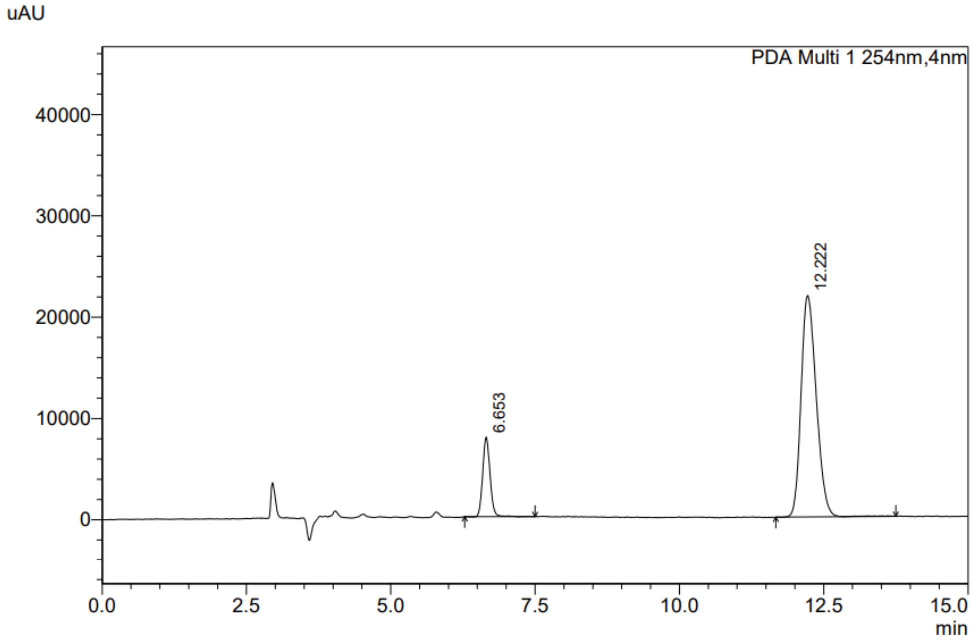

Peak Table

PDA Ch1 254nm

Peak#	Ret. Time	Area	Height	Area%
1	6.653	69122	7819	14.735
2	12.222	399990	21849	85.265
Total		469112	29668	100.000

Supplementary Figure 146 HPLC analysis of **4ae** (racemic)

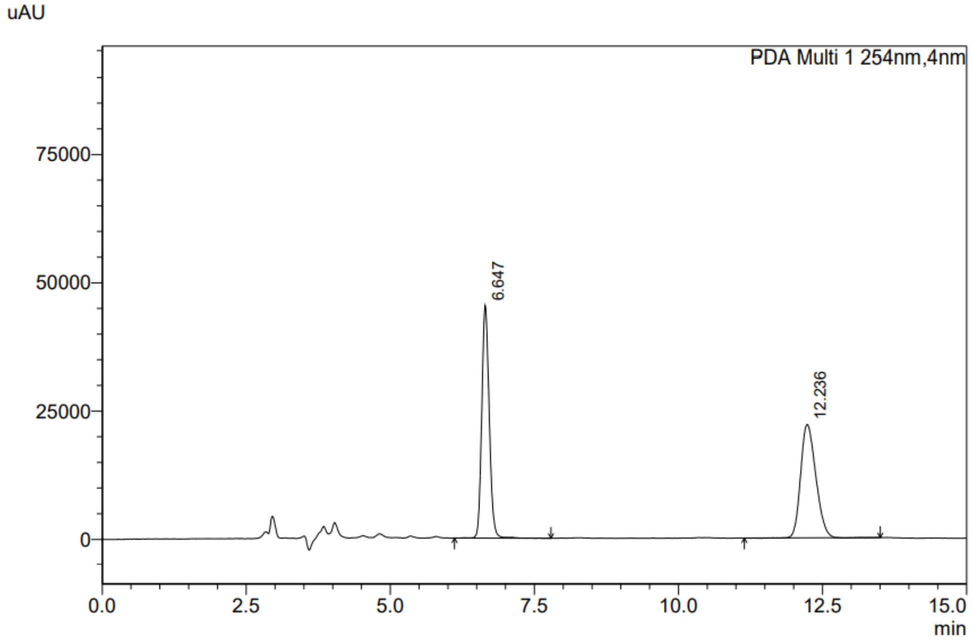

Peak Table

PDA Ch1 254nm

Peak#	Ret. Time	Area	Height	Area%
1	6.647	408525	45303	50.327
2	12.236	403224	22093	49.673
Total		811749	67396	100.000

Supplementary Figure 147 ^{1}H NMR of **5**

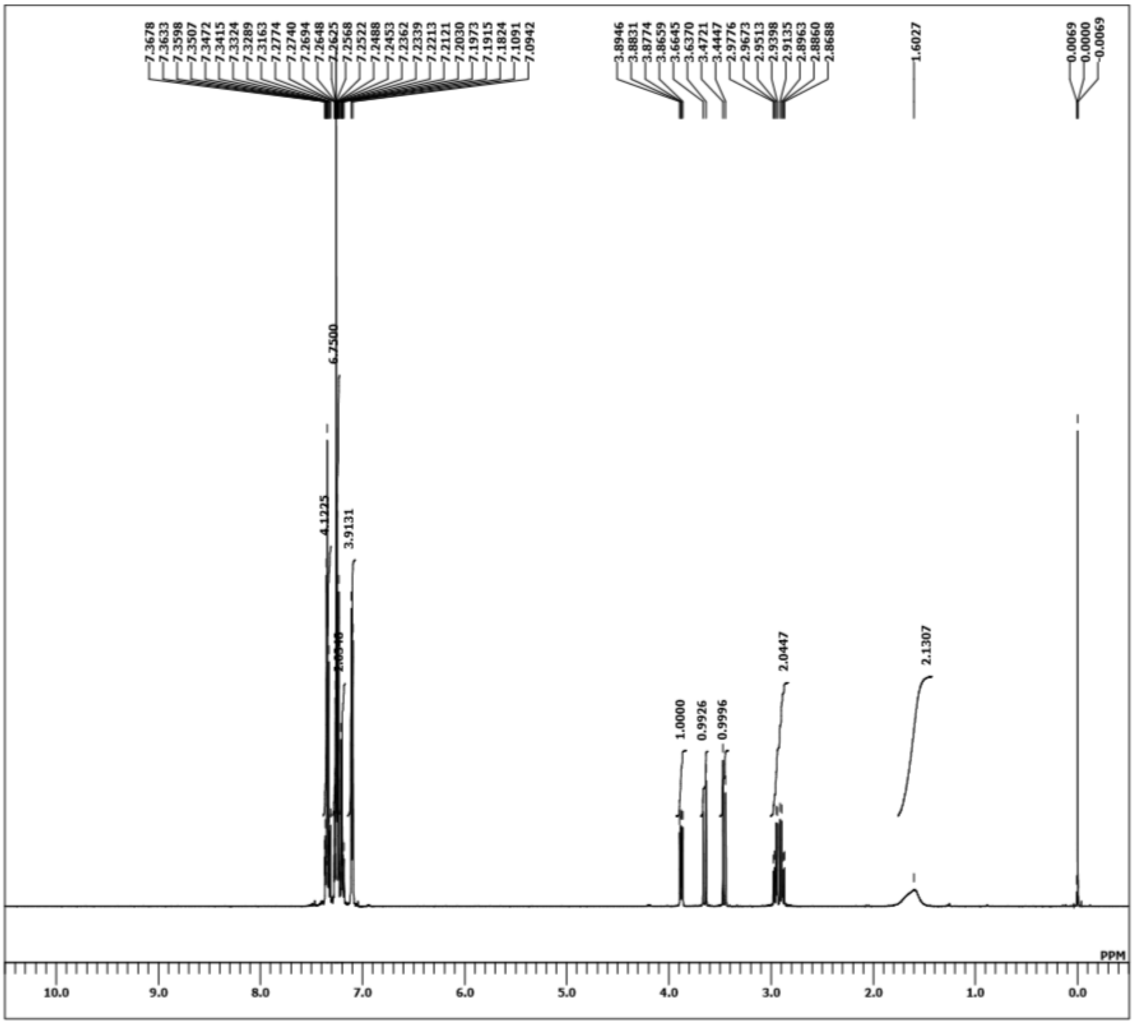

Supplementary Figure 148 ^{13}C NMR of **5**

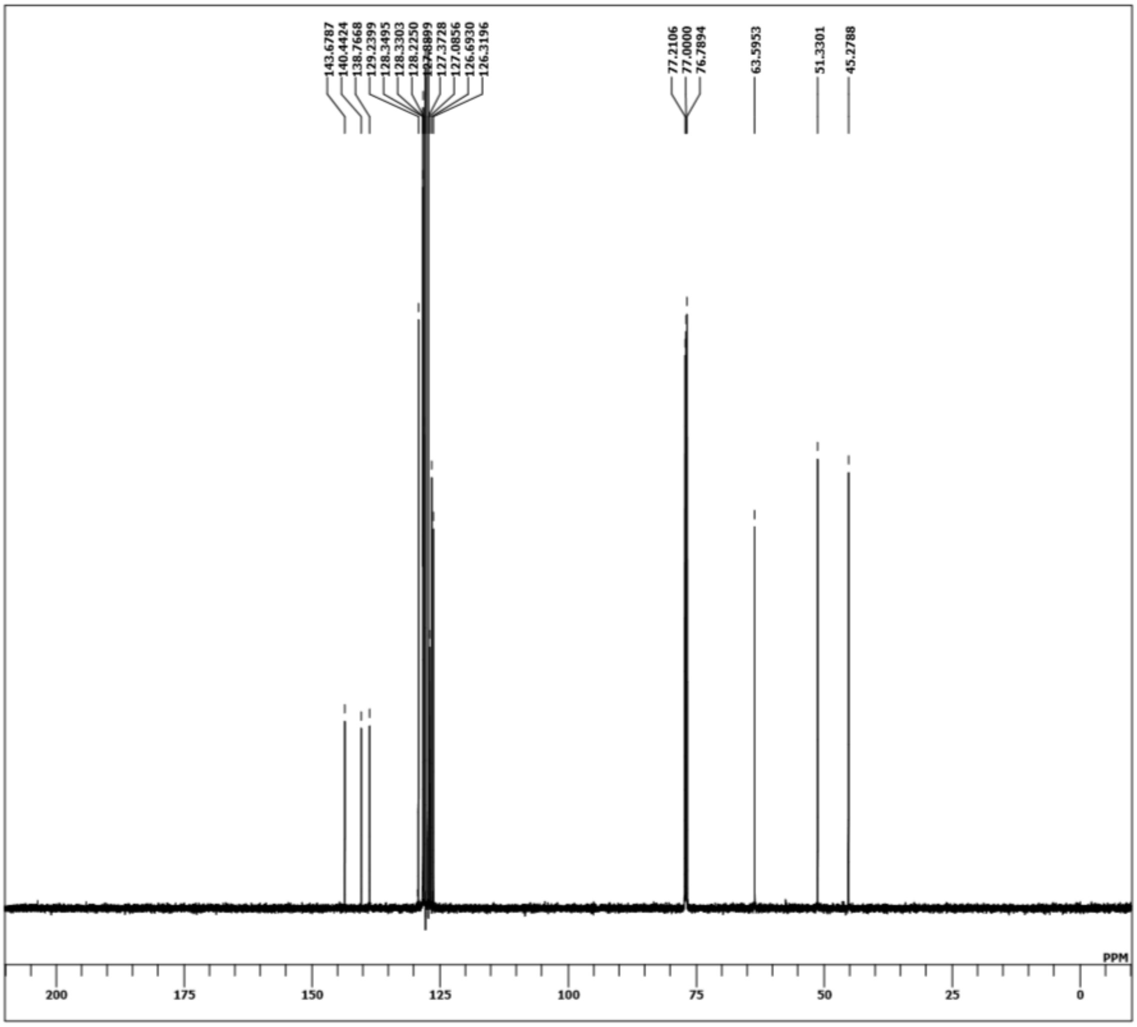

Supplementary Figure 149 ^{1}H NMR of **6**

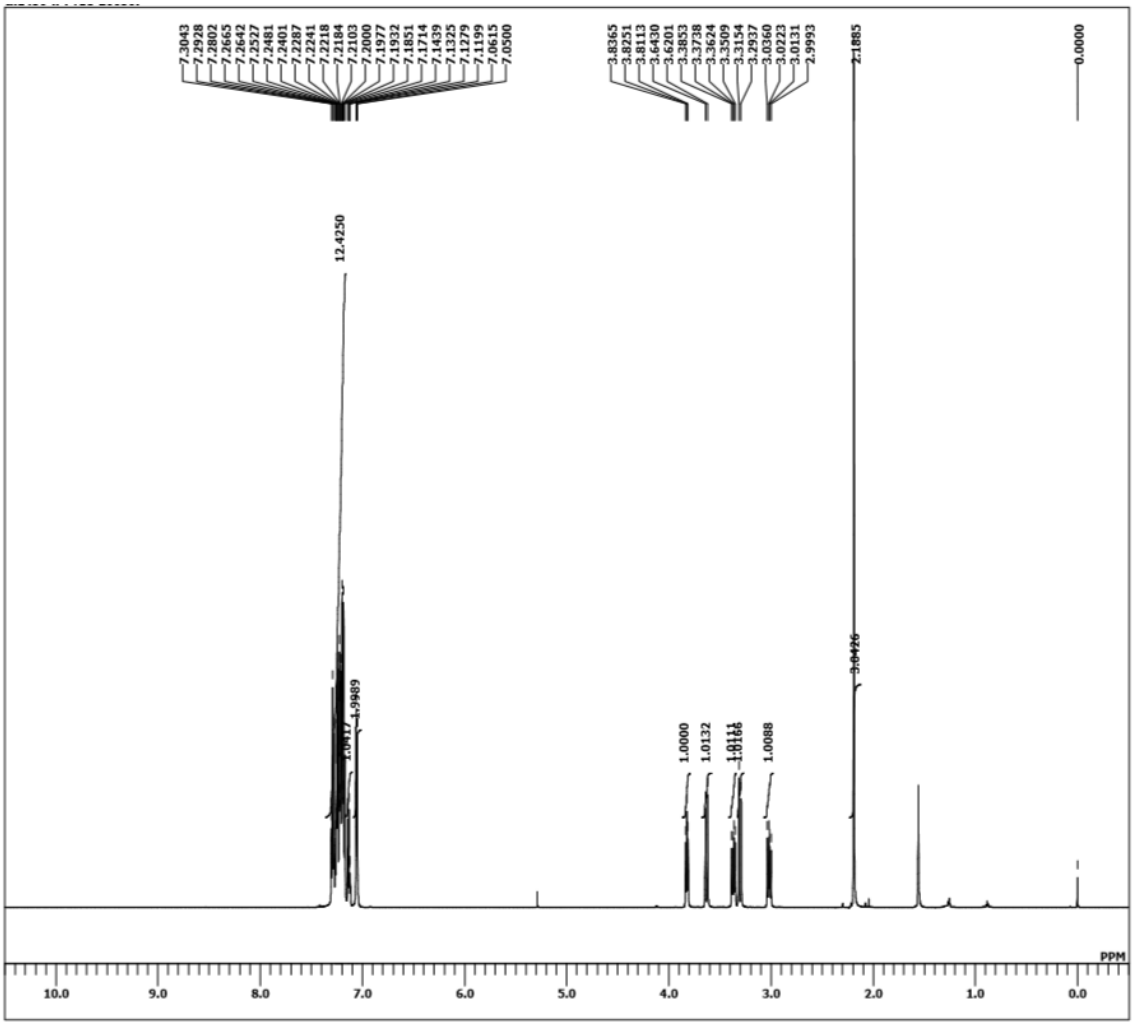

Supplementary Figure 150 ^{13}C NMR of **6**

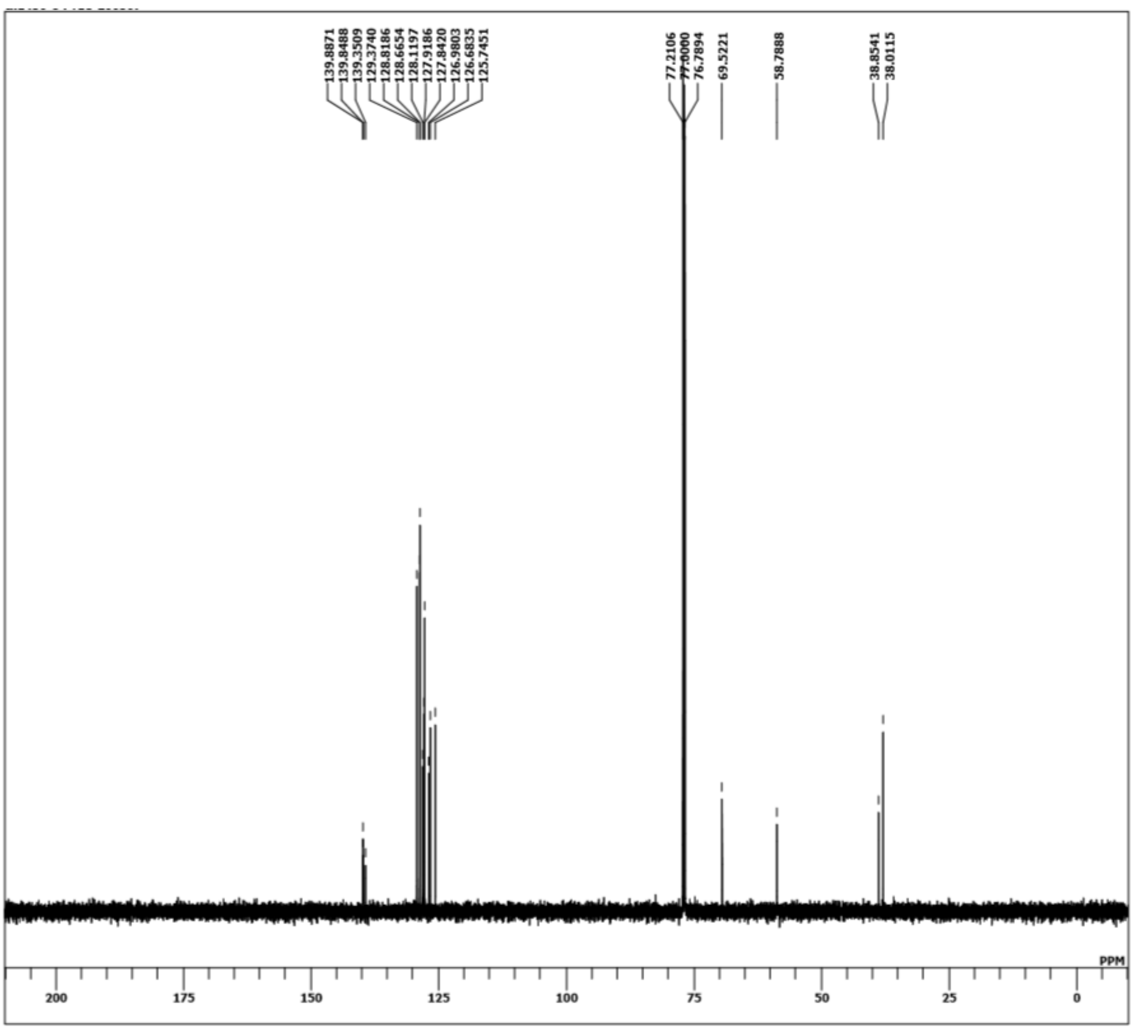

Supplementary Figure 151 HPLC analysis of **6**

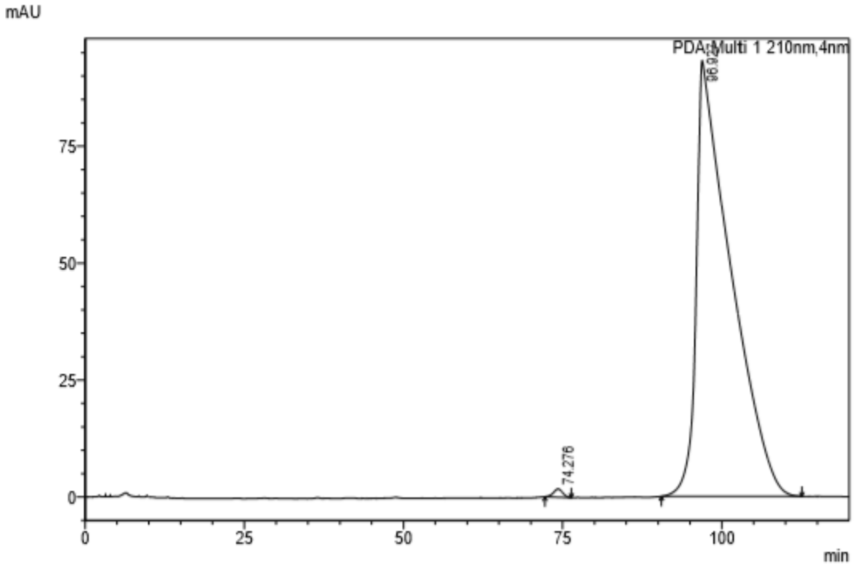

Peak Table

PDA Ch1 210nm

Peak#	Ret. Time	Area	Height	Area%
1	74.276	176614	1747	0.500
2	96.927	35149148	93196	99.500
ΣŒv		35325762	94943	100.000

Supplementary Figure 152 HPLC analysis of **6** (racemic)

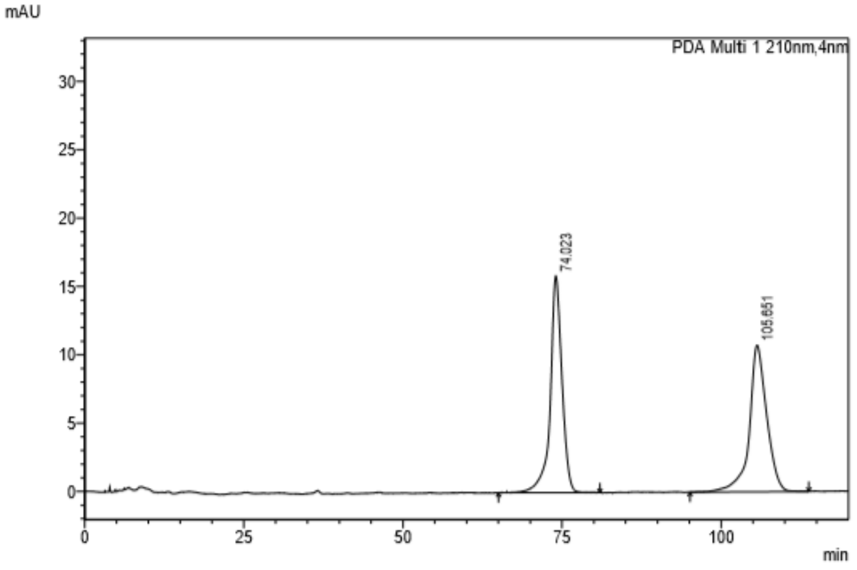

Peak Table

PDA Ch1 210nm

Peak#	Ret. Time	Area	Height	Area%
1	74.023	2044488	15868	49.954
2	105.651	2048291	10716	50.046
ΣŒv		4092779	26584	100.000

Supplementary Figure 153 ^{1}H NMR of **7**

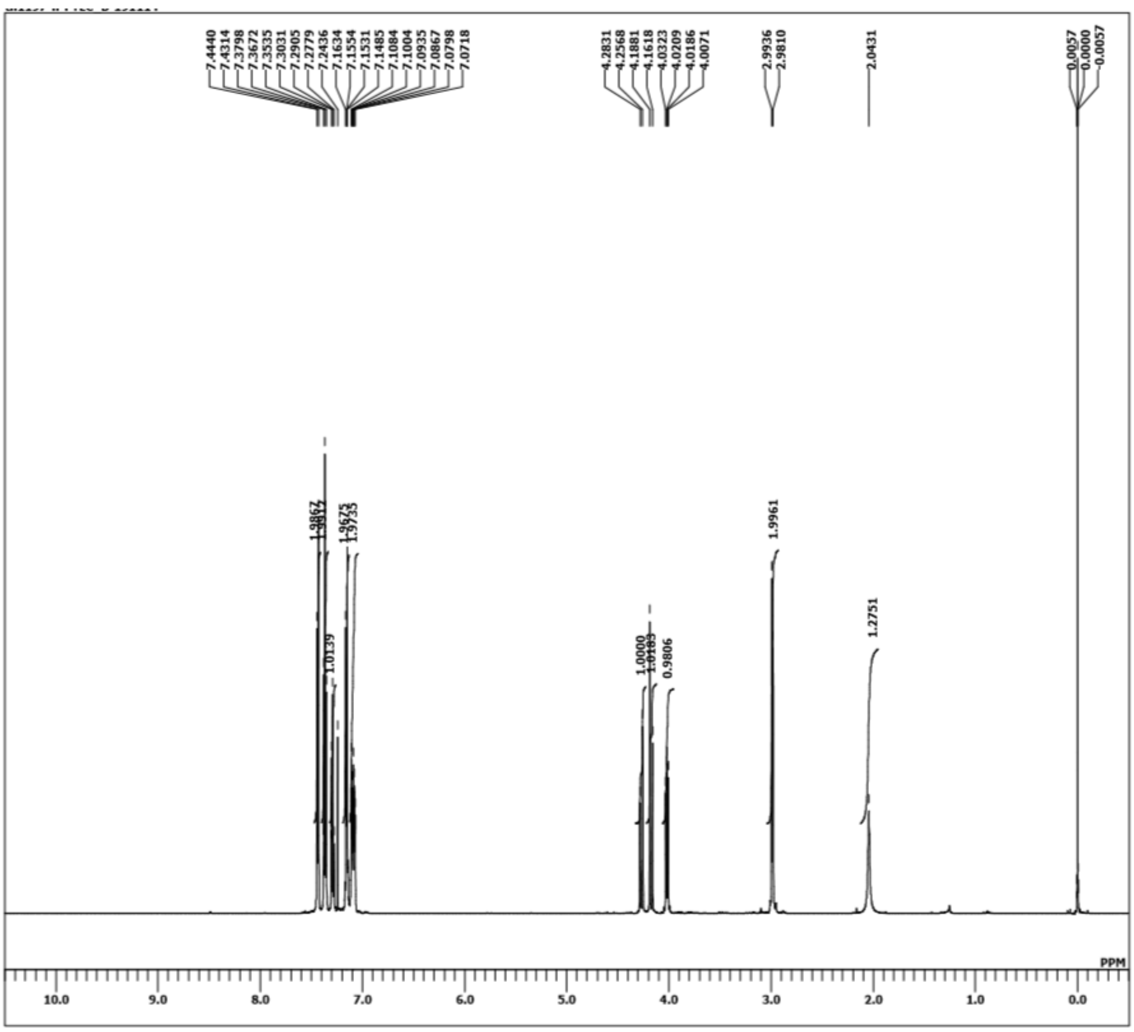

Supplementary Figure 154 ^{13}C NMR of **7**

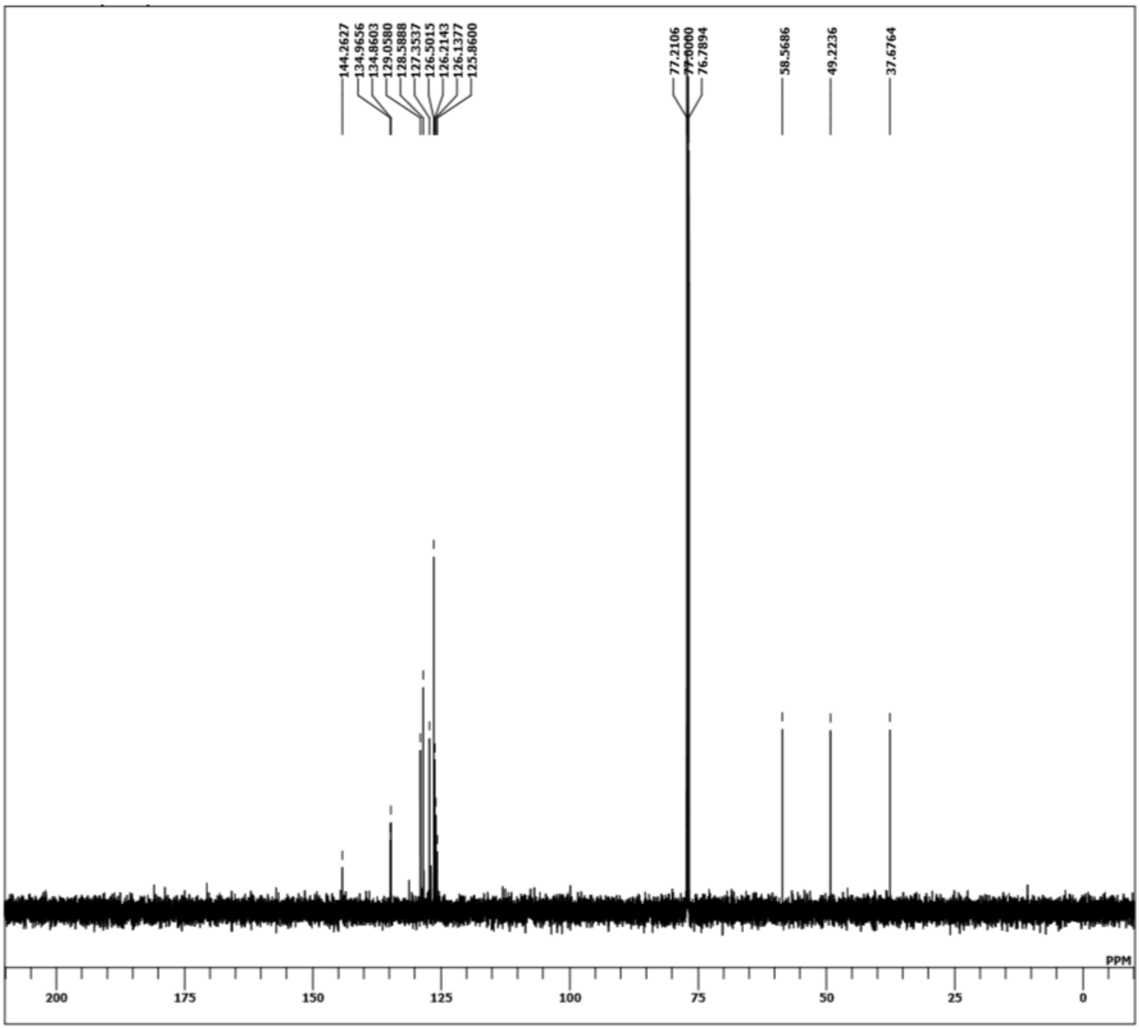

Supplementary Figure 155 HPLC analysis of **7**

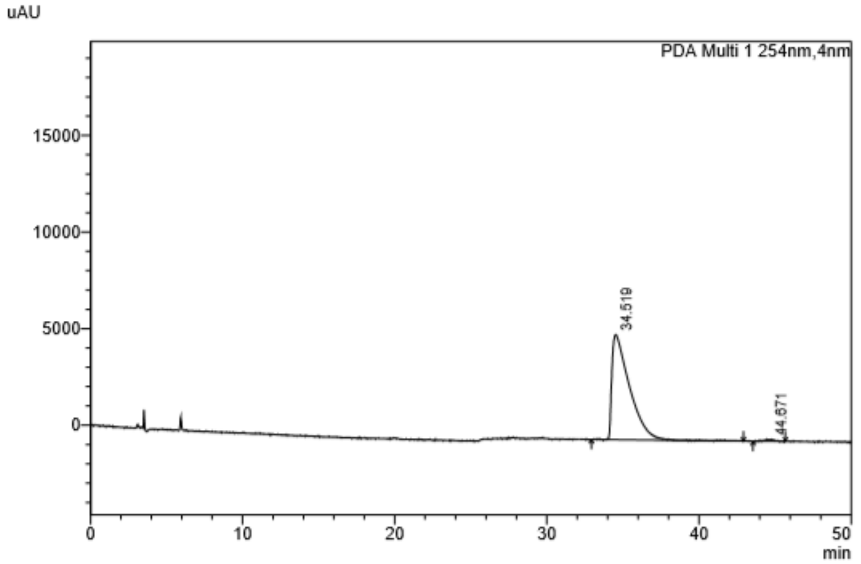

Peak Table

PDA Ch1 254nm

Peak#	Ret. Time	Area	Height	Area%
1	34.519	440543	5438	99.062
2	44.671	4169	94	0.938
Total		444712	5532	100.000

Supplementary Figure 156 HPLC analysis of **7** (racemic)

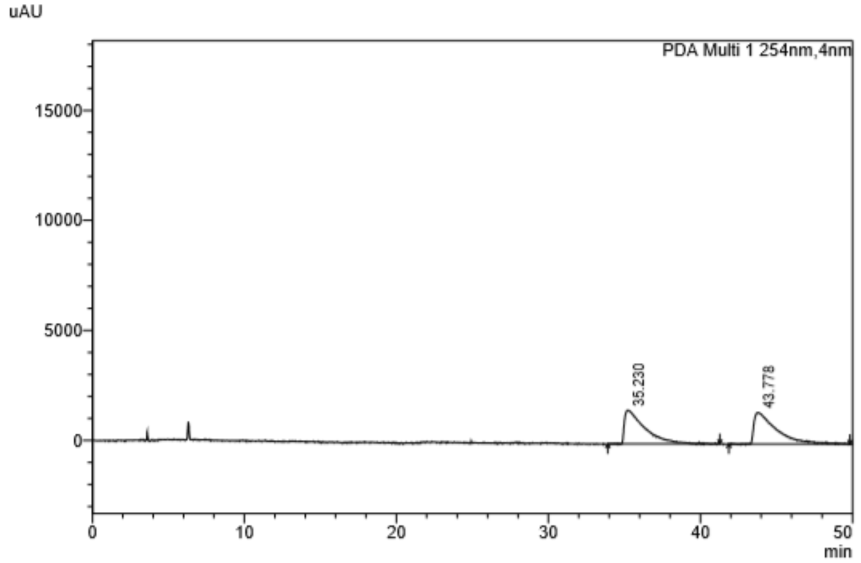

Peak Table

PDA Ch1 254nm

Peak#	Ret. Time	Area	Height	Area%
1	35.230	139783	1523	49.015
2	43.778	145400	1422	50.985
Total		285183	2945	100.000
